# Targeting CDK4/6 in Cancer: Molecular Docking and Cytotoxic Evaluation of *Thottea siliquosa* Root Extract

**DOI:** 10.3390/biomedicines13071658

**Published:** 2025-07-07

**Authors:** Maruthamuthu Rathinam Elakkiya, Mohandas Krishnasreya, Sureshkumar Tharani, Muthukrishnan Arun, L. Vijayalakshmi, Jiseok Lim, Ayman A. Ghfar, Balasundaramsaraswathy Chithradevi

**Affiliations:** 1 Department of Botany, PSGR Krishnammal College for Women, Coimbatore 641004, Tamil Nadu, India; 21phdby002@psgrkcw.ac.in (M.R.E.); 21phdby004@psgrkcw.ac.in (M.K.); 2 GRG BIRAC EYUVA Centre, PSGR Krishnammal College for Women, Coimbatore 641004, Tamil Nadu, India; 3 Department of Biotechnology, Bharathiar University, Coimbatore 641046, Tamil Nadu, India; 4 Department of Mechanical Engineering, Yeungnam University, Gyeongsan-si 38541, Republic of Korea; 5 Department of Chemistry, College of Science, King Saud University, Riyadh 11451, Saudi Arabia

**Keywords:** *Thottea siliquosa*, CDK4/6 inhibition, molecular docking, ADME profiling, cytotoxicity, cell migration

## Abstract

**Background**: Cyclin-dependent kinases 4 and 6 (CDK4/6) are pivotal regulators of the cell cycle, whose dysregulation is closely linked to cancer progression. While synthetic CDK4/6 inhibitors such as Palbociclib and Ribociclib are clinically effective, their use is limited by significant adverse effects. **Methods:** In this study, the aqueous root extract of *Thottea siliquosa*, a traditionally used medicinal plant, was evaluated for its potential as a natural CDK4/6 inhibitor. Phytochemical profiling using GC-MS identified bioactive compounds, which were subsequently subjected to molecular docking, ADME prediction, and in vitro cell-based assays using HCT116 and L929 cells. **Results:** The docking results revealed that Isocorydine (−7.4 kcal/mol for CDK4 and −7.2 kcal/mol for CDK6) and Thunbergol (−6.5 kcal/mol for CDK4 and −7.0 kcal/mol for CDK6) exhibited promising binding affinities comparable to standard CDK inhibitors, Palbociclib (−7.2, −8.3 kcal/mol) and Ribociclib (−7.1, −8.1 kcal/mol). Among the other tested natural compounds, Squalene (−7.1 kcal/mol for CDK4) and 2-palmitoylglycerol (−5.2 kcal/mol for CDK4, −4.9 kcal/mol for CDK6) demonstrated moderate binding affinities. ADME analysis confirmed favorable drug-like properties with minimal toxicity alerts. The extract displayed dose-dependent cytotoxicity with an IC_50_ of 140 μg/mL and reduced cell migration in HCT116 cells, indicating potential anti-proliferative effects. These findings suggest that *T. siliquosa* root extract, through synergistic phytochemical interactions, holds promise as a multi-targeted, plant-based therapeutic candidate for CDK4/6-associated cancers, warranting further in vitro and in vivo validation.

## 1. Introduction

Cyclin-dependent kinases (CDKs) are essential regulatory molecules that govern cell cycle progression by modulating cellular growth and proliferation. Among them, CDK4 and CDK6 play critical roles in driving the transition from the G1 to S phase of the cell cycle [1,2,3]. During the process of malignant formation, the proto-oncogene cyclin D1 binds with CDK4 and CDK6, forming active complexes that promote uncontrolled cell proliferation, an underlying hallmark of cancer development [4,5]. Consequently, targeting CDK4 and CDK6 has emerged as a promising strategy in anticancer drug development.

Despite advances in chemotherapy and radiotherapy, these conventional treatments are often limited by high costs and severe side effects. In recent years, medicinal plants have gained increasing attention as potential alternatives in cancer therapy, offering diverse bioactive compounds with therapeutic potential. Notably, approximately 50% of commercially available antineoplastic drugs are plant-derived, including well-known compounds such as leucovorin, vincristine, taxol, camptothecin, and podophyllotoxin [6,7]. The adverse effects and resistance associated with synthetic chemotherapeutic agents further emphasize the need for safer, targeted therapies. In this regard, medicinal plants, with their broad spectrum of pharmacologically active compounds, present valuable candidates for drug discovery, often exhibiting fewer side effects and enhancing the safety margin [8].

*Thottea siliquosa*, a medicinal shrub belonging to the family Aristolochiaceae, native to India and Sri Lanka, holds significant value in Ayurvedic medicine and is traditionally used by several tribal communities to manage various ailments. The roots of *T. siliquosa* are known for managing headaches, coughs, and chest pain, and are even used as an antidote for poisonous bites. Pharmacological studies have further reported a wide range of biological activities, including anticancer, antimicrobial, antioxidant, antigenotoxic, anti-inflammatory, and cytotoxic activities [9,10]. Given the therapeutic potential of *T. siliquosa*, exploring its phytochemical constituents for their ability to modulate molecular targets such as CDK4 and CDK6 offers a promising direction for anticancer research.

Molecular docking provides an efficient computational strategy to predict and visualize interactions between bioactive compounds and target proteins, enabling the identification of potential lead molecules [11]. In this study, *T. siliquosa* root extract was subjected to phytochemical profiling using GC-MS, followed by molecular docking, ADME prediction, cytotoxicity evaluation, cell cycle analysis, and cell migration assays to comprehensively assess its potential as a natural source of CDK-4 and CDK-6 inhibitors with possible therapeutic applications in cancer.

## 2. Materials and Methods

### 2.1. Collection and Authentication of Plant Material

*T. siliquosa* saplings were collected from Vithura town, Nedumangad, located in Thiruvananthapuram district, Kerala, India, in May 2023 and maintained in a greenhouse. The specimen was duly authenticated at the Botanical Survey of India, Southern Regional Centre, Coimbatore-03. The same specimen was preserved as a herbarium for future reference.

### 2.2. Phytochemical Extraction

Fresh roots were collected in January 2024, washed, shade dried, and powdered. Twenty grams of the powdered sample was decocted in distilled water for 20 min, filtered, and dried in a hot air oven. It was then stored in vials for further analysis. Aqueous extraction mimics the traditional preparation methods of *T. siliquosa*, which minimizes the use of organic solvents and avoids toxic residues [12].

### 2.3. GC-MS Analysis

GC-MS analysis was conducted using a gas chromatography system (Model: CH-GCMSMS02, 8890) integrated with a 7000 GC/TQ mass spectrometer. A capillary column (30 m × 250 mm × 0.5 μm) was used with helium as the carrier gas. Methanol was used as the solvent. The thermal program involved gradual heating to 280 °C over a total run time of 38 min. The mass range was set to 30–900 *m*/*z*, and compounds were identified via NIST library comparison.

### 2.4. Cell Culture and Cytotoxicity (MTT Assay)

#### 2.4.1. HCT116 Cell Lines

The human colorectal carcinoma cell line (HCT116) was selected for this study due to its well-characterized cell cycle regulation and high CDK activity, making it a suitable model for evaluating the effects of CDK inhibitors [13]. The cells were obtained from NCCS, Pune, India, and were cultured in Dulbecco’s Modified Eagle’s Medium (DMEM, HiMedia Laboratories Pvt. Ltd., Mumbai, India), which was supplemented with 10% Fetal Bovine Serum (Gibco, Thermo Fisher Scientific, Waltham, MA, USA), 1% Sodium Bicarbonate, and 1% Sodium pyruvate (HiMedia Laboratories Pvt. Ltd., Mumbai, India). The cells were maintained in a humidified 5% CO_2_ atmosphere at 37 °C for further experiments. MTT (tetrazolium salt; HiMedia Laboratories Pvt. Ltd., Mumbai, India) was used to analyze the effective cytotoxic concentration of the root extract. HCT116 cells were seeded (1 × 10^4^ cells per well) in a 96-well plate. The cells were then incubated for 24 h. Cells were treated with the extract (10 concentrations from 100 μg/mL to 1000 μg/mL) and incubated for another 24 h. Then, 100 μL of complete media containing MTT (0.5 mg/mL) was added to each well, the cells were incubated for 5 h, the media was carefully removed, and the formazan crystals were dissolved in 100 μL DMSO for 30 min. The absorbance was then obtained on a microplate reader at 570 nm and 650 nm, and the IC_50_ value was calculated [14].

#### 2.4.2. L929 Cell Lines

L-929 fibroblast cell lines, obtained from NCCS, Pune, India, were cultured in DMEM medium supplemented with 10% FBS, penicillin, and streptomycin, and maintained at 37 °C with 5% CO_2_. Cells were seeded in 96-well plates and treated with varying concentrations (5–100 μg/mL) of the extract. The MTT assay was performed to evaluate cytotoxicity following incubation with MTT reagent, solubilization with acidified isopropanol, and absorbance measurement at 650 nm.

### 2.5. Cell Migration Assay

#### 2.5.1. HCT116 Cell Lines

To assess the anti-migratory effect of the extract, a wound healing assay was performed. HCT116 cells were seeded (2 × 10^5^ cells per well) in a 24-well plate and then incubated for 24 h; a scratch was made in the middle by a 200 μL pipette tip after 24 h of incubation of cells to reach 90% confluency. The cells were washed with PBS to remove any floating cells after wound creation. Only 50% of the IC_50_ concentration was administered to reduce cytotoxic impact, considering that the full IC_50_ concentration is cytotoxic to 50% of cells. The cells were then incubated for another 24 h, while the untreated cells served as a control. The potential of the cells to move from the edge to the wounded area is observed for three time periods (0 h, 24 h, and 36 h).

#### 2.5.2. L929 Cell Lines

L-929 cells were seeded in 6-well plates to confluence, and a scratch was made using a sterile pipette tip. The cell surface was then washed with serum-free culture medium three times to remove dislodged cells. Wound closure was monitored by collecting digitized images at 0 and 24 h after the scratch was made.

### 2.6. Cell Cycle Analysis

The cell cycle arrest was evaluated in the HCT116 cell line treated with IC_50_ concentrations of the extract. 1.2 × 10^5^ HCT116 cells were seeded in each well of the 24-well plate and grown with DMEM media for 24 h under standard cell culture conditions till they reached 80–90% confluency. The cells were treated with the extract and incubated for 24 h, while untreated cells served as controls. Then, the trypsinized cells were washed twice with PBS, and the cells were suspended in PI staining solution (50 μg/mL propidium iodide, 200 μg/mL DNase-free RNase, 4 mM sodium citrate, 0.1% Triton X-100) and incubated in the dark. The fluorescently labeled cells were then analyzed using flow cytometry (DxFLEX flow cytometer, Beckman Coulter, Brea, CA, USA).

### 2.7. Selection and Preparation of Ligand Molecule

The bioactive compounds identified through GC-MS and FDA-approved CDK inhibitors (Palbociclib and Ribociclib) were retrieved from the PubChem database (http://pubchem.ncbi.nlm.nih.gov/) (accessed on 28 March 2025) and saved in a structure data file format (*.sdf file). These were then converted into protein data bank file format (*.pdb file) using OpenBabel 2.4.1 software and prepared for docking [15].

### 2.8. Target Retrieval

The crystal structures of CDK4 (PDB Code: 2W9F) and CDK6 (PDB Code: 1JOW) were obtained from the RCSB Protein Data Bank (https://www.rcsb.org) (accessed on 28 March 2025).

### 2.9. Molecular Docking

Molecular docking was performed using Autodock 4.2 and Autodock Vina. The target proteins were prepared by removing water molecules from the target protein. In due course, polar hydrogens, Kollman charges, and Gasteiger charges were added, and AD4-type atoms were assigned. The torsion tree was set up for the ligand. These prepared proteins and ligands were saved in PDBQT format (*.pdbqt file). Blind docking was carried out, considering the entire protein molecule as the binding site. The results were analyzed based on binding energies [16,17].

### 2.10. ADME Profiling

SwissADME (http://www.swissadme.ch/) (accessed on 11 February 2025) was used to predict ADME properties, evaluating absorption, distribution, metabolism, and excretion potential [18].

### 2.11. Statistical Analysis

The mean ± standard deviation (SD) was calculated for all experiments conducted in triplicate. IC_50_ values were estimated from dose–response curves, and all plots were prepared using Microsoft Excel.

## 3. Results

### 3.1. GC-MS Analysis

**Figure 1:**
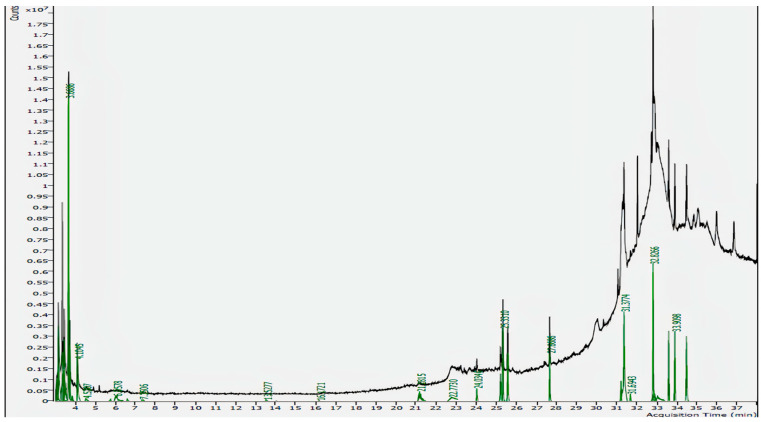
GC-MS spectrum of aqueous root extract of *Thottea siliquosa* showing peaks of bioactive compounds.

GC-MS analysis identified seven phytochemicals in the *T. siliquosa* aqueous root extract, including 2-palmitoylglycerol, Hentriacontane, Isocorydine, Squalene, Methyl Palmitate, Thunbergol, and Methyl Stearate. The compounds were analyzed for their retention time (RT), molecular formula, molecular mass, and peak area percentage (Figure 1, Table 1). Notably, 2-palmitoylglycerol exhibited the highest peak area (19.23%), followed by Hentriacontane (15.97%) and Isocorydine (8.73%).

### 3.2. Cell Viability and Cytotoxicity (MTT Assay)

#### 3.2.1. HCT116 Cell Lines

**Figure 2:**
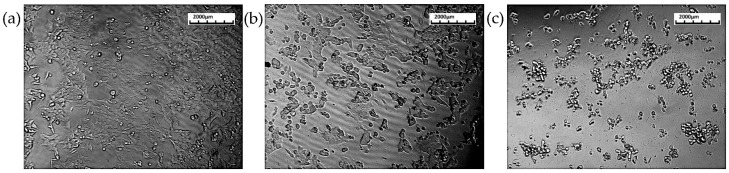
Cytotoxicity assay showing morphological changes in HCT116 cells. Subfigures (**a**–**c**) depict cell death after 24 h in control, 100 μg/mL-treated, and 200 μg/mL-treated groups, respectively.

**Figure 3:**
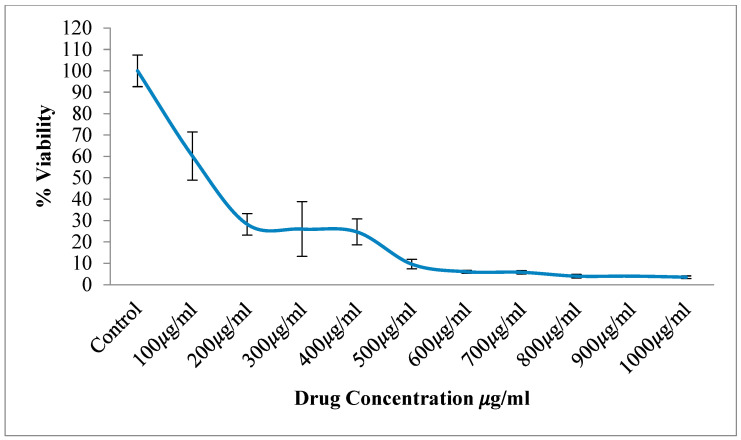
Dose–response curve showing % viability of HCT116 cells treated with *T. siliquosa* root extract.

The cytotoxic effect of the aqueous root extract of *T. siliquosa* was assessed using the MTT assay on HCT116 cell lines (Figure 2). The results demonstrated a dose-dependent cytotoxicity during the 24 h treatment with increasing concentrations of the extract (100 to 1000 μg/mL). The extract exhibited potent cytotoxic effects, reducing cell viability to below 10% at higher concentrations. The calculated IC_50_ value was 140 μg/mL, indicating a moderate to strong cytotoxic potential of the crude extract against HCT116 cells (Figure 3, Table 2).

#### 3.2.2. L929 Cell Lines

**Figure 4:**
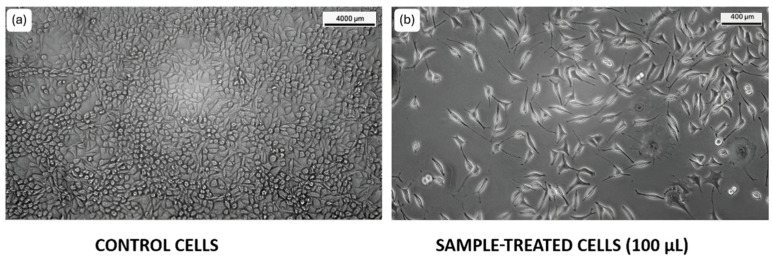
Cytotoxicity assay showing morphological changes in L929 cells. Subfigures (**a**,**b**) depict control and 100 μg/mL-treated group after 24 h incubation.

The cytotoxic effect of the *Thottea siliquosa* root extract on L-929 fibroblast cells was evaluated using the MTT assay. The results revealed a dose-dependent reduction in cell viability. At a low concentration of 5 μg/mL, cell viability remained high (90.66%), indicating minimal impact on cell metabolic activity. As the extract concentration increased, a gradual decline in viability was observed, with the lowest value of 45.50% recorded at 100 μg/mL (Figure 4). However, the extract did not achieve 50% inhibition (IC_50_) within the tested concentration range. It is important to note that the observed decrease in OD values may partly reflect reduced metabolic activity due to serum starvation or other stress conditions rather than direct cytotoxic effects. These findings suggest that the extract exhibits low to moderate toxicity towards normal fibroblast cells and appears relatively safe at lower concentrations.

### 3.3. Cell Migration Assay

#### 3.3.1. HCT116 Cell Lines

**Figure 5:**
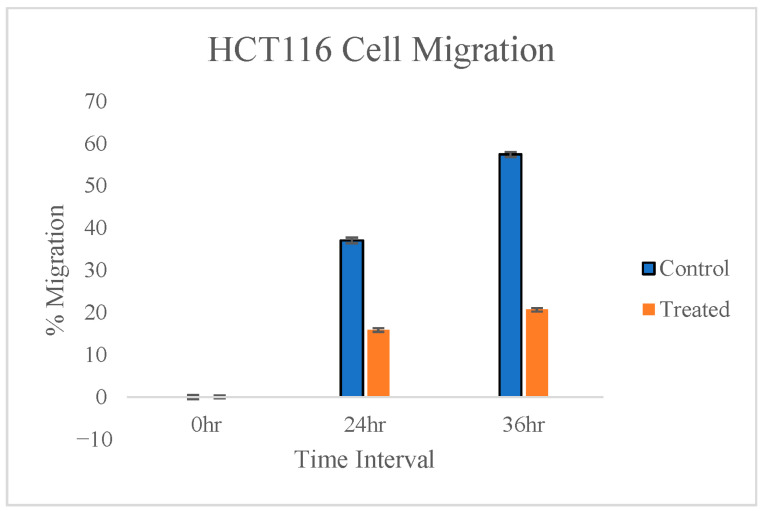
% Cell migration in HCT116 cells induced by *T. siliquosa* root extract.

**Figure 6:**
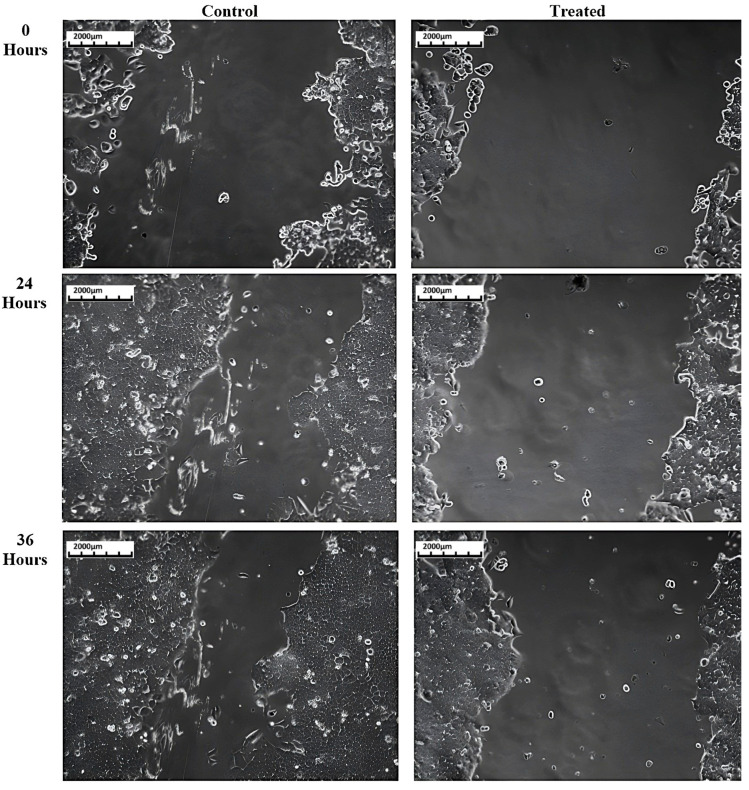
Cell migration assay images at 0 h, 24 h, and 36 h post-treatment with *T. siliquosa* extract (70 μg/mL).

The anti-migratory potential of *T. siliquosa* root extract was evaluated through a cell migration (scratch) assay on HCT116 cells. The cells were treated only with 50% of the IC_50_ concentration, which is 70 μg/mL. This minimizes the cytotoxic effect on cells, as the IC_50_ concentration (140 μg/mL) can be cytotoxic to 50% of the cell population. A reference line was drawn in the middle of 24-well plates to ensure images were taken at the same field for the three time periods where the wound was created. The wound made in the human colorectal carcinoma cells (HCT116) is observed under a phase contrast microscope at three time intervals: 0 h, 24 h, and 36 h (Figure 5). Images were analyzed with ImageJ software version 1.54p to find the percentage of migration between the control and treated cells (Figure 6). The results reveal that the extract is capable of significantly inhibiting the migration of HCT116 cells (Table 3).

#### 3.3.2. L929 Cell Lines

**Figure 7:**
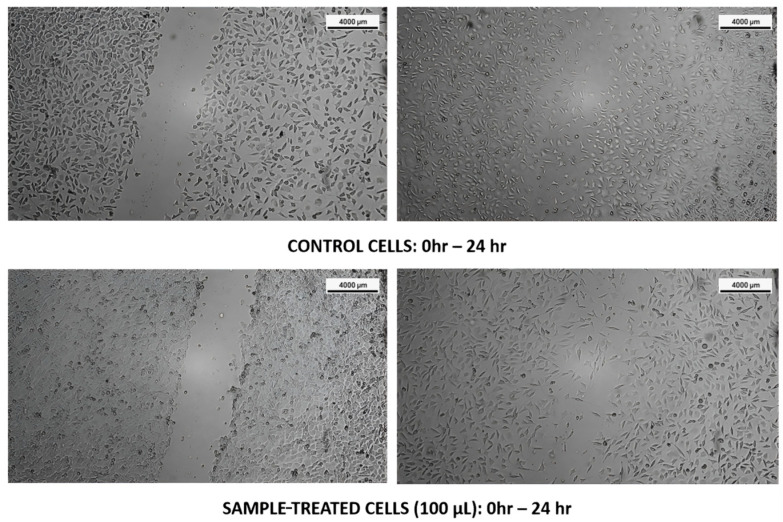
Cell migration assay images at 0 h and 24 h post-treatment with *T. siliquosa* extract (100 μg/mL).

The cell migration potential of *T. siliquosa* root extract (100 μg/mL) was evaluated using the wound scratch assay on L-929 fibroblast cells. The assay revealed that after 24 h, untreated control cells exhibited significant migration into the scratch area, consistent with normal proliferative and migratory behavior. Similarly, cells treated with the root extract also demonstrated marked migration into the wound region, indicating that the extract did not inhibit the migratory capacity of these normal fibroblasts. This observation suggests that the extract is non-toxic to normal cells and selectively inhibits malignant cell proliferation, aligning with its proposed role as a natural CDK4/6 inhibitor (Figure 7).

### 3.4. Cell Cycle Analysis

**Figure 8:**
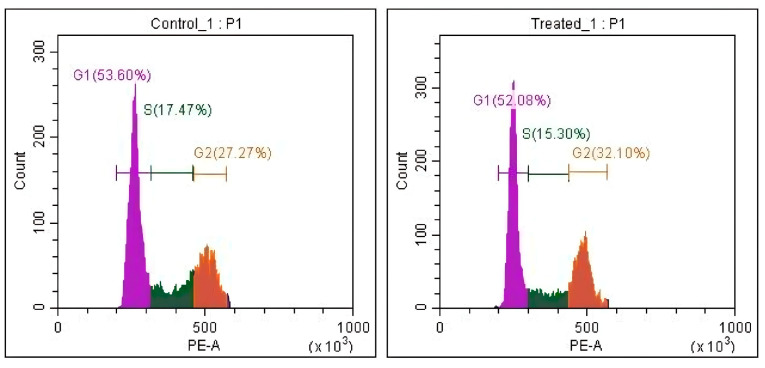
Differential cell cycle distribution in HCT116 cells under control cells exhibited a typical distribution with the majority of cells in G1 phase. Treated cells showed a marked increase in the G2/M population, indicating cell cycle arrest.

**Figure 9:**
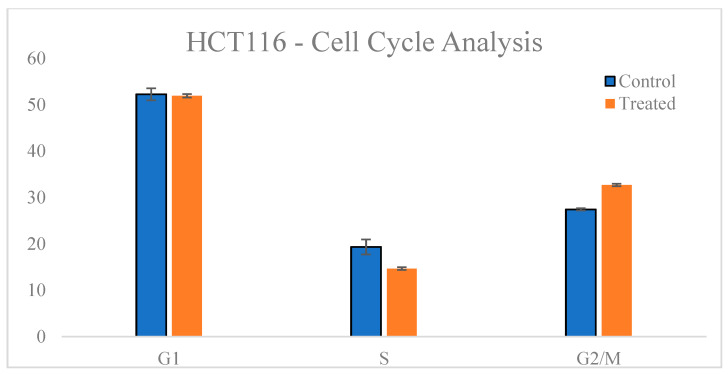
Quantitative bar graphs represent the percentage of cells in each phase (G1, S, and G2/M). The minimal change in G1 population suggests partial CDK4/6 inhibition, while the increase in G2/M points to involvement of other regulatory proteins such as CDK1 and Cyclin B1.

To further investigate the mechanism underlying the cytotoxic effects of *T. siliquosa* root extract, cell cycle analysis was performed on HCT116 cells treated with the IC_50_ concentration (140 μg/mL) for 24 h. Flow cytometry following propidium iodide (PI) staining revealed a notable accumulation of cells in the G2/M phase of the cell cycle compared to the control. In untreated cells, the average distribution was 52.25% (G1 phase), 19.34% (S phase), and 27.44% (G2/M checkpoint). Upon treatment, the G2/M population increased to 32.7%, accompanied by a decrease in the S phase population to 14.67% (Table 4, Figure 8 and Figure 9). This shift indicates that the extract induces cell cycle arrest at the G2/M checkpoint, thereby suppressing cell division. Although CDK4/6 is primarily involved in the G1-S transition, the minimal change in G1 phase suggests partial inhibition, but not complete suppression, of CDK4/6 activity. The prominent G2/M arrest indicates that the extract likely affects additional regulatory kinases such as CDK1 or Cyclin B1, supporting a potential multi-targeted mechanism of anticancer action.

### 3.5. Molecular Docking

Molecular docking studies revealed significant interactions between phytochemicals from *T. siliquosa* and the target proteins CDK4 (2W9F) and CDK6 (1JOW). Among the tested compounds, Isocorydine exhibited the highest docking scores, recording binding energies of −7.4 kcal/mol with CDK4 and −7.2 kcal/mol with CDK6. Thunbergol showed strong interaction with CDK6 (−7 kcal/mol) and moderate interaction with CDK4 (−6.5 kcal/mol). Squalene demonstrated notable docking scores as well, with binding energies of −7.1 kcal/mol against CDK4 and −4.9 kcal/mol against CDK6. Other phytochemicals, such as Methyl Palmitate, Methyl Stearate, 2-palmitoylglycerol, and Hentriacontane, displayed weaker interaction (Table 5).

**Figure 10:**
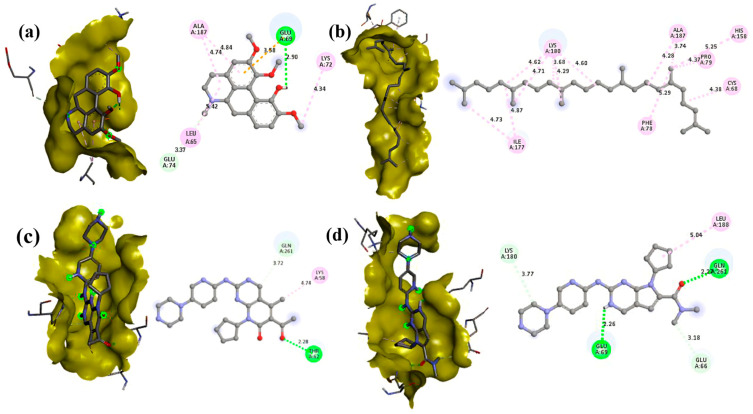
Docking complexes and interactions between the CDK4 protein and the ligands with the best binding energies. Subfigures (**a**,**b**) show the interactions of Isocorydine (−7.4) and Squalene (−7.1), respectively, whereas (**c**,**d**) depict the interactions of the standard drugs Palbociclib (−7.2) and Ribociclib (−7.1).

**Figure 11:**
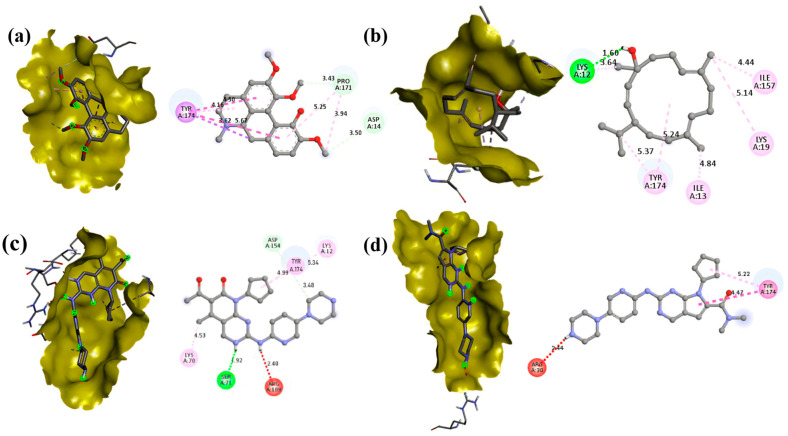
Docking complexes and interactions between the CDK6 protein and the ligands with the best binding energies. Subfigures (**a**,**b**) show the interactions of Isocorydine (−7.2) and Thunbergol (−7), respectively, whereas (**c**,**d**) depict the interactions of the standard drugs Palbociclib (−8.3) and Ribociclib (−8.1).

In comparison, the FDA-approved CDK4/6 inhibitors Palbociclib exhibited −7.2 kcal/mol (CDK4) and −8.3 kcal/mol (CDK6) binding energies, and Ribociclib displayed −7.1 kcal/mol (CDK4) and −8.1 kcal/mol (CDK6) binding energies, respectively. Additionally, the specific amino acid residues interacting with the phytochemicals and the control drugs are detailed in Table 6 and Table 7. Notably, the docking scores of key phytochemicals, such as Isocorydine, Thunbergol, and Squalene, were comparable to those of the control drugs. This suggests that these phytochemicals might act synergistically with existing CDK4/6 inhibitors, offering enhanced therapeutic potential. The strong and stable interactions of Isocorydine, Thunbergol, and Squalene with the active sites of CDK4/6 highlight their promise as natural drug candidates for further development and exploration (Figure 10 and Figure 11).

### 3.6. ADME Profiling

In silico ADME (Absorption, Distribution, Metabolism, and Excretion) profiling, conducted using the SwissADME tool, evaluated the pharmacokinetic properties of the phytochemicals from *T. siliquosa* in comparison to the control drugs Palbociclib and Ribociclib. The analysis highlighted that the phytochemicals, such as Isocorydine, Methyl Palmitate, and Thunbergol, exhibit pharmacokinetic properties comparable to the control drugs, underscoring their potential as drug-like candidates. The phytochemicals complied with Lipinski’s Rule of Five (Ro5), demonstrating parameters like molecular weight, hydrogen bond donors/acceptors, lipophilicity, and topological polar surface area (TPSA) within favorable ranges, similar to that of the control drugs. Notably, Isocorydine and Methyl Palmitate exhibited high gastrointestinal (GI) absorption, mirroring the oral bioavailability characteristics of Palbociclib and Ribociclib. Furthermore, minimal blood–brain barrier (BBB) permeation was observed for Squalene and Hentriacontane, suggesting a reduced likelihood of central nervous system side effects, similar to the safety profile of the control drugs.

The absence of significant PAINS (Pan-assay Interference Compounds) alerts among the tested phytochemicals indicates a lower risk of non-specific interactions in bioassays, further reinforcing their drug-like potential (Table 8). Collectively, these attributes align the phytochemicals closely with established pharmacokinetic profiles of Palbociclib and Ribociclib, suggesting their potential not only as standalone therapeutic agents but also as complementary molecules that might enhance or synergize with existing CDK4/6 inhibitors.

## 4. Discussion

The present study provides scientific evidence supporting the potential of *T. siliquosa* root extract as a natural source of bioactive compounds with inhibitory effects on cyclin-dependent kinases CDK4 and CDK6, key regulators of cell cycle progression commonly implicated in various cancers [19]. GC-MS analysis of the aqueous root extract revealed seven major phytochemical compounds, each known for diverse pharmacological properties, including anticancer, antioxidant, anti-inflammatory, and antitumor effects (Table 9).

This phytochemical diversity highlights the therapeutic potential of *T. siliquosa* as a natural resource for drug discovery. Importantly, this study supports the concept of synergistic action, where the combined effects of multiple phytochemicals within the crude extract may enhance biological activity and mitigate potential side effects compared to isolated compounds [34,35].

Following the identification of bioactive compounds through GC-MS analysis, the crude extract was evaluated for biological activity. The cytotoxicity assay demonstrated a dose-dependent cytotoxic effect of the crude extract on HCT116 cancer cells, with an IC_50_ of 140 μg/mL. This dose-dependent profile indicated a potential therapeutic window where selective cytotoxicity can be harnessed against target cells while sparing normal cells [36]. The cell migration assay further indicated that the extract significantly inhibited cancer cell migration, as demonstrated by reduced wound closure in treated cells compared to the control [37]. This anti-migratory effect suggests potential anti-proliferative and anti-metastatic properties, aligning with the proposed mechanism of CDK4/6 inhibition, which is known to arrest the cell cycle and suppress cancer cell progression. Supporting this, the extract did not exhibit cytotoxic or anti-migratory effects on L929 murine fibroblast cells, which continued to proliferate and migrate normally in the scratch assay. These results suggest that the extract is non-toxic to normal cells, reinforcing its selective action against cancer cells.

Furthermore, flow cytometric analysis revealed that treatment with the extract demonstrated a marked accumulation of cells at the G2/M checkpoint alongside minimal change in G1 phase (53.60% to 52.08%). These findings suggest that while CDK4/6 inhibition was proposed based on molecular docking, the extract’s in vitro effect may not be limited to CDK4/6 alone. The observed G2/M arrest implies potential interference with other key regulators of cell cycle progression, such as CDK1, Cyclin B1, or PLK1, which are essential for G2/M transition. Given the complex nature of crude plant extracts, it is plausible that multiple cell cycle regulators are concurrently affected. Therefore, while partial inhibition of CDK4/6 cannot be excluded, the overall effect likely involves a multi-targeted mechanism. Further studies employing protein-level validation, like Western blotting, RT-qPCR, or kinase activity assays, are warranted to confirm the specific contribution of CDK4/6 inhibition to the extract’s anticancer activity [38,39].

Subsequent molecular docking studies revealed significant interactions between phytochemicals identified from the crude extract and the target proteins CDK-4 and CDK-6. Isocorydine exhibited the highest binding affinity, followed closely by Thunbergol and Squalene. These targets are crucial regulators of the cell cycle and have been implicated in various cancers, including breast cancer, glioblastoma, and melanoma [40,41,42]. Notably, their binding energies were comparable to the FDA-approved CDK4/6 inhibitors Palbociclib and Ribociclib, reinforcing the potential of *T. siliquosa* phytochemicals as natural CDK inhibitors.

Additionally, compounds with the most negative binding energies and lowest inhibition constants were considered the most effective binders, validating the potential of these phytochemicals as promising therapeutic agents [43,44]. The amino acid residues involved in the docking interactions, detailed in Table 4 and Table 5, provide further evidence of the stability and effectiveness of these interactions. Given the central role of CDK4/6 in promoting uncontrolled cell proliferation in cancer, these results are significant and suggest that the extract could serve as a multi-targeted therapeutic agent. The weaker interactions observed for Methyl Palmitate, Methyl Stearate, 2-palmitoylglycerol, and Hentriacontane may still contribute to the overall pharmacological profile when acting in synergy with the more potent compounds.

Further in silico ADME profiling strengthened the potential of the extract as a drug-like candidate. Most phytochemicals satisfied Lipinski’s Rule of Five, exhibited good gastrointestinal absorption, and showed low blood–brain barrier penetration, reducing the likelihood of central nervous system toxicity. Minimal PAINS alerts also suggest a low risk of non-specific interactions, which is favorable for drug development [45].

While Palbociclib and Ribociclib have demonstrated clinical success as selective CDK4/6 inhibitors, their usage is often associated with notable side effects. Each drug presents a distinct profile of frequently reported adverse effects, including anemia; neutropenia; fatigue; gastrointestinal disturbances like nausea, diarrhea, etc.; and increased risk of infections. In particular, Palbociclib is known for causing stomatitis, fatigue, epistaxis, alopecia, and hot flushes, with neutropenia, leukopenia, infections, and gastrointestinal disturbances. Ribociclib has been associated with anemia, polyneuropathy, thrombocytopenia, QT interval prolongation, and spinal pain, along with hematological abnormalities and elevated liver enzymes [46,47,48,49,50]. These limitations highlight the need for alternative therapeutic agents with reduced toxicity profiles.

Overall, the combined results from cytotoxicity, cell migration assays, cell cycle arrest, molecular docking, and ADME profiling emphasize the potential of *T. siliquosa* root extract as a natural, multi-compound therapeutic candidate. Further in vitro and in vivo studies are warranted to validate these findings, explore the mechanistic pathways involved, and assess the extract’s safety and efficacy in CDK4/6-associated cancers.

## 5. Conclusions

This study highlights the therapeutic potential of *T. siliquosa* root extract as a natural and multi-compound agent capable of targeting cyclin-dependent kinases CDK4 and CDK6, which are key drivers of cell cycle progression in various cancers. Through a combination of GC-MS analysis, biological assays, molecular docking, and ADME profiling, this study has demonstrated that the extract exhibits diverse pharmacological properties, including cytotoxicity, anti-migratory effects, cell cycle arrest in cancer cells, and favorable drug-likeness. Molecular docking revealed strong interactions between the phytochemicals and cancer-associated proteins, with binding affinities comparable to FDA-approved inhibitors, supporting its viability as a natural CDK inhibitor. Additionally, ADME profiling confirmed the extract’s drug-like attributes, such as favorable gastrointestinal absorption and minimal risks of central nervous system toxicity, which are essential for safe therapeutic application. Given the limitations and side effects of existing CDK4/6 inhibitors, *T. siliquosa* root extract offers a promising alternative with potential advantages in toxicity and multi-targeted action. While these findings provide a solid foundation, further in vitro and in vivo studies are crucial to validate the efficacy, elucidate the mechanistic pathways involved, and ensure its safety in clinical settings. These findings pave the way for further research involving protein-level validation, which is essential to delineate the precise molecular targets and mechanisms to confirm the extract’s effectiveness and safety in treating CDK4/6-related cancers.

## Figures and Tables

**Table 1:** Phytochemical compounds identified from *T.siliquosa* using GC-MS analysis.

RT (Min)	Molecular Formula	Molecular Mass (g/mol)	Compound Name	Peak Area%	Match Factor
31.3774	C_19_H_38_O_4_	330.50	2-palmitoylglycerol	19.23%	81.4
32.8266	C_31_H_64_	436.85	Hentriacontane	15.97%	90.1
34.4893	C_20_H_23_NO4	341.4	Isocorydine	8.73%	79.6
33.9098	C_30_H_50_	410.73	Squalene	7.69%	85.5
25.3310	C_17_H_34_O_2_	270.45	Methyl Palmitate	7.63%	94.8
25.5736	C_20_H_34_O	290.5	Thunbergol	5.77%	77.9
27.6686	C_19_H_38_O_2_	298.50	Methyl Stearate	5.04%	93.0

**Table 2:** % of cell viability and cytotoxicity of control and sample-treated cells.

	Concentrations (µg/mL)	% of Viability ± SD
Control cells		100 ± 6.04
Sample-treated cells	100	60.18 ± 9.21
	200	28.24 ± 4.12
	300	26.02 ± 10.43
	400	24.70 ± 4.93
	500	9.64 ± 1.82
	600	6.06 ± 0.58
	700	5.82 ± 0.63
	800	3.99 ± 0.67
	900	4.02 ± 0.43
	1000	3.53 ± 0.50

**Table 3:** % of cell migration in control and sample-treated cells.

	% Migration	
	Control Cells	Treated Cells
**0 h**	0	0
**24 h**	34.93	27.01
**36 h**	57.26	30.78

**Table 4:** % distribution of HCT116 cells in different phases of cell cycle.

Group	G1 Phase (%)	S Phase (%)	G2 Phase (%)
**Control**	52.25 ± 0.66	19.34 ± 1.22	27.44 ± 0.24
**Treated**	51.95 ± 0.36	14.67 ± 0.60	32.70 ± 0.45

**Table 5:** Binding energy of selected ligands against CDK4/6.

Compound	PubChem ID	Binding Energy (kcal/mol)	
		CDK4 (2W9F)	CDK6 (1JOW)
Isocorydine	10143	**−7.4**	**−7.2**
Thunbergol	5363523	−6.5	**−7**
Squalene	638072	**−7.1**	−4.9
2-Palmitoylglycerol	123409	−5.2	−4.9
Methyl palmitate	8181	−4.5	−5.5
Methyl stearate	8201	−4.4	−4.7
Hentriacontane	12410	−4.8	−3.9
Palbociclib	5330286	−7.2	−8.3
Ribociclib	44631912	−7.1	−8.1

**Table 6:** Interacting amino acid residues of the CDK4 (2W9F) with the phytochemicals and standards.

10143	5363523	638072	123409	8181	8201	12410	5330286 (Standard)	4431912 (Standard)
-	-	-	-	-	-	-	Lys58	-
-	-	-	-	Gln183	Gln183 (Pi-Sigma)	-	-	-
Glu69 (Pi-Alkyl)	-	-	-	-	-	-	-	Glu69 (Pi-Sigma)
-	-	Phe78	-	Phe78 (Pi-Alkyl)	-	-	-	-
-	-	-	-	-	-	-	Thr62 (Pi-Alkyl)	-
Ala187	Ala187	Ala187 (Pi-Alkyl)	Ala187	Ala187	Ala187	Ala187	-	-
-	-	-	Ser258	-	-	-	-	-
-	-	-	-	-	Cys73 (Pi-Alkyl)	-	-	-
-	-	-	-	-	-	-	-	Glu66 (Alkyl)
-	-	Ile177	-	-	-	-	-	-
-	-	Pro79	-	Pro79 (Pi-Sigma)	-	-	-	-
-	Leu188	-	-	-	-	Leu188 (Pi-Sigma)	-	Leu188
Glu74 (Pi-Alkyl)	-	-	-	-	-	-	-	-
-	-	Cys68 (Pi-Sigma)	Cys68 (Pi-Alkyl)	Cys68	-	Cys68	-	-
-	Gln261 (Pi-Alkyl)	-	Gln261	-	-	-	Gln261	Gln261
Leu65	Leu65	-	Leu65	-	-	Leu65	-	-
-	-	Lys180	-	Lys180 (Pi-Alkyl)	Lys180	Lys180	-	Lys180 (Pi-Sigma)
Lys72	-	-	-	-	-	-	-	
-	-	His158 (Pi-Sigma)	-	-	-	-	-	-

**Table 7:** Interacting amino acid residues of the CDK6 (1JOW) with the phytochemicals and standards.

10143	5363523	638072	123409	8181	8201	12410	5330286 (Standard)	4431912 (Standard)
-	-	-	-	Leu185 (Pi-Alkyl)	-	-	-	-
Tyr174	Tyr174	-	Tyr174	-	Tyr174	-	Tyr174 (Pi-Sigma)	Tyr174
-	-	-	-	Leu32	-	-	-	-
-	-	Trp58	-	-	-	Trp58 (Pi-Sigma)	-	-
-	Ile13	-	-	-	-	-	-	-
-	-	-	-	-	-	-	-	Arg30
Asp14	-	-	-	-	-	-	-	-
-	-	-	Pro158 (Pi-Alkyl)	-	Pro158 (Pi-Sigma)	-	-	-
-	-	-	Asp154	-	Asp154	-	Asp154	-
-	-	Ile54	-	-	-	Ile54	-	-
-	-	-	-	Leu34 (Pi-Sigma)	-	-	-	-
-	Lys12	-	-	-	-	-	Lys12	-
-	-	Leu254	-	-	-	Leu254	-	-
-	-	-	-	-	-	-	Lys70 (Pi-Alkyl)	-
-	Lys19	-	Lys19	-	-	-	-	-
-	-	Leu55 (Pi-Alkyl)	-	-	-	Leu55	-	-
-	Ile157	-	Ile157	-	Ile157	-	-	-
-	-	-	-	-	-	-	Arg109	-
-	-	-	-	Phe37	-	-	-	-
-	-	Lys91	-	-	-	Lys91	-	-
-	-	-	-	Pro195	-	-	-	-
Pro171	-	-	Pro171	-	Pro171	-	-	-
-	-	-	-	-	-	-	Ser71 (Pi-Alkyl)	-
-	-	-	-	Ile198	-	-	-	-
-	-	Leu94 (Pi-Alkyl)	-	-	-	Leu94	-	-

**Table 8:** ADME properties of ligands.

Ligands	TPSA (Å^2^)	Water Solubility	GI Absorption	BBB Permeation	Drug Likeliness	Lipinski’s Rule of Five	PAINS Alert
Isocorydine	51.16	Soluble	High	Yes	Yes	0 violation	0
Thunbergol	20.23	Moderate	High	No	Yes	1 violation (LogP = 5.64)	0
Squalene	0.00	Poor	Low	No	Yes	1 violation (LogP = 10.74)	0
2-palmitoylglycerol	66.76	Moderate	High	Yes	Yes	0 violation	0
Methyl palmitate	26.30	Moderate	High	Yes	Yes	1 violation (LogP = 6.34)	0
Methyl stearate	26.30	Moderate	High	No	Yes	1 violation (LogP = 7.15)	0
Hentriacontane	0.00	Insoluble	Low	No	Yes	1 violation (LogP = 12.29)	0
Palbociclib	105.04	Soluble	High	No	Yes	0 violation	0
Ribociclib	91.21	Soluble	High	No	Yes	0 violation	0

**Table 9:** Pharmacological activities of bioactive compounds from *T.siliquosa*.

Compound Name	Pharmacological Activities	References
**2-palmitoylglycerol**	Anti-inflammatory, Prevents apoptosis	[20,21]
**Hentriacontane**	Anticancer, Antioxidant, Anti-inflammatory, Anti-tubercular, Immunomodulator, Hepatoprotective, Antimicrobial	[22]
**Isocorydine**	Anticancer, Antioxidant, Anti-inflammatory, Antisepsis, Anti-arrhythmia, Vasodilation, Antitumor,	[23,24,25]
**Squalene**	Anticancer, Antioxidant, Anti-inflammatory, Cardioprotective, Hepatoprotective, Promotes skin health	[26,27,28,29]
**Methyl Palmitate**	Anticancer, Antioxidant, Anti-inflammatory, Antimicrobial, Hypocholesterolemic, Hemolytic	[30,31]
**Thunbergol**	Antioxidant, Antimicrobial, Neuro-protective	[32,33]
**Methyl Stearate**	Anticancer, Anti-inflammatory, Antimicrobial	[30]

## Data Availability

The original contributions presented in this study are included in the article/Supplementary Materials. Further inquiries can be directed to the corresponding authors.

## Supplementary Materials

The following supporting information can be downloaded at: https://www.mdpi.com/article/10.3390/biomedicines13071658/s1.

## Abbreviations

The following abbreviations are used in this manuscript:

CDK	Cyclin-Dependent Kinase
ADME	Absorption, Distribution, Metabolism, and Excretion
GC-MS	Gas Chromatography-Mass Spectrometry
HCT116	Human Colonic Tumor—116 Cancer Cell Line
MTT	3-(4,5-dimethylthiazol-2yl)-2,5-diphenyltetrazolium bromide
IC_50_	Half-Maximal Inhibitory Concentration
DMEM	Dulbecco’s Modified Eagle’s Medium
DMSO	Dimethyl Sulfoxide
PBS	Phosphate-Buffered Saline
PI	Propidium Iodide
RCSB	Research Collaboratory for Structural Bioinformatics
AD4	AutoDock4
FDA	Food and Drug Administration
G1 phase	Gap1
S phase	Synthesis Phase
G2 phase	Gap2
M phase	Mitosis
