# Supplementary material for: Targeting CDK4/6 in Cancer: Molecular Docking and Cytotoxic Evaluation of Thottea siliquosa Root Extract
Source: Biomedicines. 2025 Jul 7;13(7):1658. doi: 10.3390/biomedicines13071658 (PMC12292890; doi:10.3390/biomedicines13071658)

CELL MIGRATION ASSAY

(Hulkower and Herber 2011)

Cell migration assay is also known as scratch assay is a basic technique was used to check the speed and determination of the sample treated cell.

Principle

Cell migration assay is mainly used to study the synchronized movement of a cell population. A cell–free is shaped in a confluent monolayer by physical exclusion. The exposure to the cell-free area induces the cells to migrate in to the gap.

Reagents

1. DMEM (Dulbecco's Modified Eagle Medium)

2. 10% FBS (Fetal Bovine Serum)

3. PBS (Phosphate Buffered Saline)

Procedure

Each well of a 6-well tissue culture plate was seeded with cells to a final density of 100,000 cells/well and these cells were maintained at 37˚C and 5% CO2 for 24 hrs to permit cell adhesion and the formation of a confluent monolayer. These confluent monolayers were then scored with a sterile pipette tip to leave a scratch of ~0.4-0.5 mm in width. The cell surface was then washed with serum-free culture medium for three times to remove dislodged cells. Wound closure was monitored by collecting digitized images at 0 and 24 hrs after the scratch was done. Digitized images were captured with an inverted phase contrast microscope

Control

0 hour

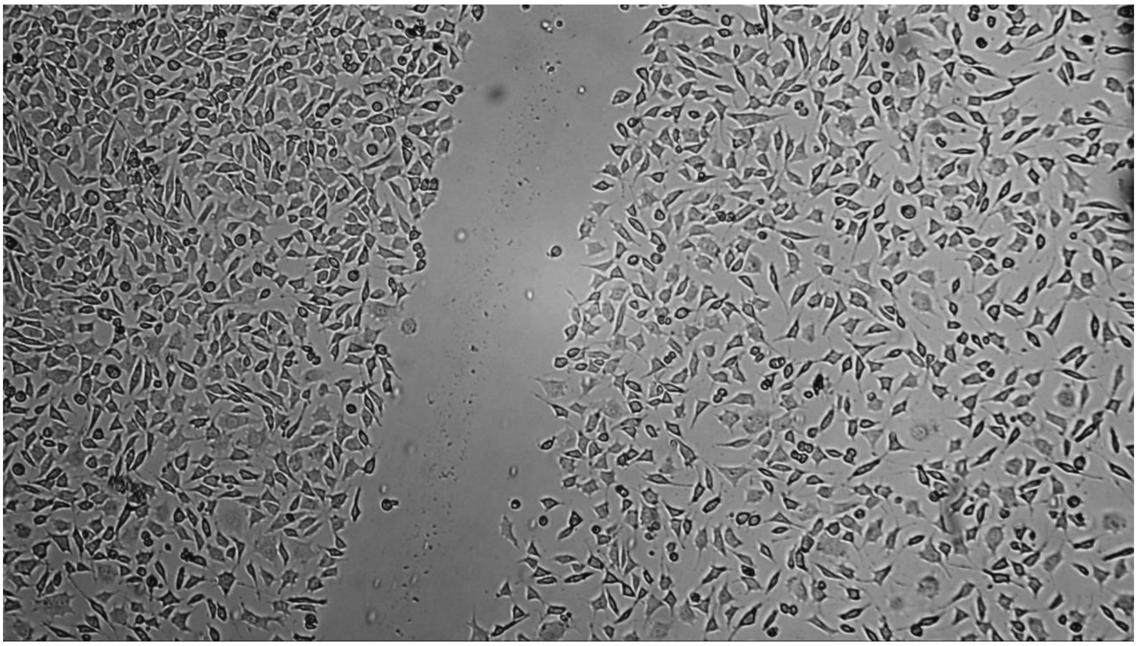

24hr

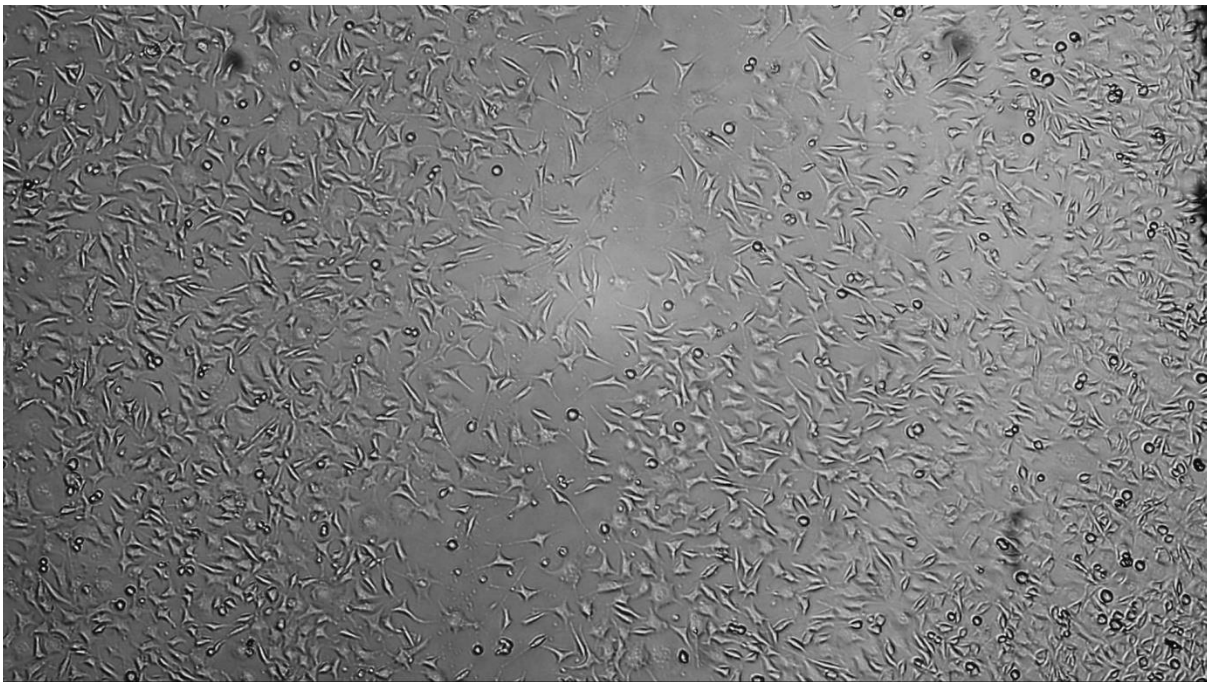

Sample treated

0hr

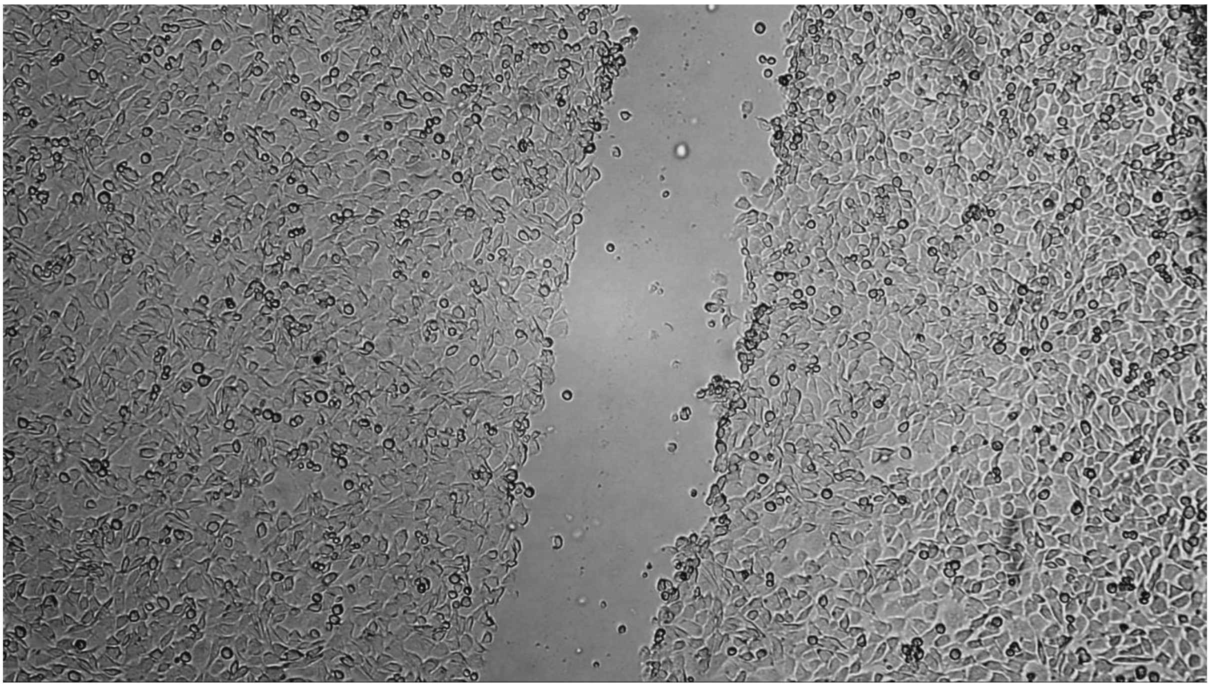

24hr

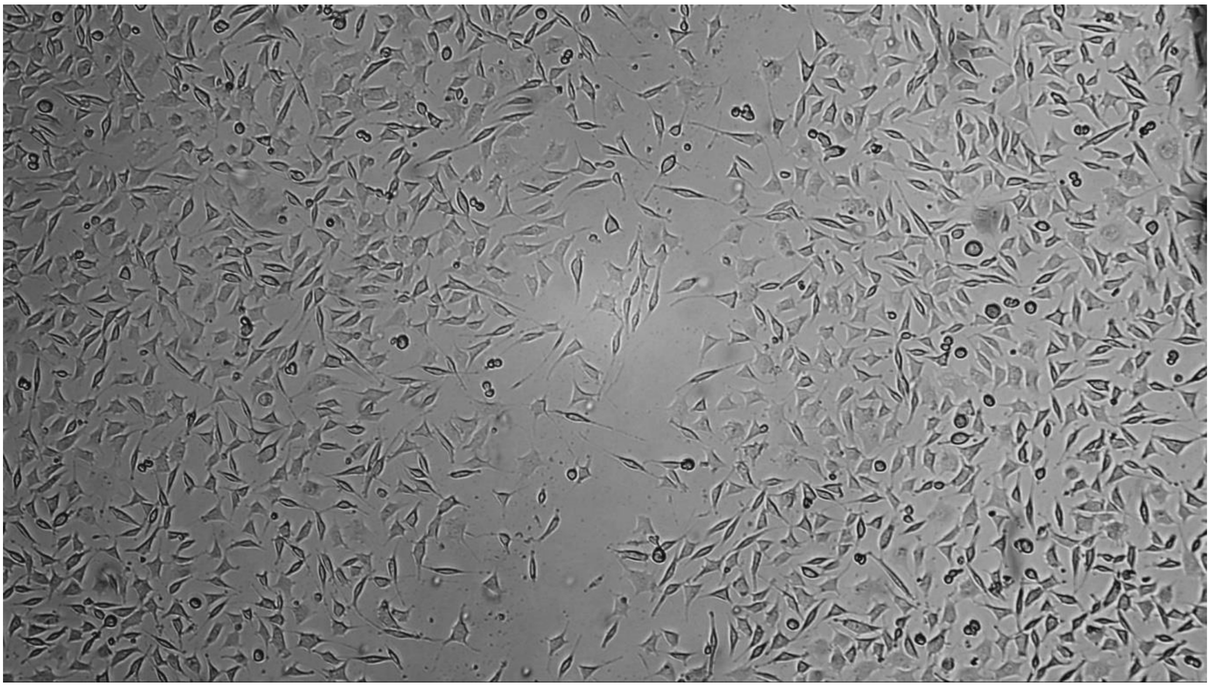

Supplement: Supplementary file 1 [file biomedicines-13-01658-s001.zip › CELL MIGRATION ASSAY.pdf]
