# Supplementary material for: Targeting CDK4/6 in Cancer: Molecular Docking and Cytotoxic Evaluation of Thottea siliquosa Root Extract
Source: Biomedicines. 2025 Jul 7;13(7):1658. doi: 10.3390/biomedicines13071658 (PMC12292890; doi:10.3390/biomedicines13071658)
Supplement: Supplementary file 1 [file biomedicines-13-01658-s001.zip › cultutre maintenence- L-929 cells.pdf]

Culturing and maintenance of cell lines

The cell lines namely L-929 cells (Fibroblast-connective mouse tissue) were purchased from National Centre for Cell Science (NCCS), Pune, India. The cells were maintained in a CO_2 incubator with 5% CO_2 and 95% humidity atmosphere supplemented with DMEM medium, 10% FBS, penicillin and streptomycin at 1X final concentration from a 100X stock. Once the cells attained confluent growth, the cells were trypsinized using Trypsin-EDTA and the cells (10^5) was seeded into sterile 96-well and 6 well plates for carrying out for assays. The cytotoxicity assays were carried out in 96-well plates. In each well of the 96-well plates cells were seeded, the cells were seeded followed by the incubation in a CO_2 incubator (Innova CO-170, United States) with 5 % CO_2 and 95% humidity atmosphere.
