# Supplementary material for: Targeting CDK4/6 in Cancer: Molecular Docking and Cytotoxic Evaluation of Thottea siliquosa Root Extract
Source: Biomedicines. 2025 Jul 7;13(7):1658. doi: 10.3390/biomedicines13071658 (PMC12292890; doi:10.3390/biomedicines13071658)

Cell viability assay/Cytotoxicity Test

The extent of cytotoxicity of the synthesized sample to the L-929 cells was determined by the MTT dye reduction assay as described by Igarashi and Miyazawa (2001).

Principle

MTT is a water-soluble tetrazolium salt that is reduced by metabolically viable cells to a colored water insoluble formazan salt. Live cells convert MTT into its formazan derivative; the number of surviving cells can be determined by the amount of MTT formazan produced, which is measured in a micro titre plate reader

Reagents

1. PBS (phosphate buffered saline)-pH- 7.4

2. MTT - 3mg/ml in PBS

3. Isopropanol in 0.04N HCl (acid-propanol)

Procedure

The treated cells were centrifuged and the medium was removed and then incubated with 50µl of MTT at 37°C for 3 hours. After incubation, 200µl of PBS was added to all samples and the liquid was then carefully aspirated. Acid propanol of 200µl was added and left overnight in the dark. The absorbance was read at 650nm in a micro titre plate reader (Bio RAD U.S.A). The optical density of the control cells were fixed to be 100% viable and the per cent viability of the cells in the treatment groups were calculated accordingly.

$$\text{Percent viability} = \frac{\text{Control OD} - \text{Sample OD}}{\text{Control OD}} \text{ X } 100$$

Result

	**Concentrations**	**OD value**	**% of viability**	**% of cytotoxicity**
Control cells		0.589	100%	-
Sample: EL-M	5 µl	0.055	90.66	9.34
	10 µl	0.067	88.62	11.38
	20 µl	0.099	83.19	16.81
	50 µl	0.248	57.89	42.11
	100 µl	0.321	45.50	54.50

Images

Control Cells

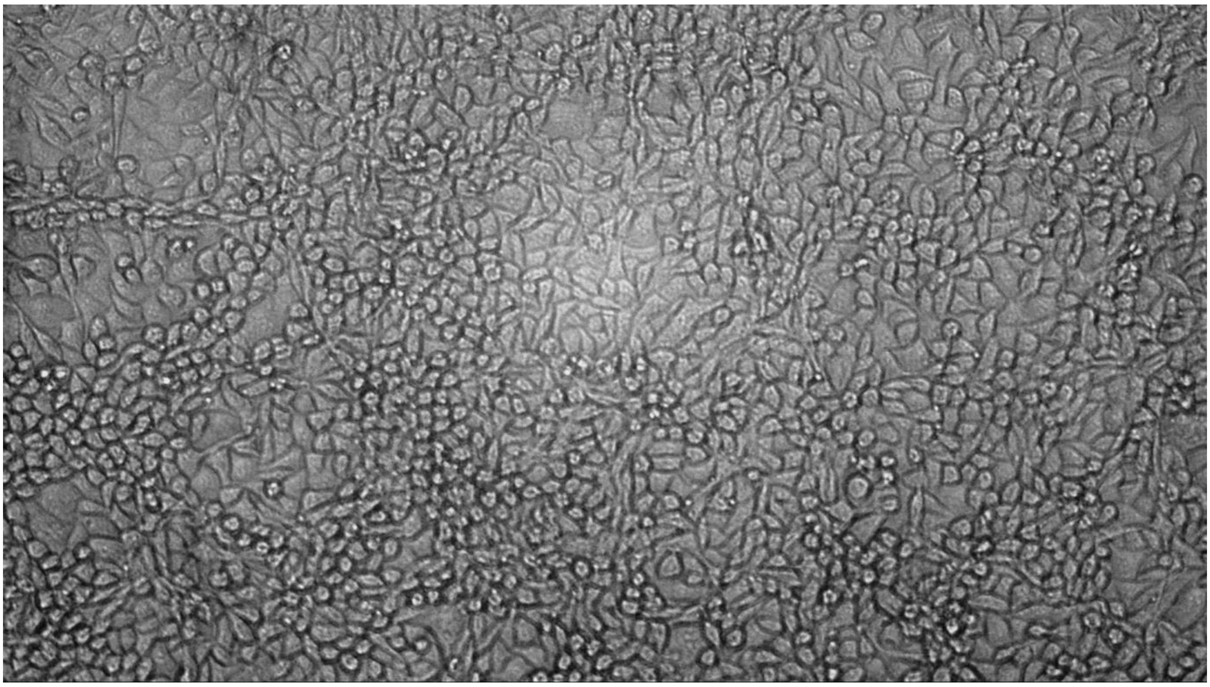

Sample treated cells

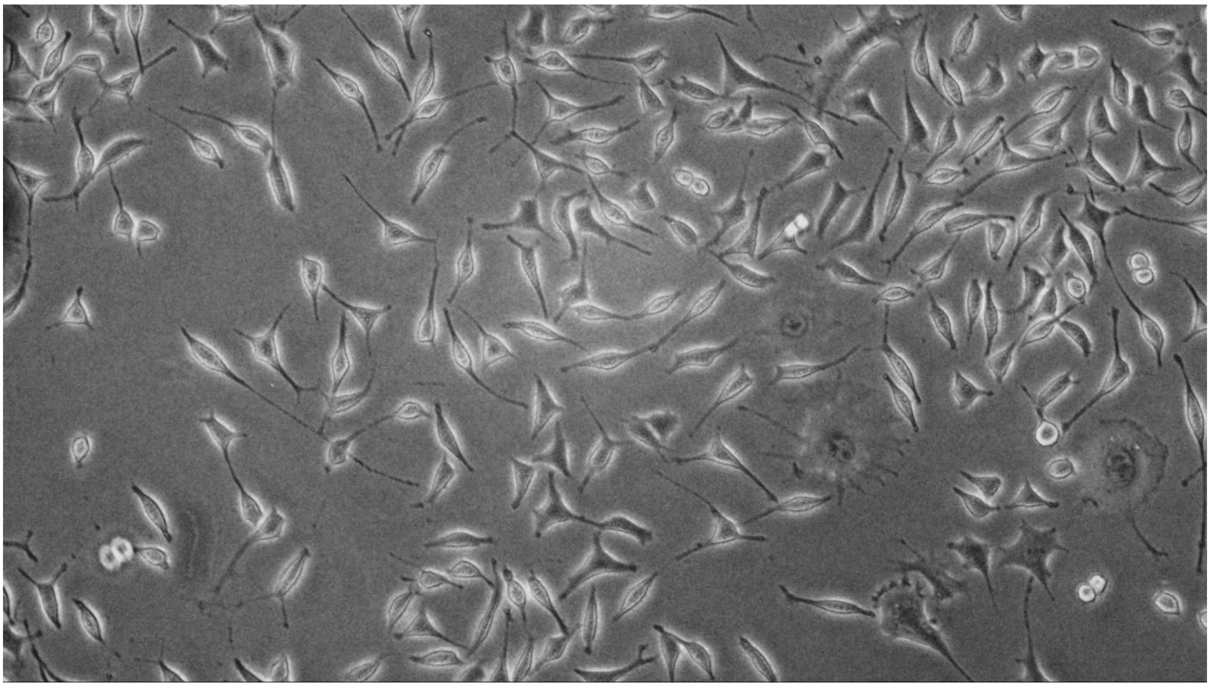

Supplement: Supplementary file 1 [file biomedicines-13-01658-s001.zip › Cytotoxicity assay Result.pdf]
