# Supplementary material for: Targeting CDK4/6 in Cancer: Molecular Docking and Cytotoxic Evaluation of Thottea siliquosa Root Extract
Source: Biomedicines. 2025 Jul 7;13(7):1658. doi: 10.3390/biomedicines13071658 (PMC12292890; doi:10.3390/biomedicines13071658)

Library Search Results - NonTarget Hits with Details

Agilent Trusted Answers

**Batch Path**	D:\data\UNKNOWN SAMPLES\2024\APR-24\02-04-2024		
**Analysis File Name**	02-04-2024 unknown analysis methanol.uaf		
**Analyst Name**	GCMS	**Analysis Time**	04-04-2024 09:30:08 AM
**File Name**	E2400002-2 AQ-R-MR .D	**Path Name**	D:\data\UNKNOWN SAMPLES\2024\APR-24\02-04-2024
**Sample Name**	E2400002-2 AQ-R-MR	**Sample Type**	Sample
**Acq. Method File**	UNKNOWN SAMPLE	**Acq. Method Path**	D:\MassHunter\GCMS\1\methods\
**Acq. Date-Time**	02-04-2024 03:00:26 PM	**Acq. Operator**	
**Instrument Name**	CH-GCMSMS-02	**Dil.**	1

Component RT	Compound Name	CAS#	Formula	Component Area	Match Factor	Estimated Conc.
3.0737	Methane, (methylsulfinyl)(methylthio)-	33577-16-1	C3H8OS2	3176049.1	69.8	
3.1215	Silane, dimethoxydimethyl-	1112-39-6	C4H12O2Si	492000.3	69.3	
3.1642	Acetic acid	64-19-7	C2H4O2	9605185.0	97.2	
3.3126	Acetamide, N,N'-ethylenebis(N-nitro-	922-89-4	C6H10N4O6	1920117.9	68.9	
3.3574	Acetic acid, methyl ester	79-20-9	C3H6O2	5200164.9	81.7	
3.4162	Phenol, 4-ethyl-2-methyl-	2219-73-0	C9H12O	140396.2	67.0	
3.4395	Hydrogen isocyanate	75-13-8	CHNO	2424964.3	78.7	
3.4572	Silanediol, dimethyl-	1066-42-8	C2H8O2Si	5397608.0	98.2	
3.5114	Propane-1,1-diol diacetate	33931-80-5	C7H12O4	1667313.3	63.4	
3.5207	Diacetyl sulphide	3232-39-1	C4H6O2S	3322957.9	65.7	
3.6686	n-Propyl acetate	109-60-4	C5H10O2	21325914.1	76.4	
3.7292	L-Lactic acid	79-33-4	C3H6O3	4266813.2	82.9	
3.8623	1-Phenyl-1-decanol	21078-95-5	C16H26O	431747.8	81.3	
4.1045	Propanoic acid, 2-oxo-, methyl ester	600-22-6	C4H6O3	4825824.0	87.6	
4.5307	Pyrazine, methyl-	109-08-0	C5H6N2	423145.0	77.0	
5.7694	1-Pentamethyldisilanyl-4-trimethylsiloxybenzene	1000427-15-5	C14H28OSi3	200910.6	66.1	
6.0578	n-Amyl ether	693-65-2	C10H22O	1902494.2	61.9	
6.5923	S-Ethyl ethanethioate	625-60-5	C4H8OS	244164.1	69.6	
7.3506	.beta.-Hydroxyethyltheophylline tert-butyldimethylsilyl ether	1000442-93-8	C15H26N4O3Si	177937.3	63.3	
13.5277	2-Oxo-4-phenyl-6-(4-chlorophenyl)-1,2-dihydropyrimidine	24030-13-5	C16H11ClN2O	152759.8	74.5	
14.0354	2-Tetrazene, 1,1,4,4-tetramethyl-	6130-87-6	C4H12N4	7393.4	61.4	
16.1721	Propanenitrile, 3-(2-methoxy-1-methylethoxy)-	35633-52-4	C7H13NO2	23889.3	63.3	
21.1806	6-Methoxythymyl isobutyrate	1000413-80-3	C15H22O3	1899277.0	68.1	
21.1815	Phenol, 4-ethenyl-2,6-dimethoxy-	28343-22-8	C10H12O3	2648244.3	64.4	
22.7730	1,3-Dioxolane, 2-methyl-2-(4-methyl-3-methylenepentyl)-	66972-05-2	C11H20O2	2945986.9	69.3	
24.0346	Undec-10-ynoic acid, tridec-2-yn-1-yl ester	1000406-96-9	C24H40O2	1354911.9	69.2	
25.2119	Isoaromadendrene epoxide	1000159-36-6	C15H24O	3670403.5	81.4	
25.3310	Hexadecanoic acid, methyl ester	112-39-0	C17H34O2	6957469.9	94.8	
25.5736	Thunbergol	25269-17-4	C20H34O	5266348.1	77.9	
27.6686	Methyl stearate	112-61-8	C19H38O2	4599766.1	93.0	
31.2201	Decane, 3,8-dimethyl-	17312-55-9	C12H26	1734251.9	73.3	
31.3774	Hexadecanoic acid, 2-hydroxy-1-(hydroxymethyl)ethyl ester	23470-00-0	C19H38O4	17537393.6	81.4	
31.6943	Phthalic acid, octyl 2-pentyl ester	1000315-48-0	C21H32O4	553053.6	60.9	
32.8225	Myristic acid, 4-methoxyphenyl ester	1000357-93-0	C21H34O3	660588.4	67.0	
32.8266	Hentriacontane	630-04-6	C31H64	14565868.0	90.1	
32.8867	1,3-Benzenediol, O-(4-butylbenzoyl)-O'-(4-fluorobenzoyl)-	1000345-61-1	C24H21FO4	1524776.2	60.6	
33.0504	Butylamine, N-acetyl-1-cyano-2-ethyl-	1000227-04-1	C9H16N2O	2135151.5	71.4	
33.6070	Octacosane	630-02-4	C28H58	6716655.7	84.0	
33.9098	Squalene	111-02-4	C30H50	7014632.2	85.5	
34.4893	(+)-(S)-Isocorydine	475-67-2	C20H23NO4	7959157.1	79.6	

Sample Chromatogram

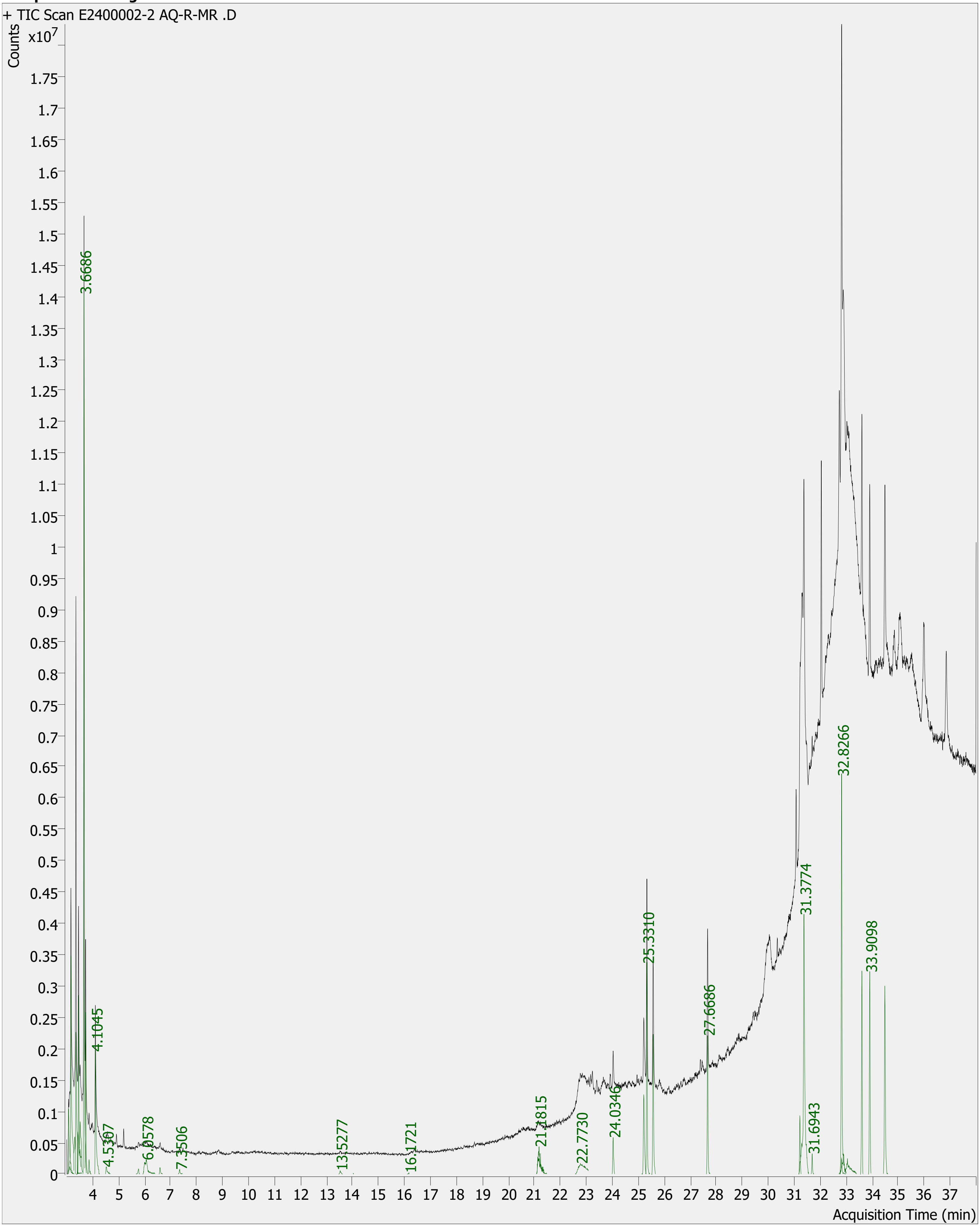

Library Search Results - NonTarget Hits with Details

Component RT	Compound Name	Component Area	Match Factor	CAS#	Formula	Estimated Conc.
3.0737	Methane, (methylsulfinyl)(methylthio)-	3176049.1	69.8	33577-16-1	C3H8OS2	

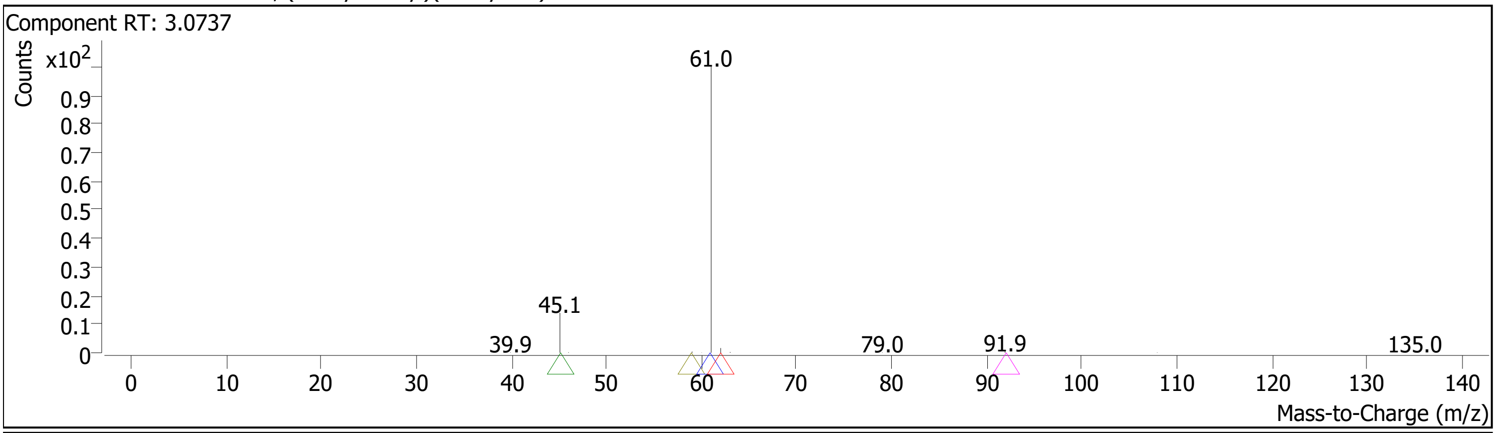

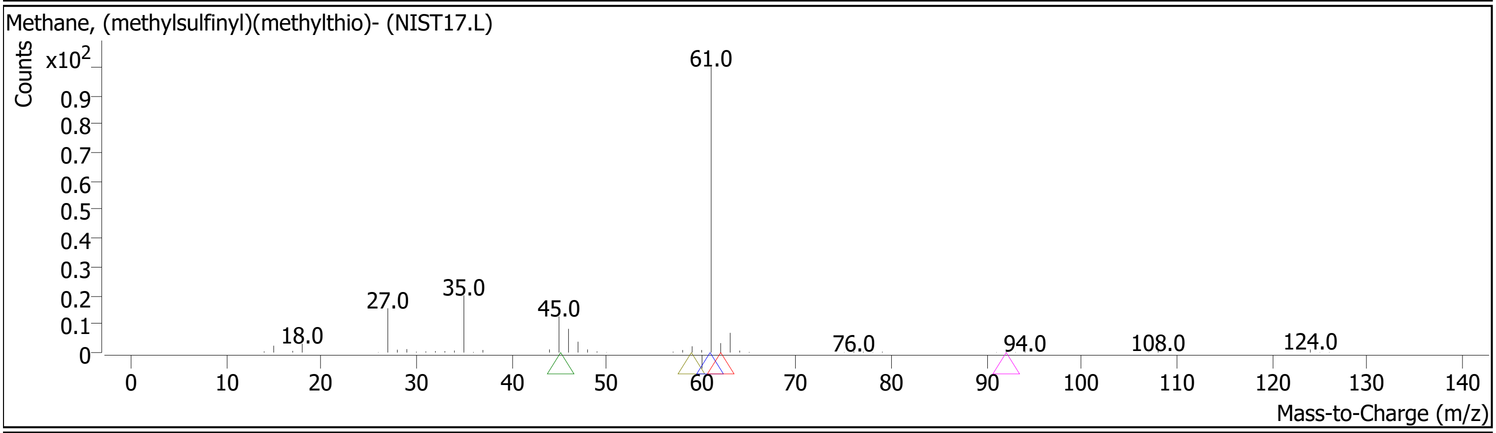

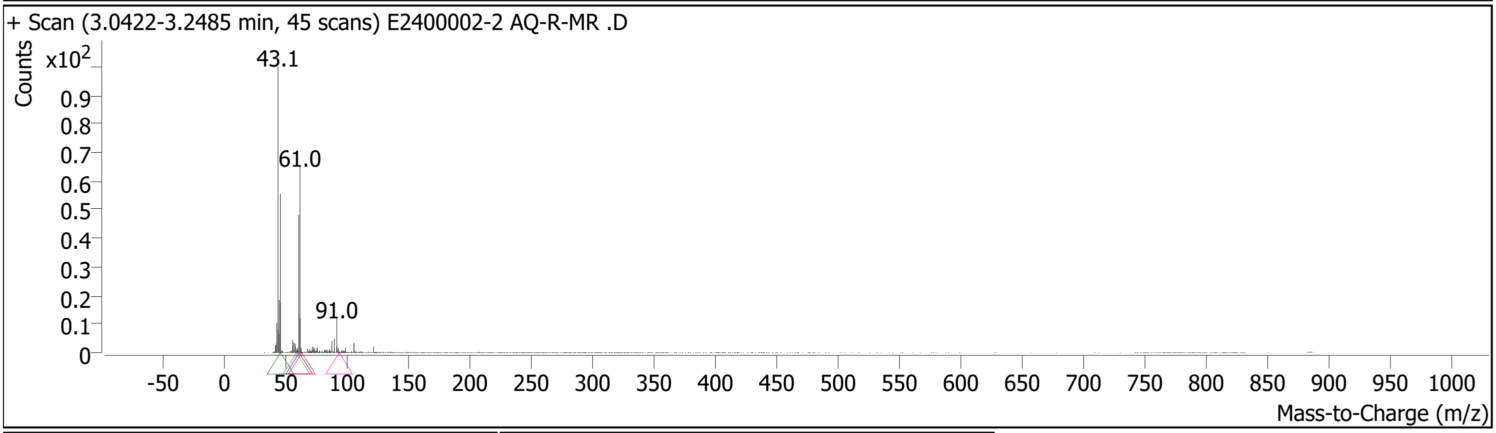

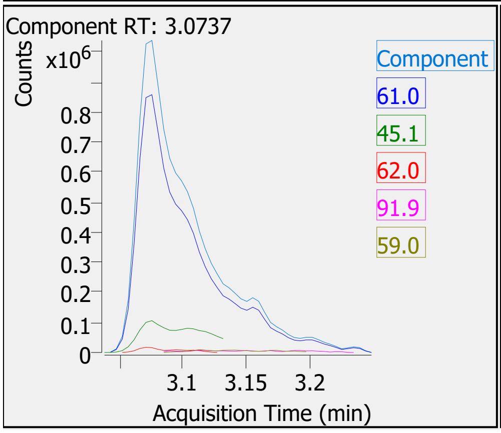

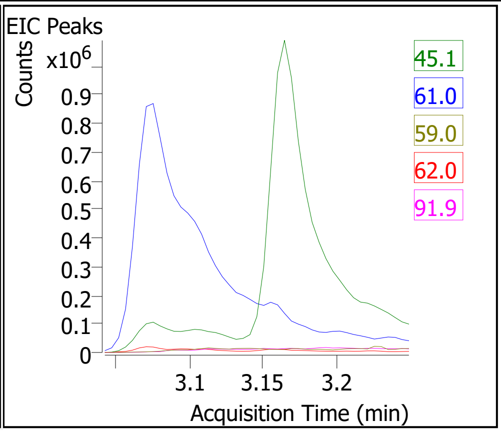

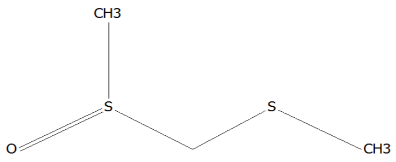

 Generated at 09:38 AM on 04-04-2024

Component RT	Compound Name	Component Area	Match Factor	CAS#	Formula	Estimated Conc.
3.1215	Silane, dimethoxydimethyl-	492000.3	69.3	1112-39-6	C4H12O2Si	

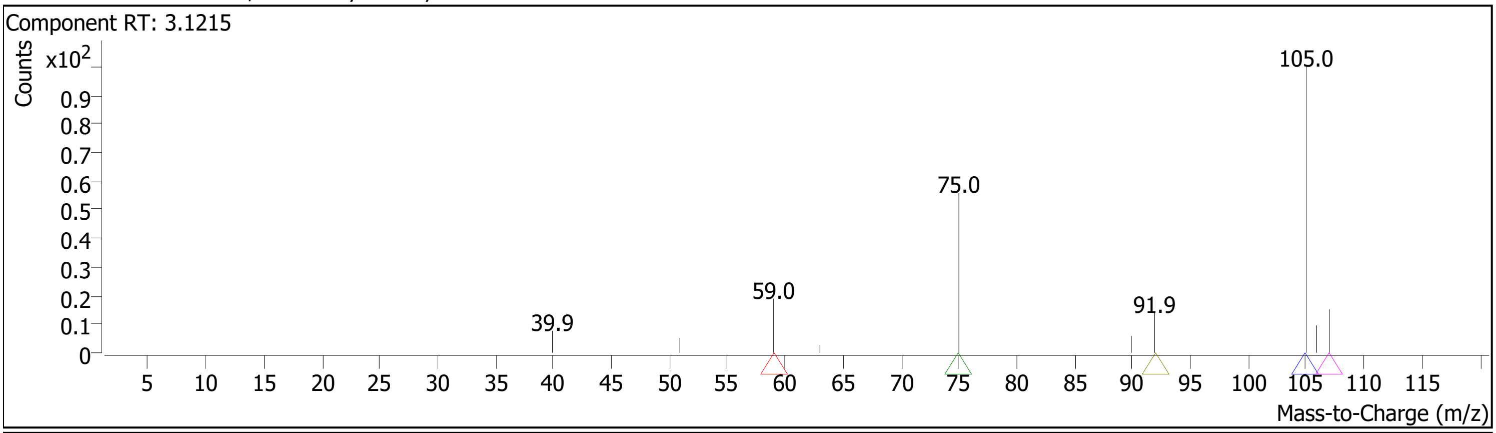

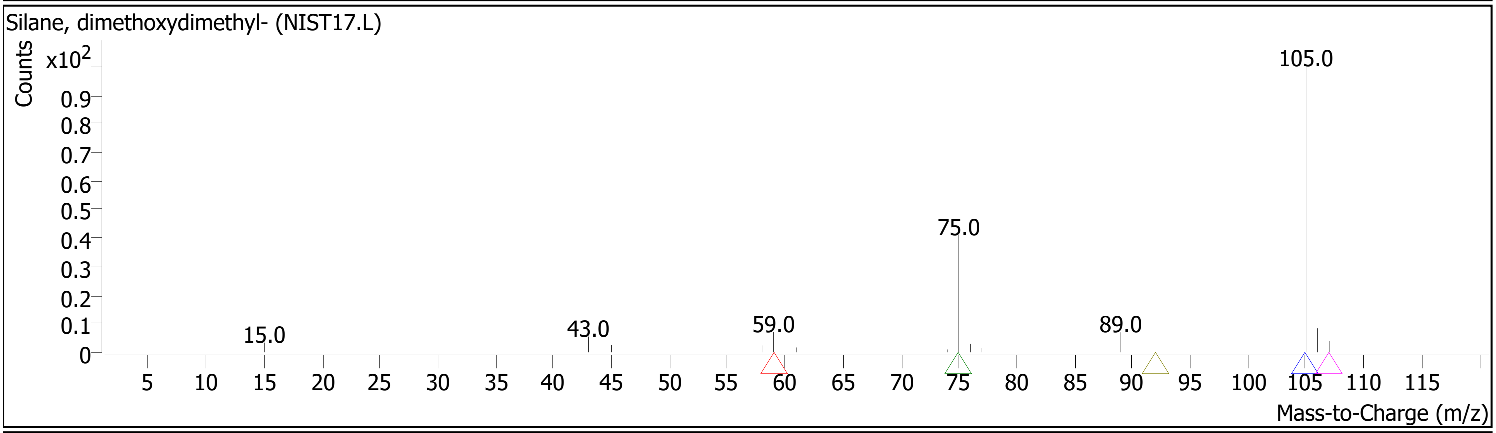

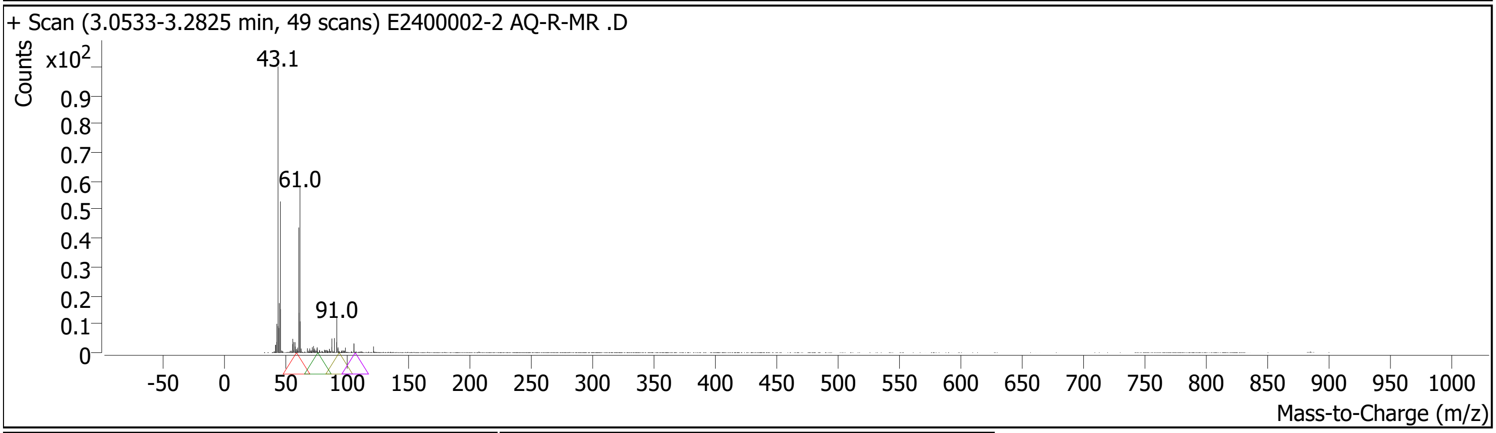

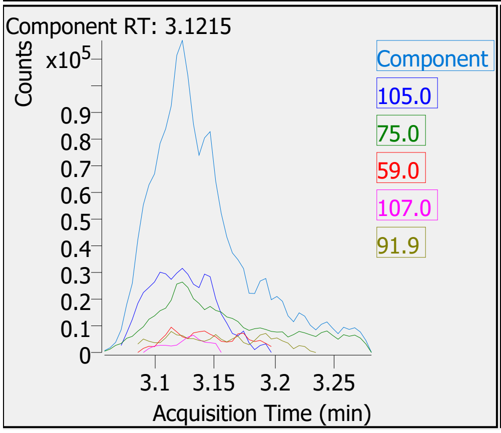

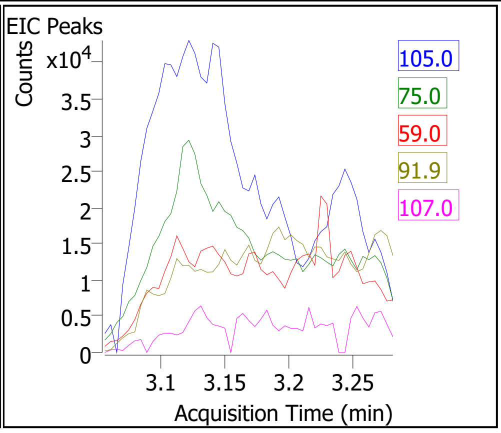

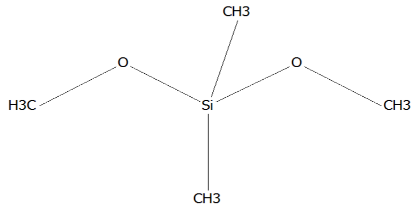

Component RT	Compound Name	Component Area	Match Factor	CAS#	Formula	Estimated Conc.
3.1642	Acetic acid	9605185.0	97.2	64-19-7	C2H4O2	

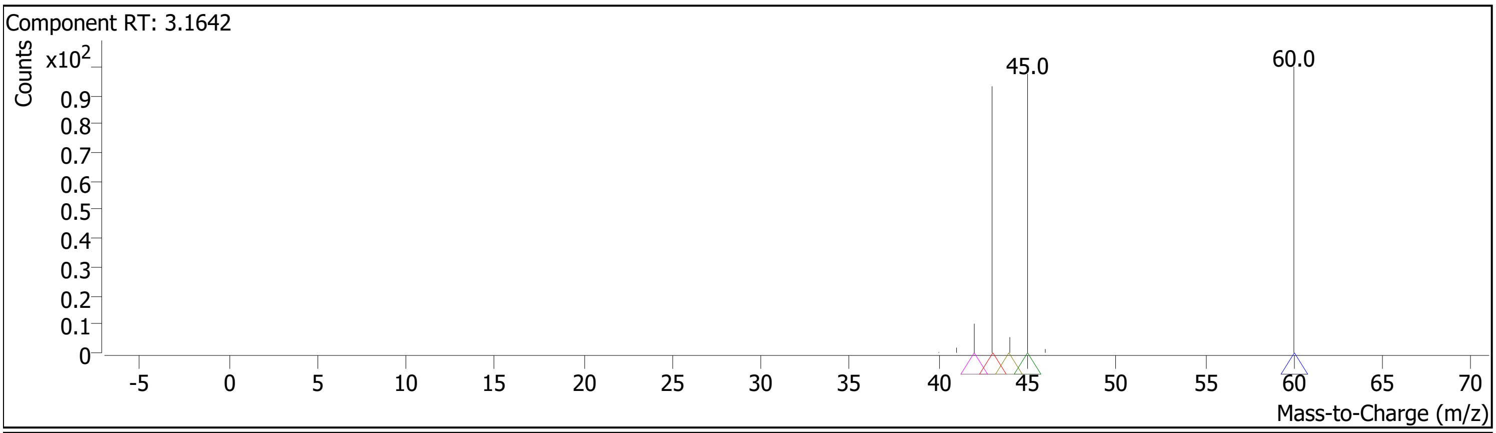

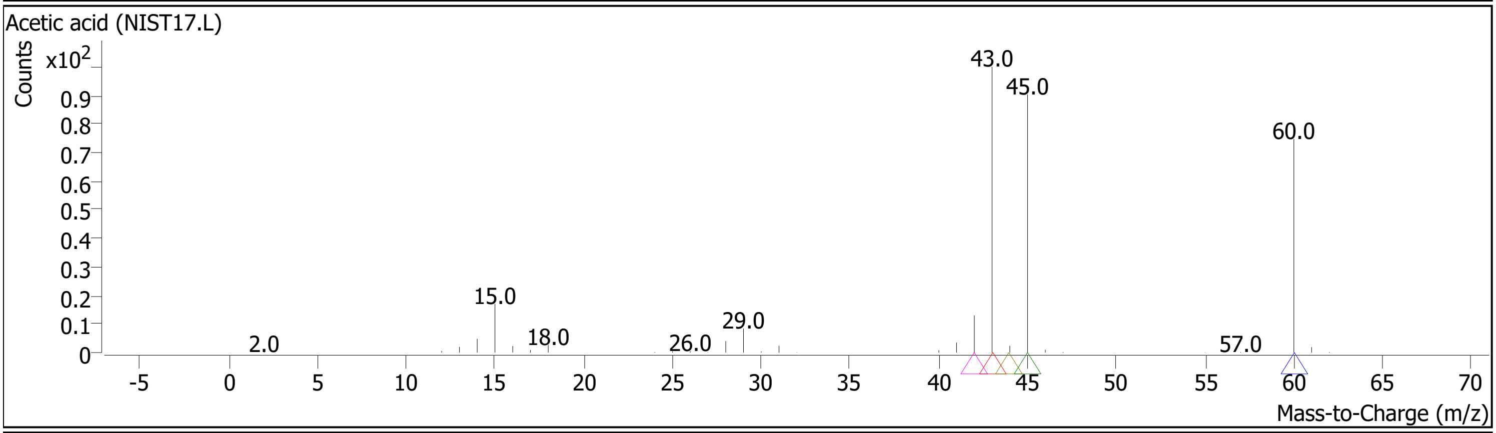

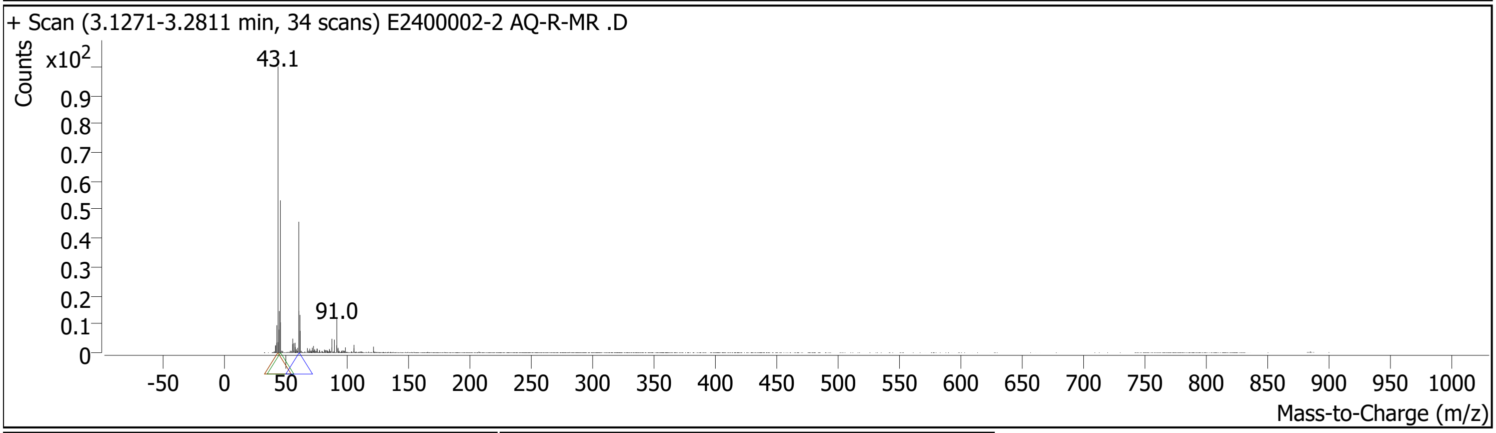

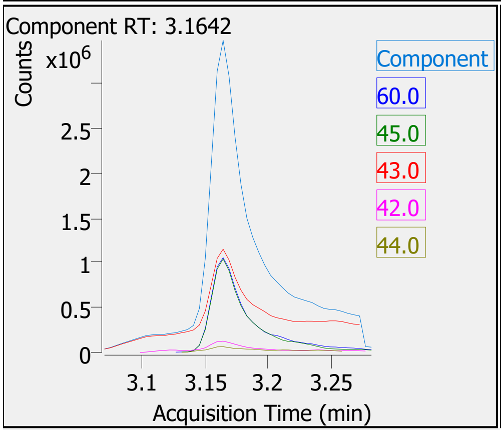

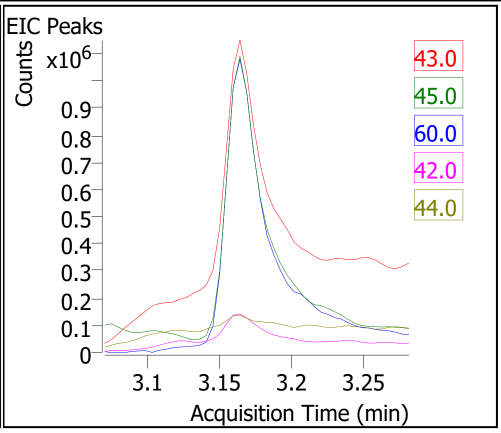

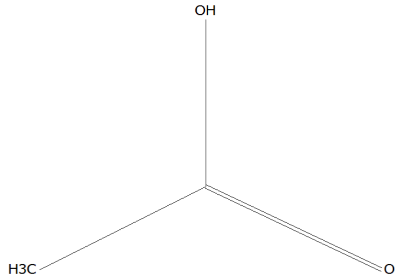

Component RT	Compound Name	Component Area	Match Factor	CAS#	Formula	Estimated Conc.
3.3126	Acetamide, N,N'-ethylenebis(N-nitro-	1920117.9	68.9	922-89-4	C6H10N4O6	

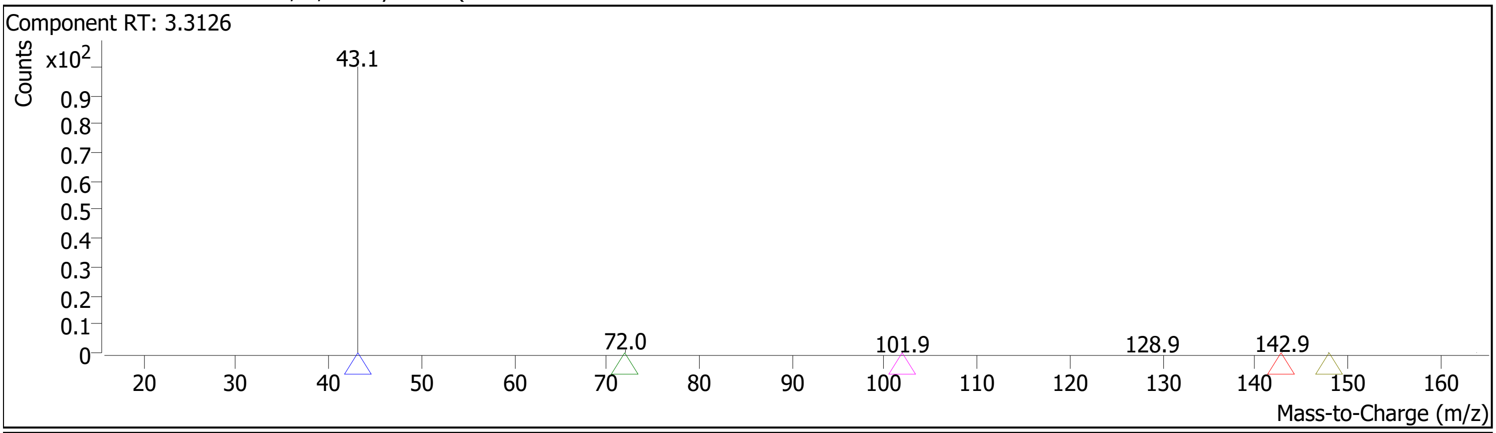

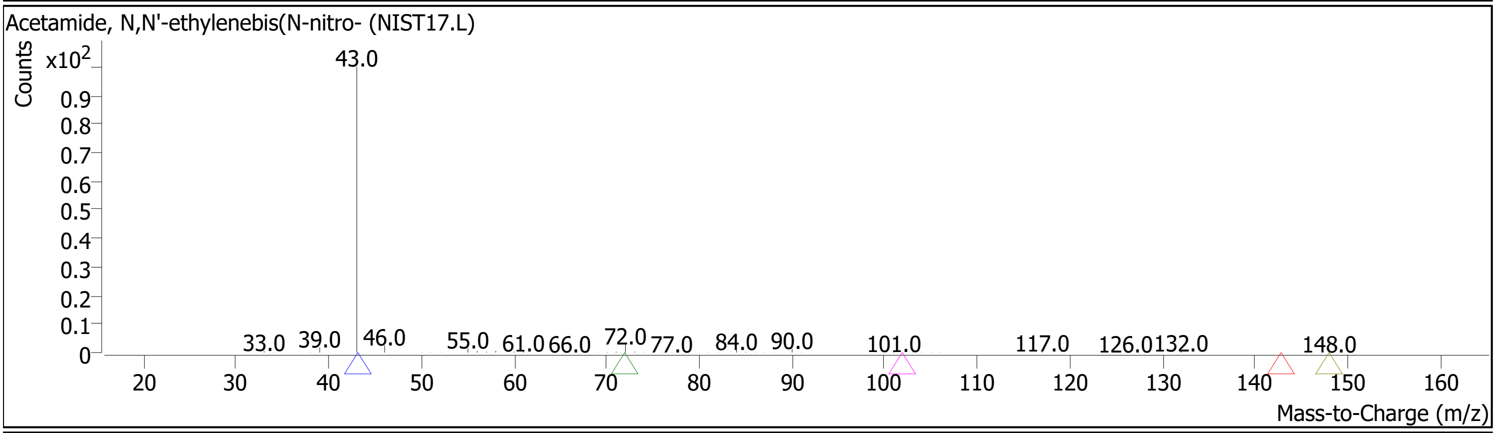

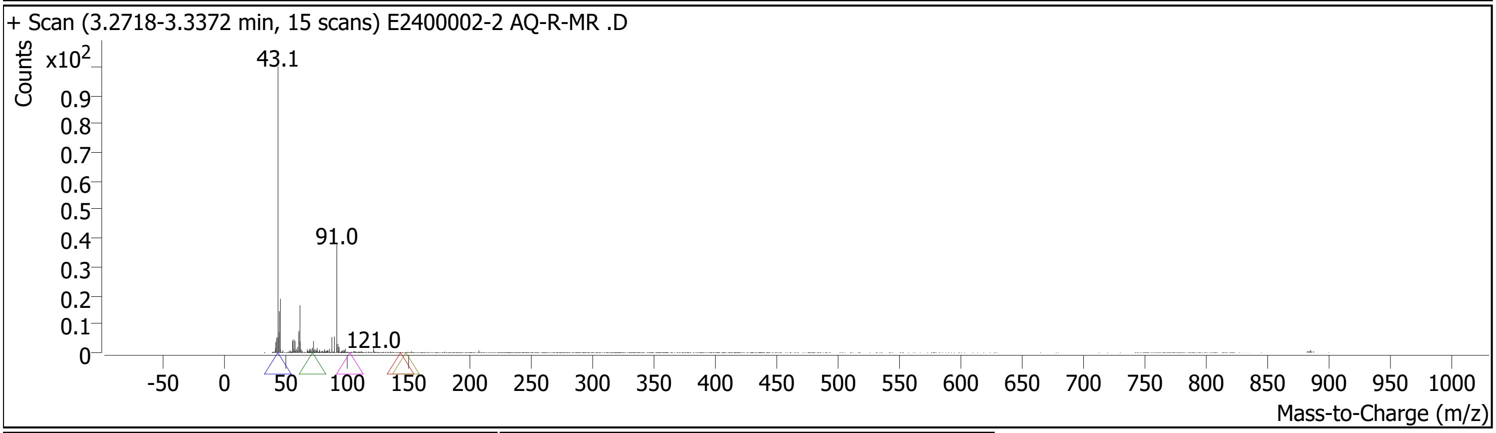

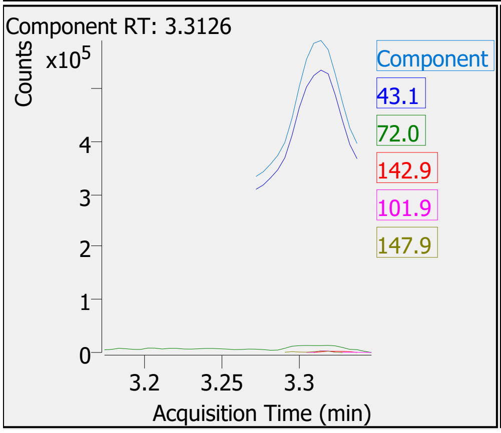

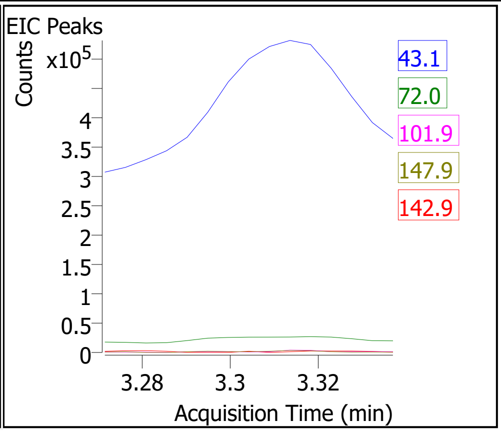

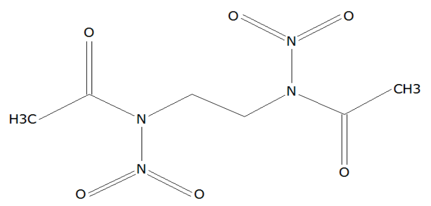

Component RT	Compound Name	Component Area	Match Factor	CAS#	Formula	Estimated Conc.
3.3574	Acetic acid, methyl ester	5200164.9	81.7	79-20-9	C3H6O2	

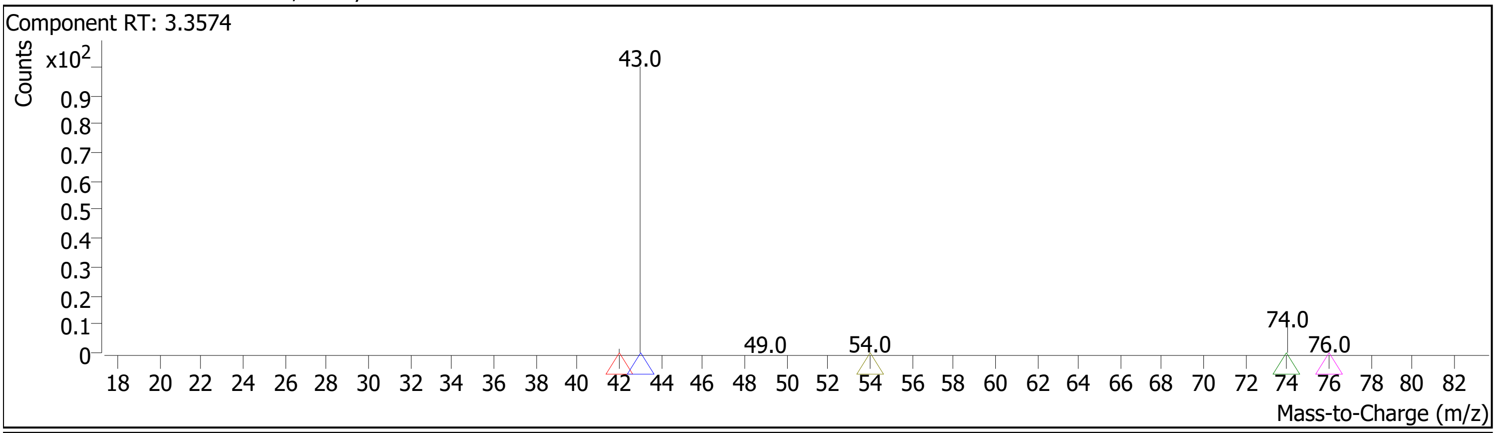

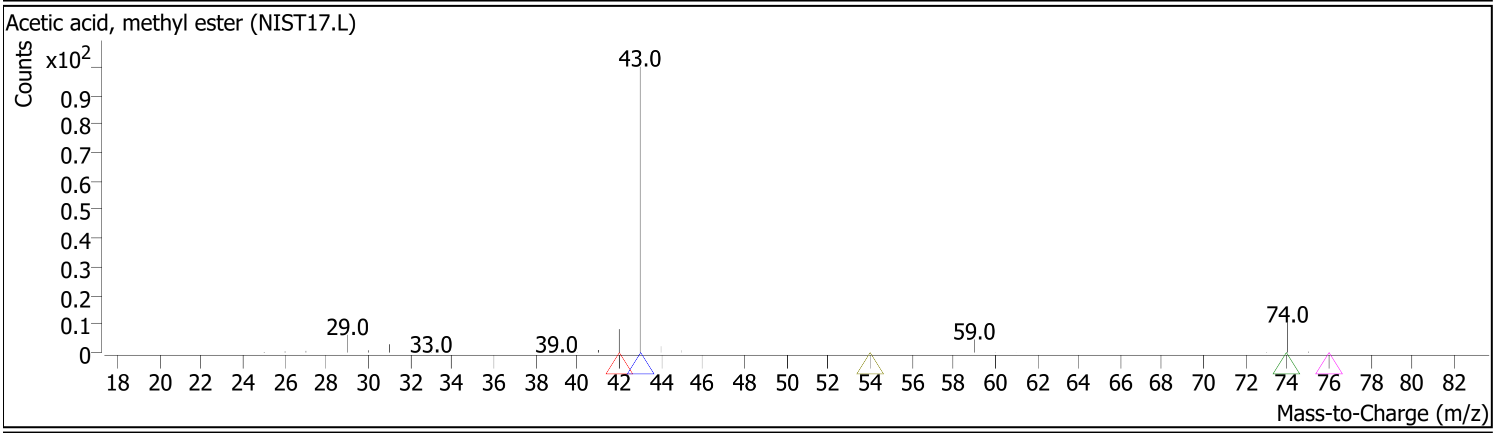

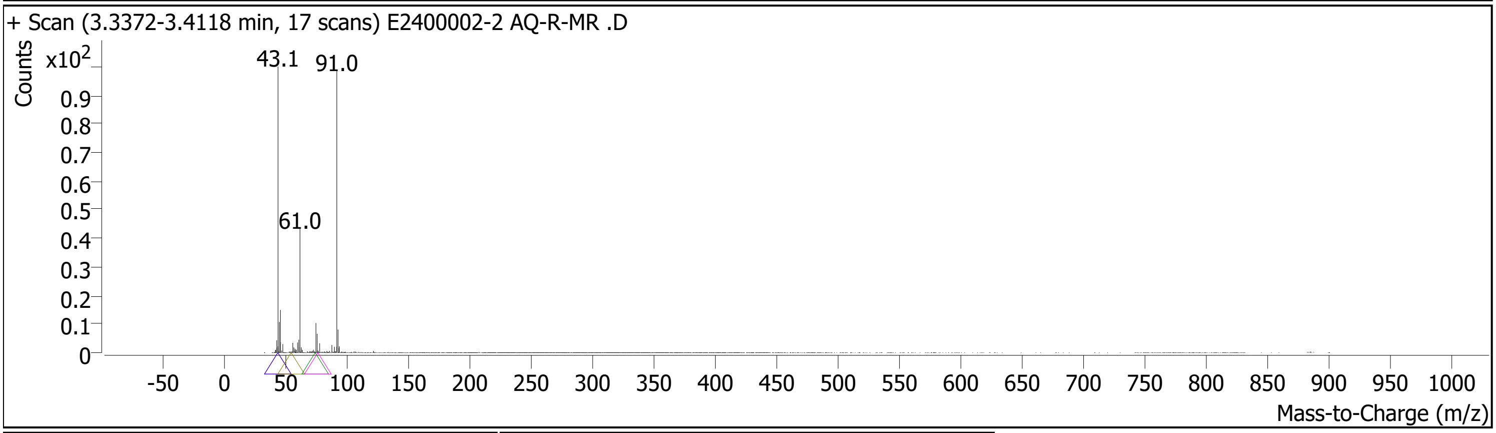

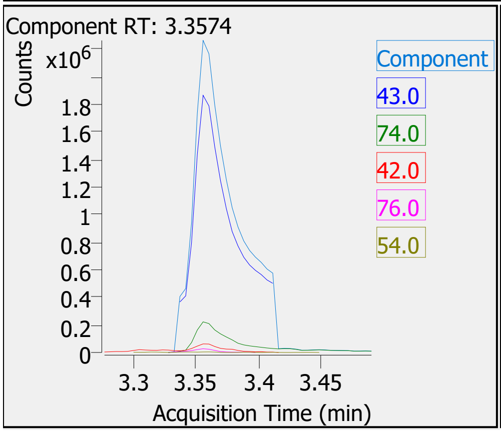

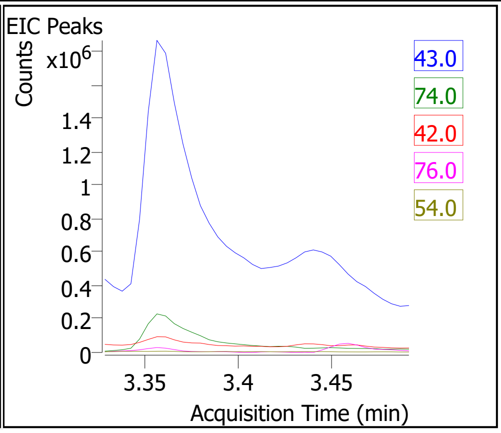

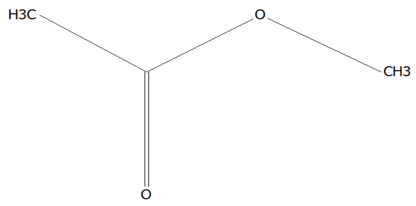

Component RT	Compound Name	Component Area	Match Factor	CAS#	Formula	Estimated Conc.
3.4162	Phenol, 4-ethyl-2-methyl-	140396.2	67.0	2219-73-0	C9H12O	

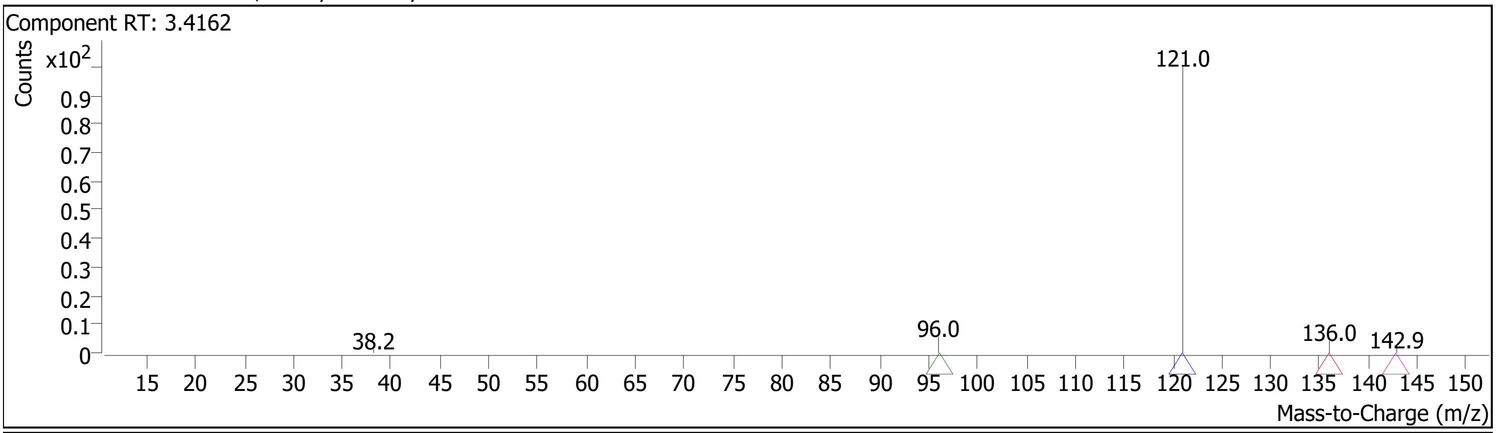

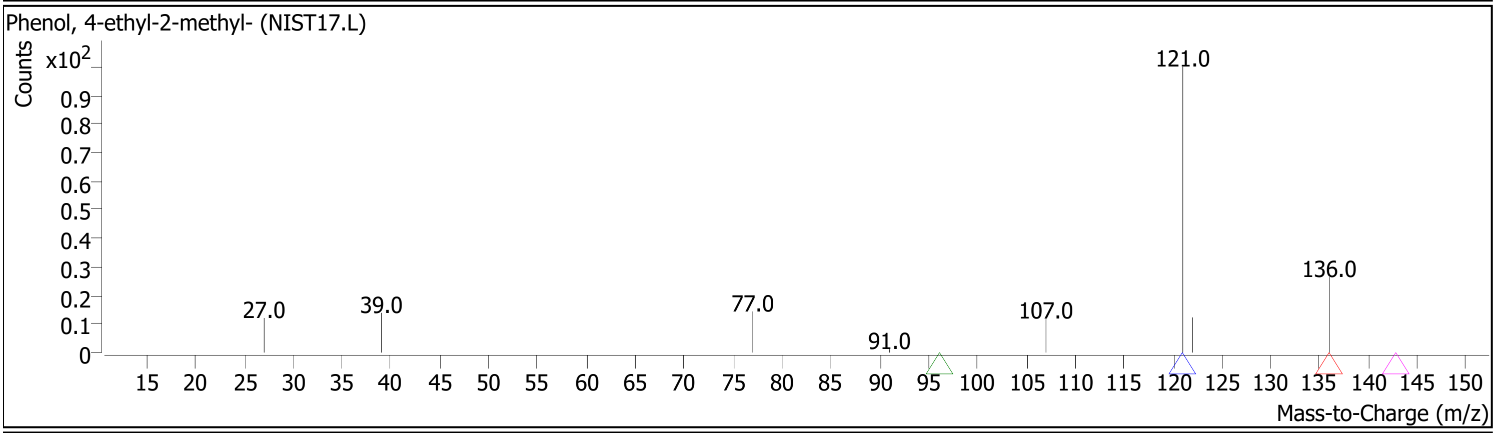

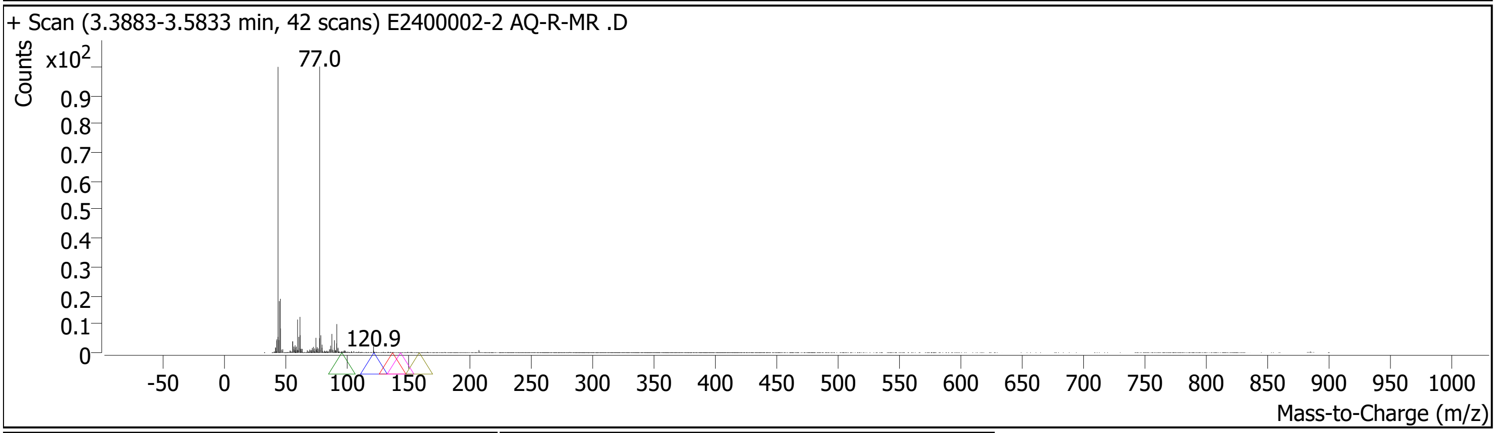

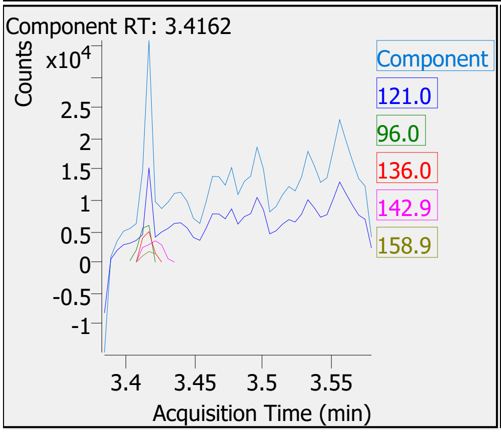

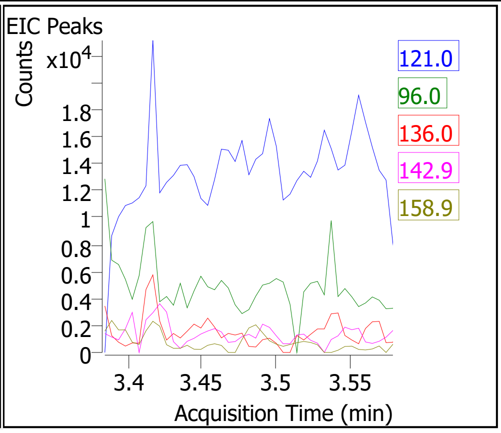

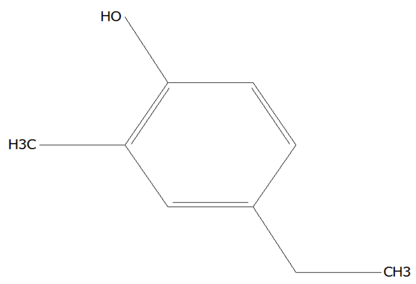

Component RT	Compound Name	Component Area	Match Factor	CAS#	Formula	Estimated Conc.
3.4395	Hydrogen isocyanate	2424964.3	78.7	75-13-8	CHNO	

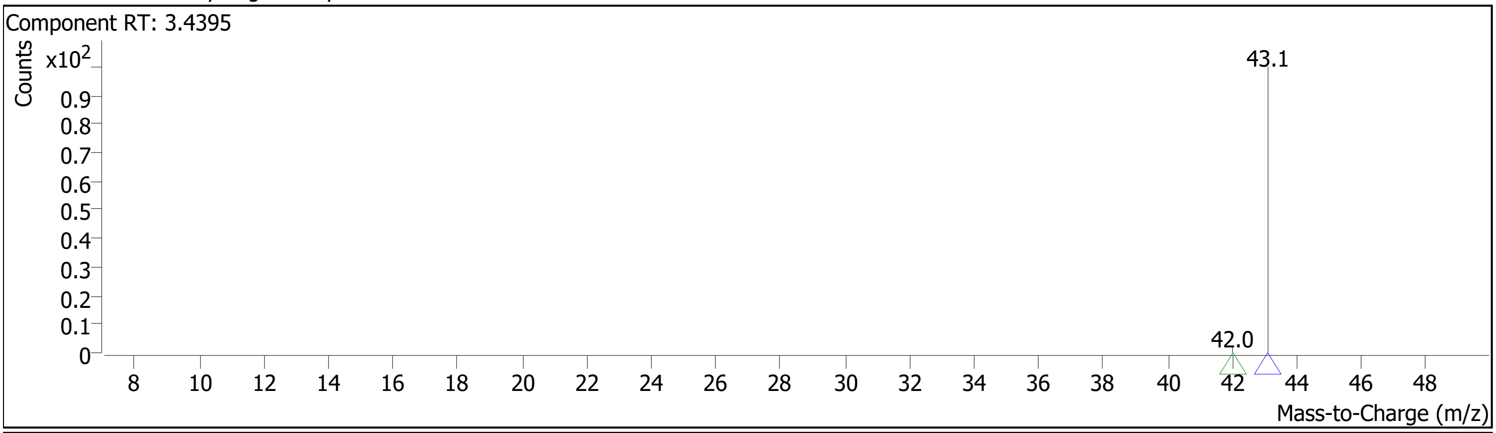

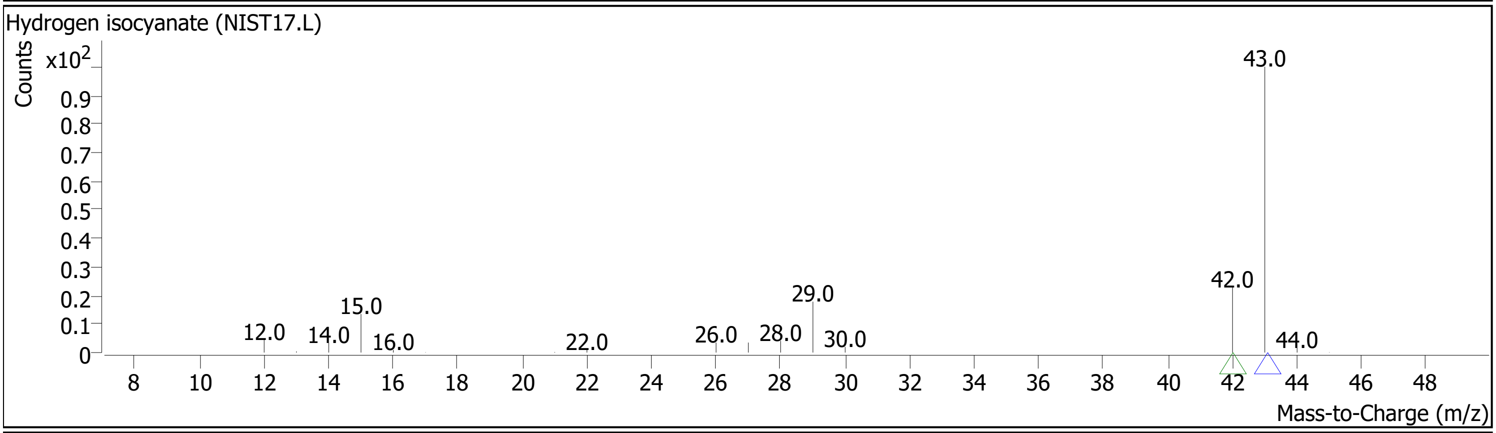

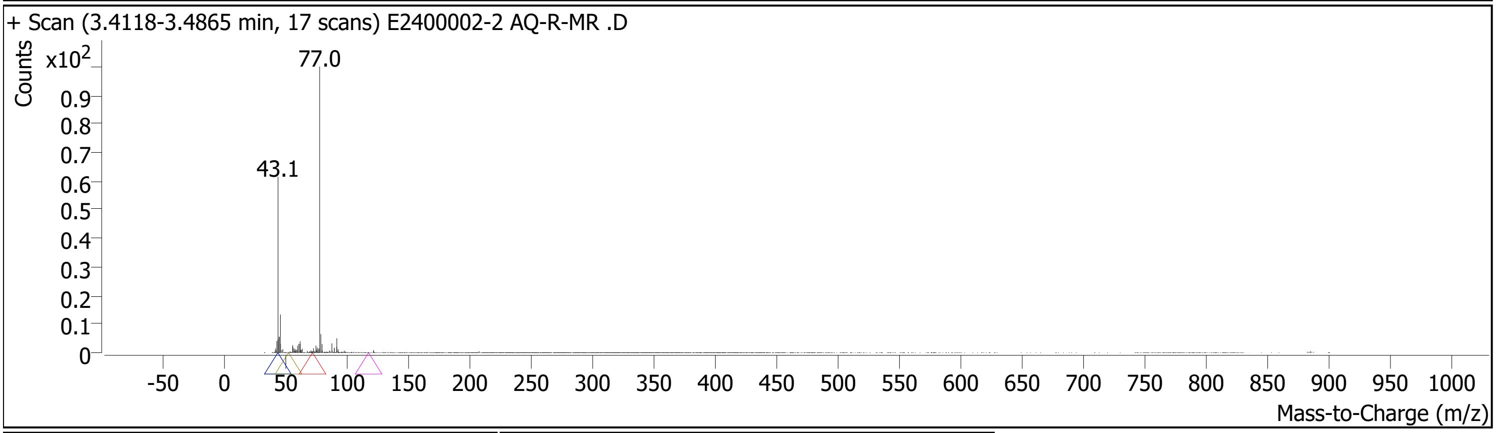

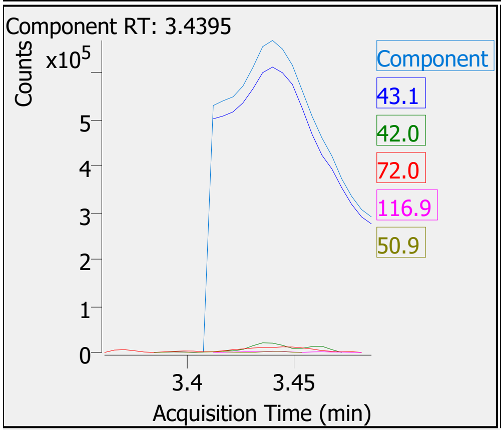

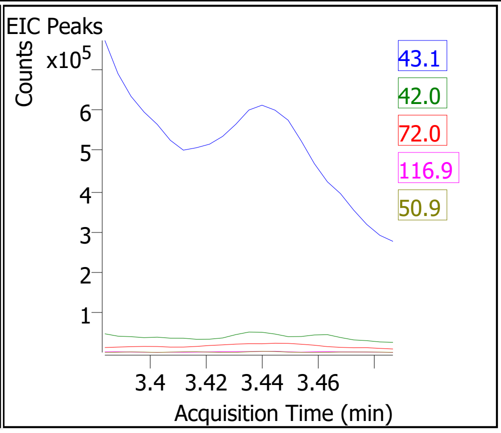

Component RT	Compound Name	Component Area	Match Factor	CAS#	Formula	Estimated Conc.
3.4572	Silanediol, dimethyl-	5397608.0	98.2	1066-42-8	C2H8O2Si	

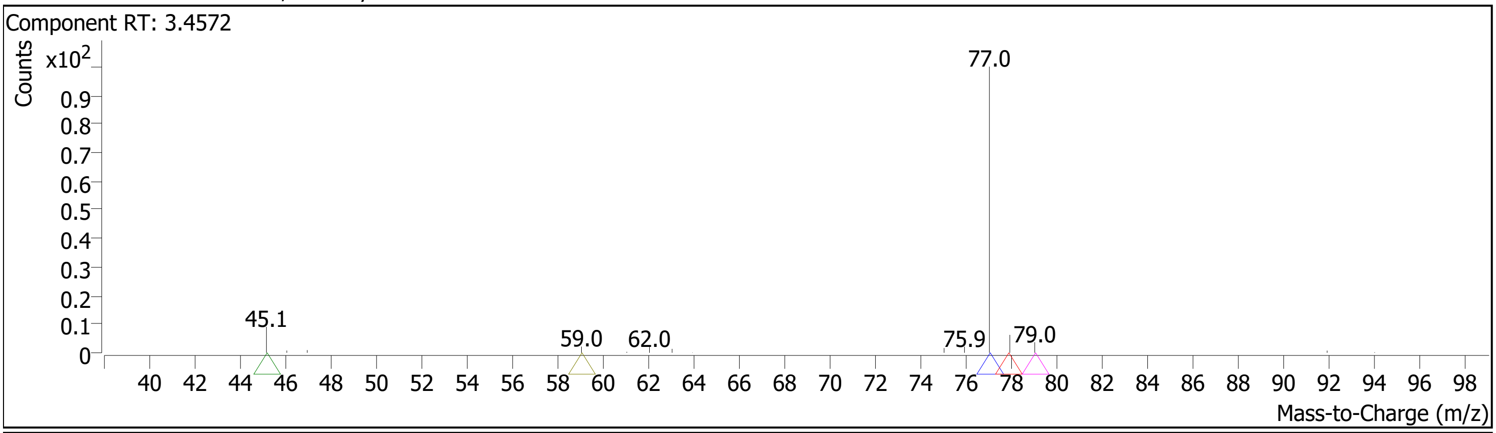

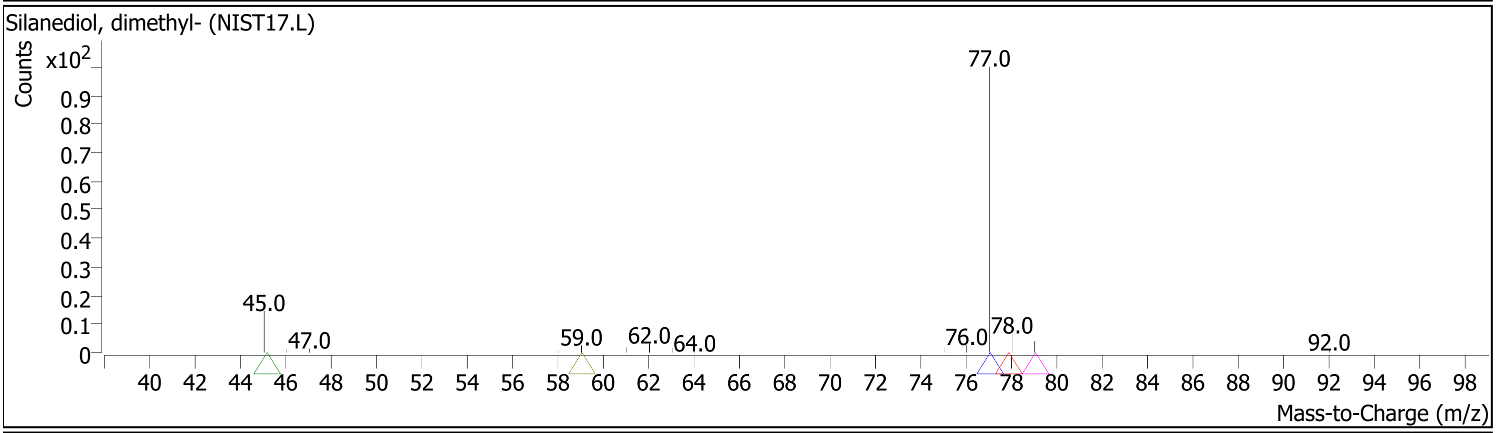

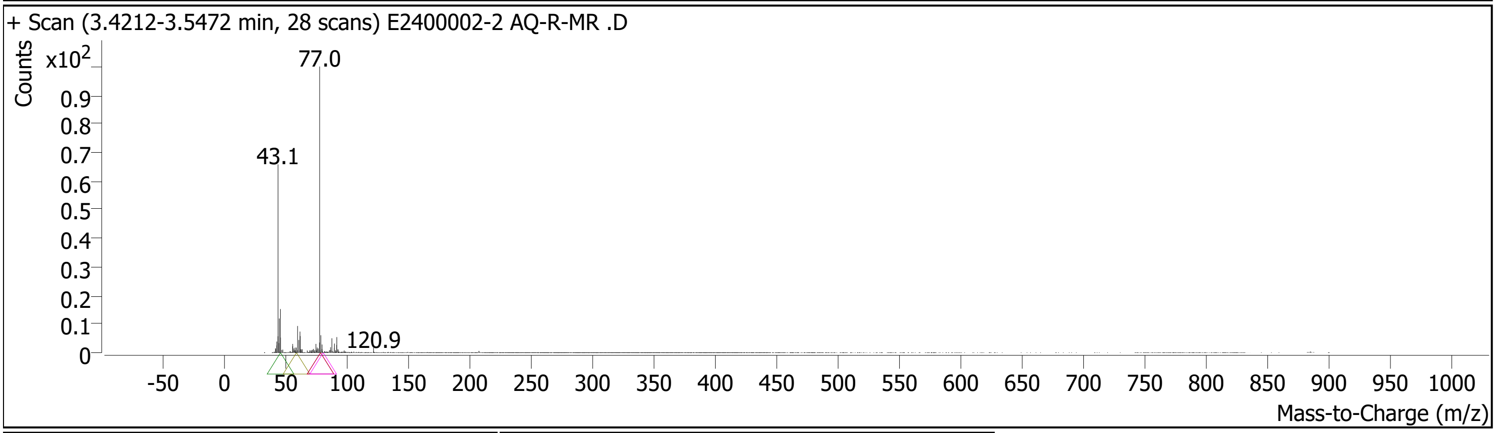

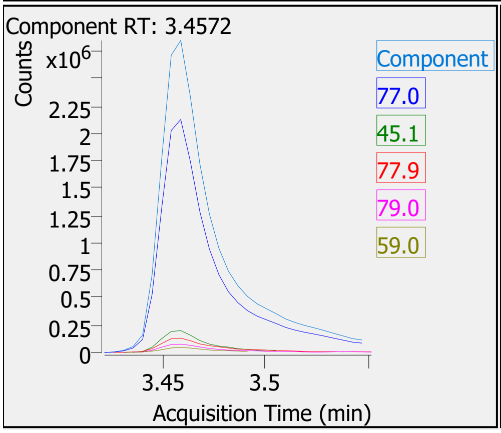

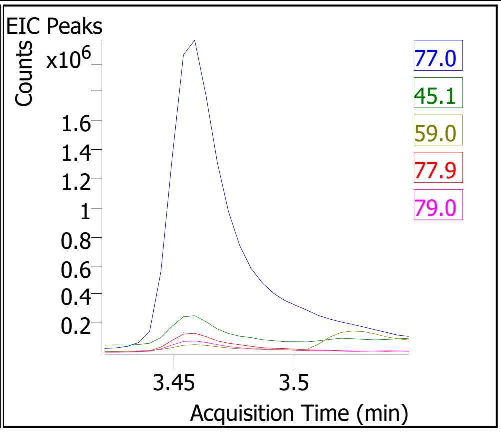

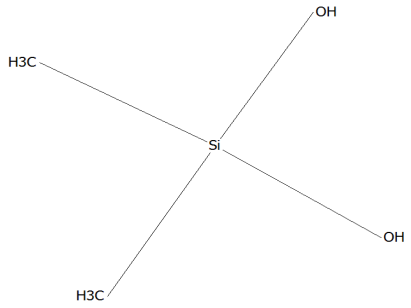

Component RT	Compound Name	Component Area	Match Factor	CAS#	Formula	Estimated Conc.
3.5114	Propane-1,1-diol diacetate	1667313.3	63.4	33931-80-5	C7H12O4	

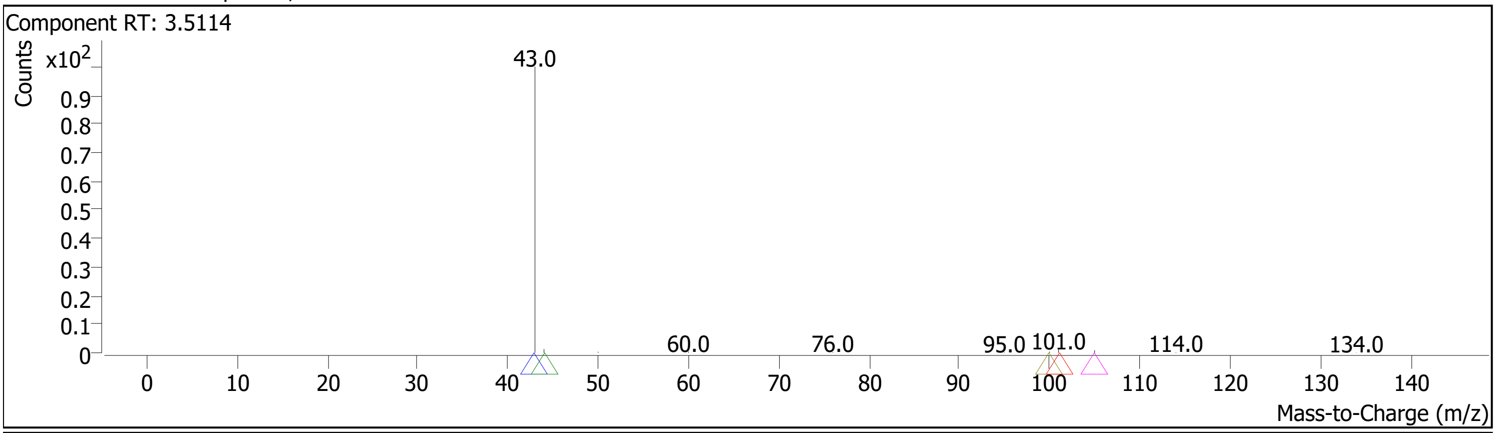

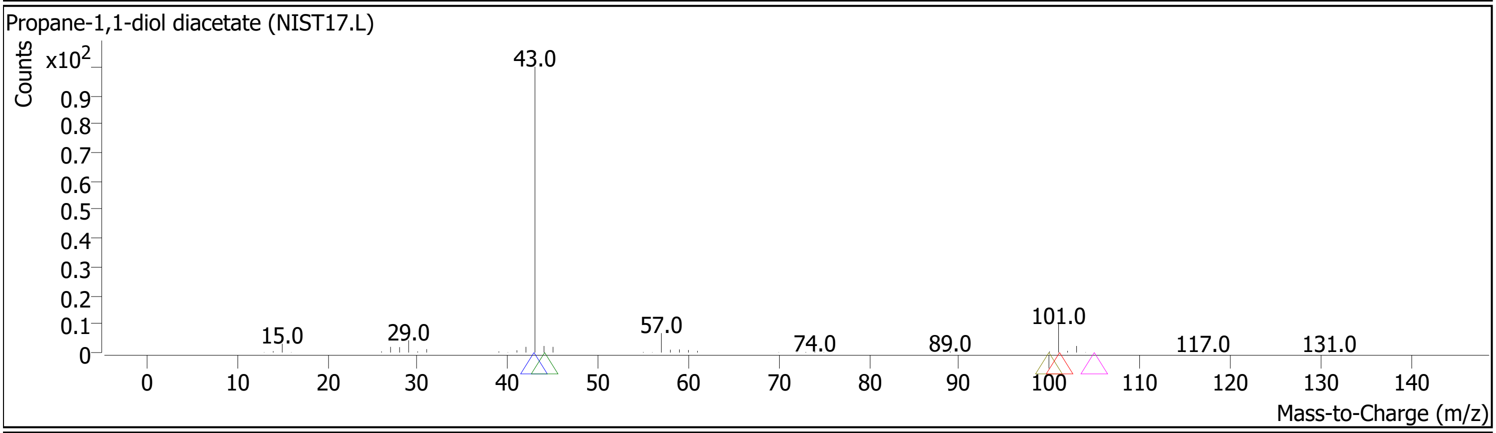

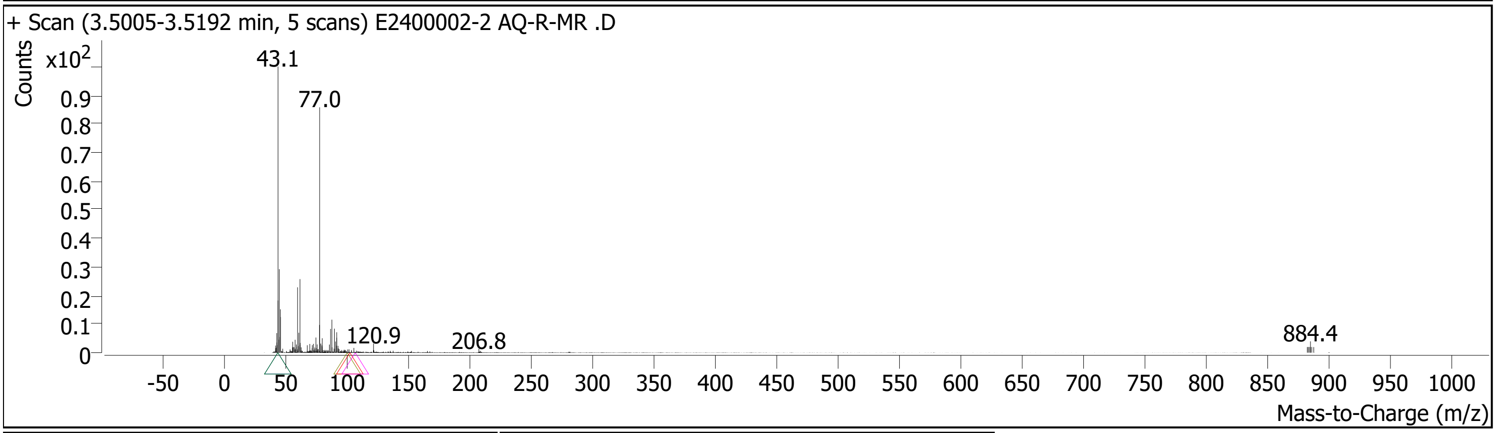

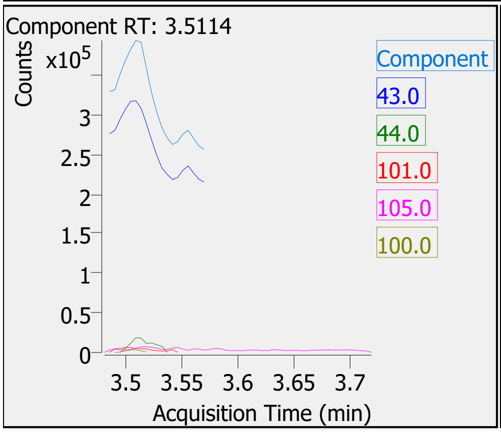

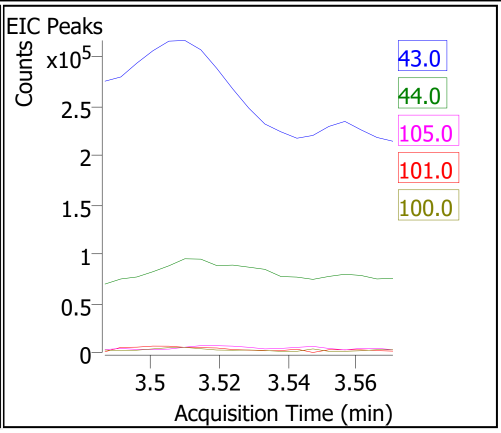

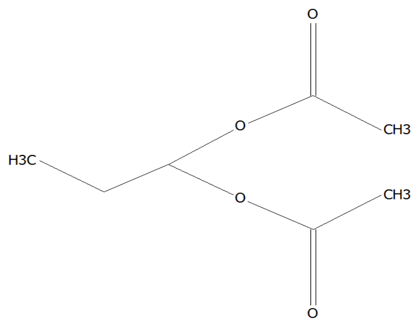

Component RT	Compound Name	Component Area	Match Factor	CAS#	Formula	Estimated Conc.
3.5207	Diacetyl sulphide	3322957.9	65.7	3232-39-1	C4H6O2S	

Component RT: 3.5207

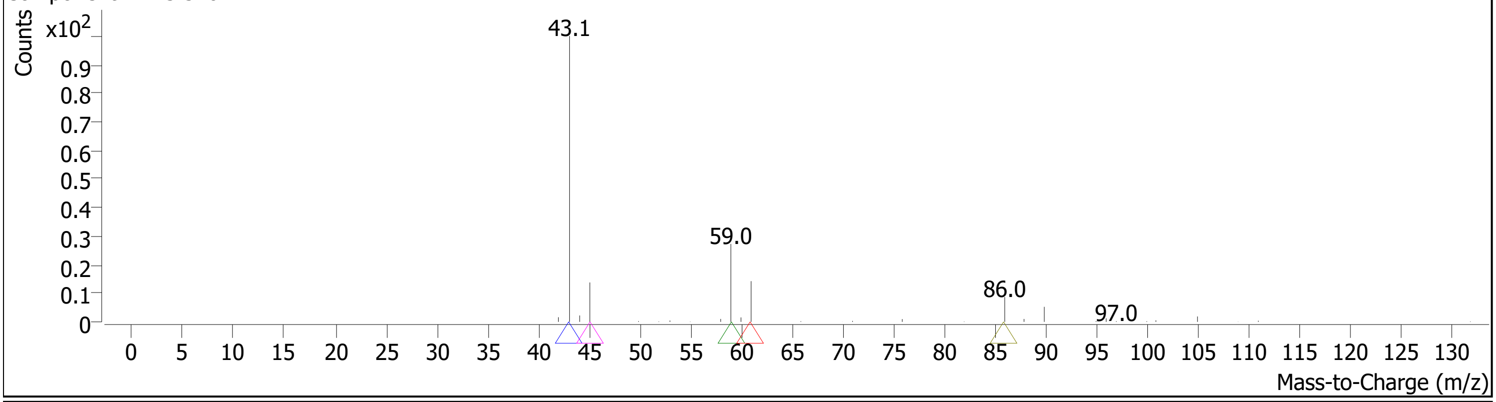

Diacetyl sulphide (NIST17.L)

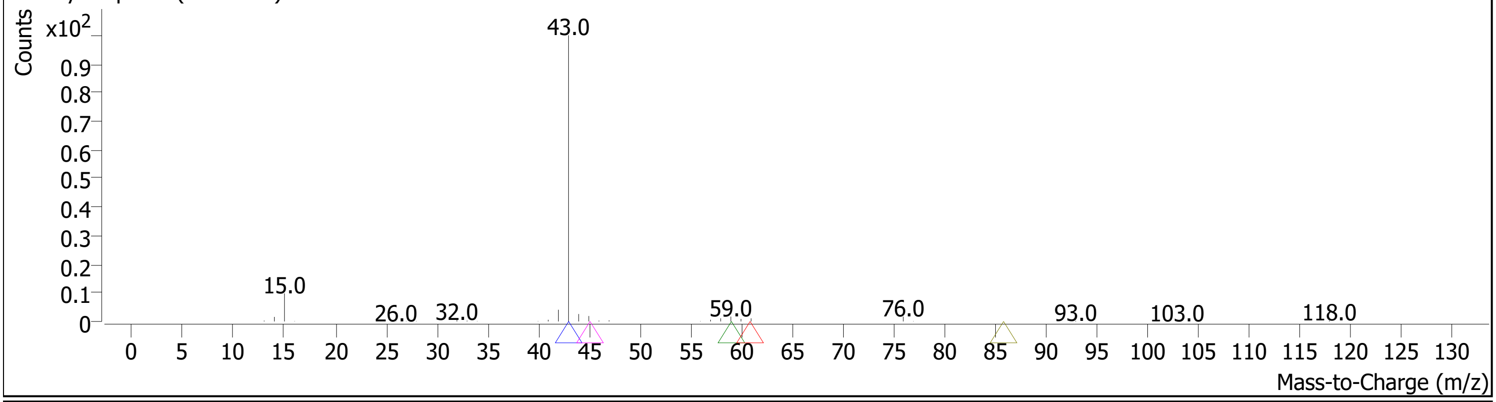

+ Scan (3.4983-3.5659 min, 15 scans) E2400002-2 AQ-R-MR .D

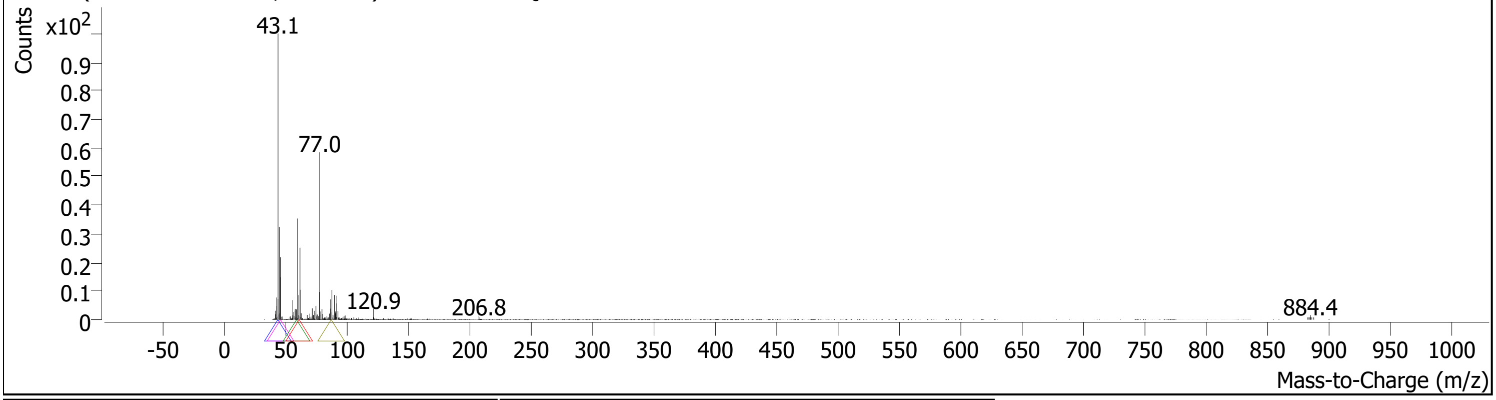

Component RT: 3.5207

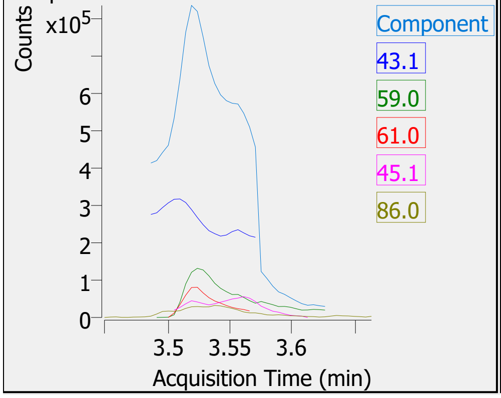

EIC Peaks

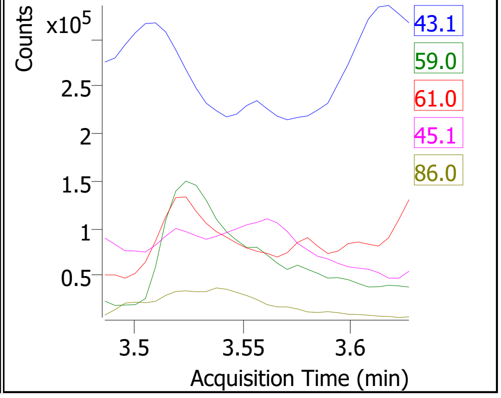

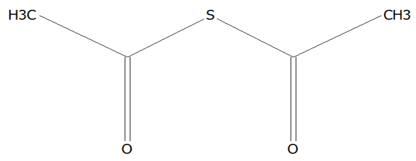

Component RT	Compound Name	Component Area	Match Factor	CAS#	Formula	Estimated Conc.
3.6686	n-Propyl acetate	21325914.1	76.4	109-60-4	C5H10O2	

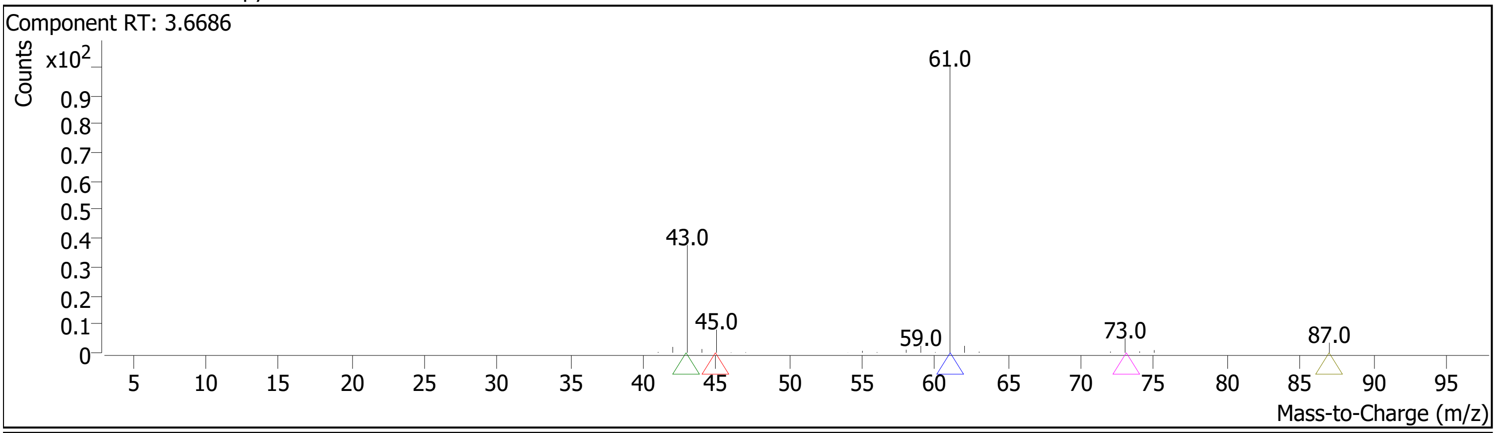

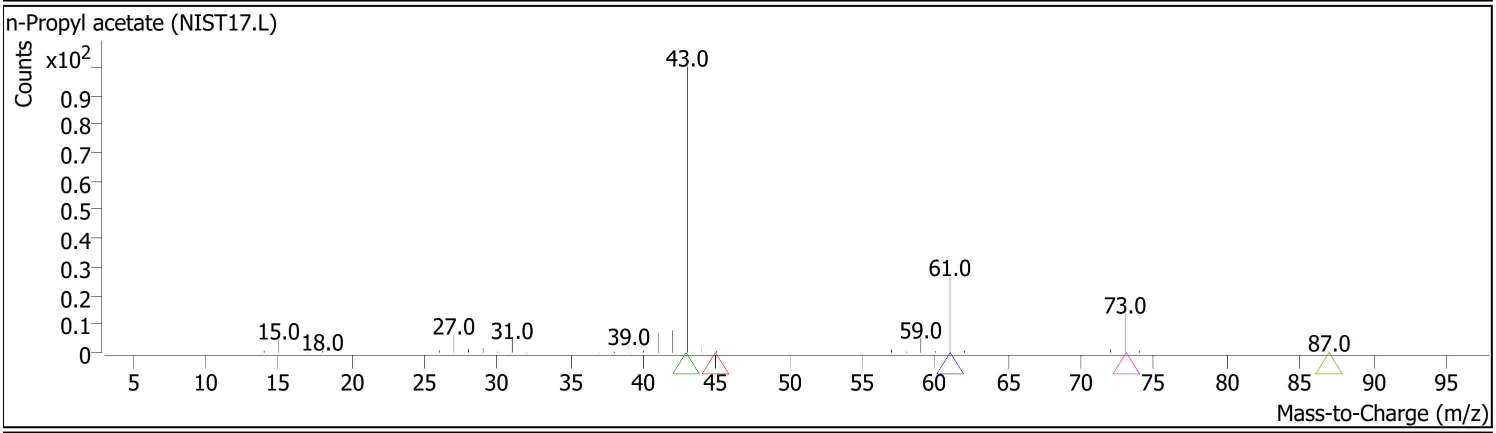

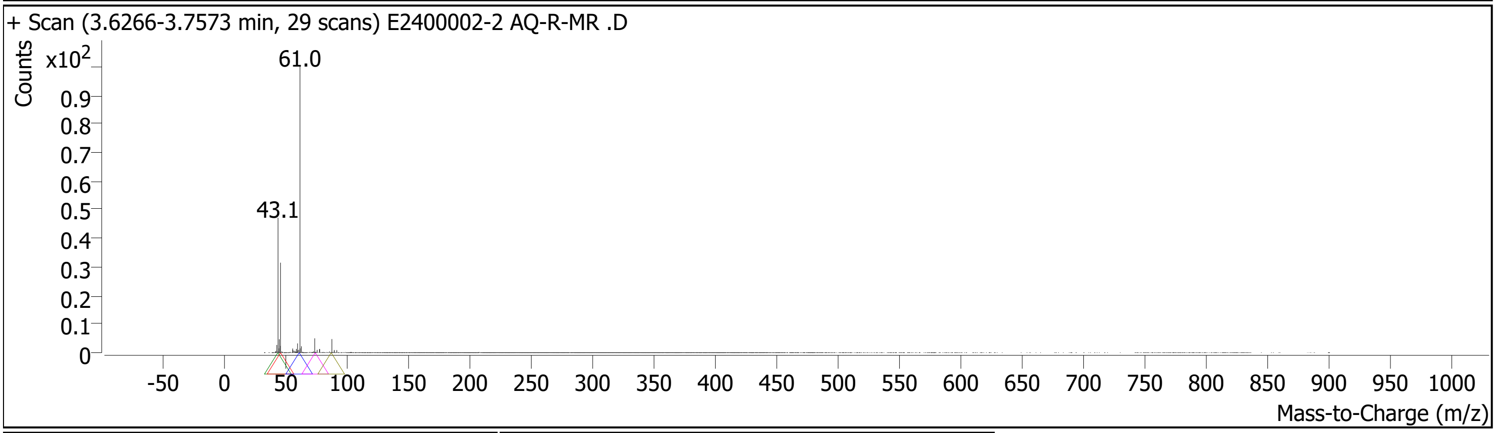

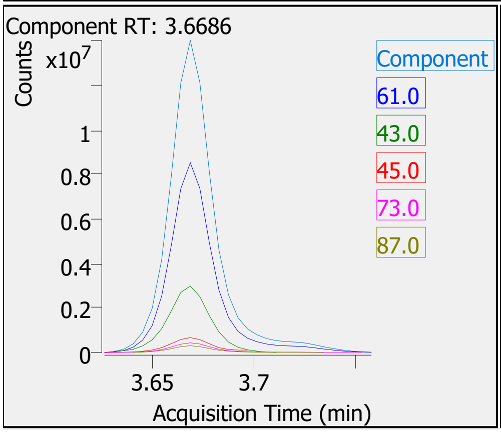

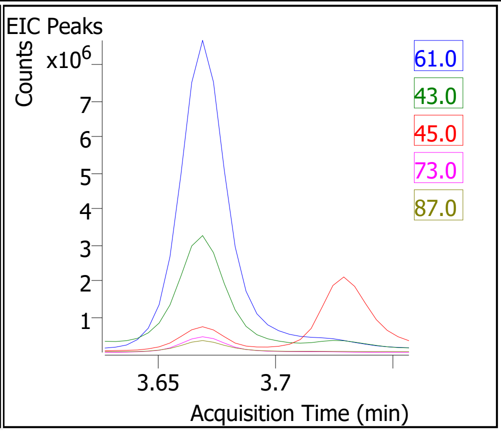

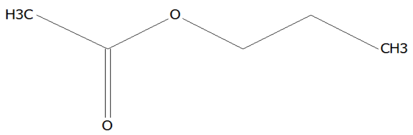

Library Search Results - NonTarget Hits with Details

Component RT	Compound Name	Component Area	Match Factor	CAS#	Formula	Estimated Conc.
3.7292	L-Lactic acid	4266813.2	82.9	79-33-4	C3H6O3	

Component RT: 3.7292

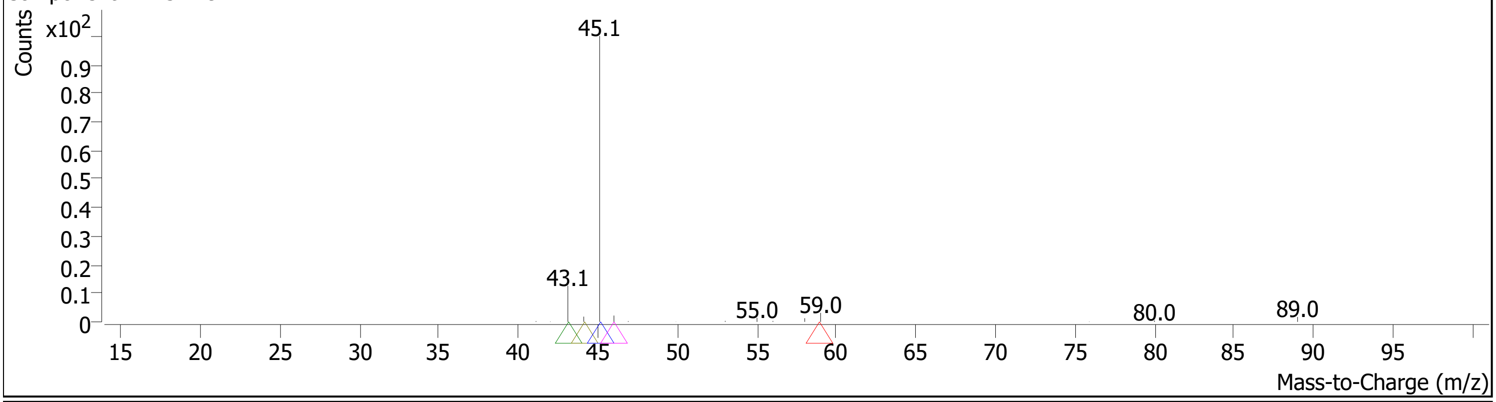

L-Lactic acid (NIST17.L)

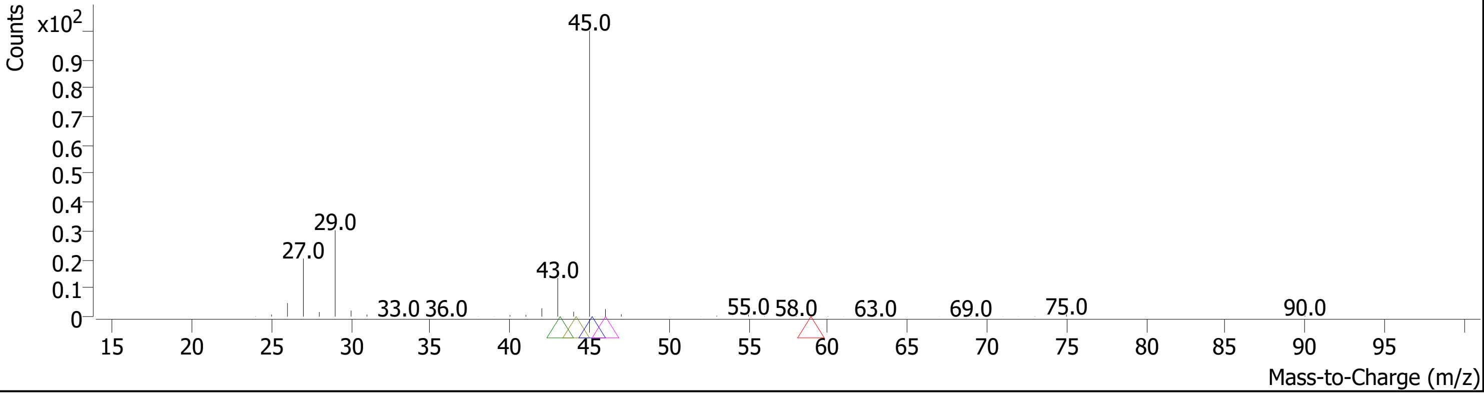

+ Scan (3.6966-3.8180 min, 27 scans) E2400002-2 AQ-R-MR .D

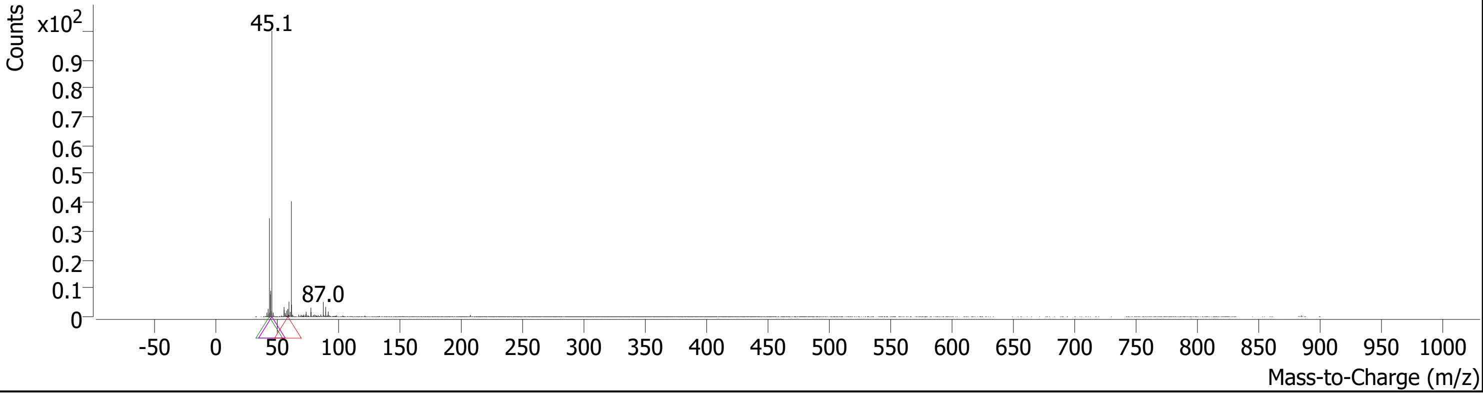

Component RT: 3.7292

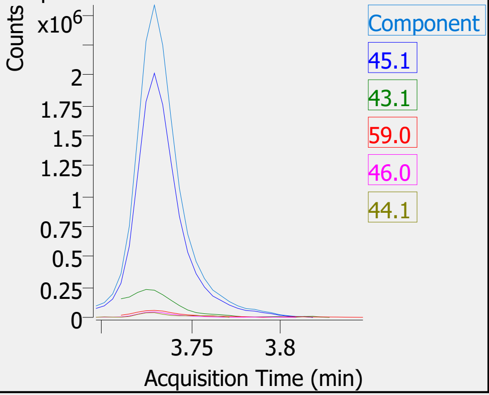

EIC Peaks

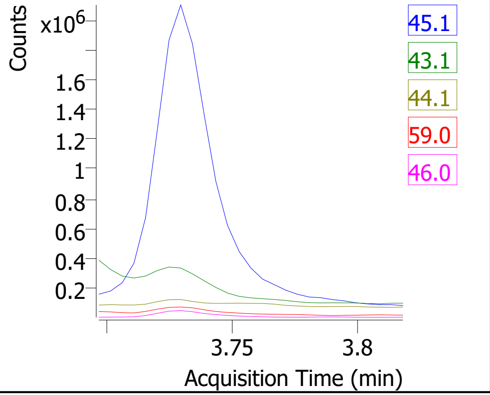

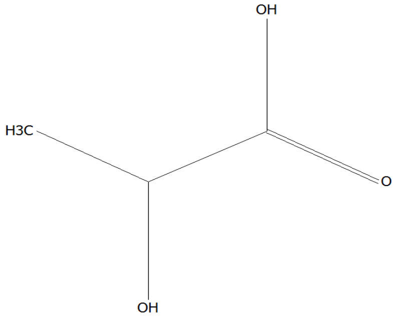

Library Search Results - NonTarget Hits with Details

Component RT	Compound Name	Component Area	Match Factor	CAS#	Formula	Estimated Conc.
3.8623	1-Phenyl-1-decanol	431747.8	81.3	21078-95-5	C16H26O	

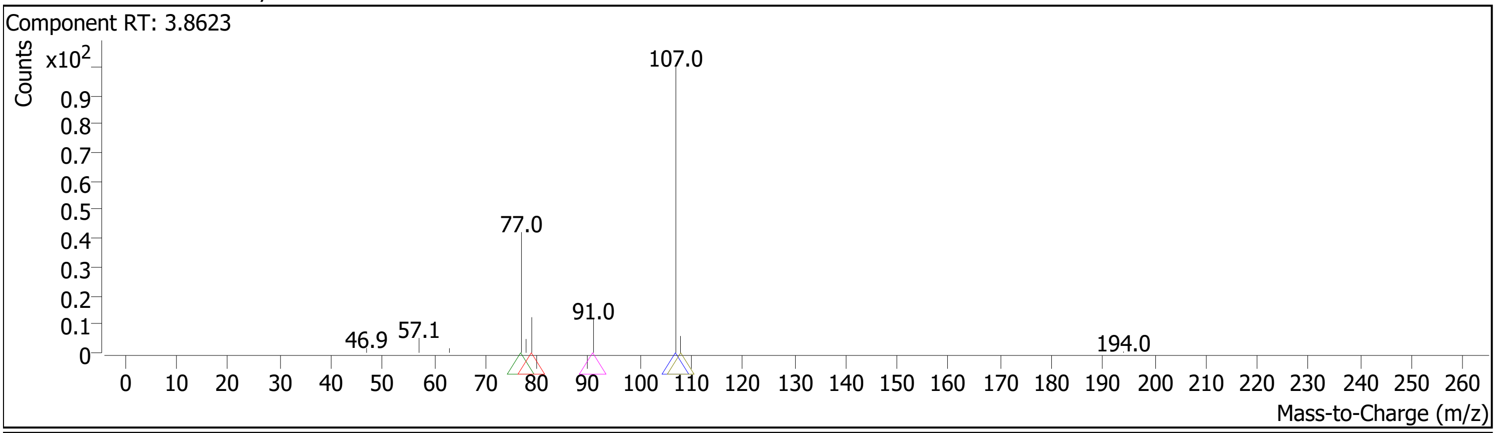

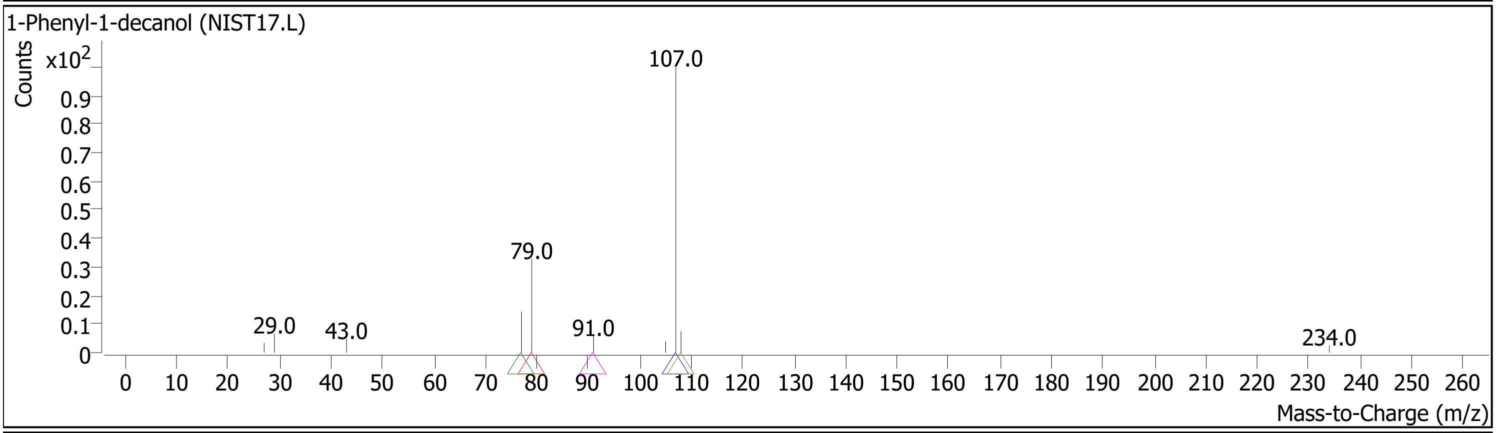

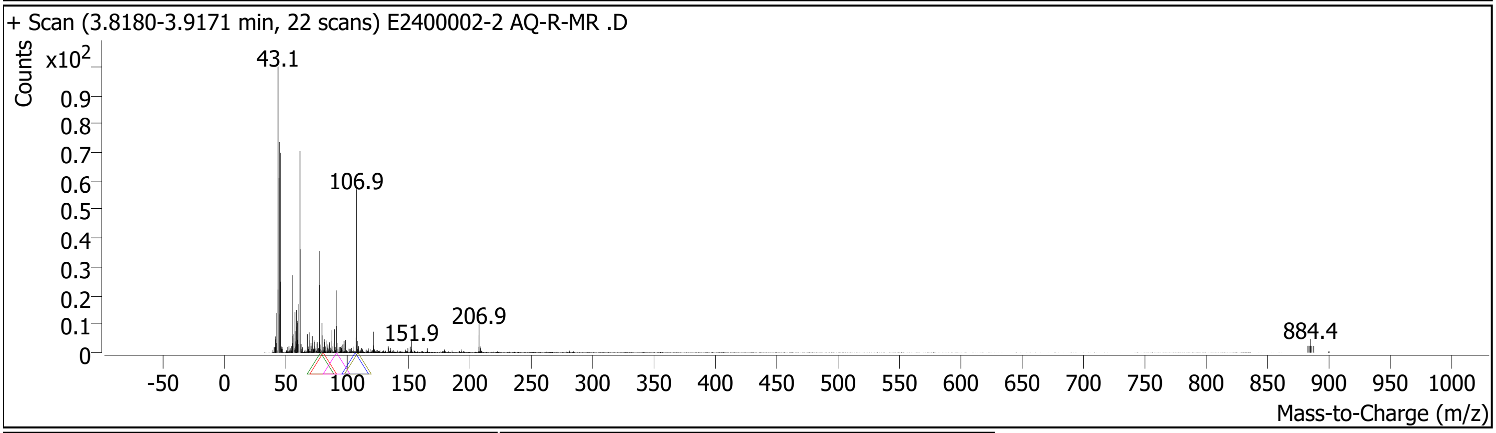

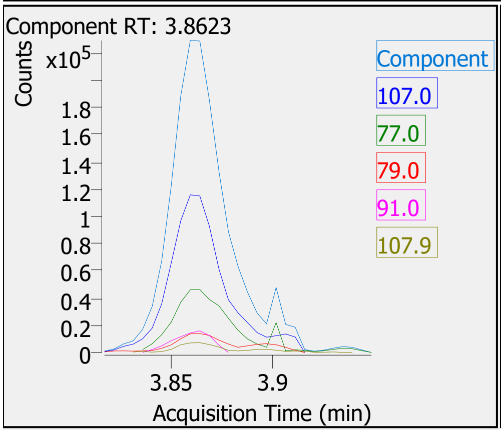

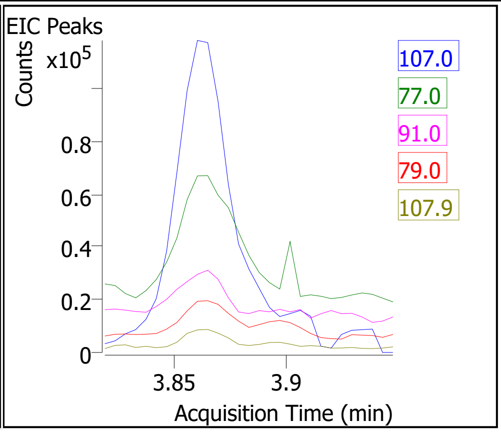

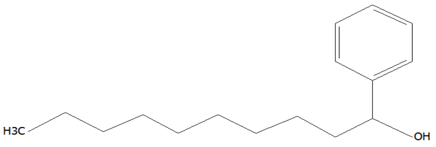

Component RT	Compound Name	Component Area	Match Factor	CAS#	Formula	Estimated Conc.
4.1045	Propanoic acid, 2-oxo-, methyl ester	4825824.0	87.6	600-22-6	C4H6O3	

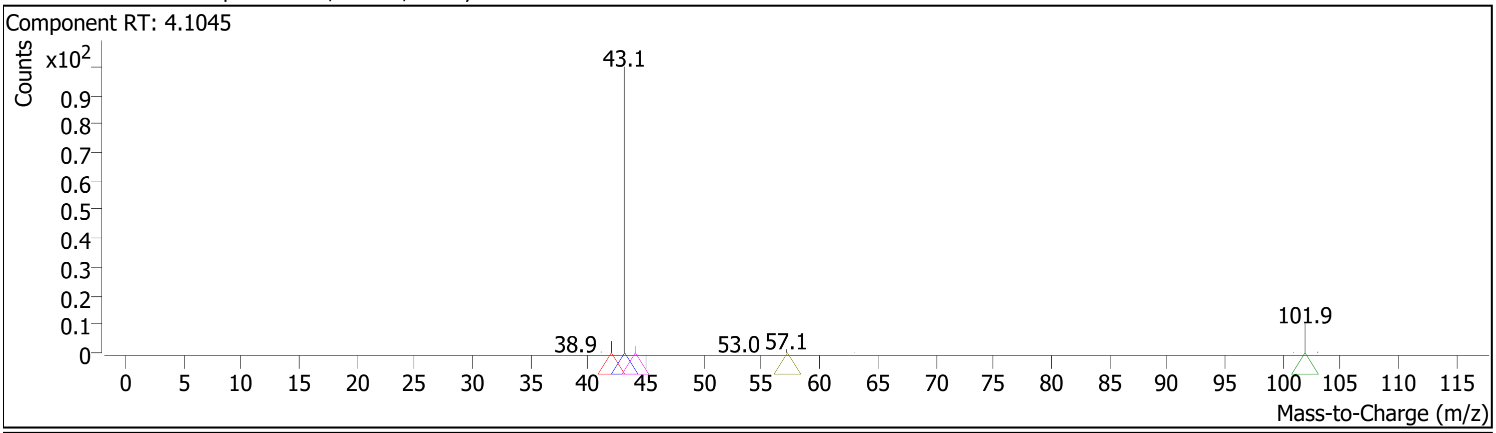

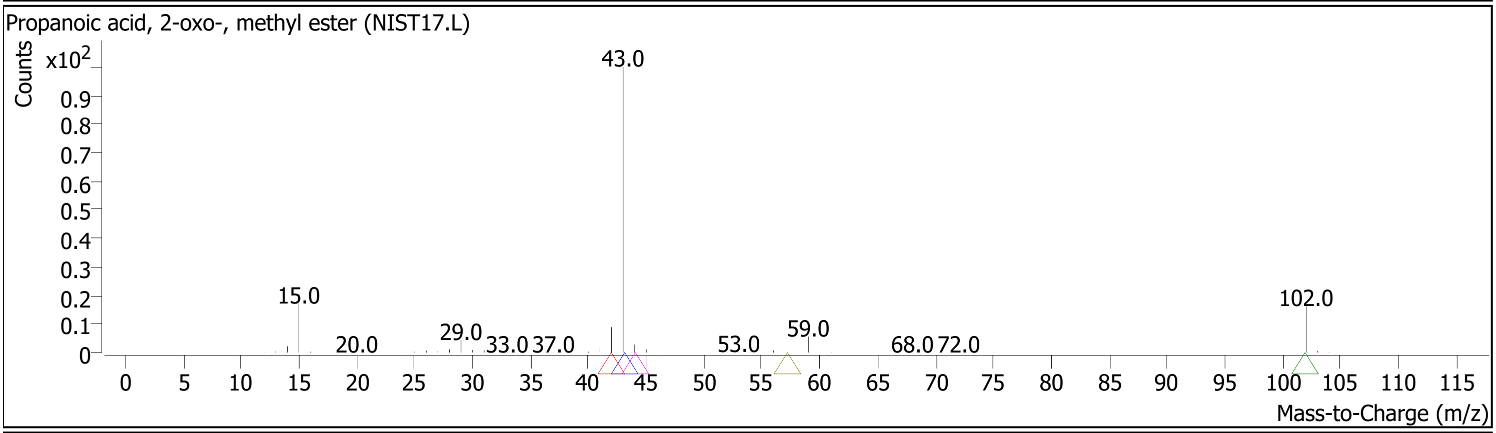

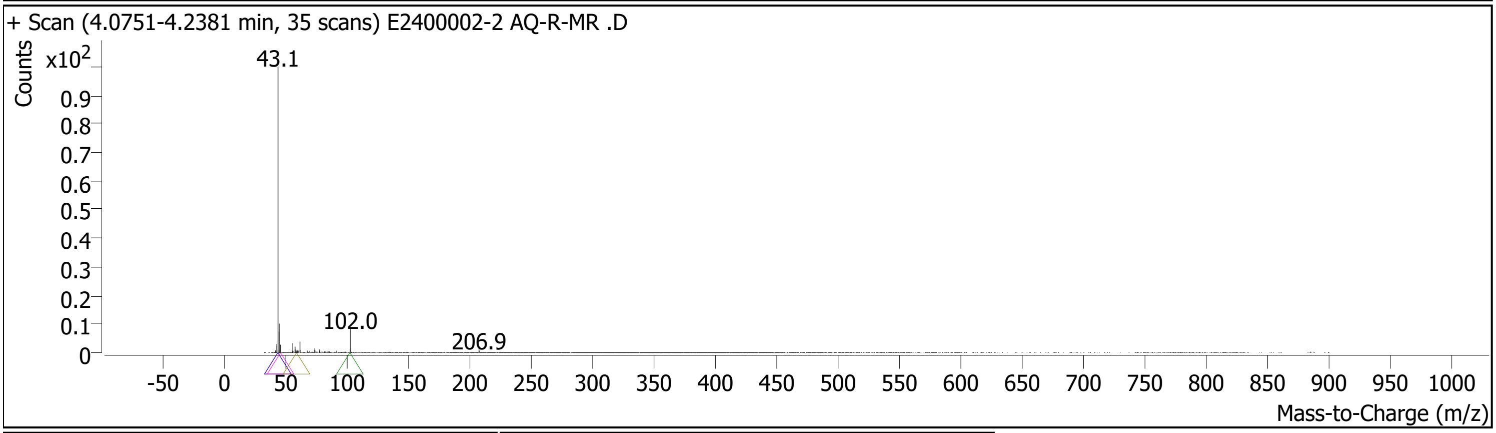

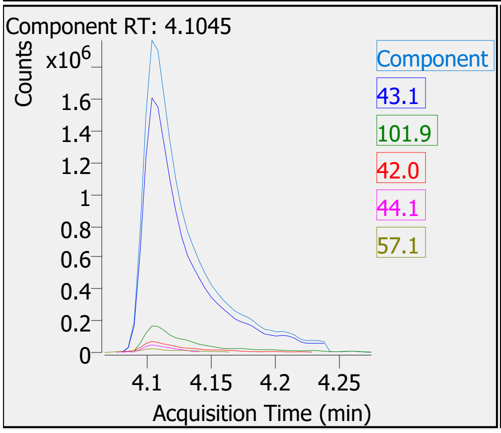

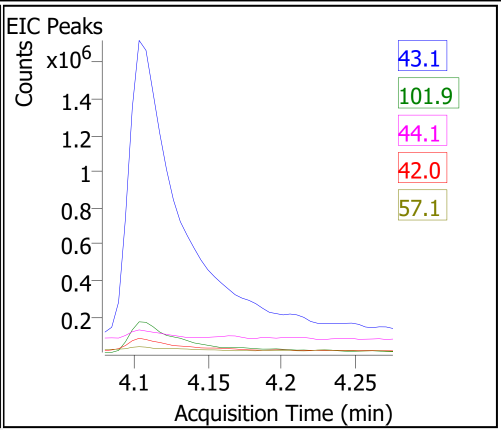

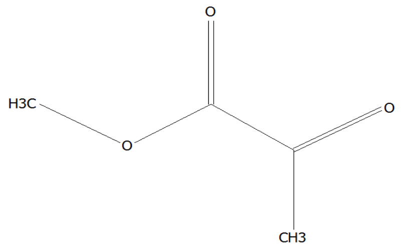

Component RT	Compound Name	Component Area	Match Factor	CAS#	Formula	Estimated Conc.
4.5307	Pyrazine, methyl-	423145.0	77.0	109-08-0	C5H6N2	

Component RT: 4.5307

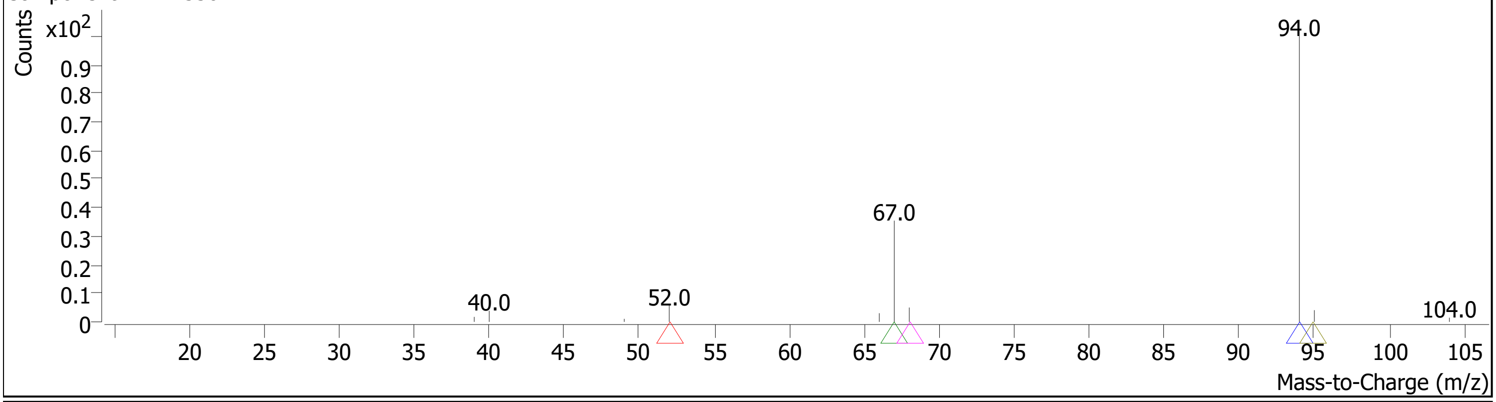

Pyrazine, methyl- (NIST17.L)

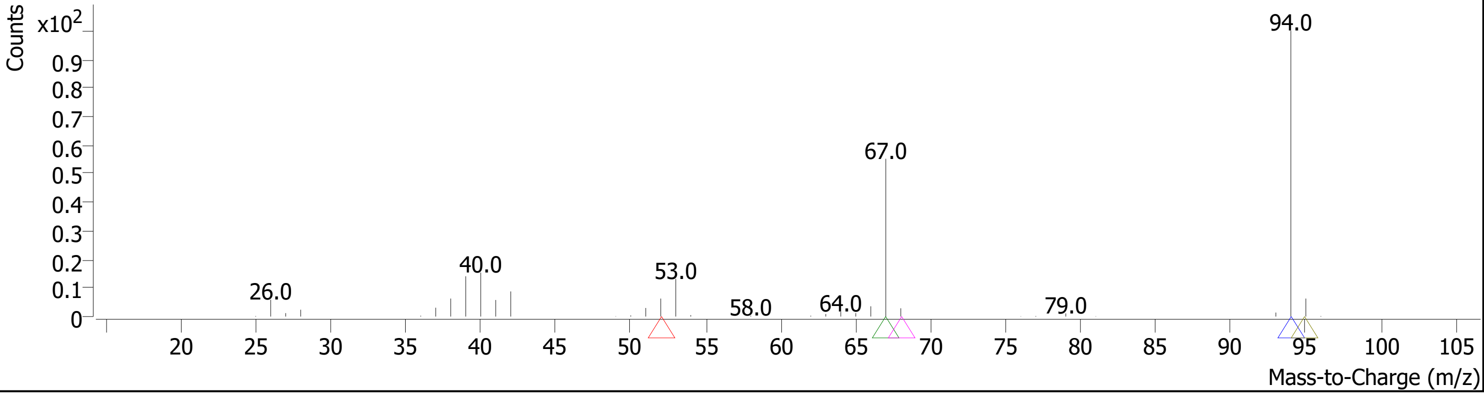

+ Scan (4.5322-4.5508 min, 5 scans) E2400002-2 AQ-R-MR .D

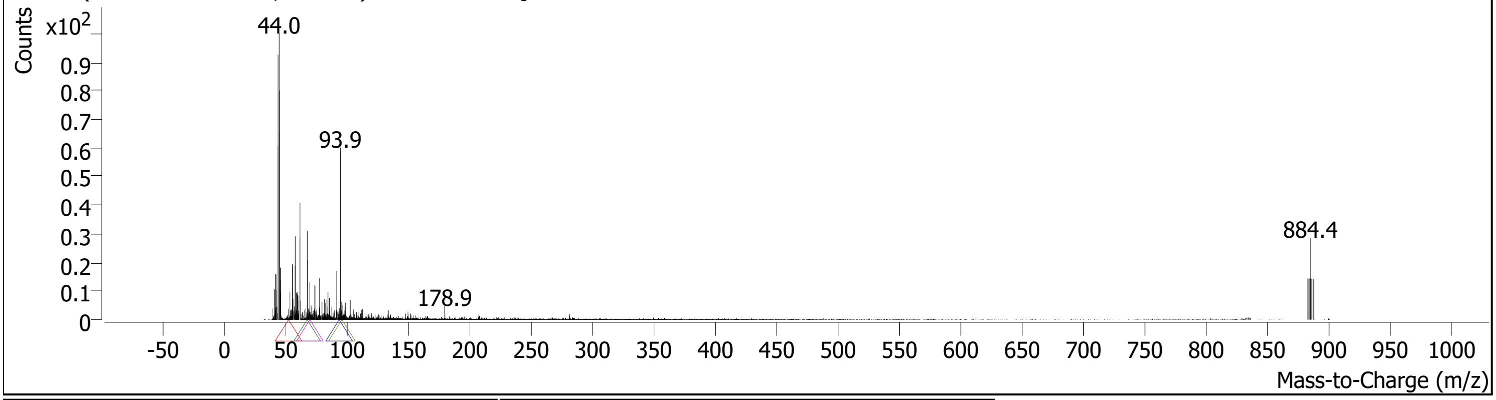

Component RT: 4.5307

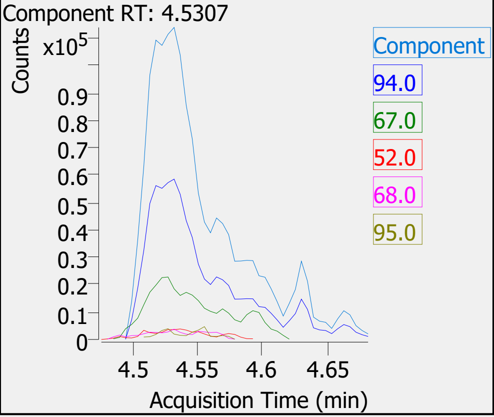

EIC Peaks

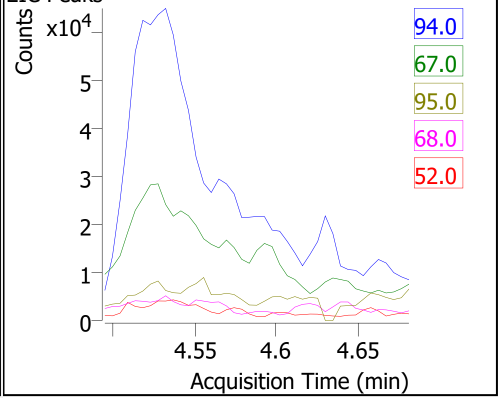

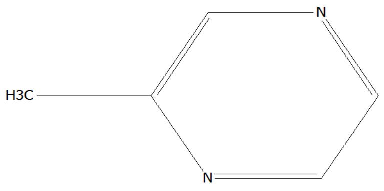

 Generated at 09:38 AM on 04-04-2024

Library Search Results - NonTarget Hits with Details

Component RT	Compound Name	Component Area	Match Factor	CAS#	Formula	Estimated Conc.
5.7694	1-Pentamethyldisilanyl-4-trimethylsiloxybenzene	200910.6	66.1	1000427-15-5	C14H28OSi3	

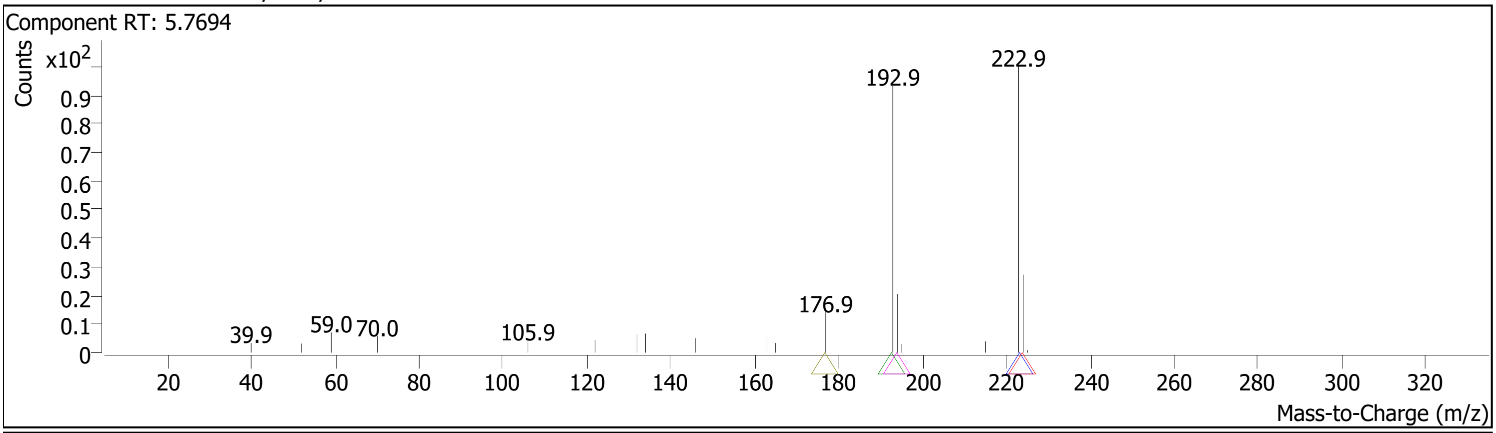

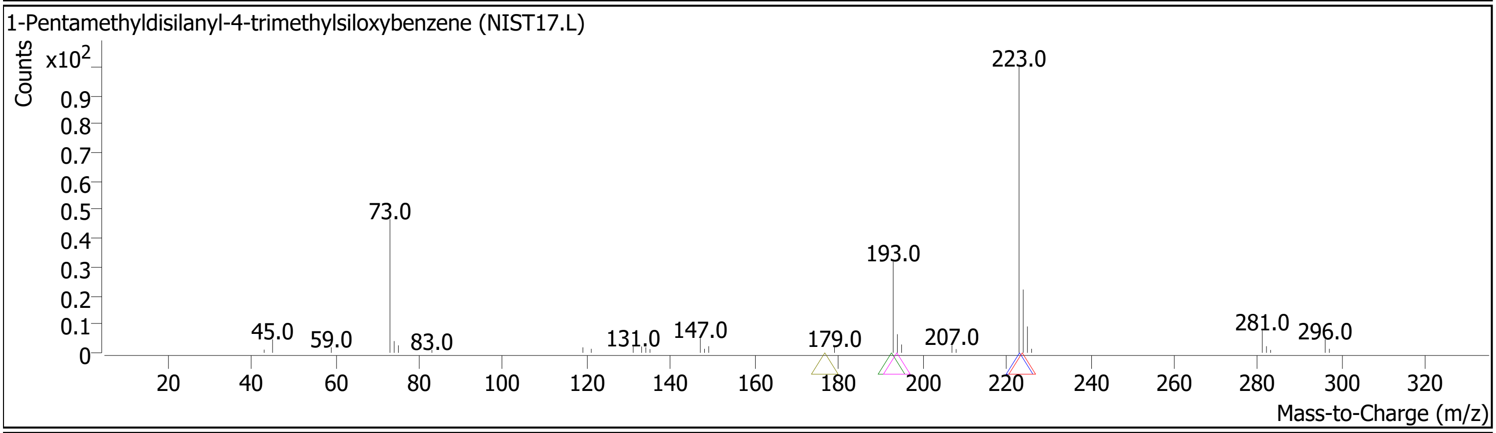

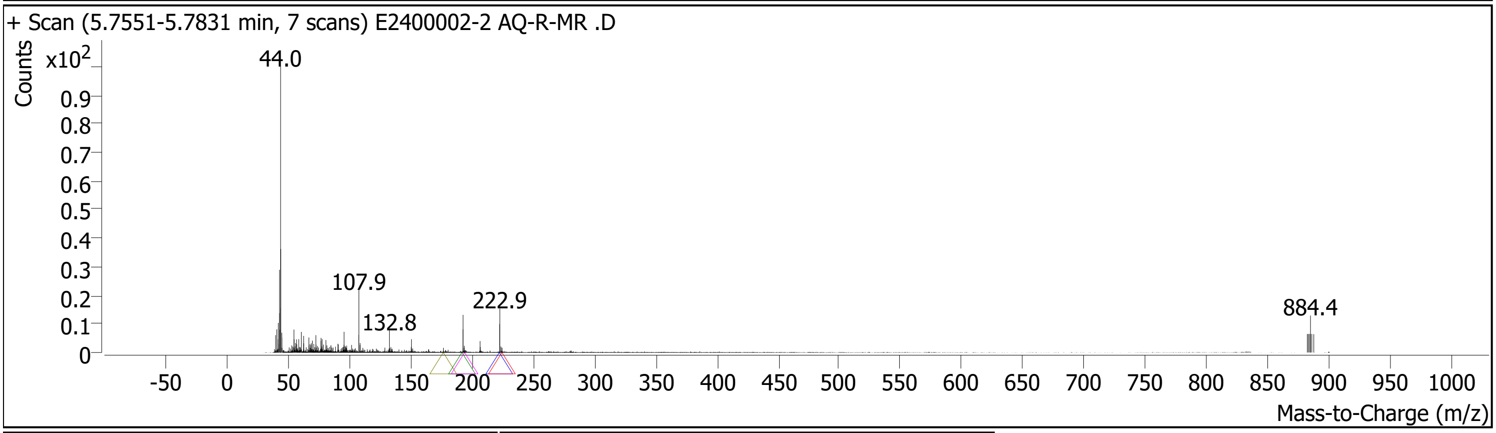

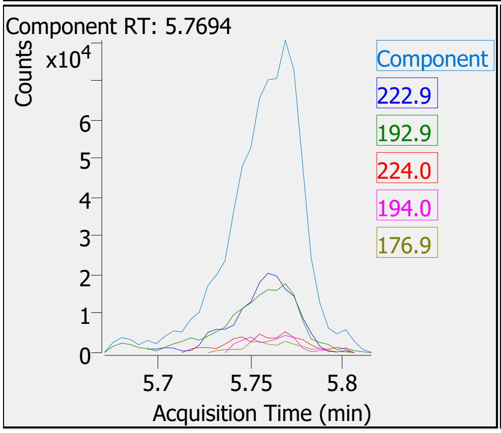

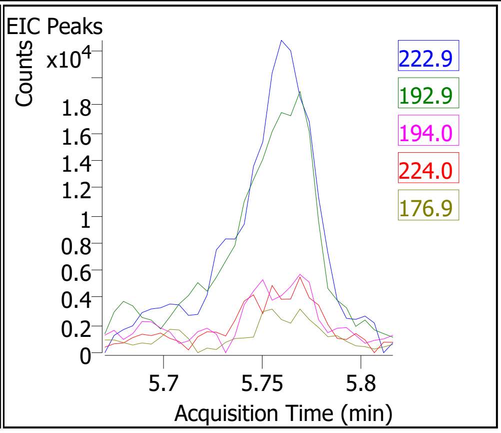

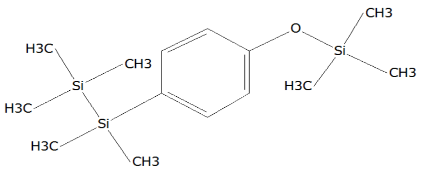

 Generated at 09:38 AM on 04-04-2024

Component RT	Compound Name	Component Area	Match Factor	CAS#	Formula	Estimated Conc.
6.0578	n-Amyl ether	1902494.2	61.9	693-65-2	C10H22O	

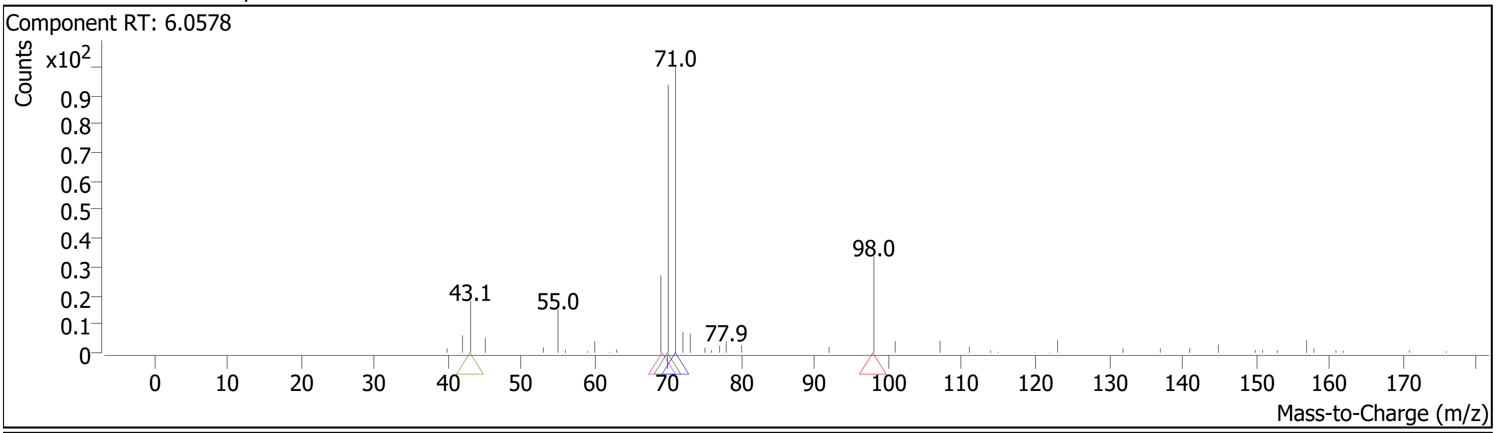

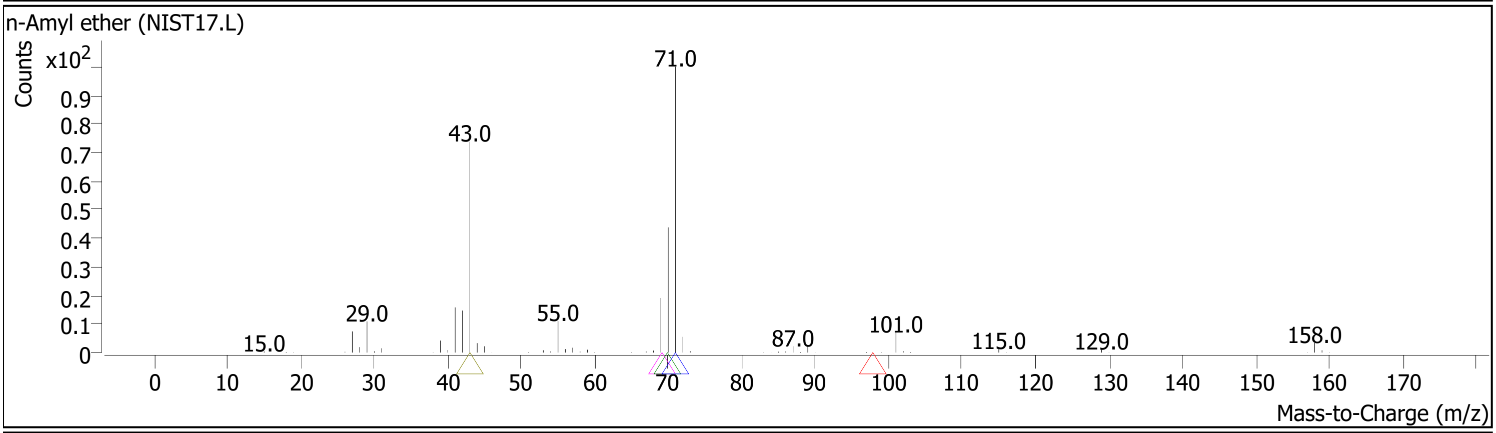

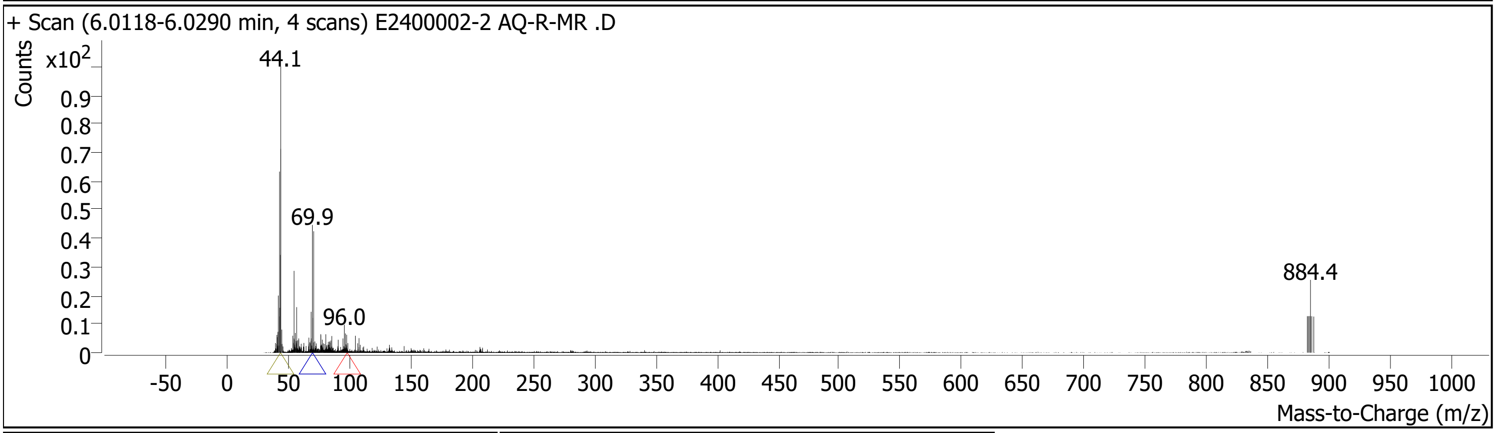

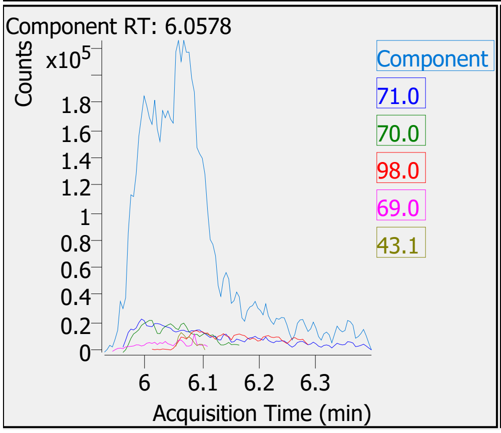

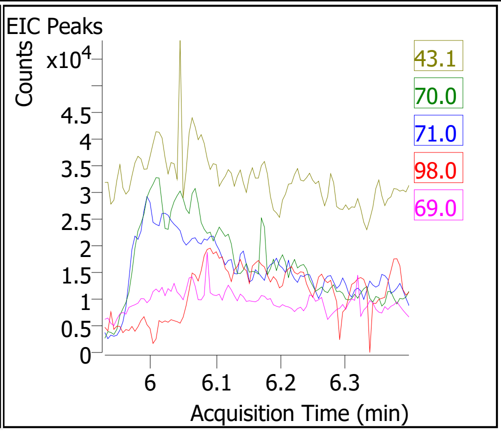

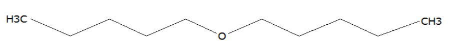

Library Search Results - NonTarget Hits with Details

Component RT	Compound Name	Component Area	Match Factor	CAS#	Formula	Estimated Conc.
6.5923	S-Ethyl ethanethioate	244164.1	69.6	625-60-5	C4H8OS	

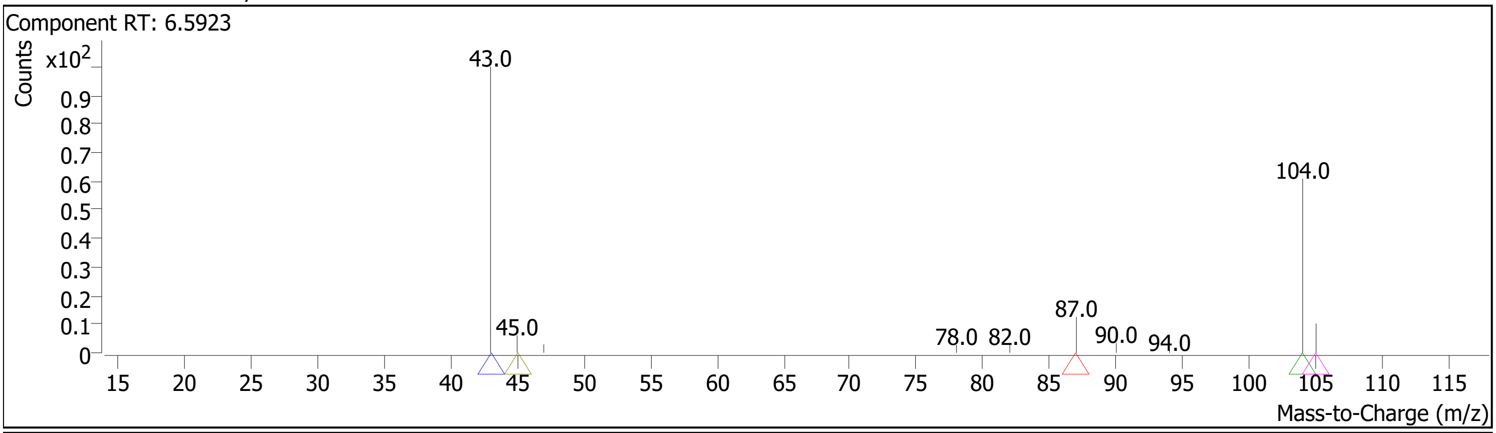

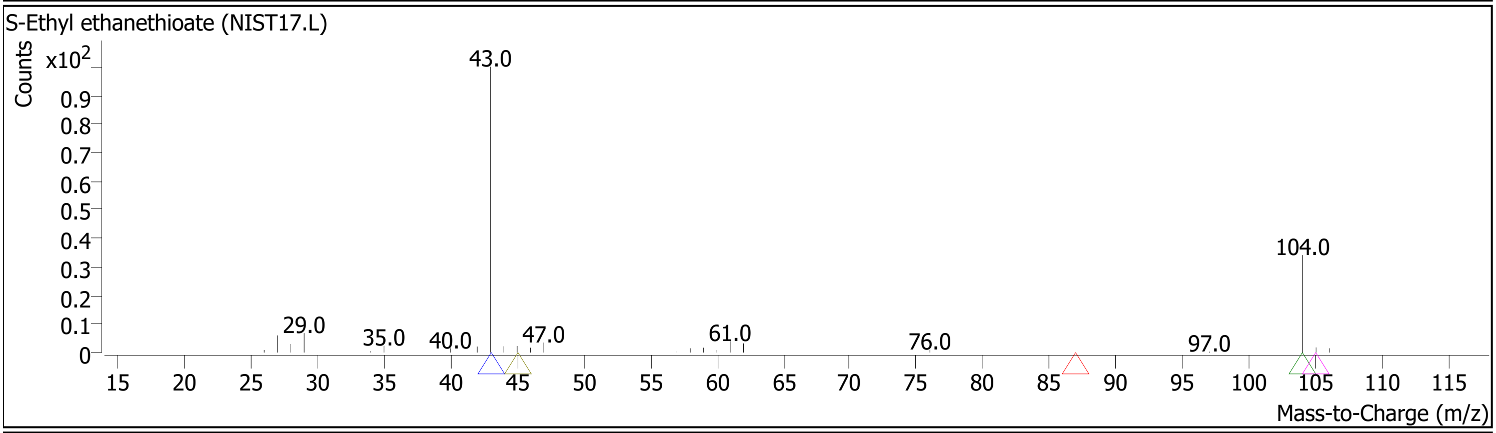

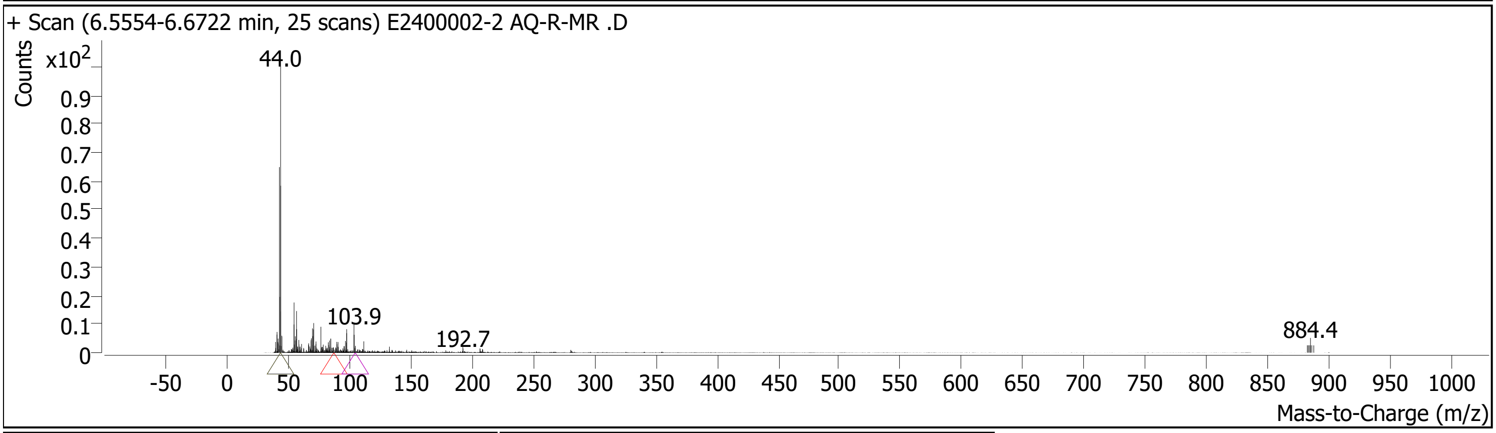

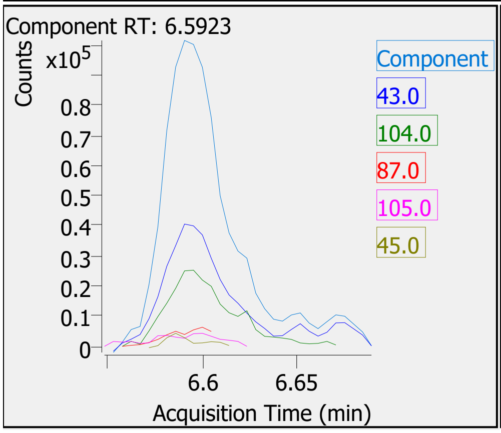

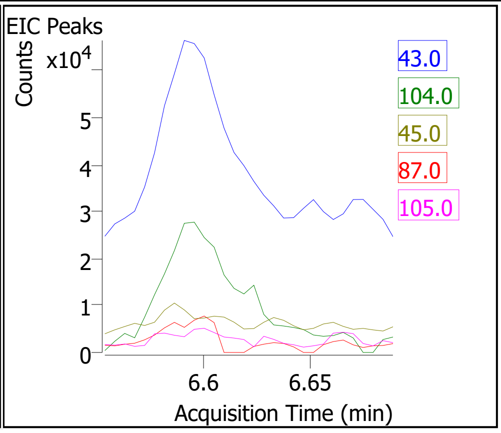

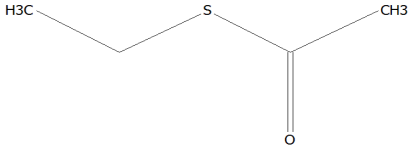

 Generated at 09:38 AM on 04-04-2024

Library Search Results - NonTarget Hits with Details

Component RT	Compound Name	Component Area	Match Factor	CAS#	Formula	Estimated Conc.
7.3506	.beta.-Hydroxyethyltheophylline tert-butyldimethylsilyl ether	177937.3	63.3	1000442-93-8	C15H26N4O3Si	

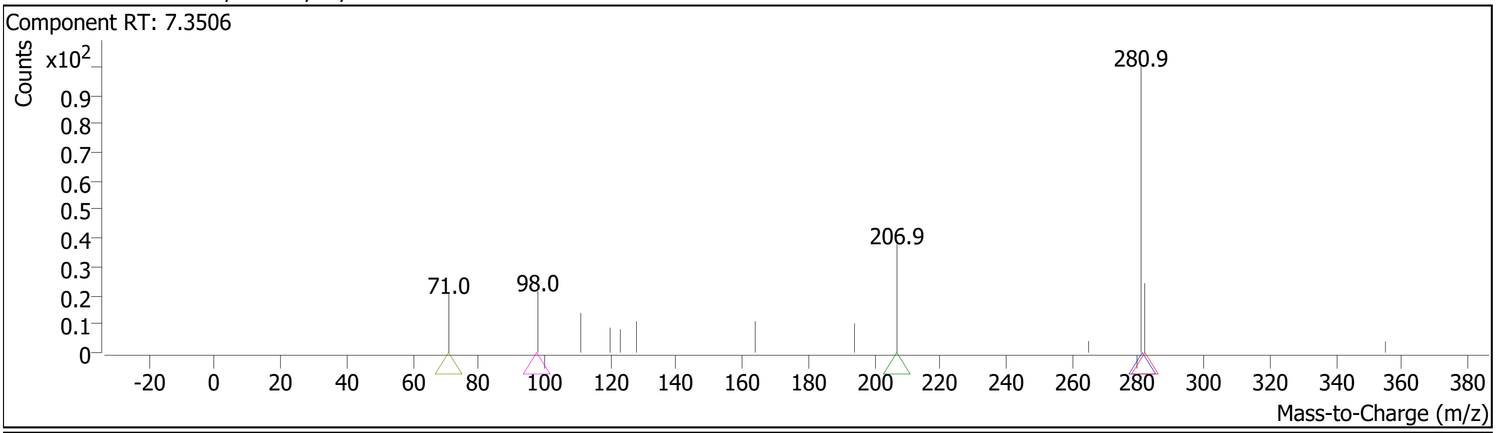

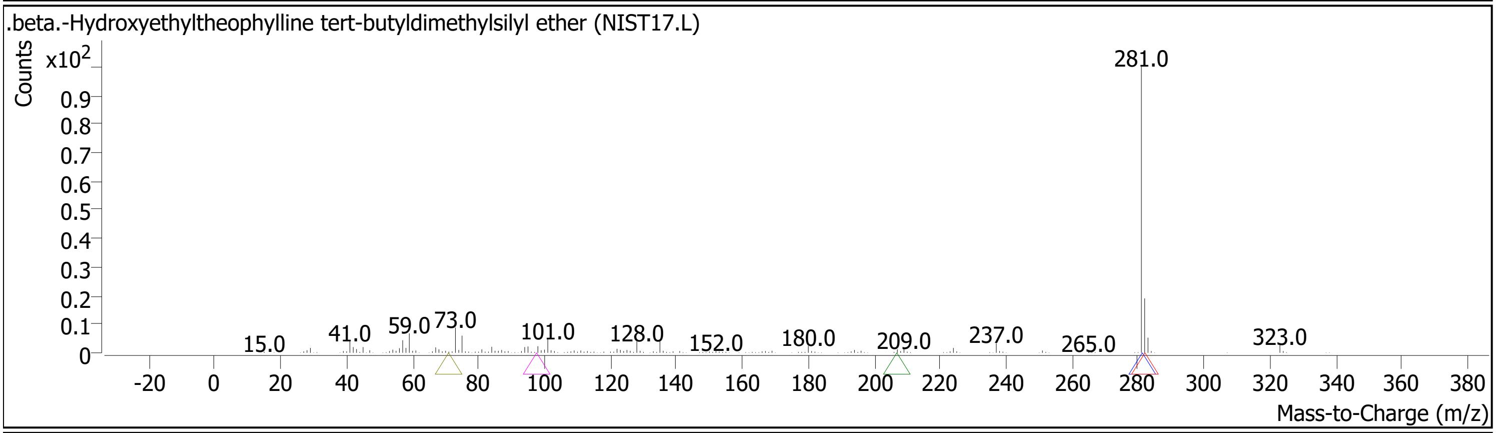

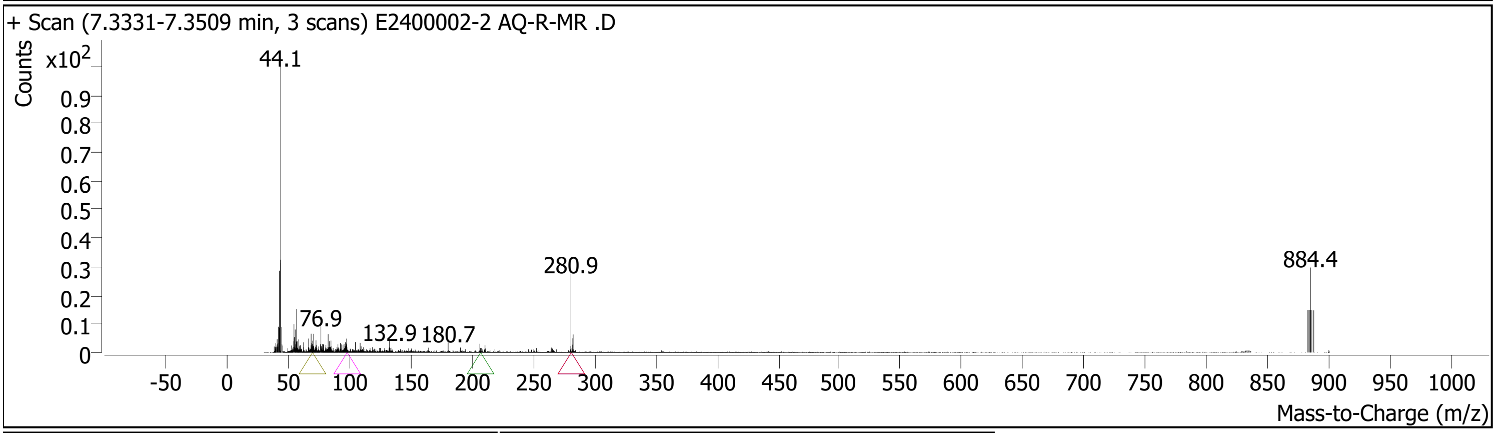

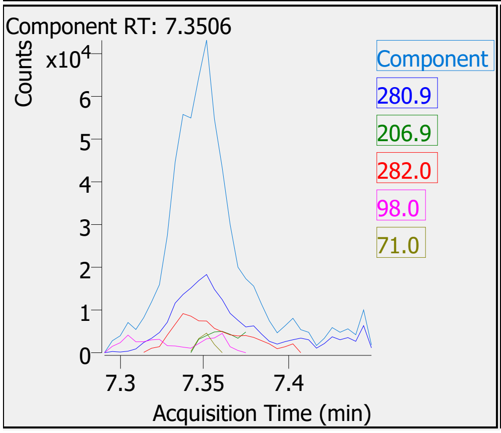

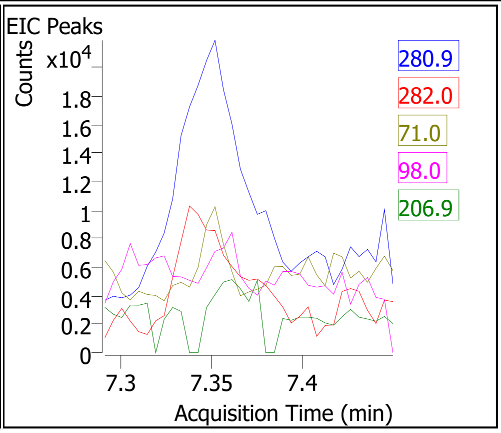

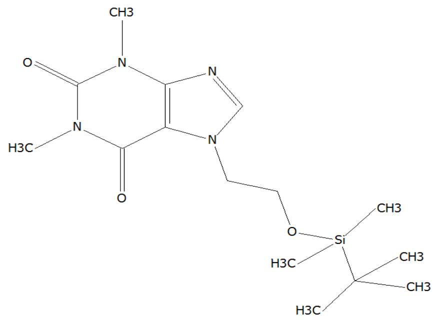

Generated at 09:38 AM on 04-04-2024

Library Search Results - NonTarget Hits with Details

Component RT	Compound Name	Component Area	Match Factor	CAS#	Formula	Estimated Conc.
13.5277	2-Oxo-4-phenyl-6-(4-chlorophenyl)-1,2-dihydropyrimidine	152759.8	74.5	24030-13-5	C16H11ClN2O	

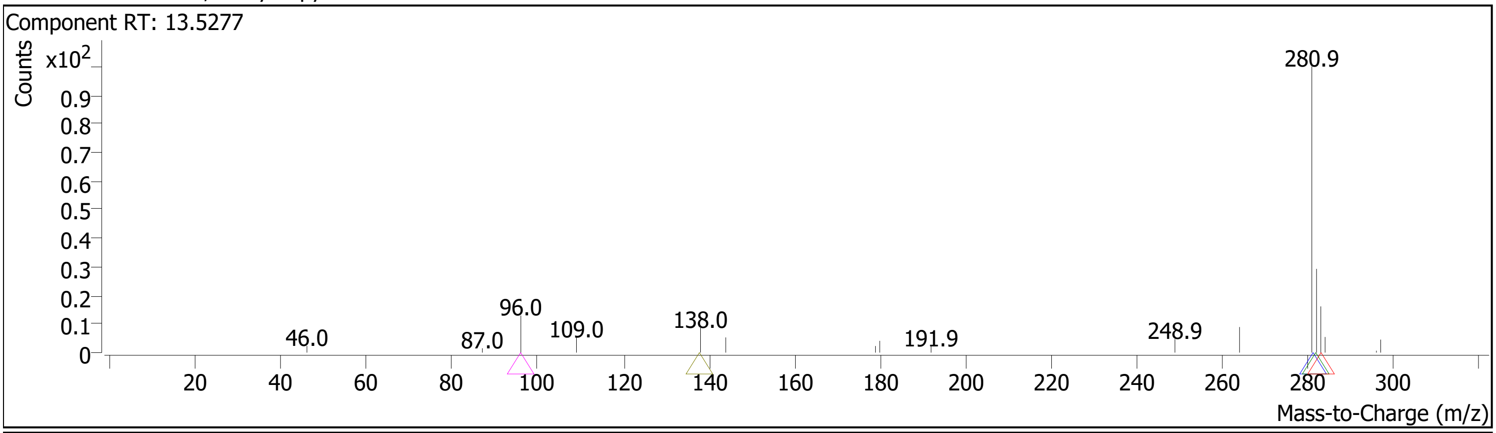

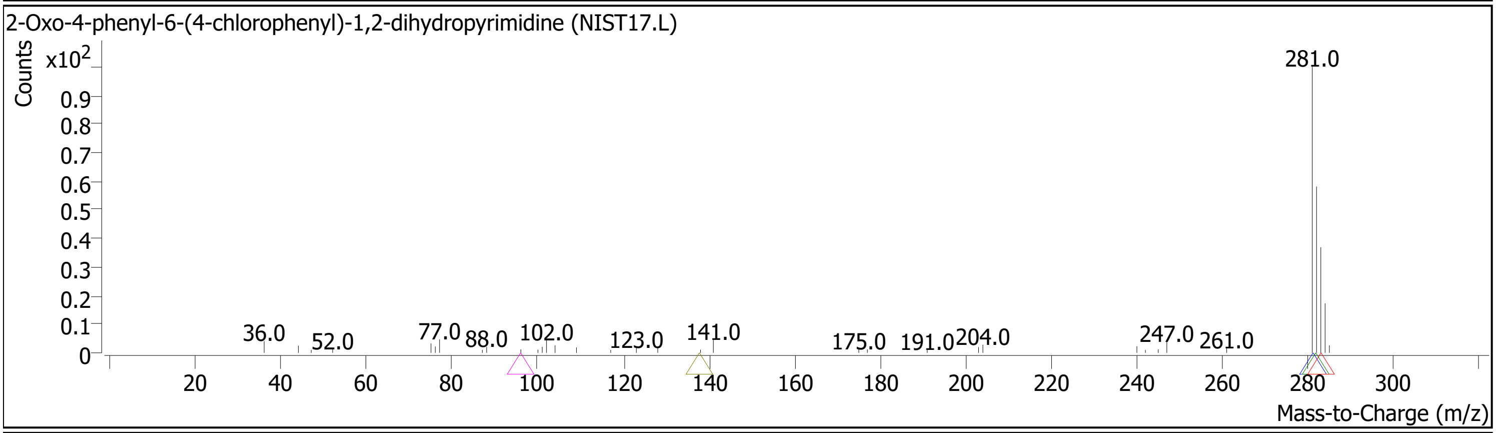

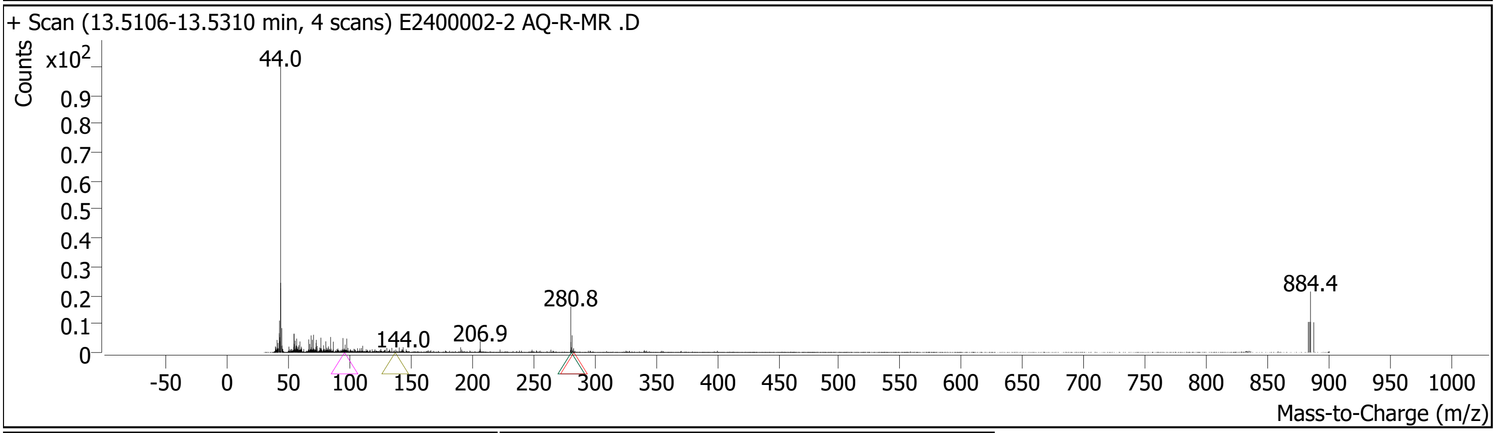

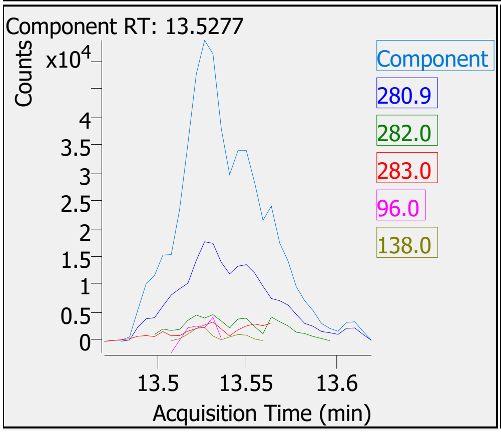

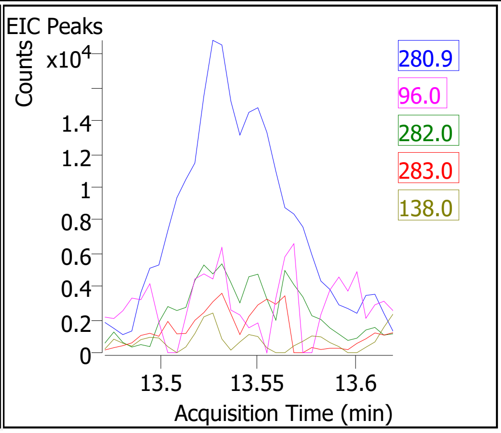

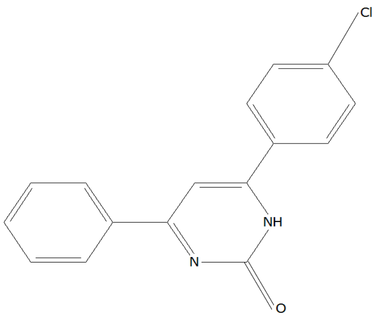

Component RT	Compound Name	Component Area	Match Factor	CAS#	Formula	Estimated Conc.
14.0354	2-Tetrazene, 1,1,4,4-tetramethyl-	7393.4	61.4	6130-87-6	C4H12N4	

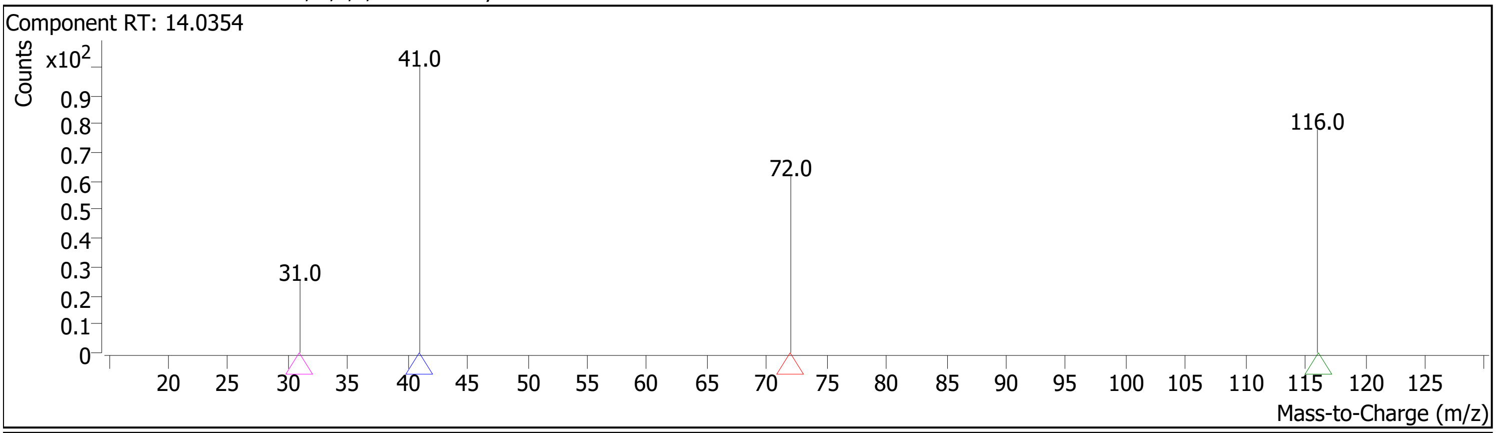

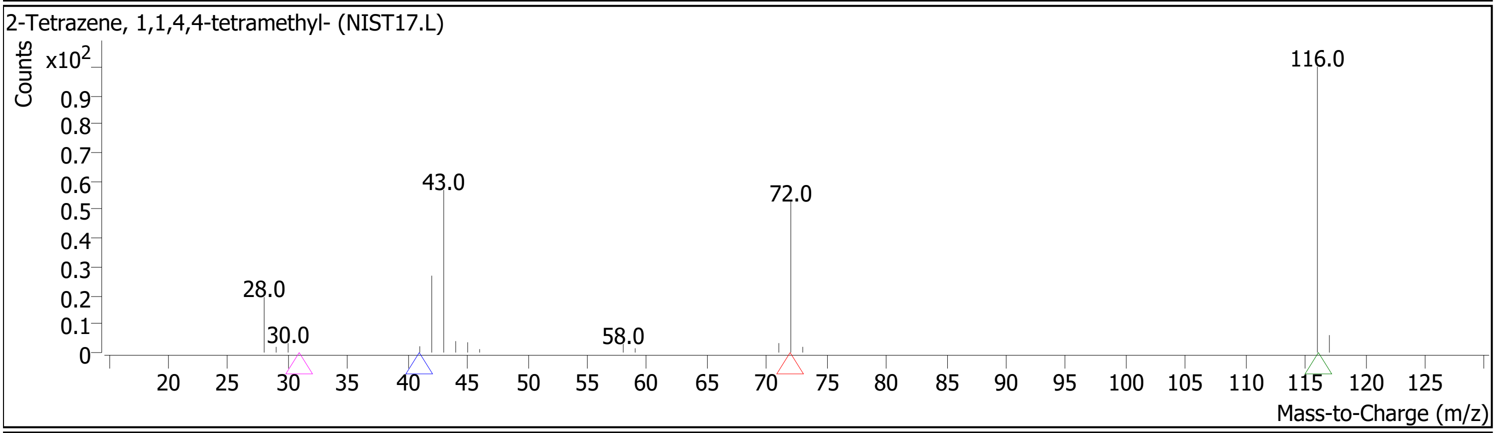

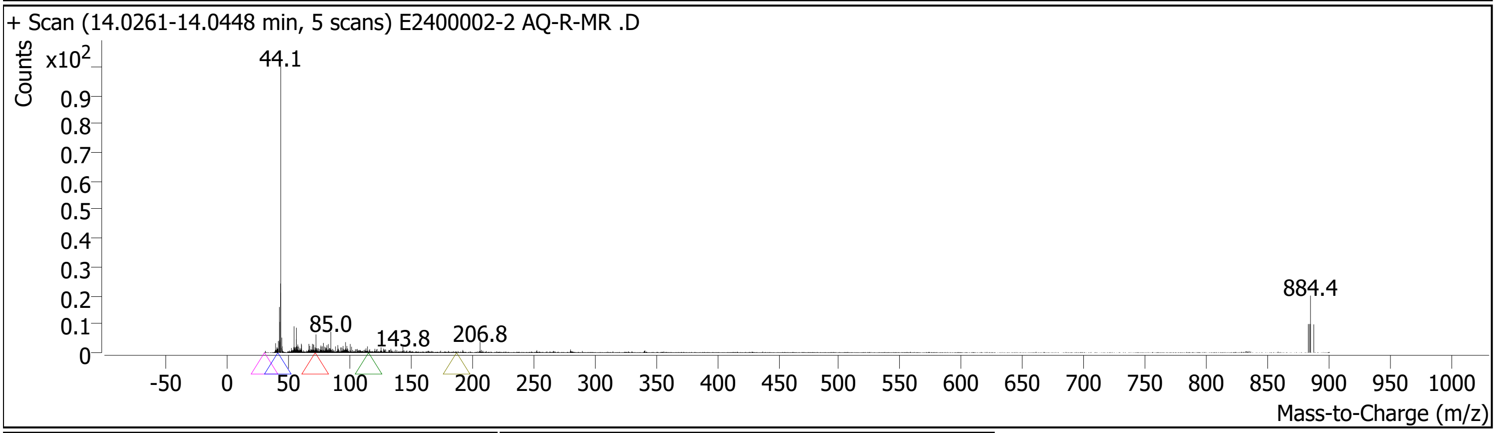

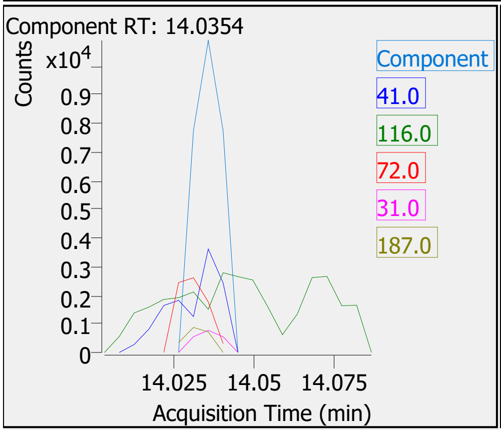

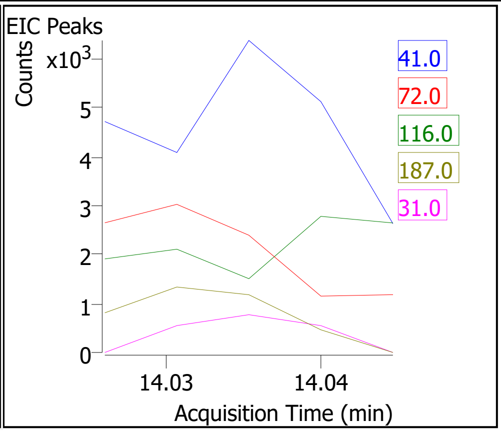

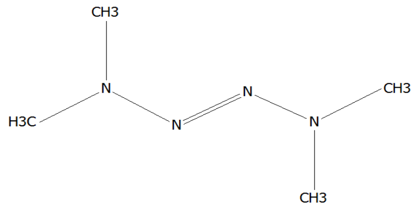

 Generated at 09:38 AM on 04-04-2024

Component RT	Compound Name	Component Area	Match Factor	CAS#	Formula	Estimated Conc.
16.1721	Propanenitrile, 3-(2-methoxy-1-methylethoxy)-	23889.3	63.3	35633-52-4	C7H13NO2	

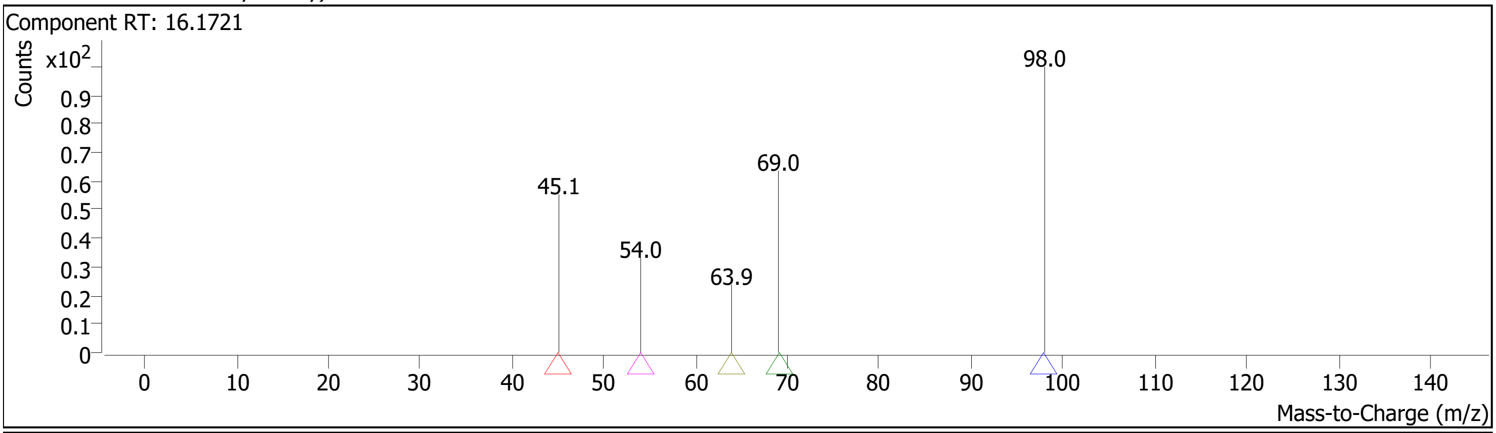

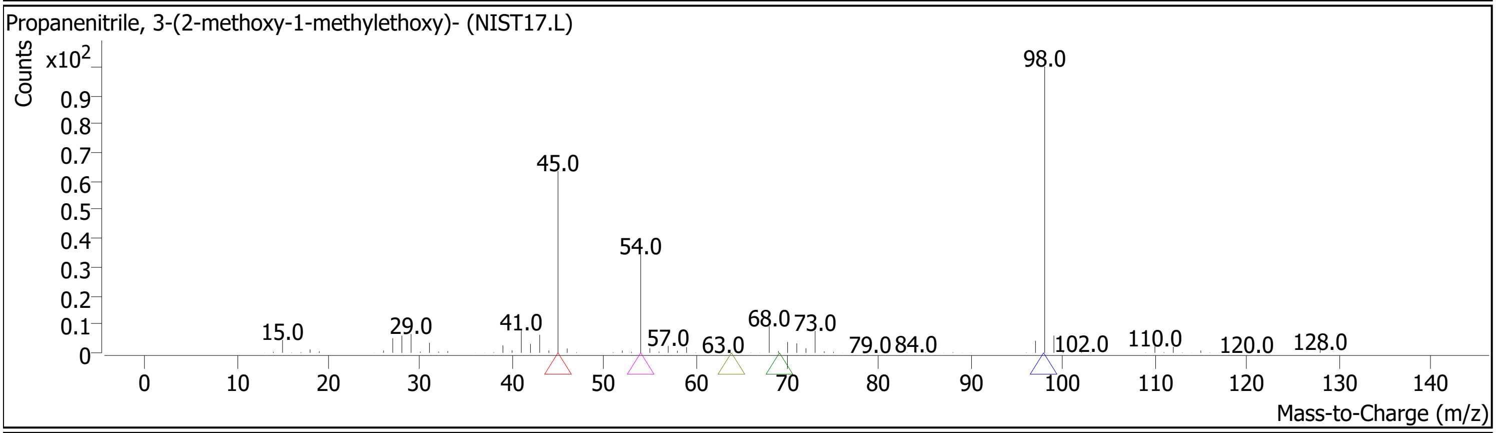

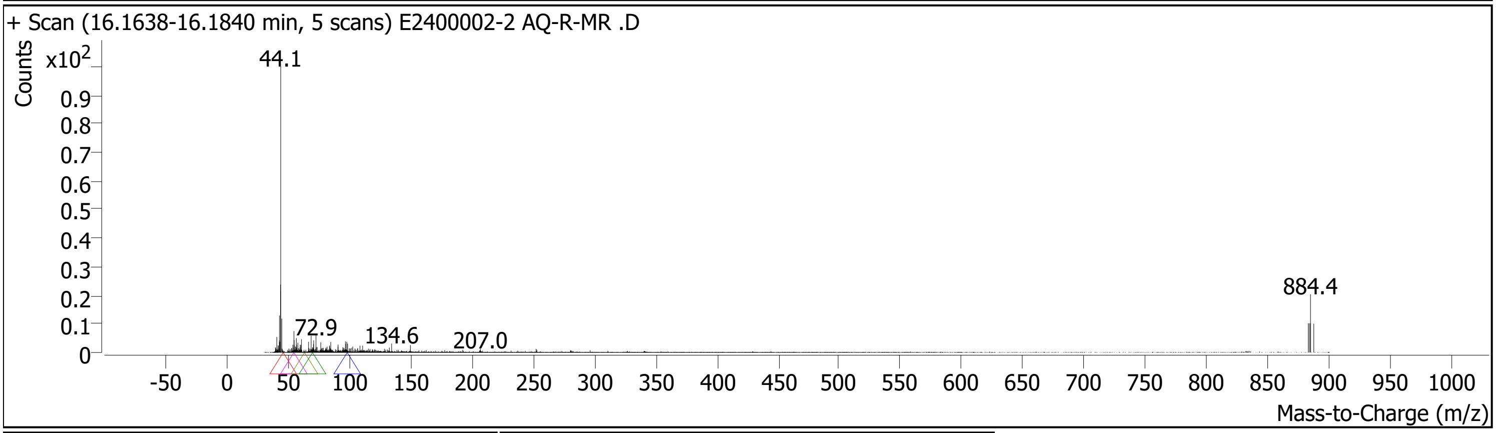

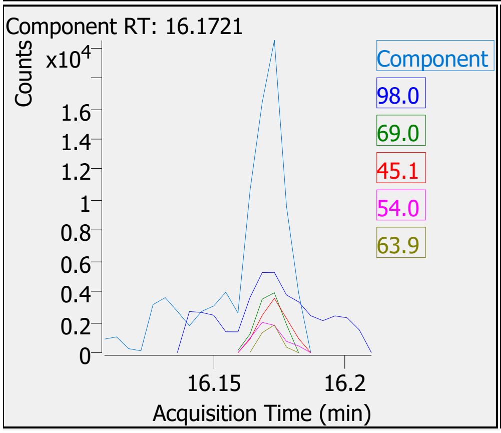

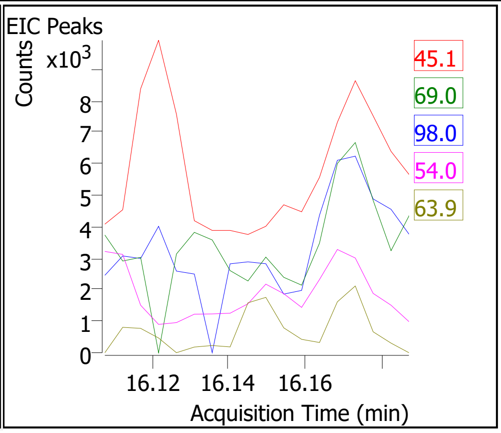

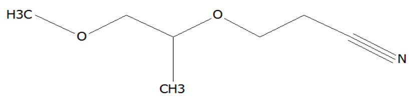

Library Search Results - NonTarget Hits with Details

Component RT	Compound Name	Component Area	Match Factor	CAS#	Formula	Estimated Conc.
21.1806	6-Methoxythymyl isobutyrate	1899277.0	68.1	1000413-80-3	C15H22O3	

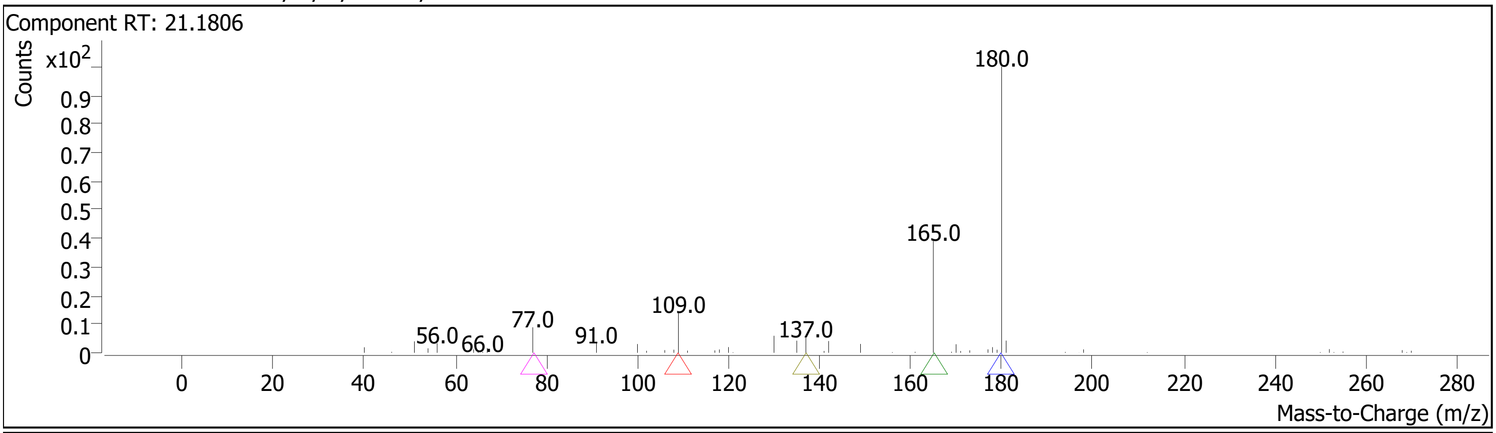

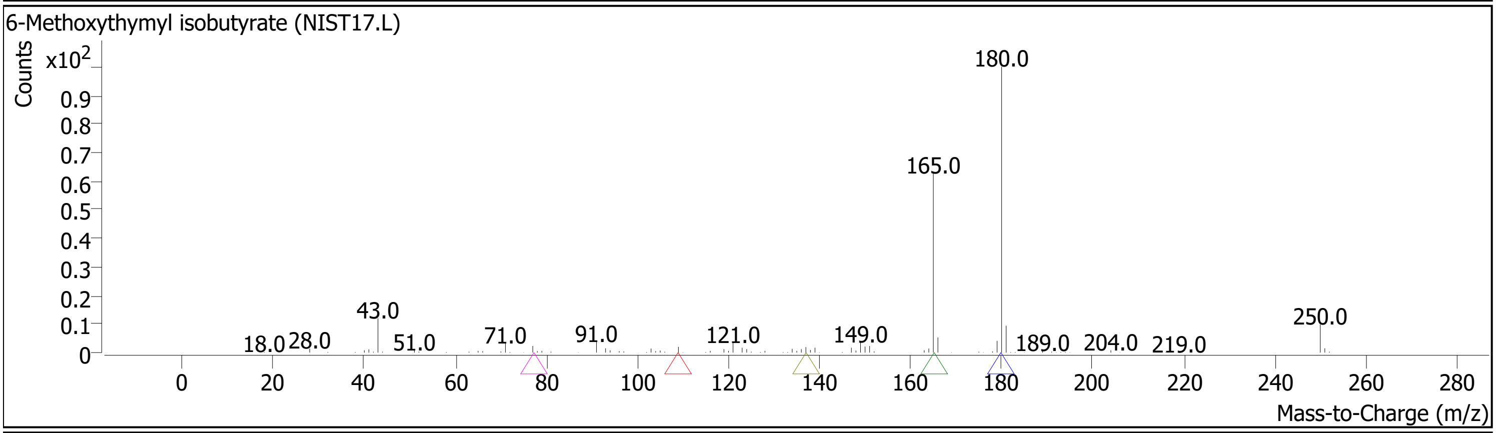

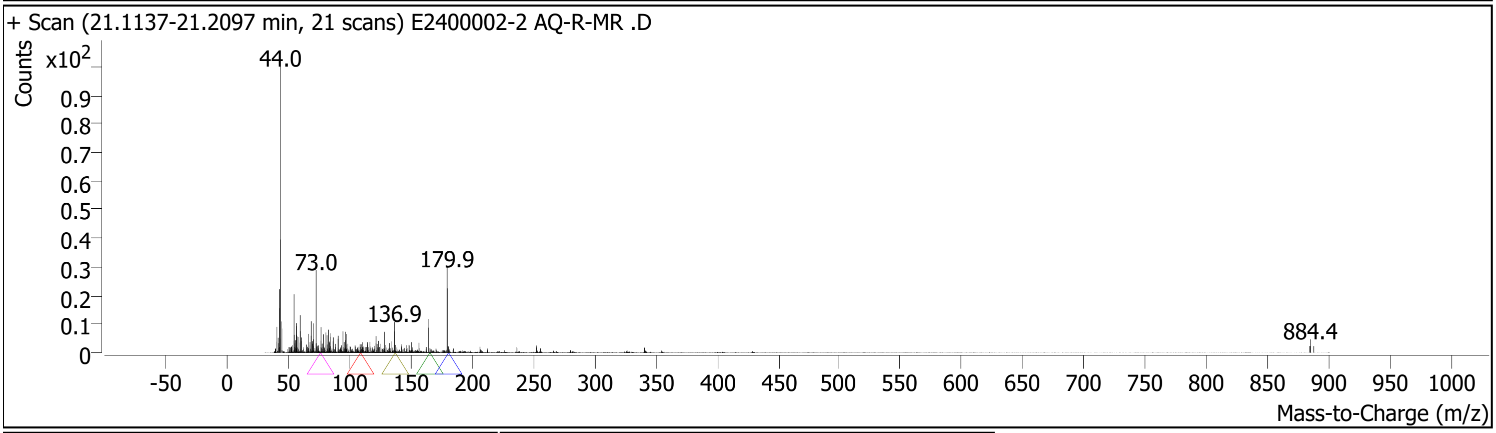

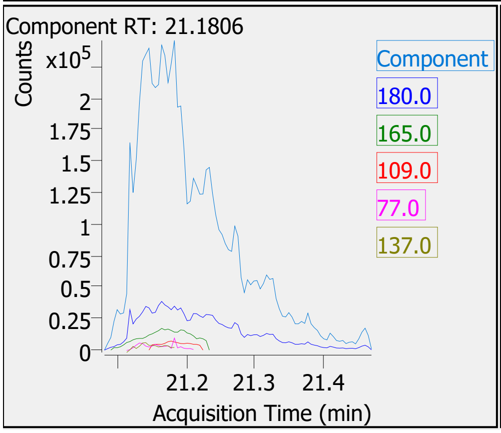

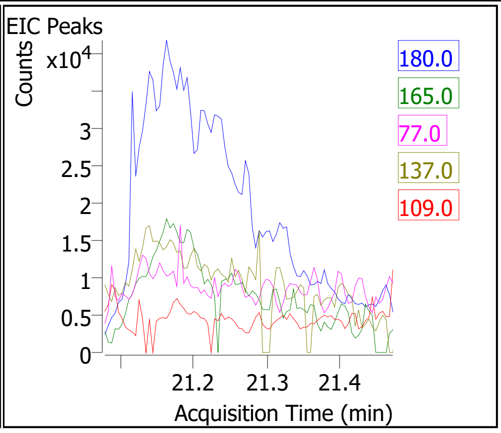

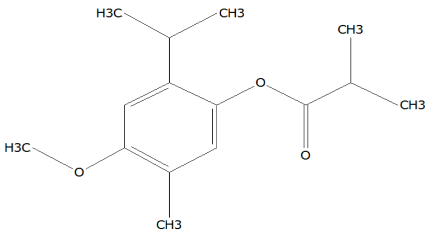

Agilent Trusted Answers

Component RT	Compound Name	Component Area	Match Factor	CAS#	Formula	Estimated Conc.
21.1815	Phenol, 4-ethenyl-2,6-dimethoxy-	2648244.3	64.4	28343-22-8	C10H12O3	

Component RT: 21.1815

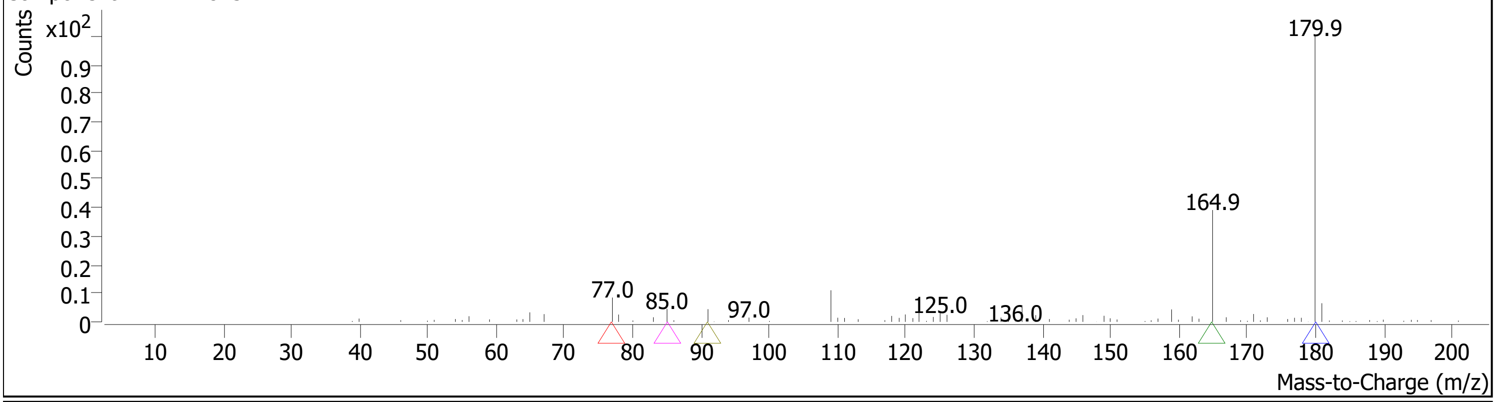

Phenol, 4-ethenyl-2,6-dimethoxy- (NIST17.L)

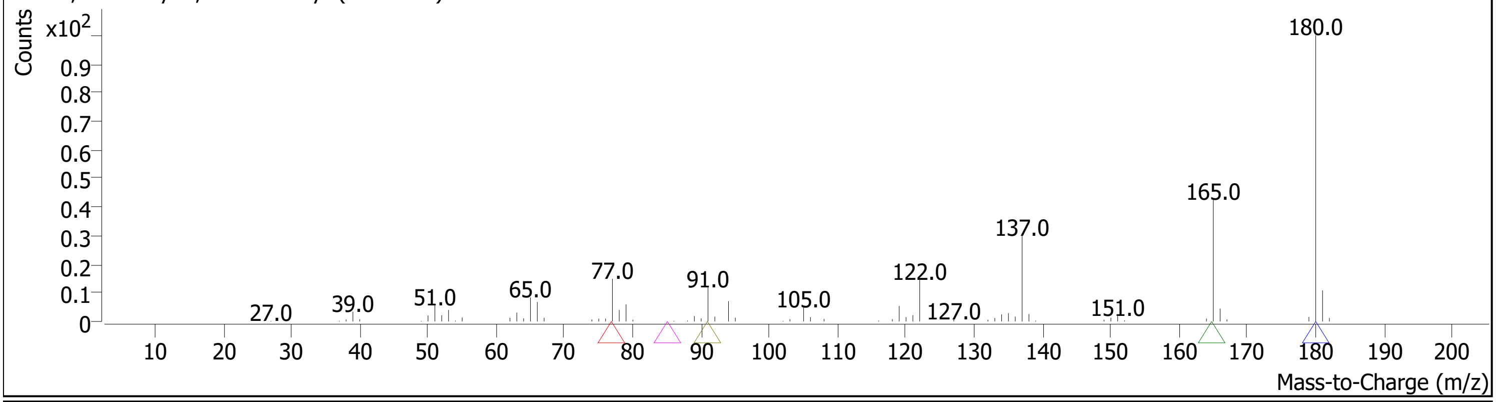

+ Scan (21.2144-21.2330 min, 5 scans) E2400002-2 AQ-R-MR .D

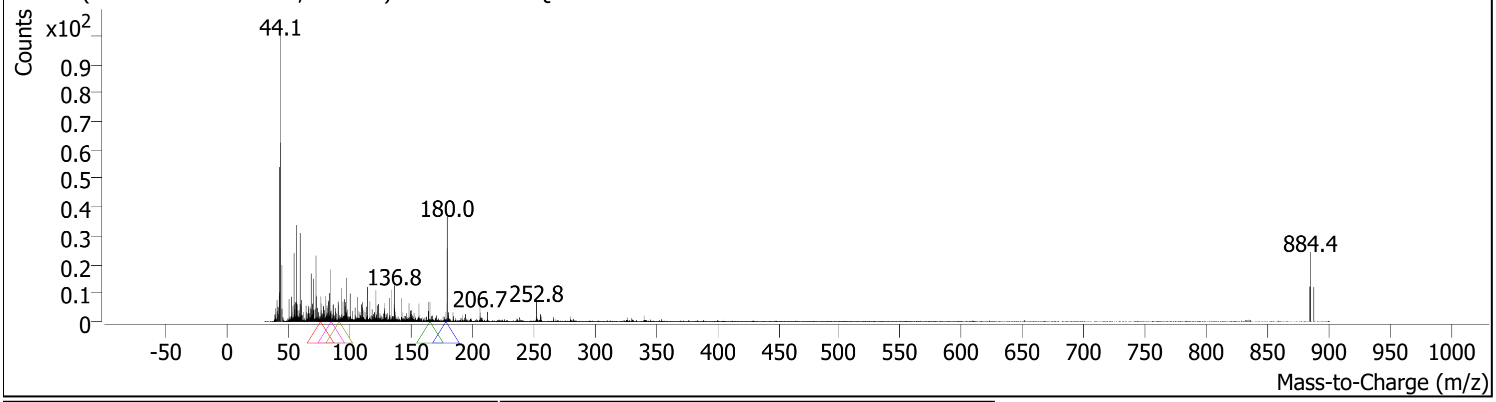

Component RT: 21.1815

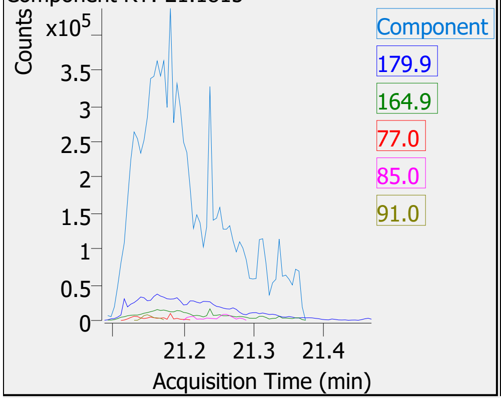

EIC Peaks

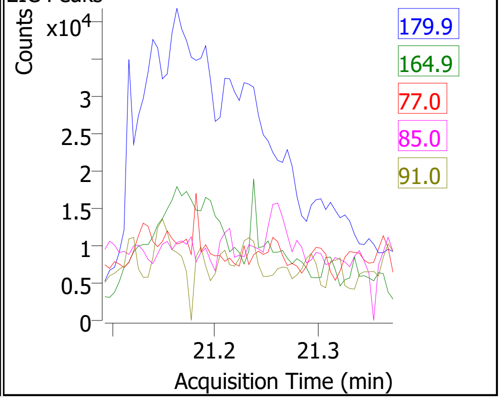

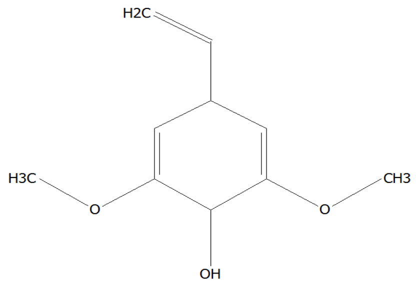

Library Search Results - NonTarget Hits with Details

Component RT	Compound Name	Component Area	Match Factor	CAS#	Formula	Estimated Conc.
22.7730	1,3-Dioxolane, 2-methyl-2-(4-methyl-3-methylenepentyl)-	2945986.9	69.3	66972-05-2	C11H20O2	

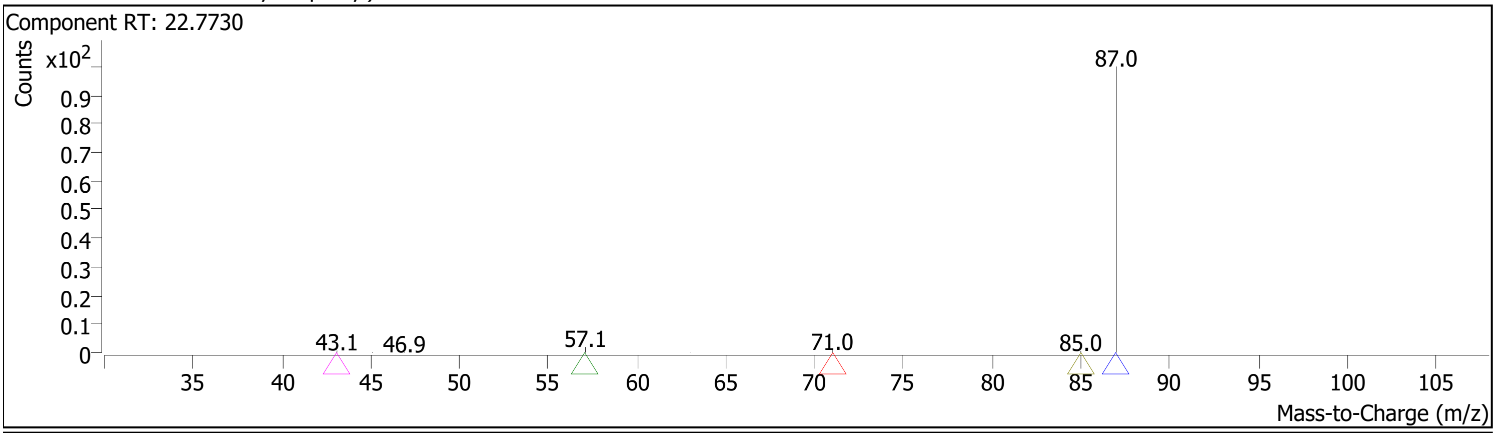

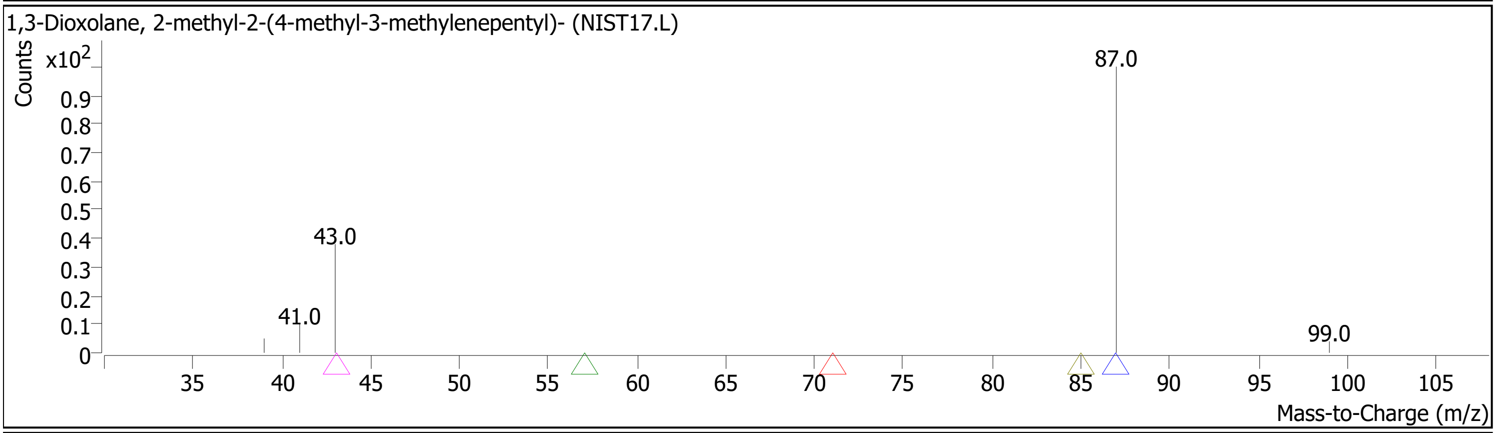

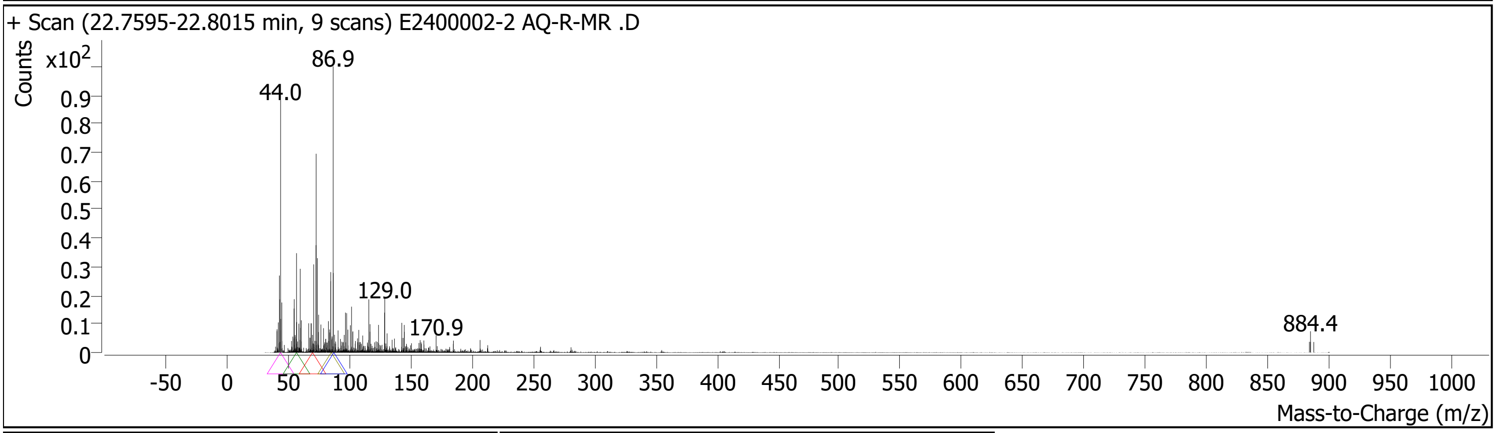

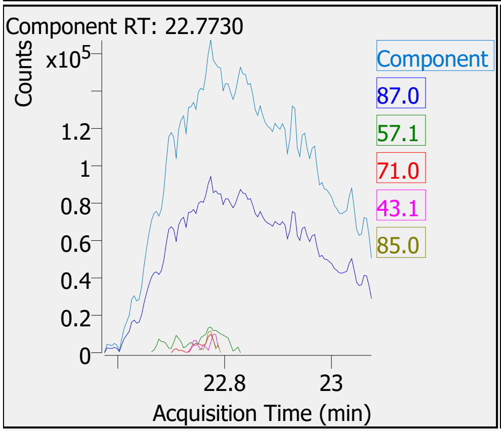

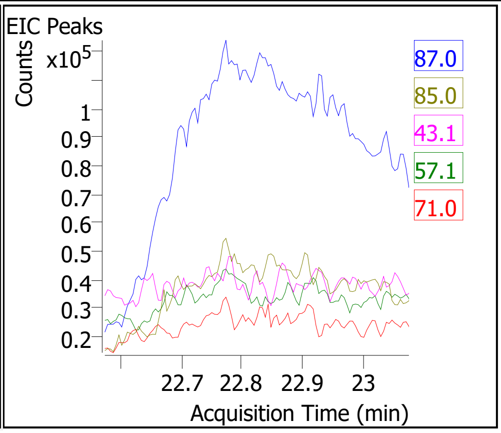

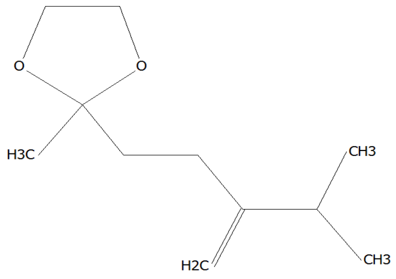

Component RT	Compound Name	Component Area	Match Factor	CAS#	Formula	Estimated Conc.
24.0346	Undec-10-ynoic acid, tridec-2-yn-1-yl ester	1354911.9	69.2	1000406-96-9	C24H40O2	

Component RT: 24.0346

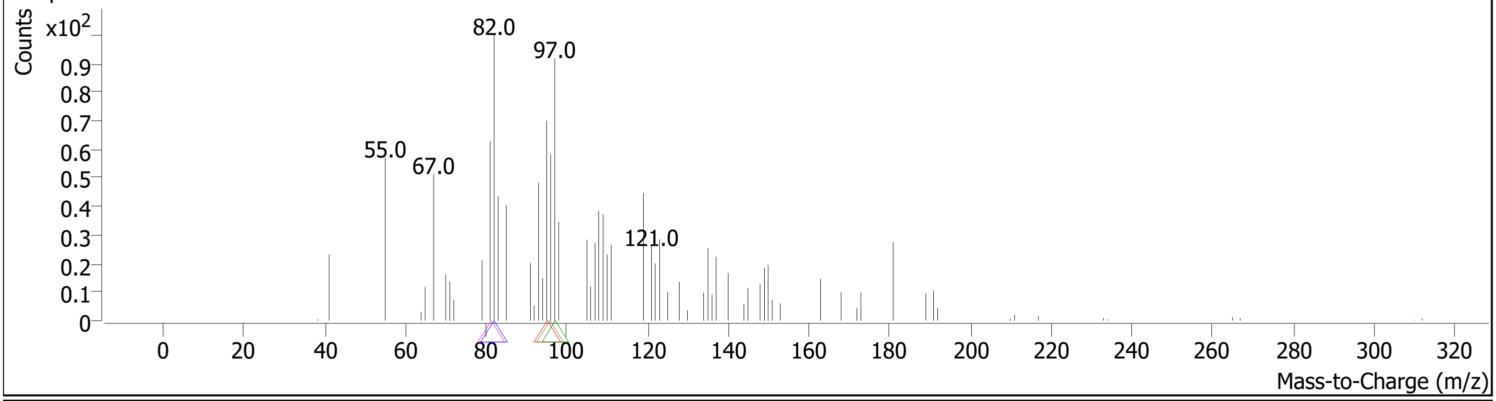

Undec-10-ynoic acid, tridec-2-yn-1-yl ester (NIST17.L)

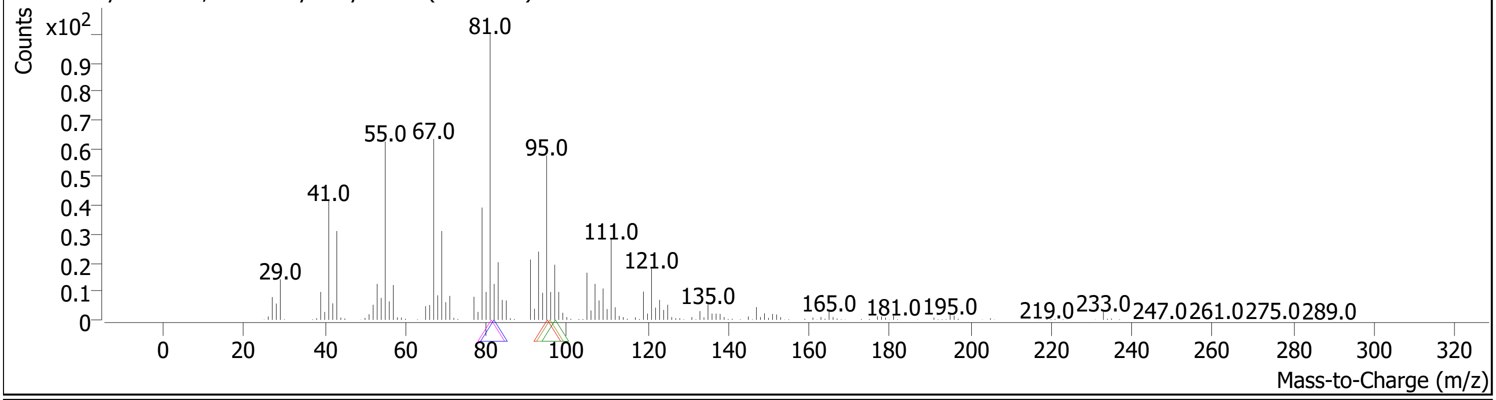

+ Scan (24.0013-24.0573 min, 13 scans) E2400002-2 AQ-R-MR .D

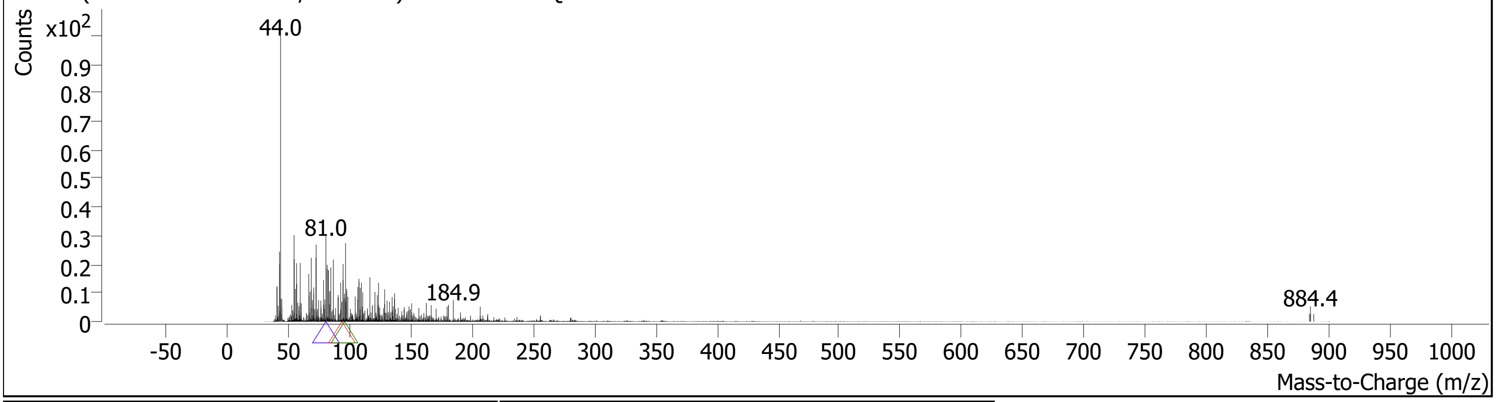

Component RT: 24.0346

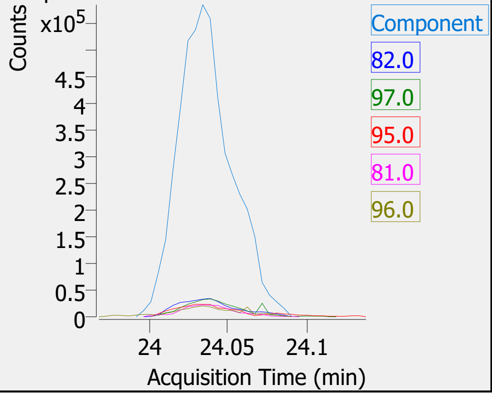

EIC Peaks

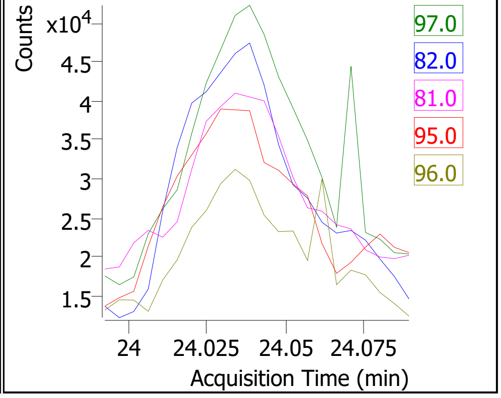

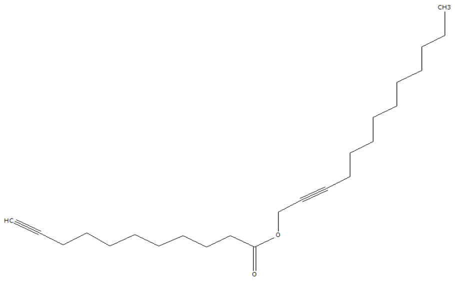

Library Search Results - NonTarget Hits with Details

Component RT	Compound Name	Component Area	Match Factor	CAS#	Formula	Estimated Conc.
25.2119	Isoaromadendrene epoxide	3670403.5	81.4	1000159-36-6	C15H24O	

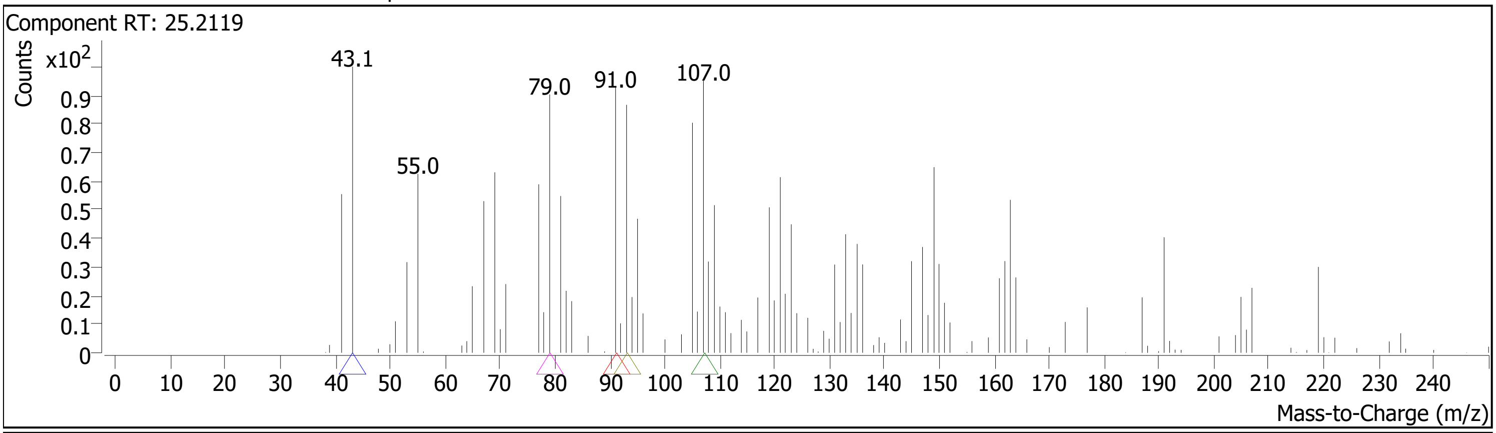

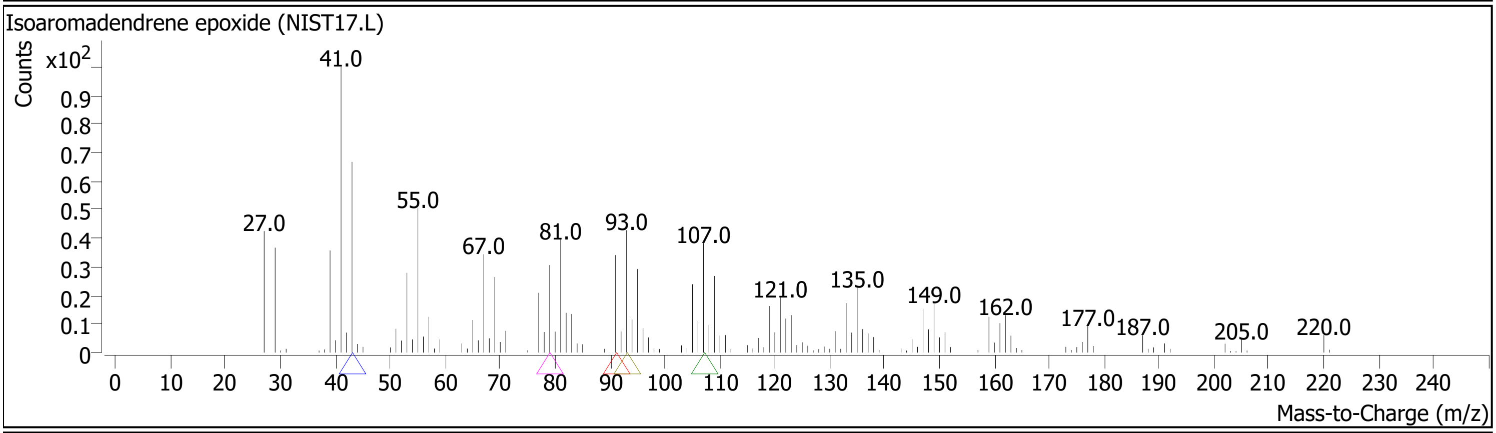

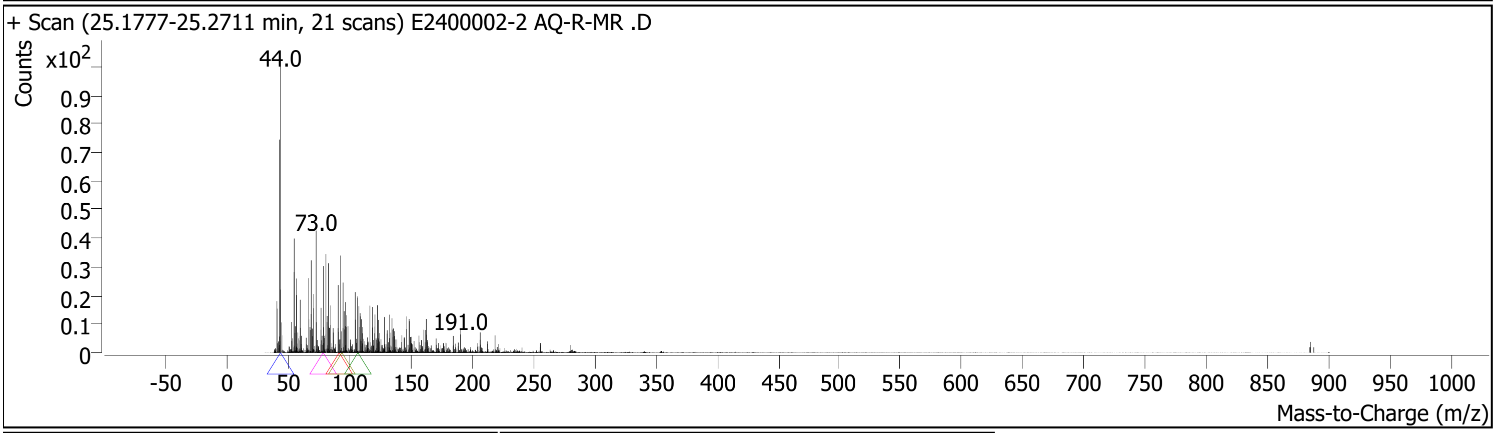

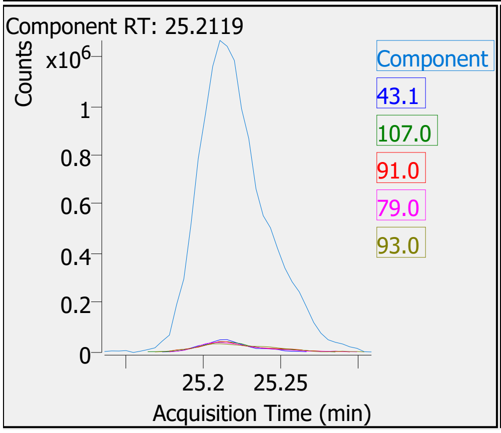

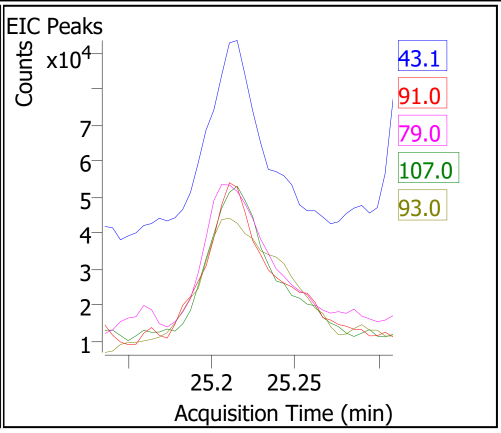

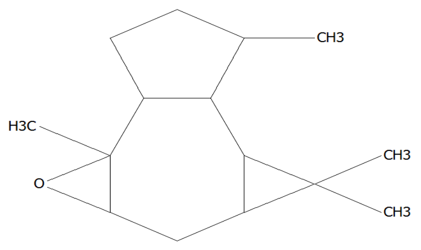

Library Search Results - NonTarget Hits with Details

Component RT	Compound Name	Component Area	Match Factor	CAS#	Formula	Estimated Conc.
25.3310	Hexadecanoic acid, methyl ester	6957469.9	94.8	112-39-0	C17H34O2	

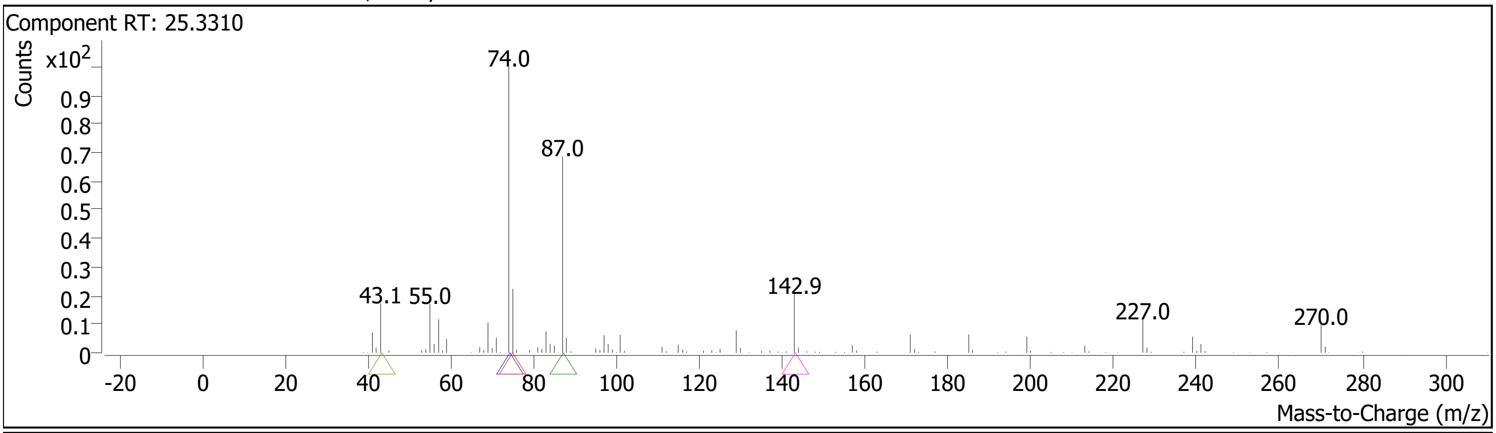

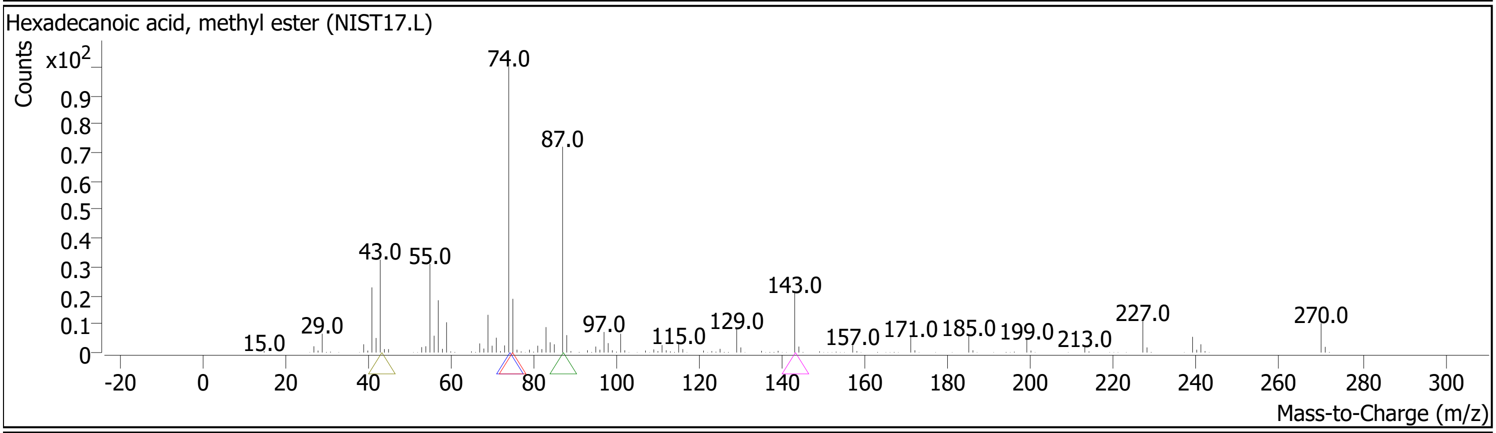

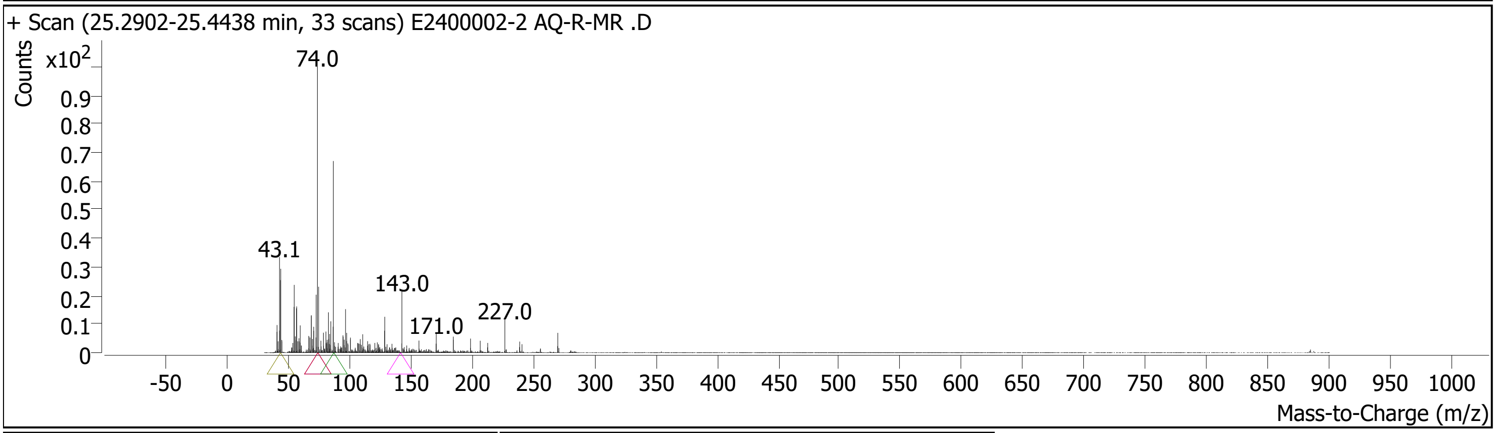

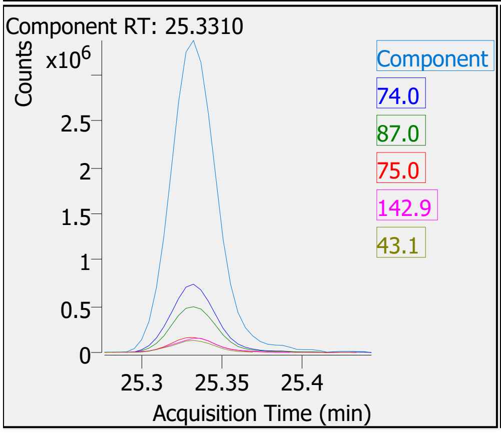

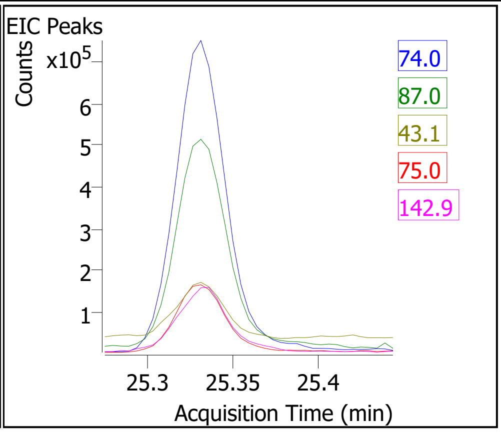

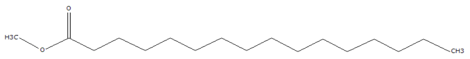

Library Search Results - NonTarget Hits with Details

Component RT	Compound Name	Component Area	Match Factor	CAS#	Formula	Estimated Conc.
25.5736	Thunbergol	5266348.1	77.9	25269-17-4	C20H34O	

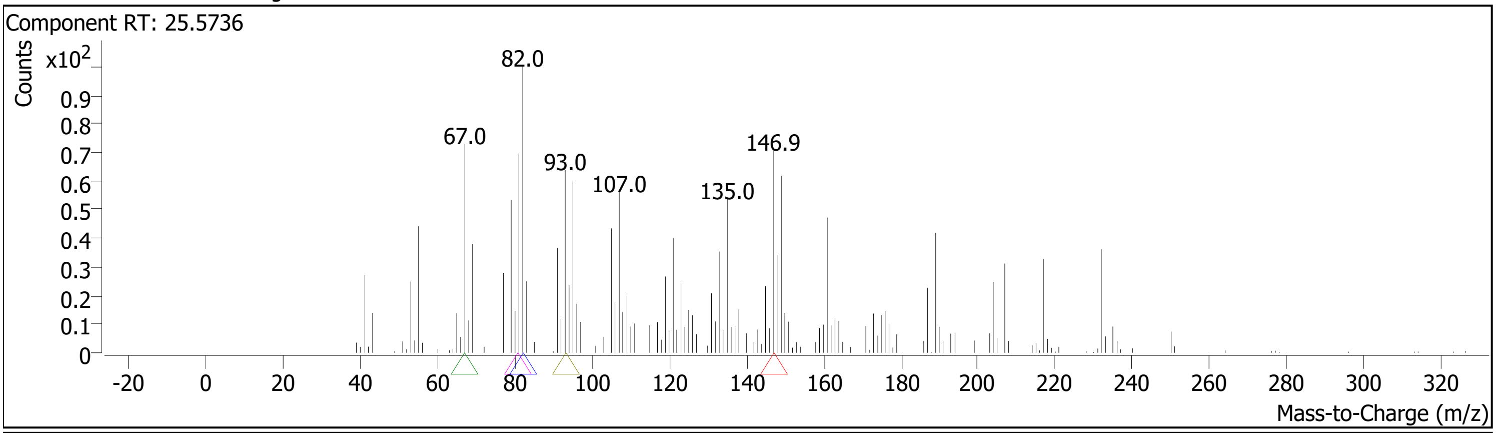

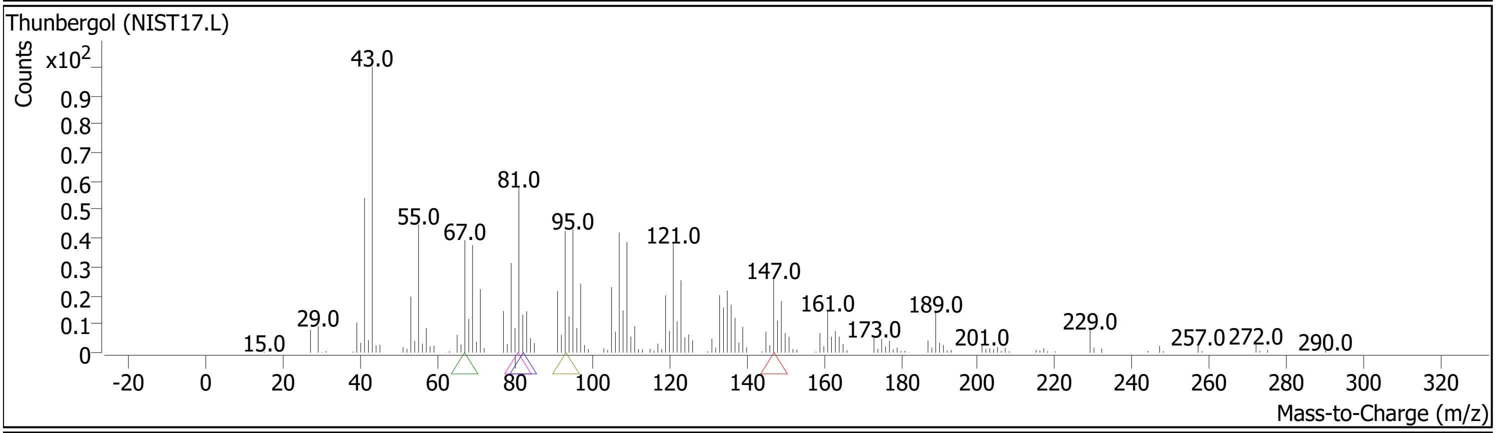

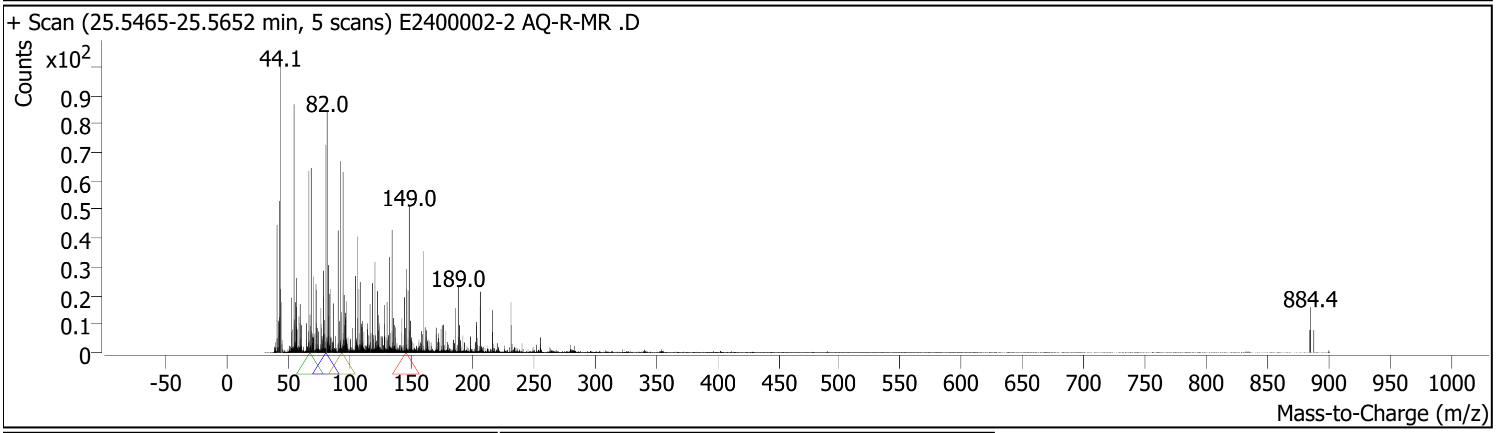

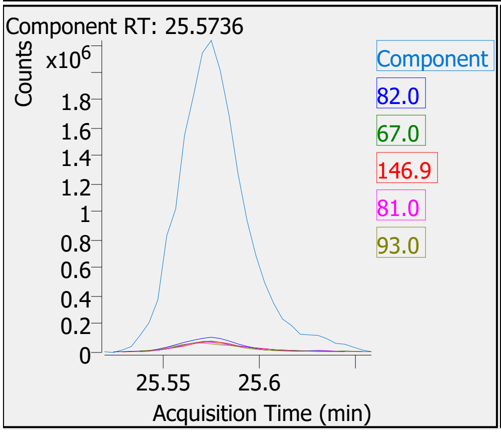

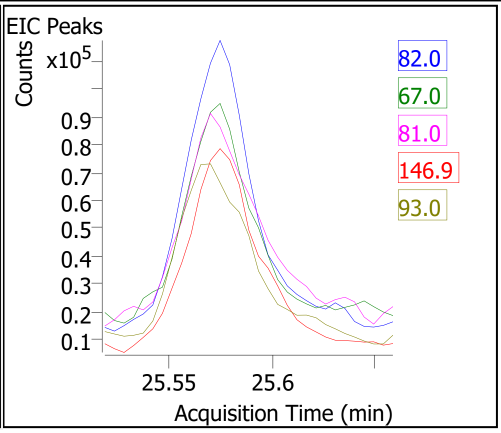

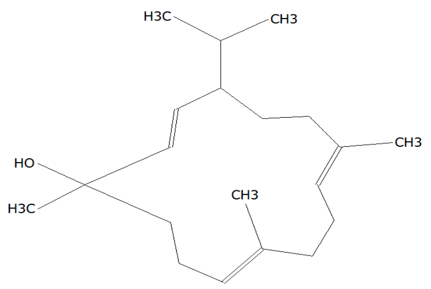

Generated at 09:38 AM on 04-04-2024

Library Search Results - NonTarget Hits with Details

Component RT	Compound Name	Component Area	Match Factor	CAS#	Formula	Estimated Conc.
27.6686	Methyl stearate	4599766.1	93.0	112-61-8	C19H38O2	

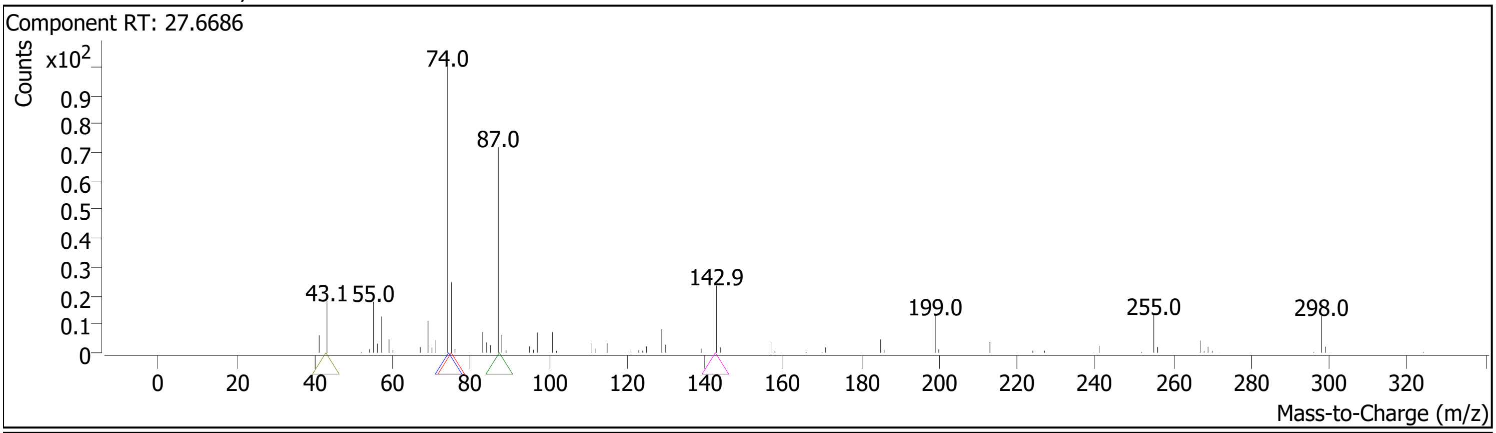

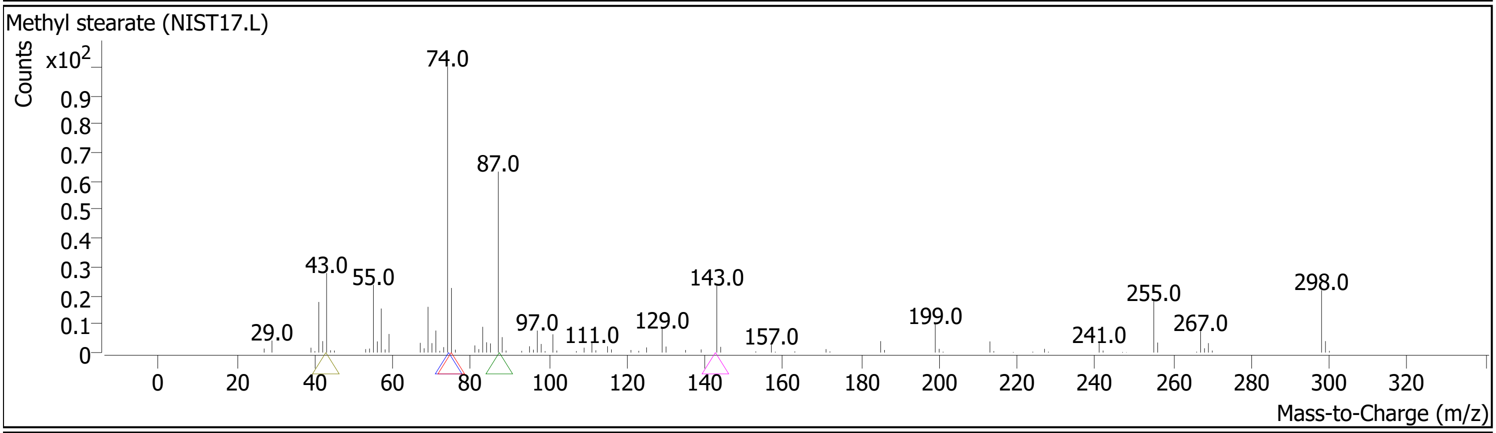

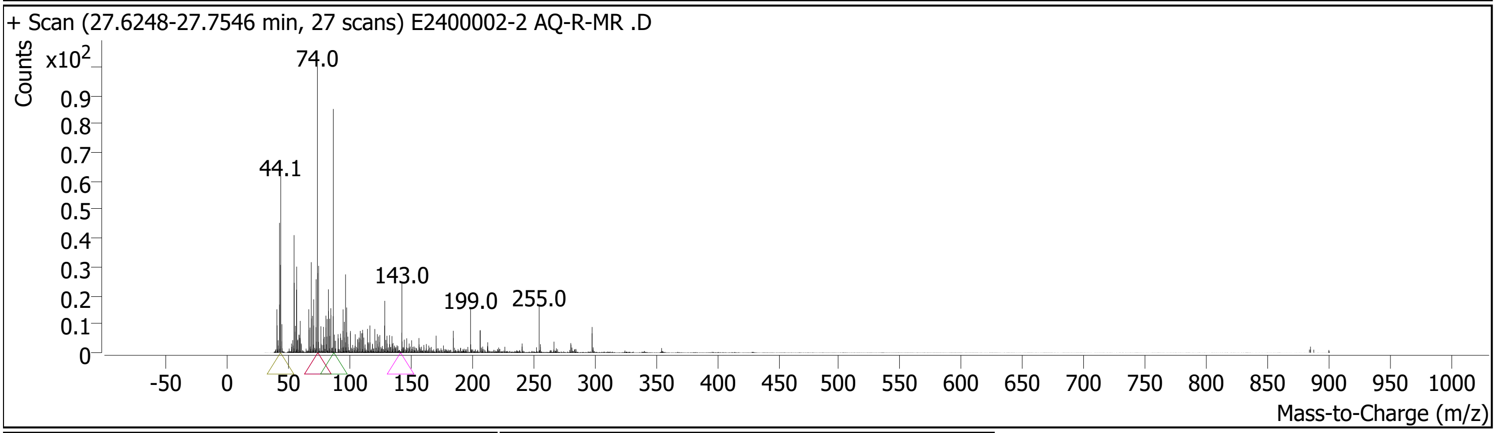

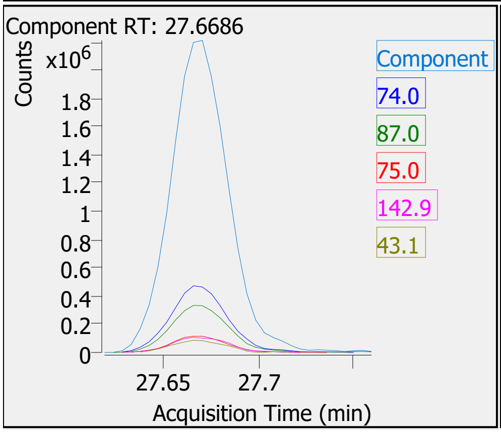

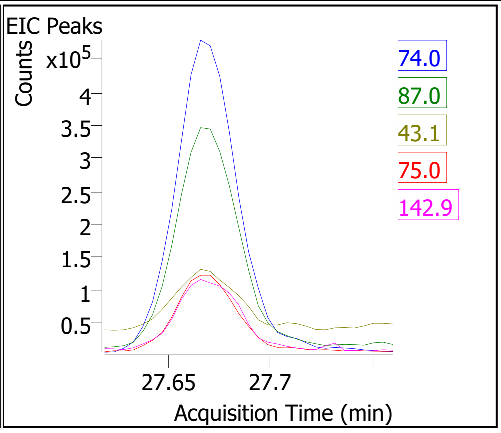

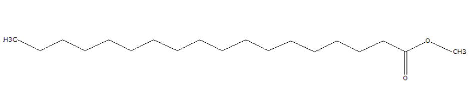

Library Search Results - NonTarget Hits with Details

Component RT	Compound Name	Component Area	Match Factor	CAS#	Formula	Estimated Conc.
31.2201	Decane, 3,8-dimethyl-	1734251.9	73.3	17312-55-9	C12H26	

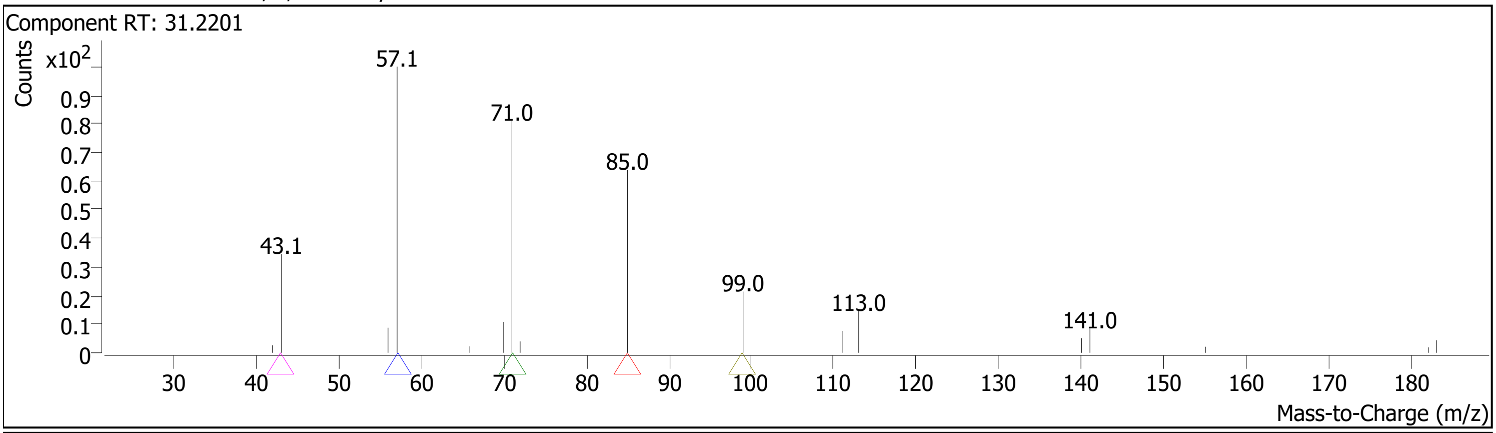

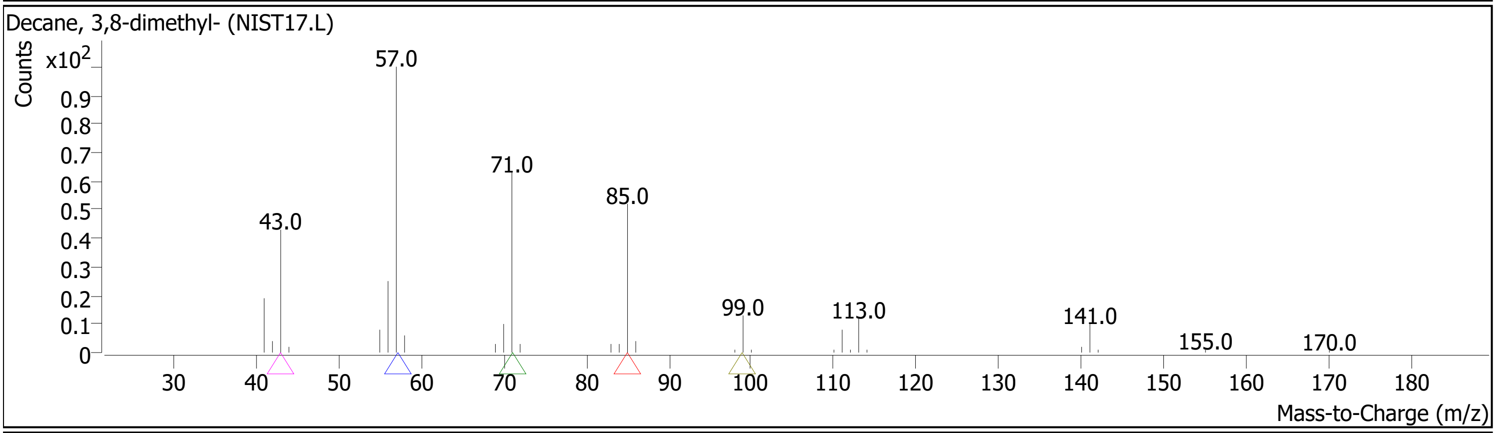

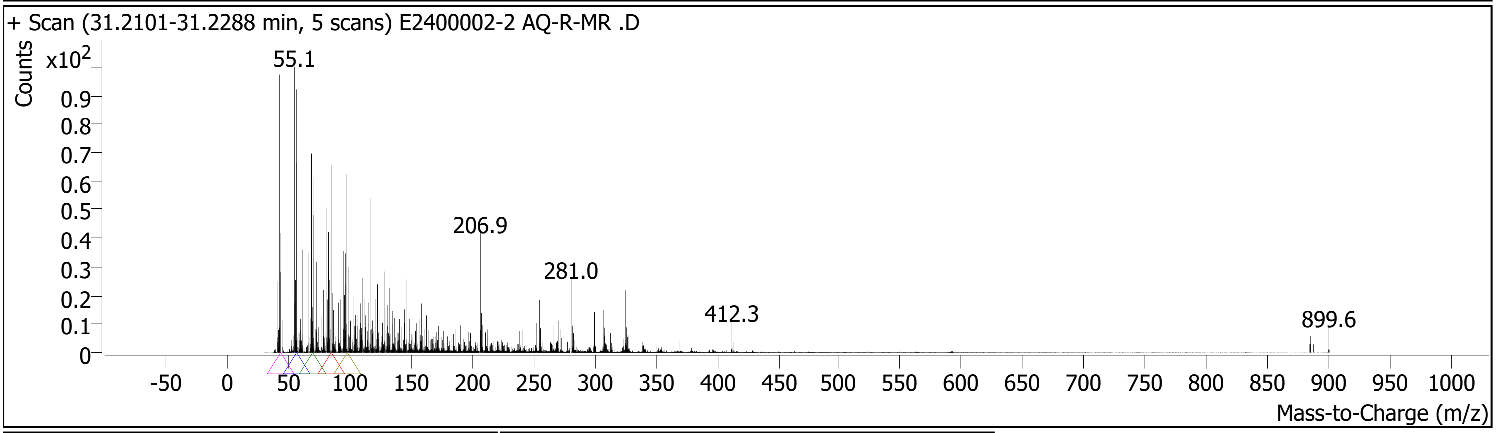

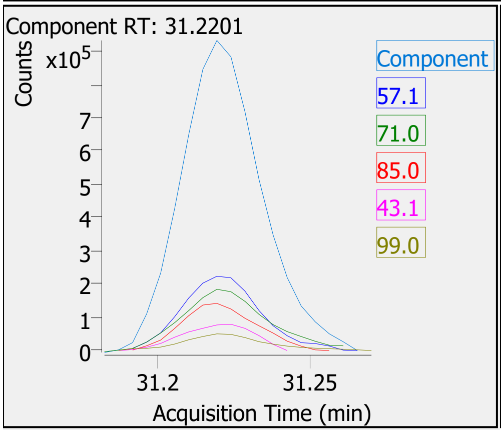

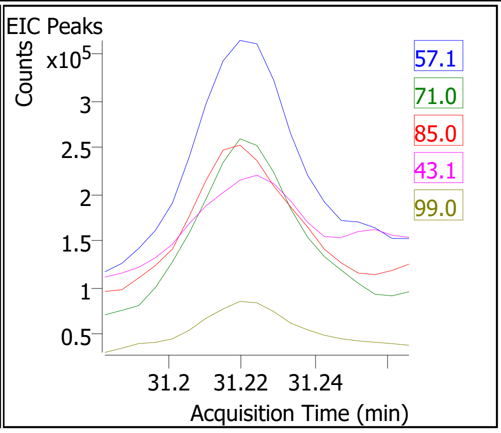

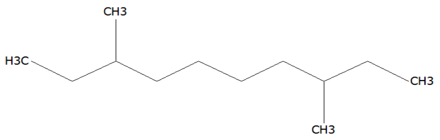

Library Search Results - NonTarget Hits with Details

Component RT	Compound Name	Component Area	Match Factor	CAS#	Formula	Estimated Conc.
31.3774	Hexadecanoic acid, 2-hydroxy-1-(hydroxymethyl)ethyl ester	17537393.6	81.4	23470-00-0	$C_{19}H_{38}O_4$	

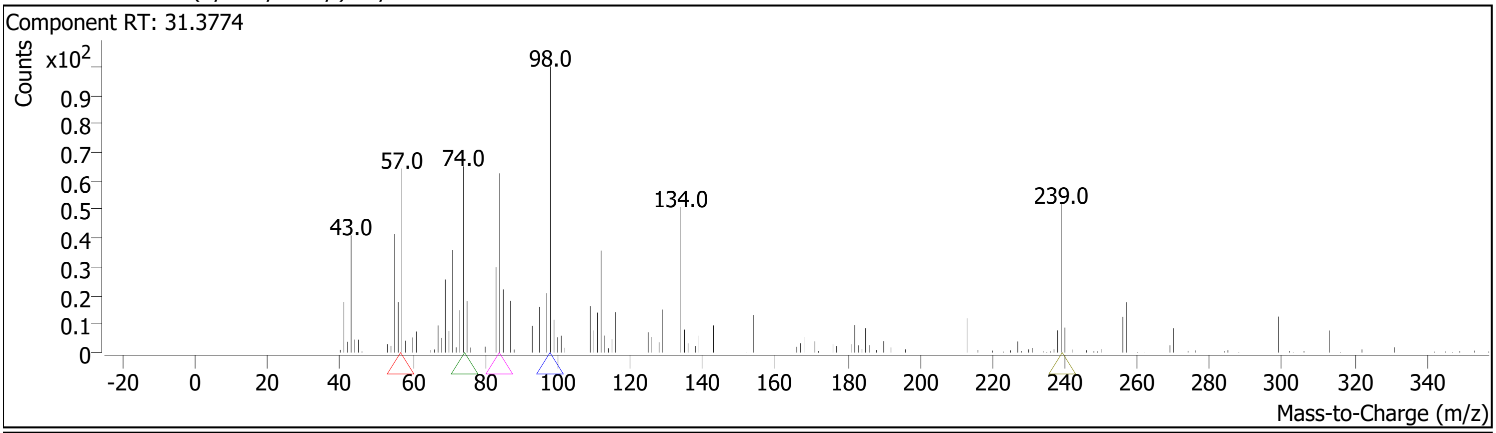

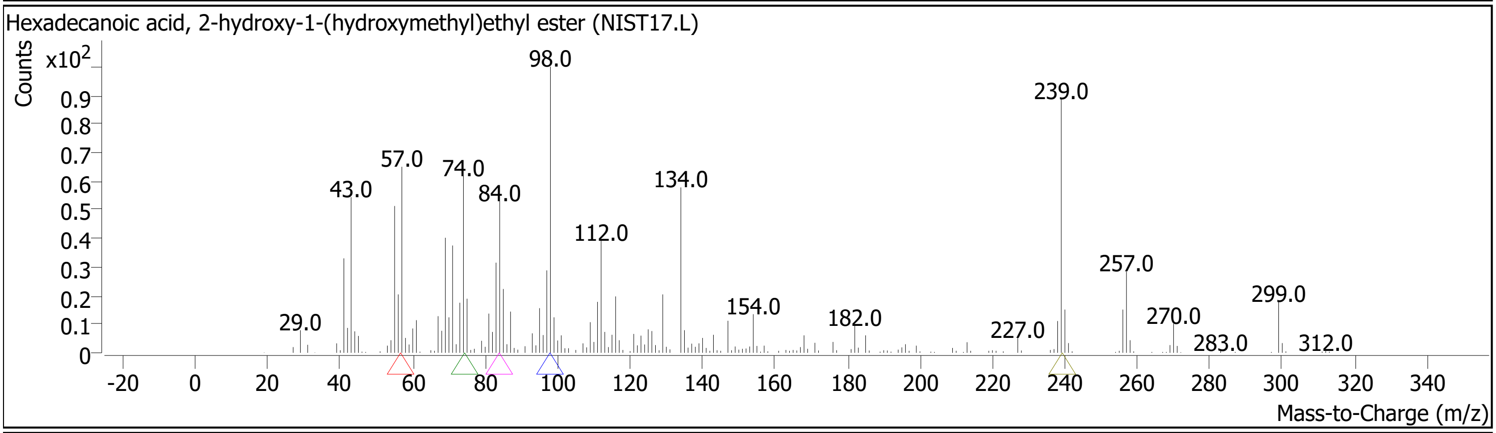

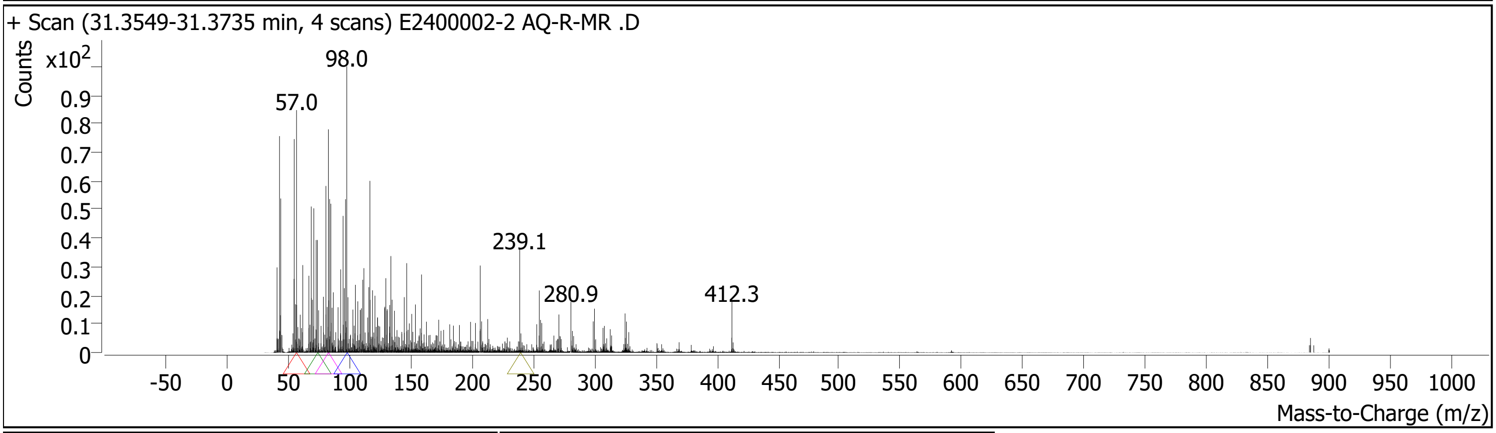

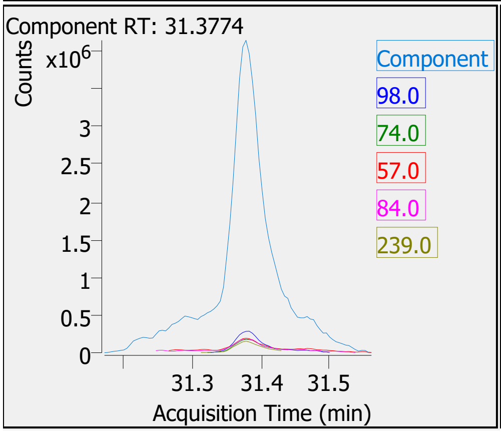

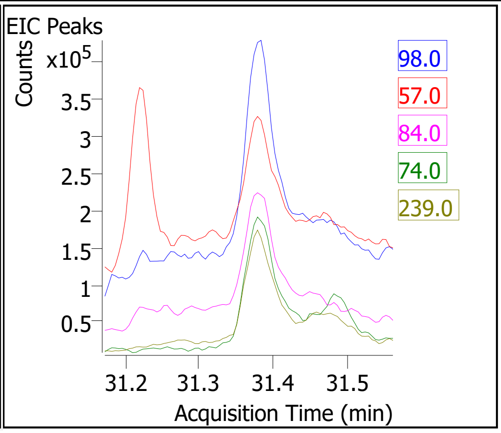

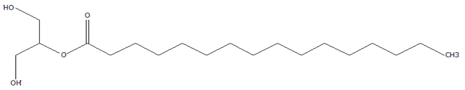

Generated at 09:38 AM on 04-04-2024

Library Search Results - NonTarget Hits with Details

Component RT	Compound Name	Component Area	Match Factor	CAS#	Formula	Estimated Conc.
31.6943	Phthalic acid, octyl 2-pentyl ester	553053.6	60.9	1000315-48-0	C21H32O4	

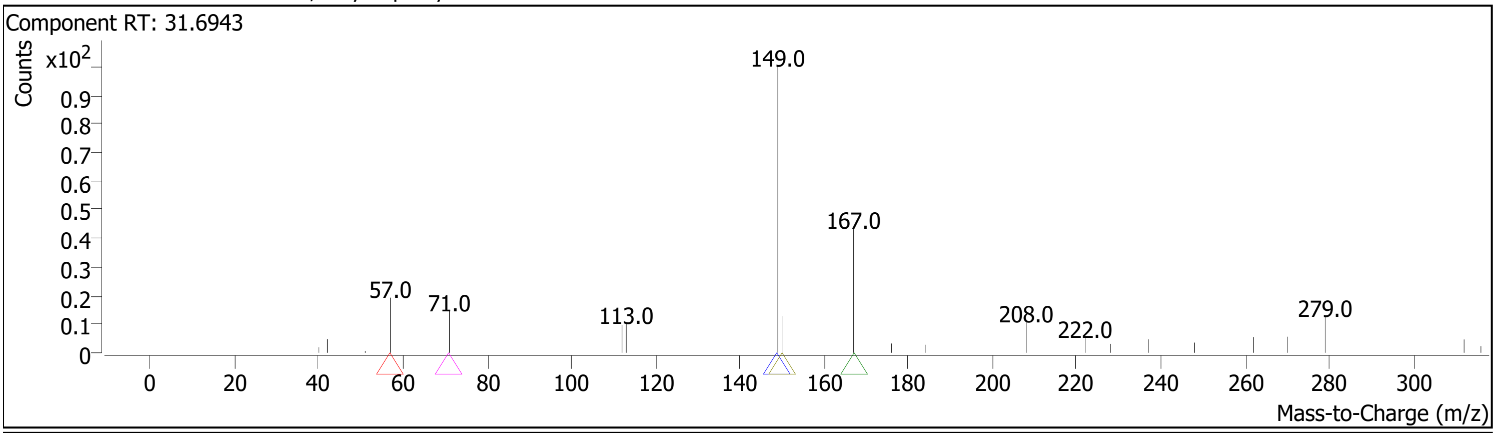

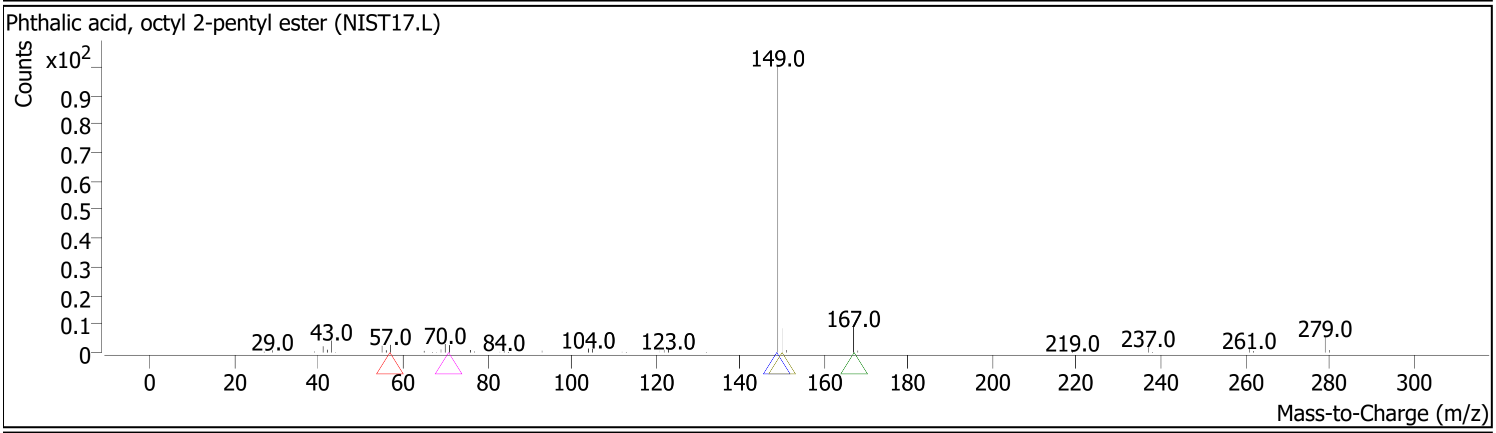

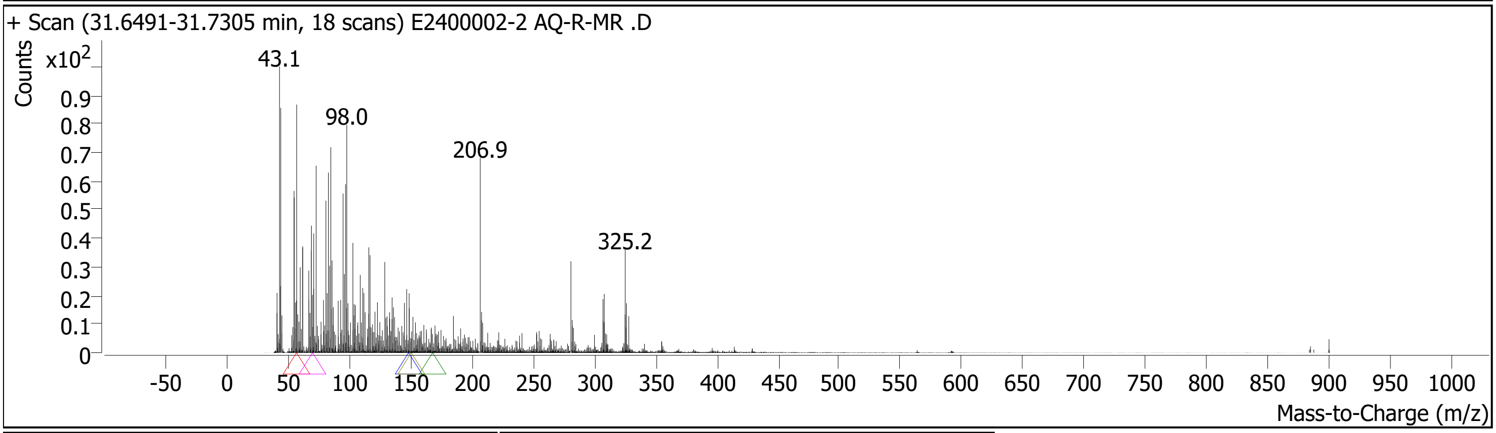

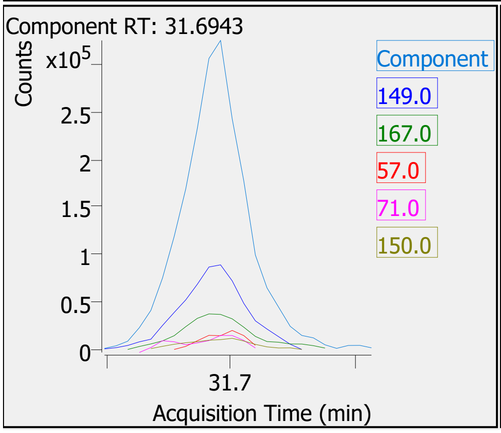

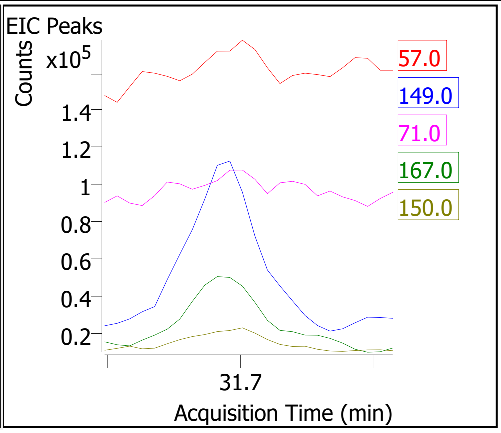

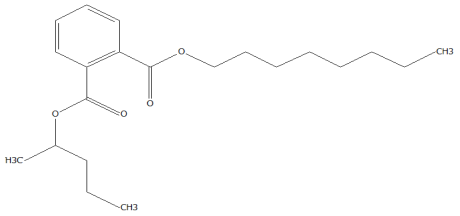

Library Search Results - NonTarget Hits with Details

Component RT	Compound Name	Component Area	Match Factor	CAS#	Formula	Estimated Conc.
32.8225	Myristic acid, 4-methoxyphenyl ester	660588.4	67.0	1000357-93-0	C21H34O3	

Component RT: 32.8225

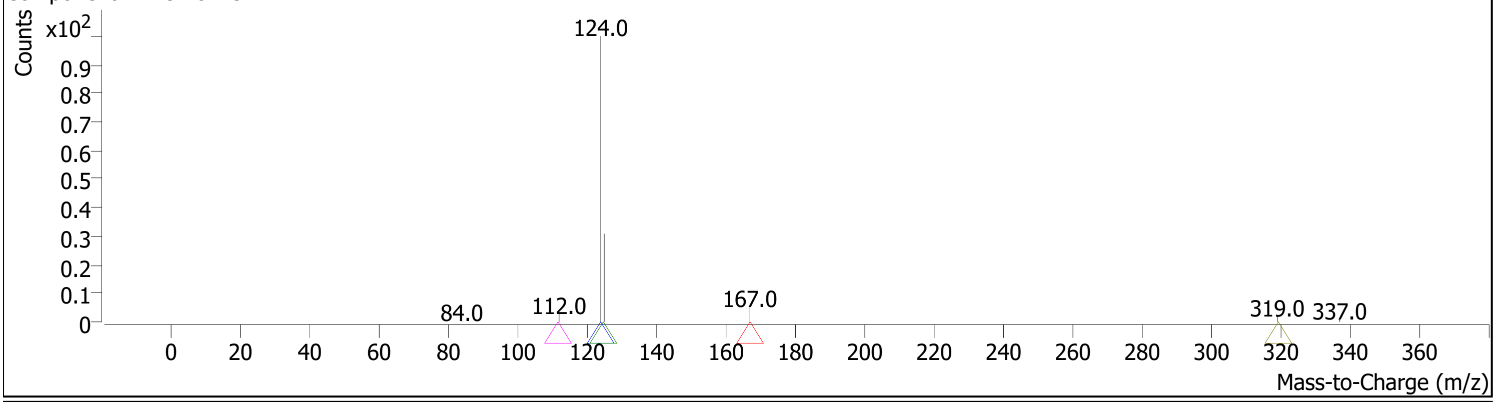

Myristic acid, 4-methoxyphenyl ester (NIST17.L)

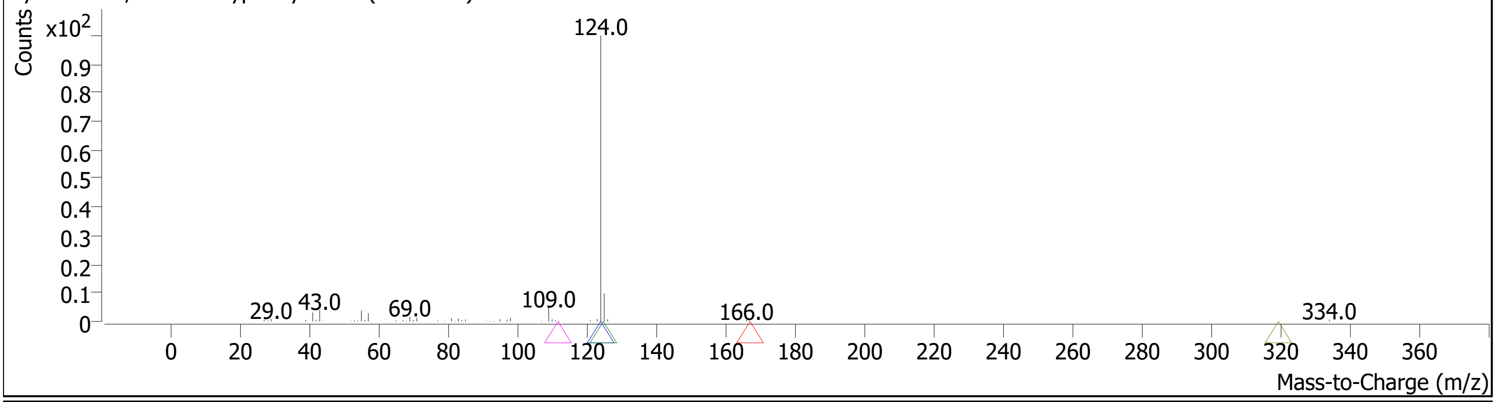

+ Scan (32.8119-32.8306 min, 5 scans) E2400002-2 AQ-R-MR .D

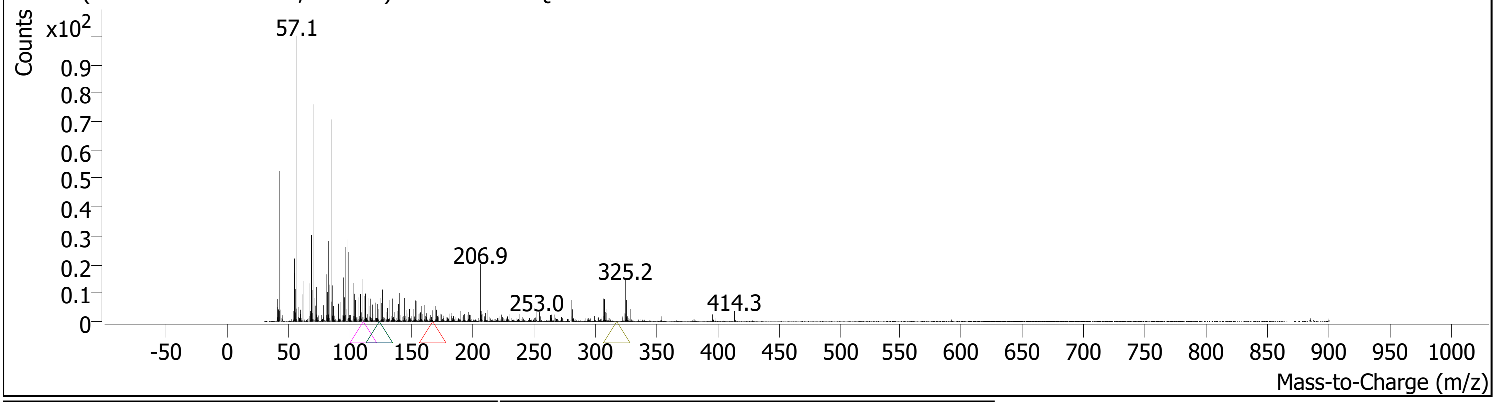

Component RT: 32.8225

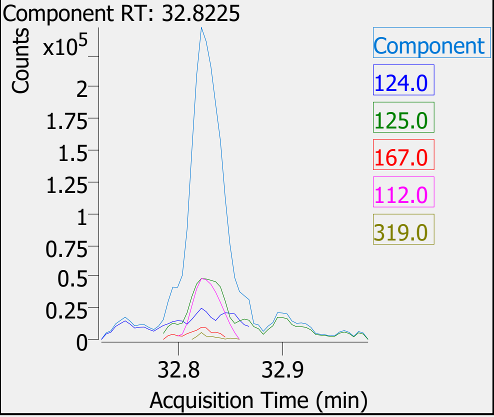

EIC Peaks

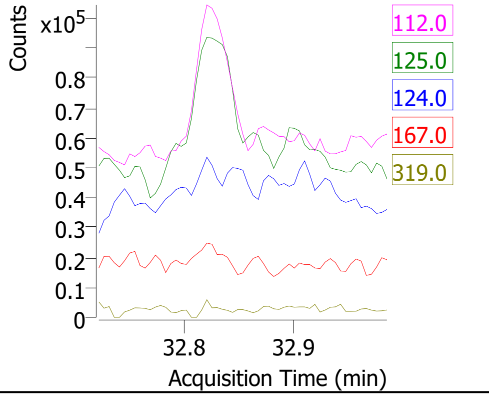

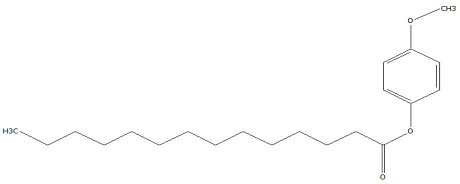

Generated at 09:38 AM on 04-04-2024

Component RT	Compound Name	Component Area	Match Factor	CAS#	Formula	Estimated Conc.
32.8266	Hentriacontane	14565868.0	90.1	630-04-6	C31H64	

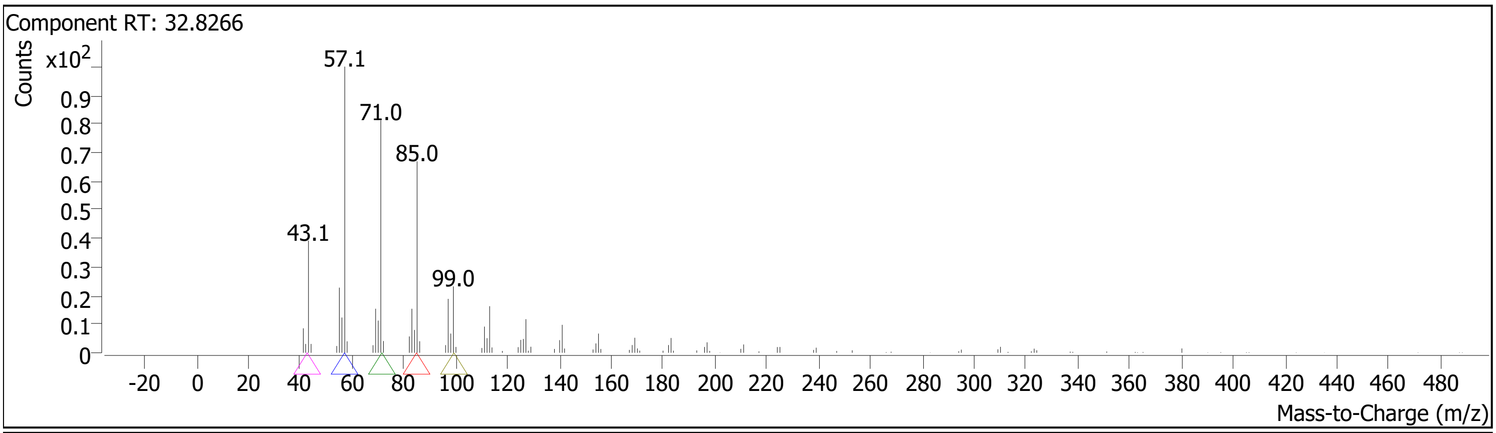

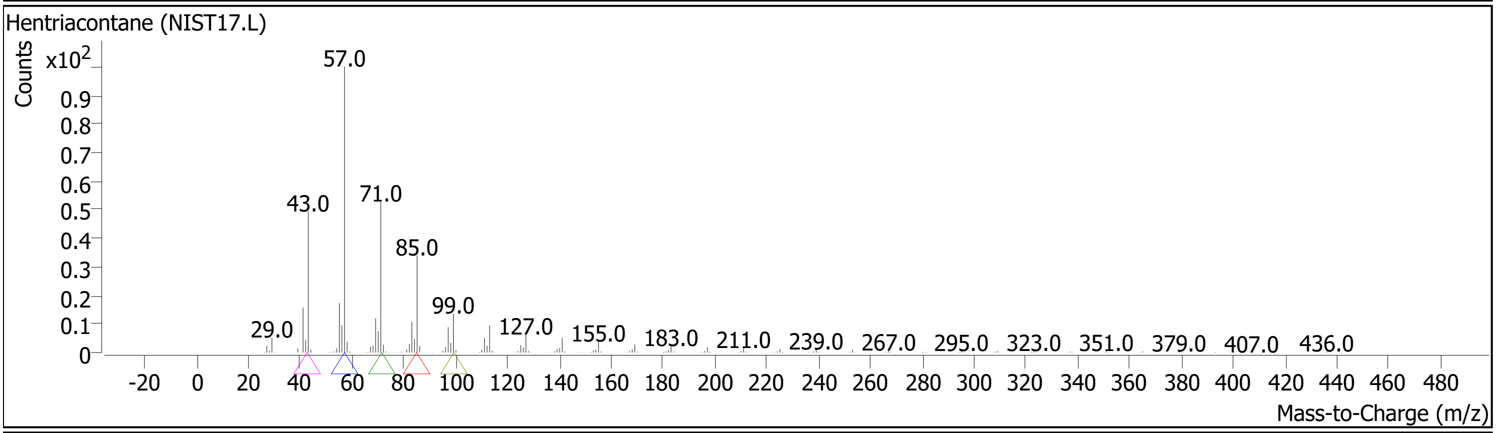

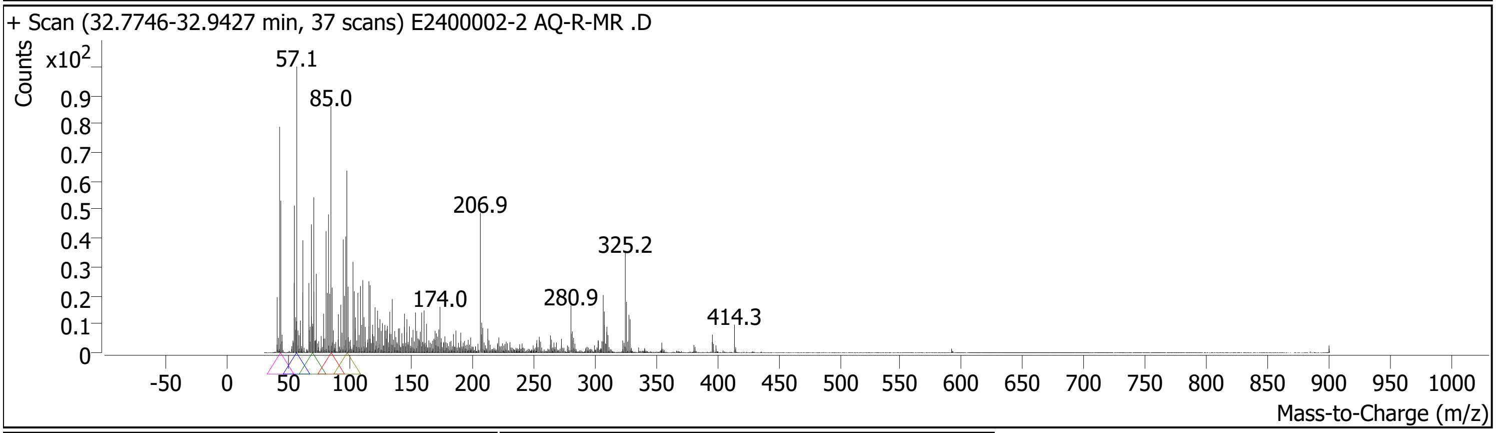

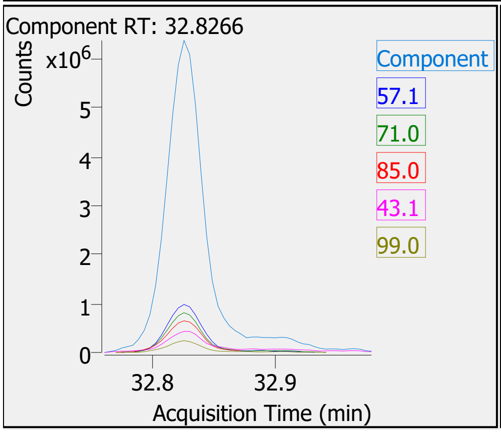

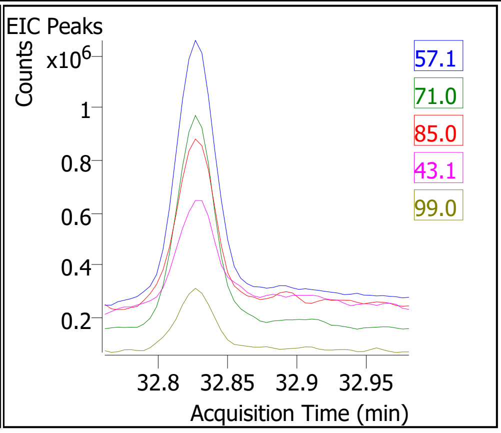

Component RT	Compound Name	Component Area	Match Factor	CAS#	Formula	Estimated Conc.
32.8867	1,3-Benzenediol, O-(4-butylbenzoyl)-O'-(4-fluorobenzoyl)-	1524776.2	60.6	1000345-61-1	C24H21FO4	

Component RT: 32.8867

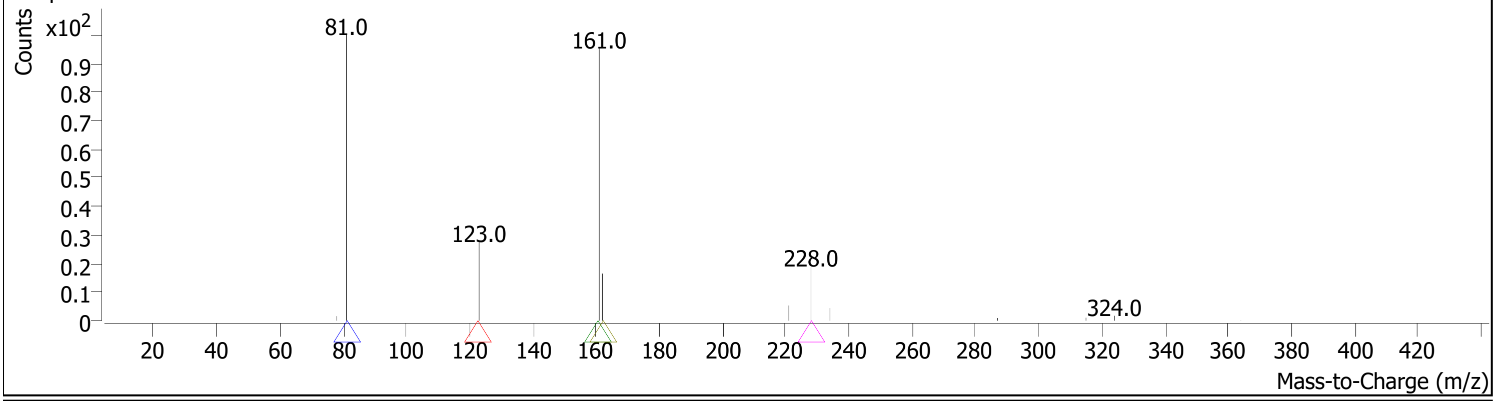

1,3-Benzenediol, O-(4-butylbenzoyl)-O'-(4-fluorobenzoyl)- (NIST17.L)

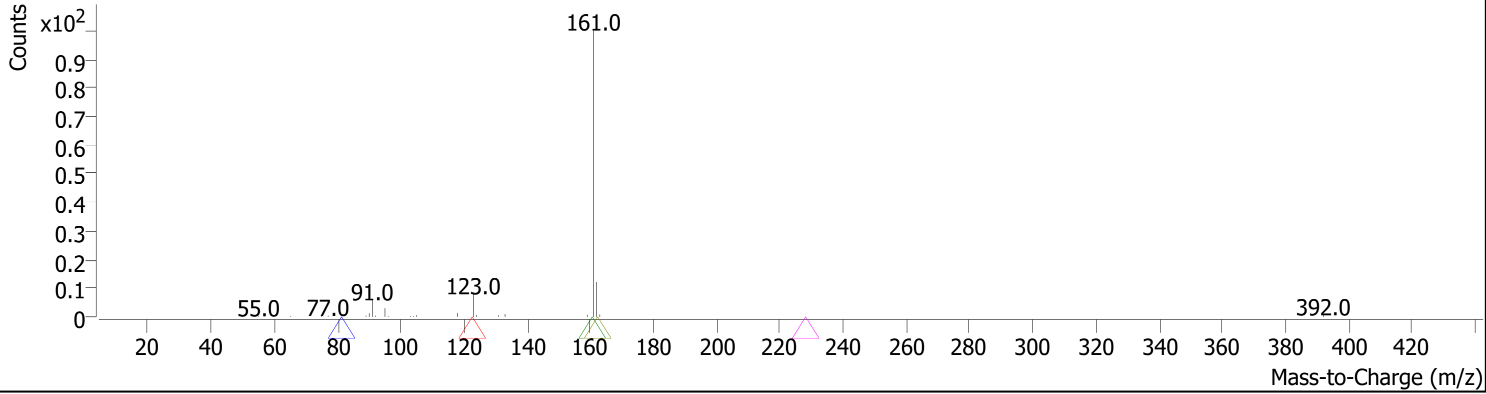

+ Scan (32.8493-32.9427 min, 21 scans) E2400002-2 AQ-R-MR .D

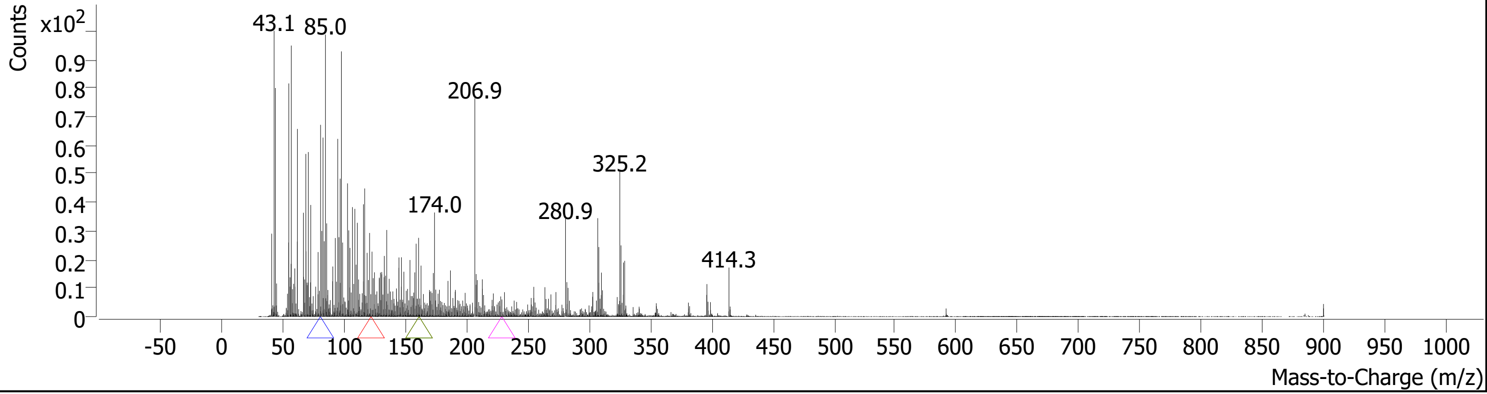

Component RT: 32.8867

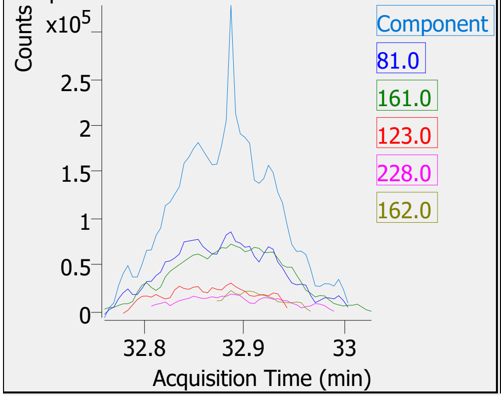

EIC Peaks

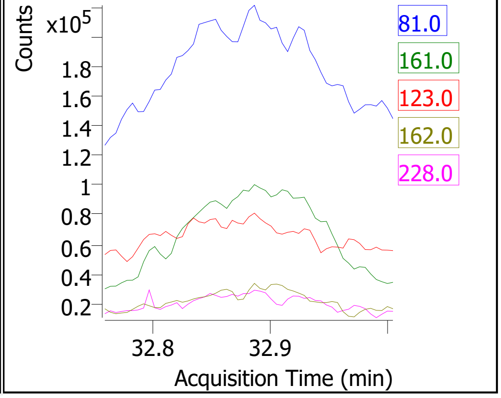

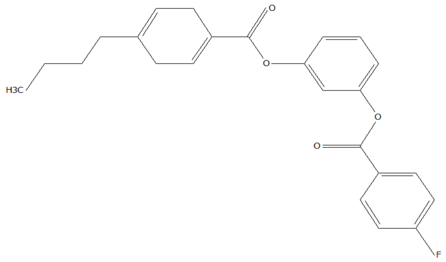

Component RT	Compound Name	Component Area	Match Factor	CAS#	Formula	Estimated Conc.
33.0504	Butylamine, N-acetyl-1-cyano-2-ethyl-	2135151.5	71.4	1000227-04-1	C9H16N2O	

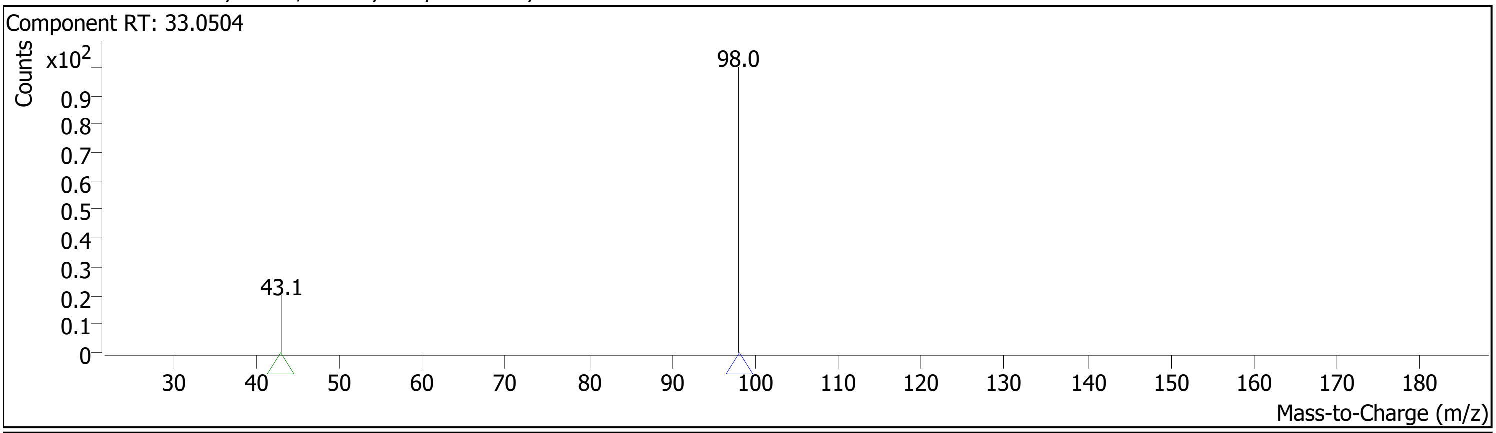

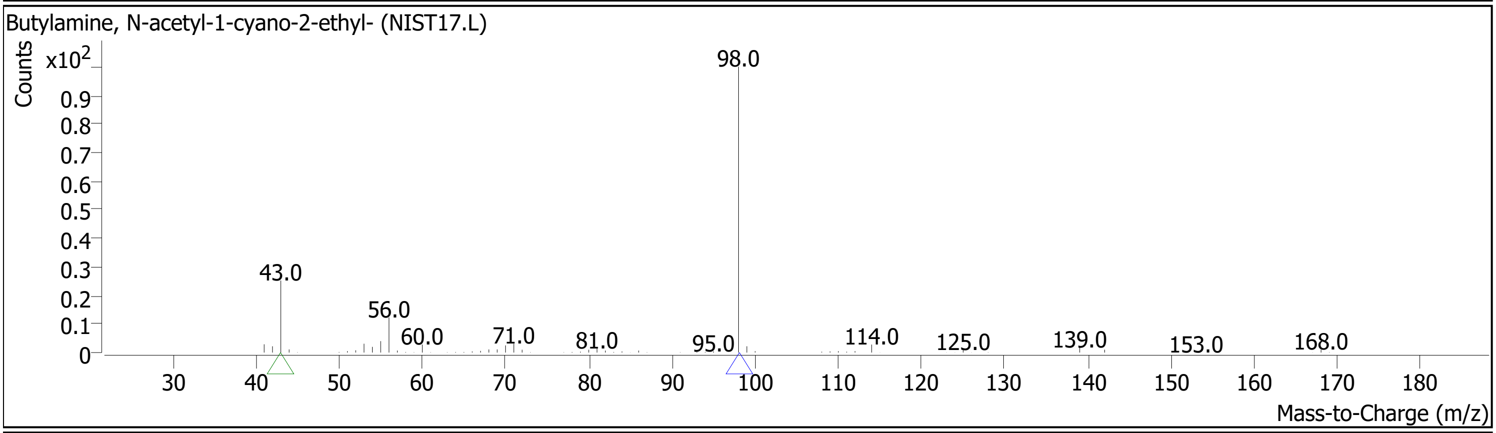

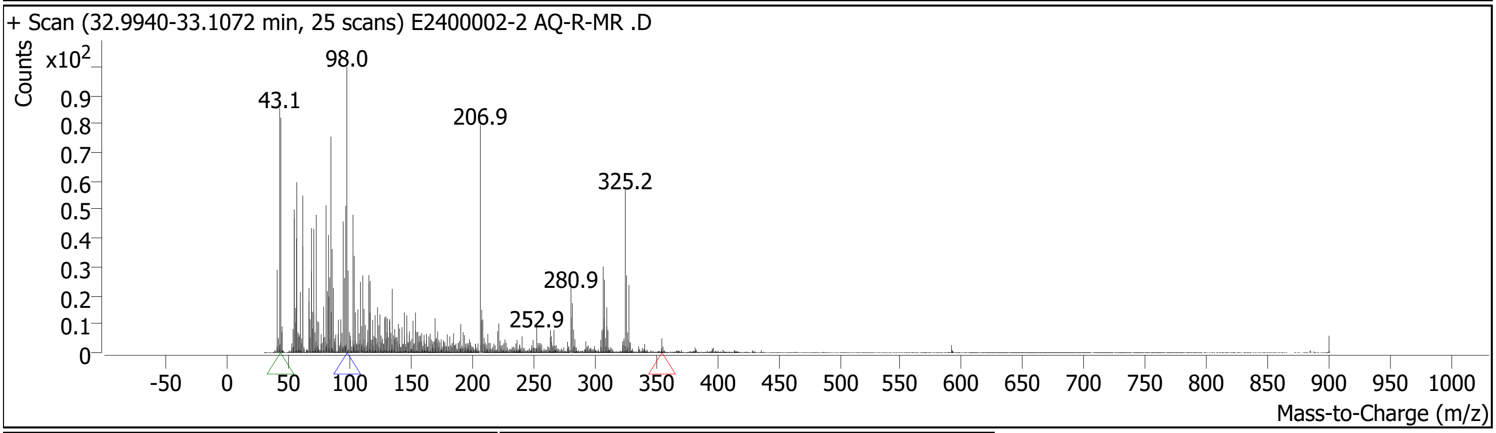

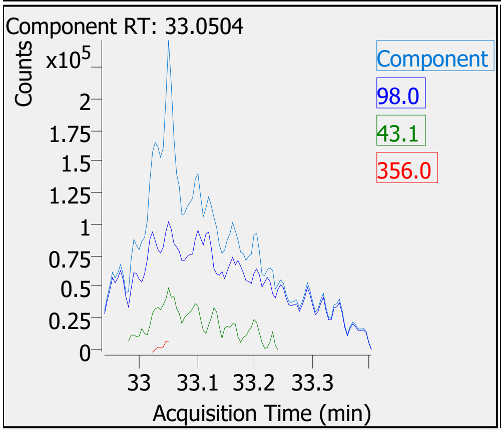

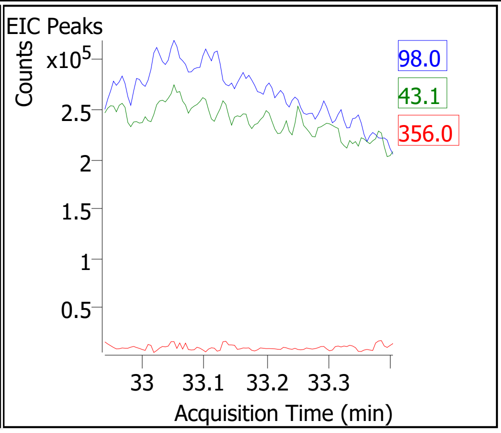

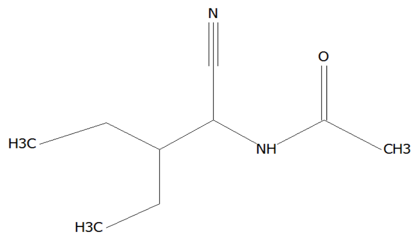

Component RT	Compound Name	Component Area	Match Factor	CAS#	Formula	Estimated Conc.
33.6070	Octacosane	6716655.7	84.0	630-02-4	C28H58	

Component RT: 33.6070

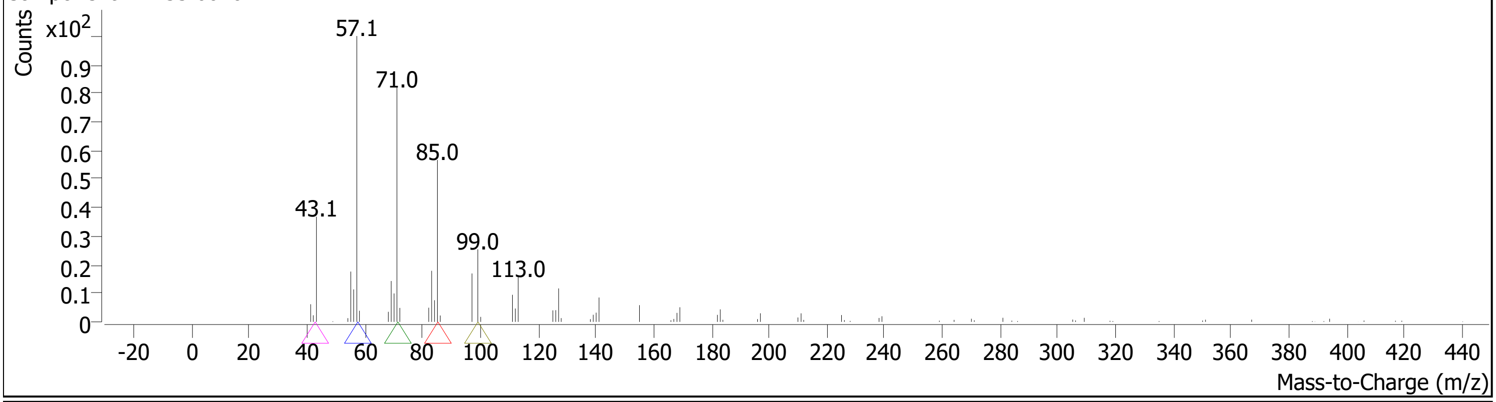

Octacosane (NIST17.L)

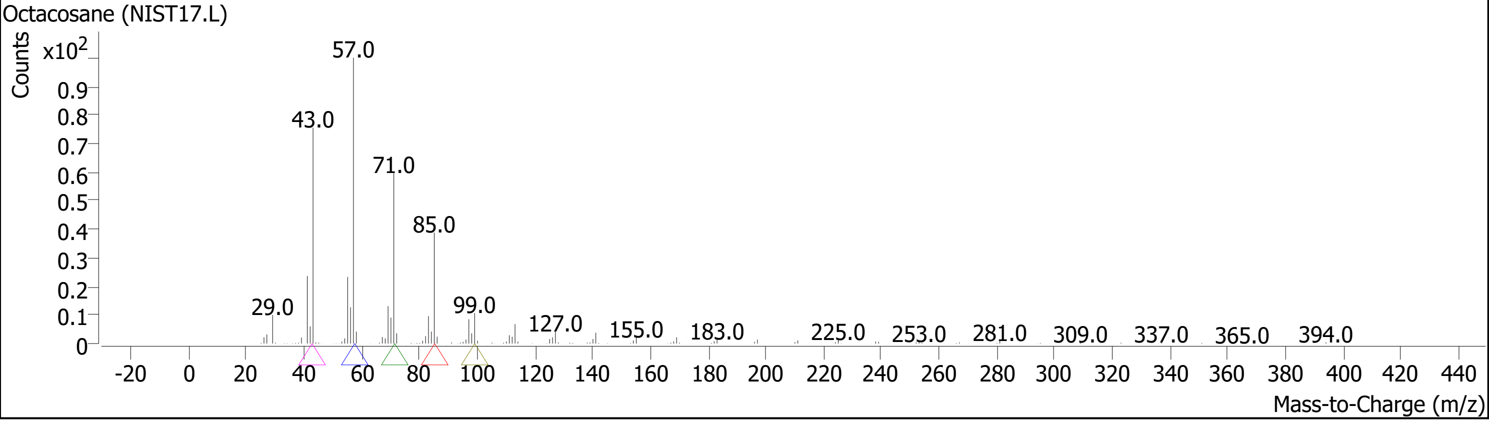

+ Scan (33.5595-33.6491 min, 19 scans) E2400002-2 AQ-R-MR .D

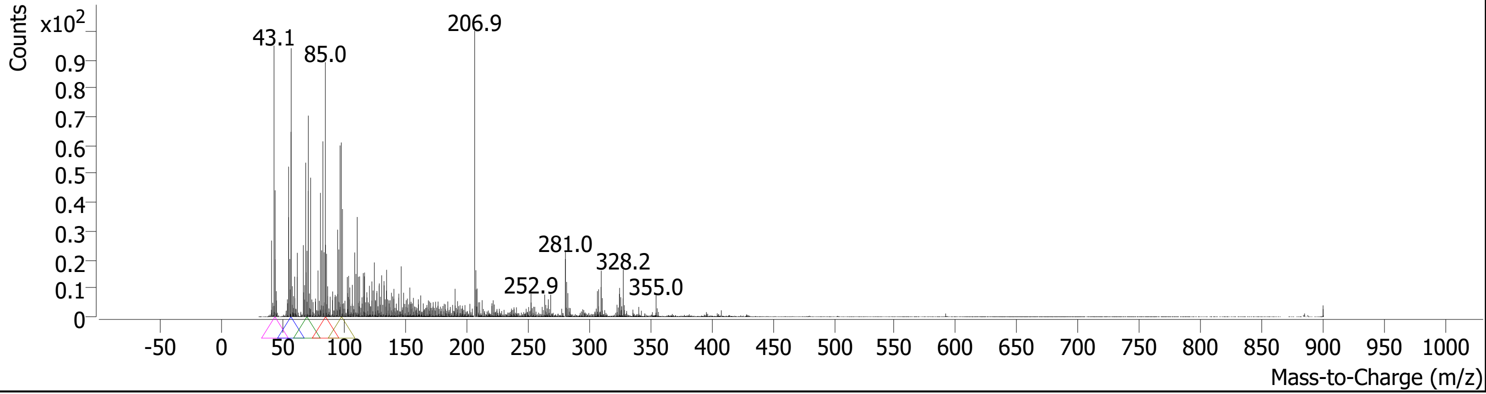

Component RT: 33.6070

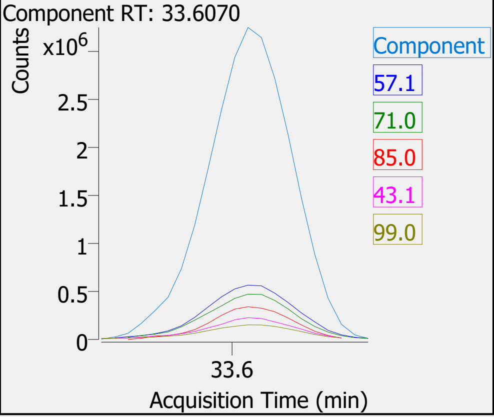

EIC Peaks

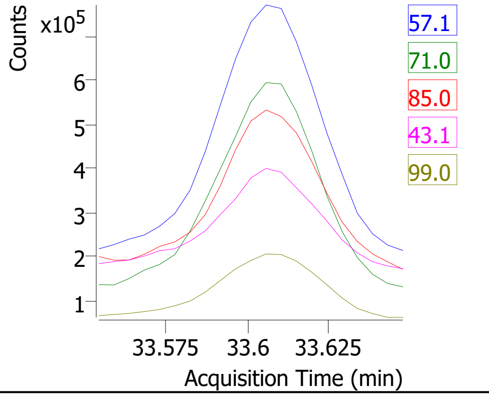

Generated at 09:38 AM on 04-04-2024

Component RT	Compound Name	Component Area	Match Factor	CAS#	Formula	Estimated Conc.
33.9098	Squalene	7014632.2	85.5	111-02-4	C30H50	

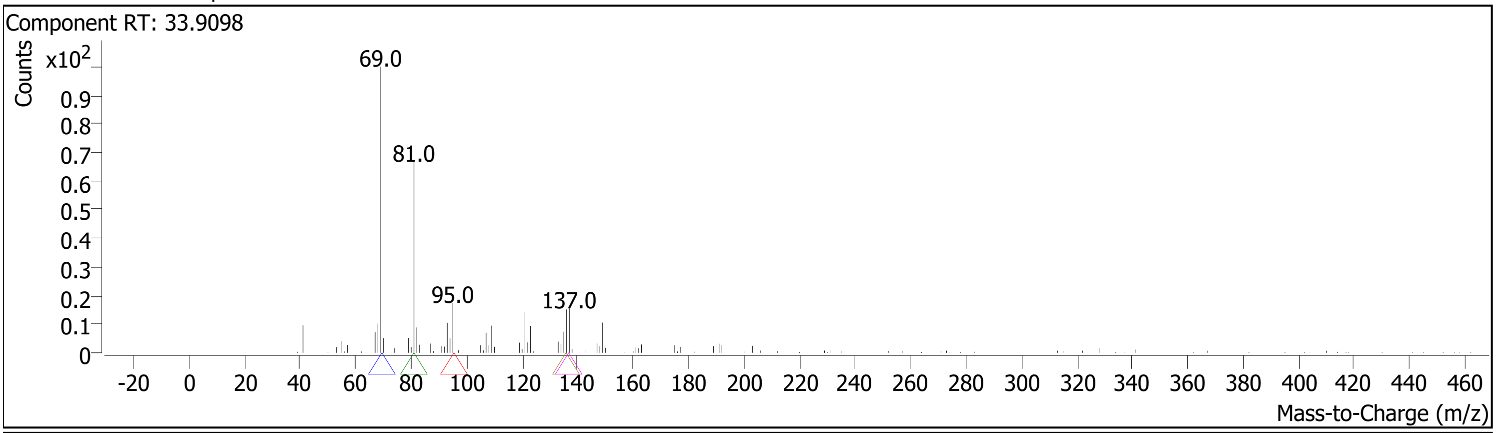

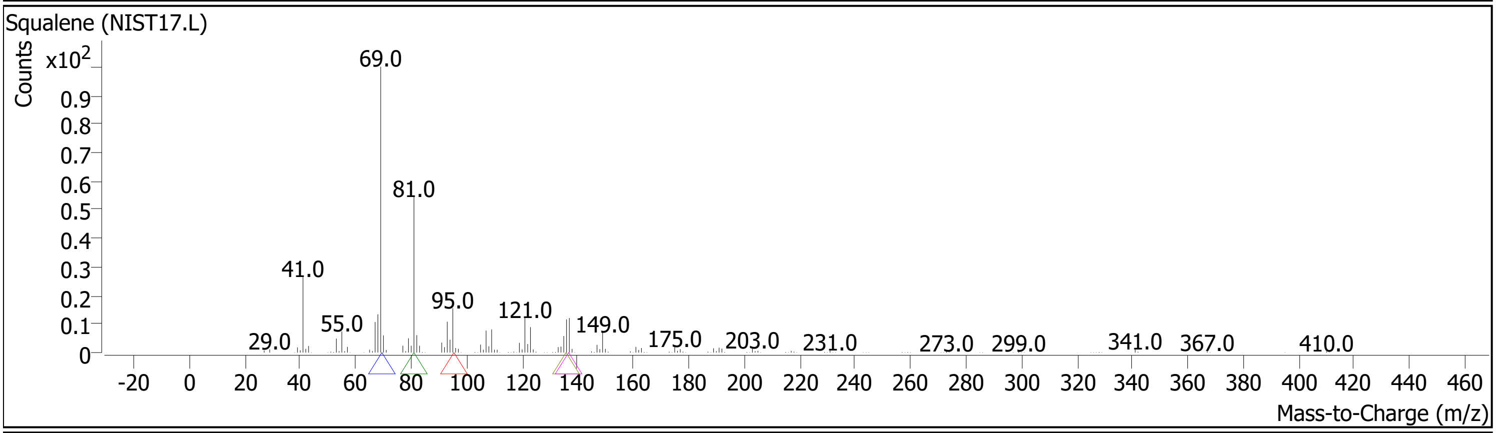

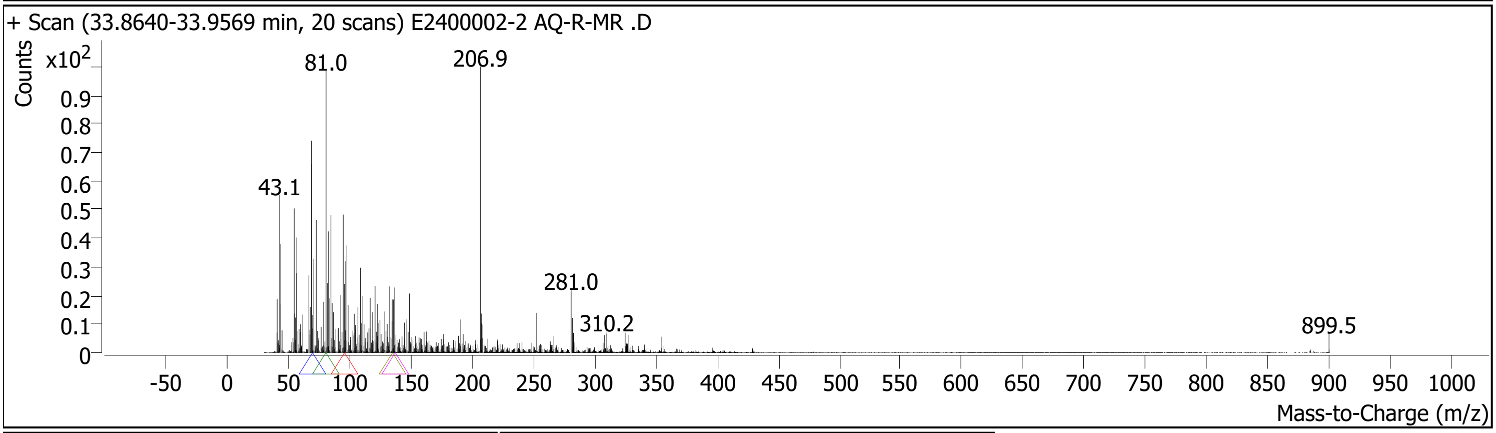

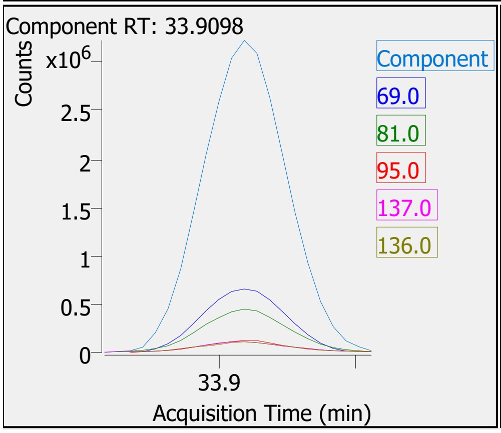

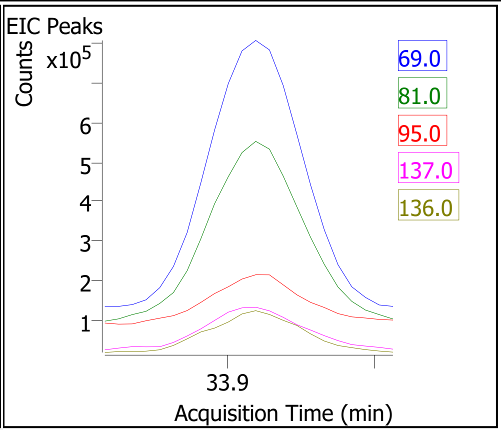

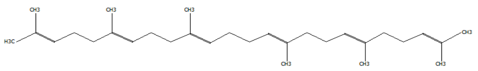

Component RT	Compound Name	Component Area	Match Factor	CAS#	Formula	Estimated Conc.
34.4893	(+)-(S)-Isocorydine	7959157.1	79.6	475-67-2	C20H23NO4	

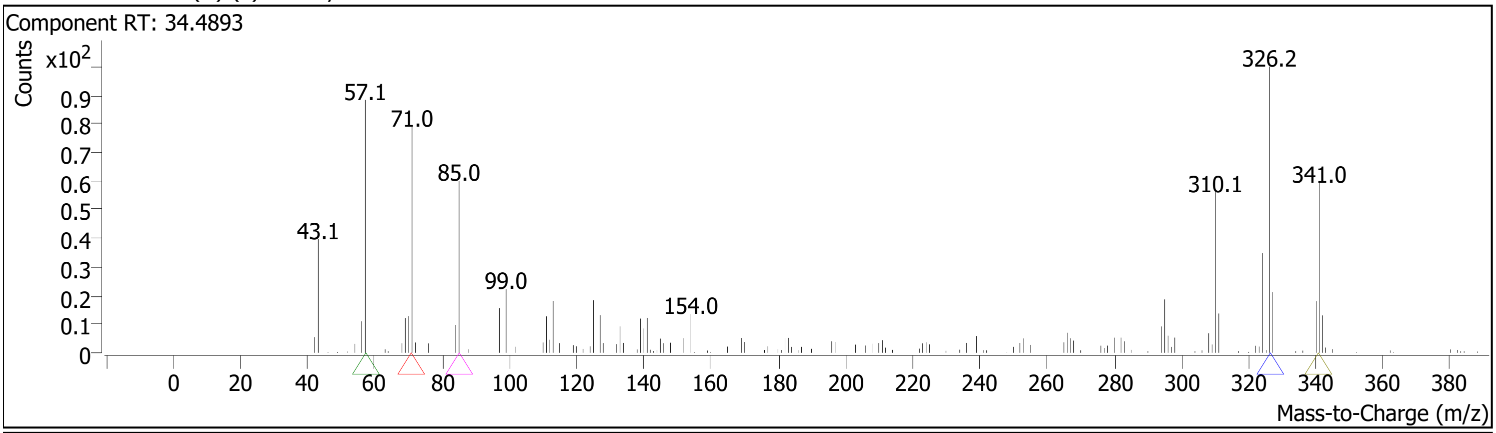

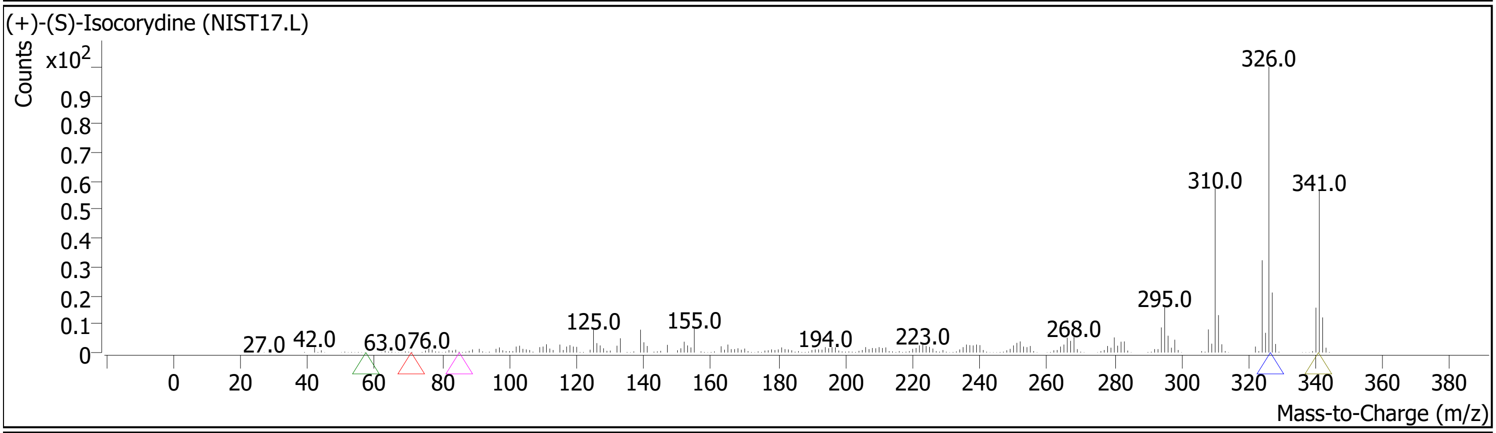

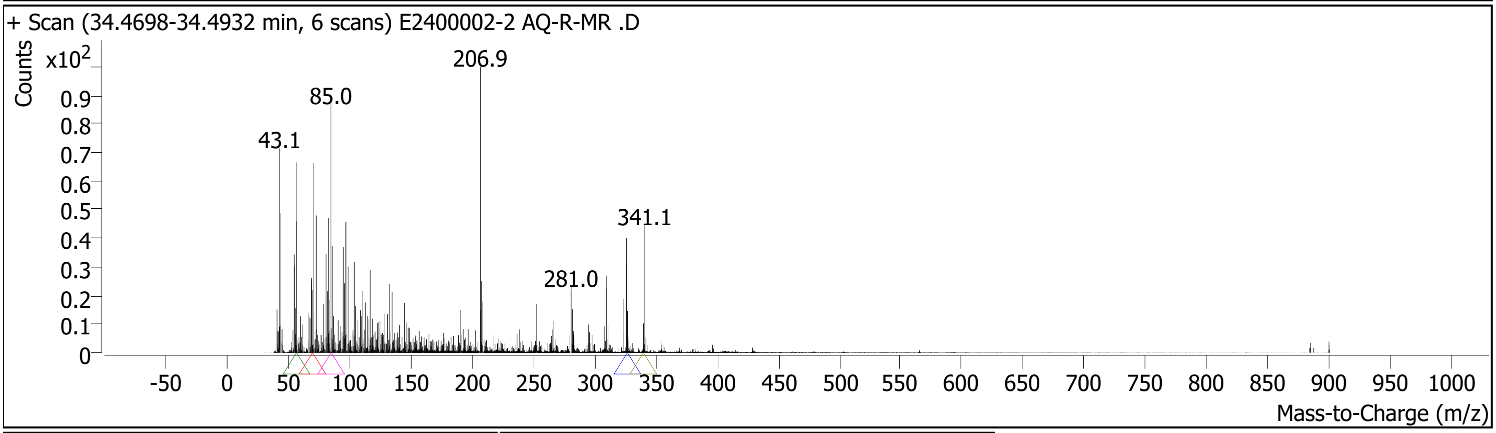

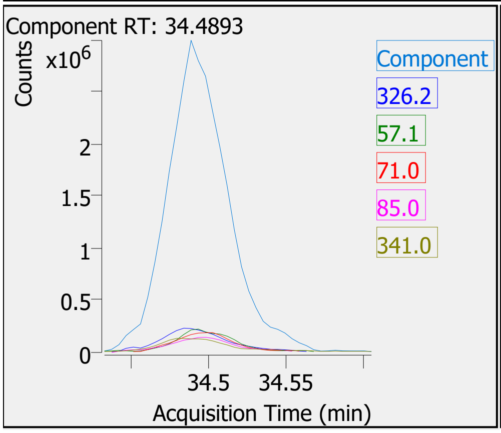

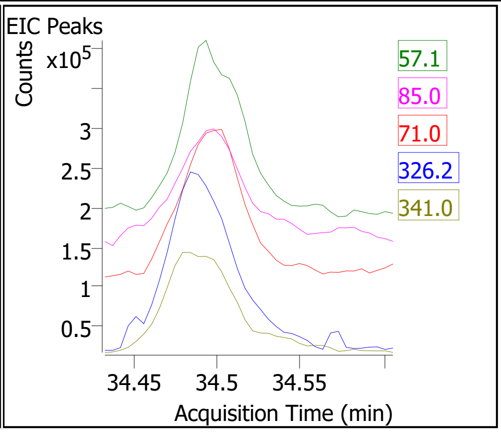

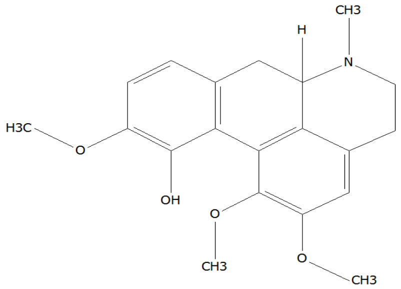

Supplement: Supplementary file 1 [file biomedicines-13-01658-s001.zip › GC MS Results.pdf]
