# Supplementary figures and images for: Targeting CDK4/6 in Cancer: Molecular Docking and Cytotoxic Evaluation of Thottea siliquosa Root Extract
Source: Biomedicines. 2025 Jul 7;13(7):1658. doi: 10.3390/biomedicines13071658 (PMC12292890; doi:10.3390/biomedicines13071658)

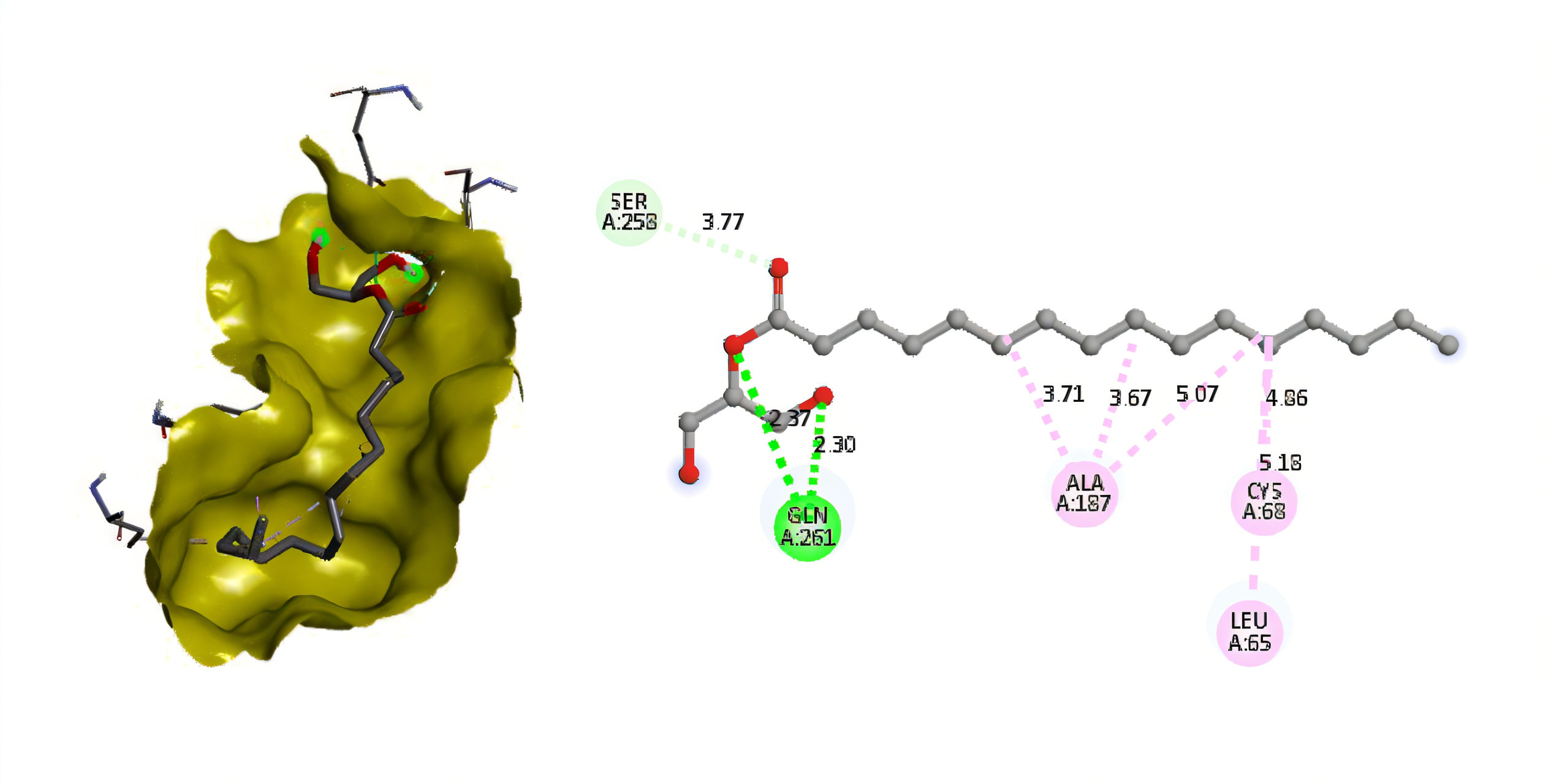

Supplement: Supplementary file 1 [file biomedicines-13-01658-s001.zip › Docking Interaction Images/CDK4_2-palmitoylglycerol.jpg]

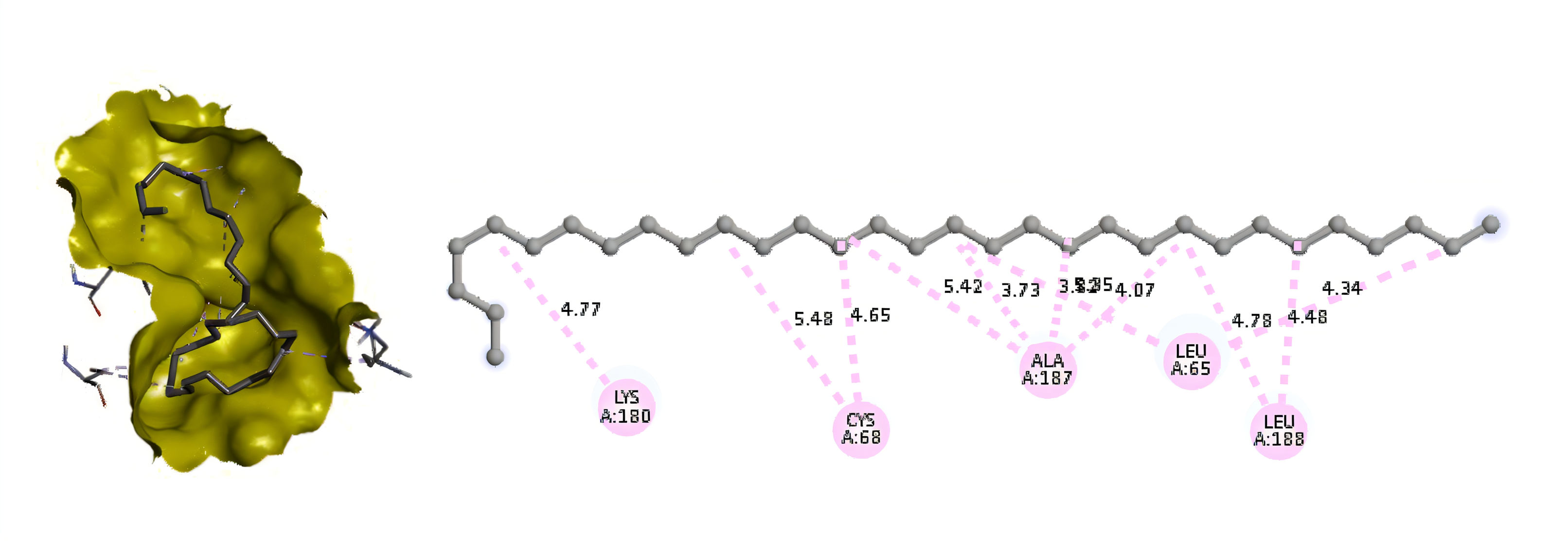

Supplement: Supplementary file 1 [file biomedicines-13-01658-s001.zip › Docking Interaction Images/CDK4_hentriacontane.jpg]

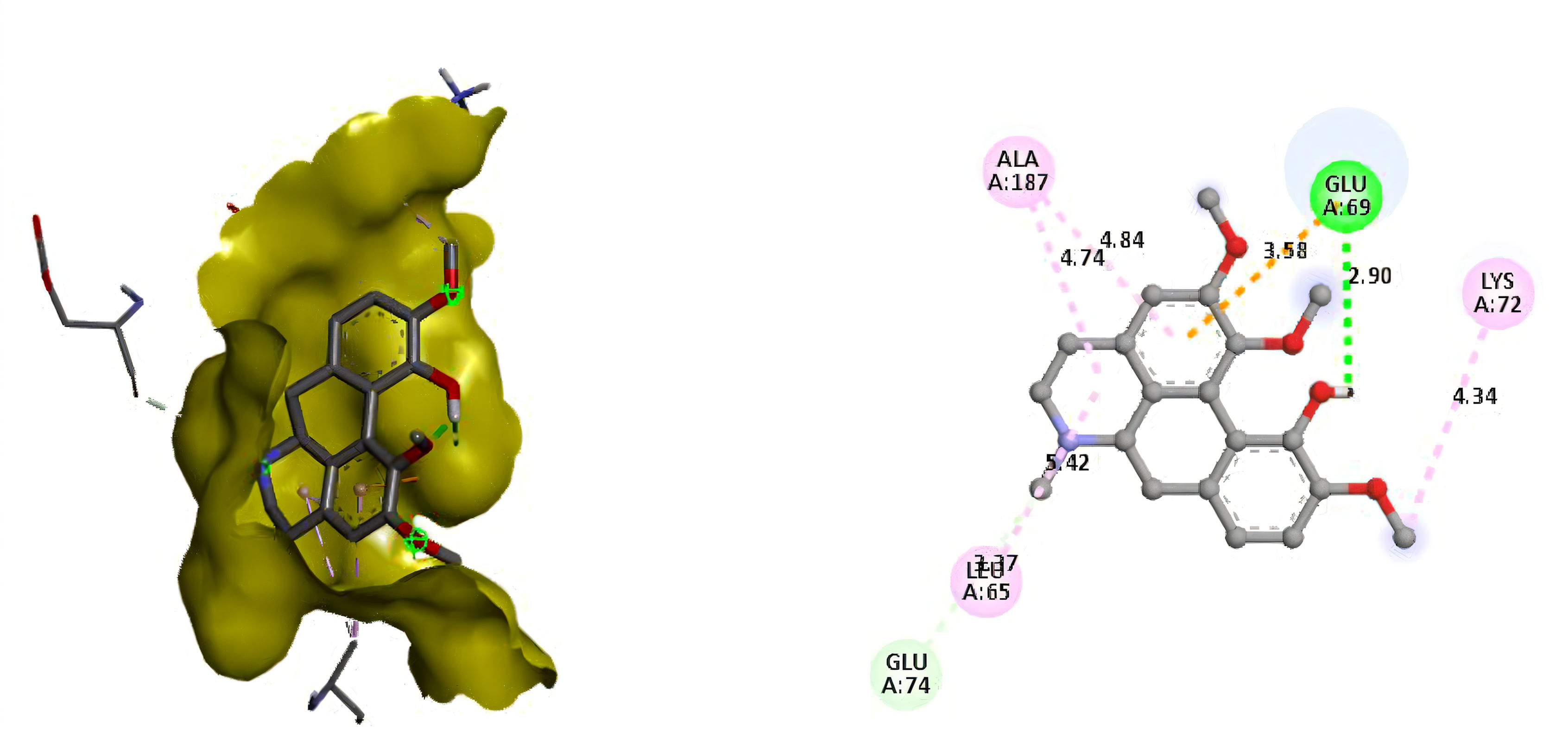

Supplement: Supplementary file 1 [file biomedicines-13-01658-s001.zip › Docking Interaction Images/CDK4_isocorydine.jpg]

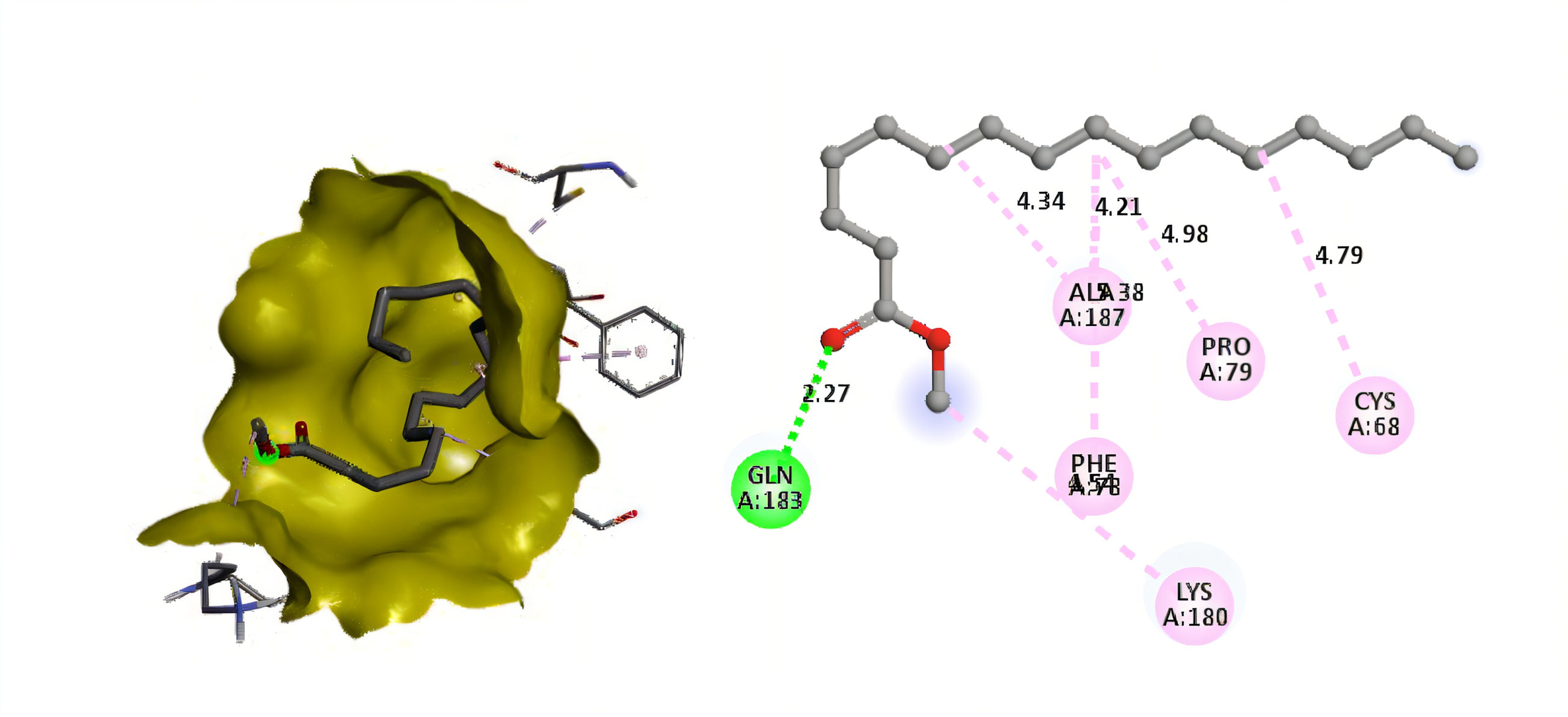

Supplement: Supplementary file 1 [file biomedicines-13-01658-s001.zip › Docking Interaction Images/CDK4_methylpalmitate.jpg]

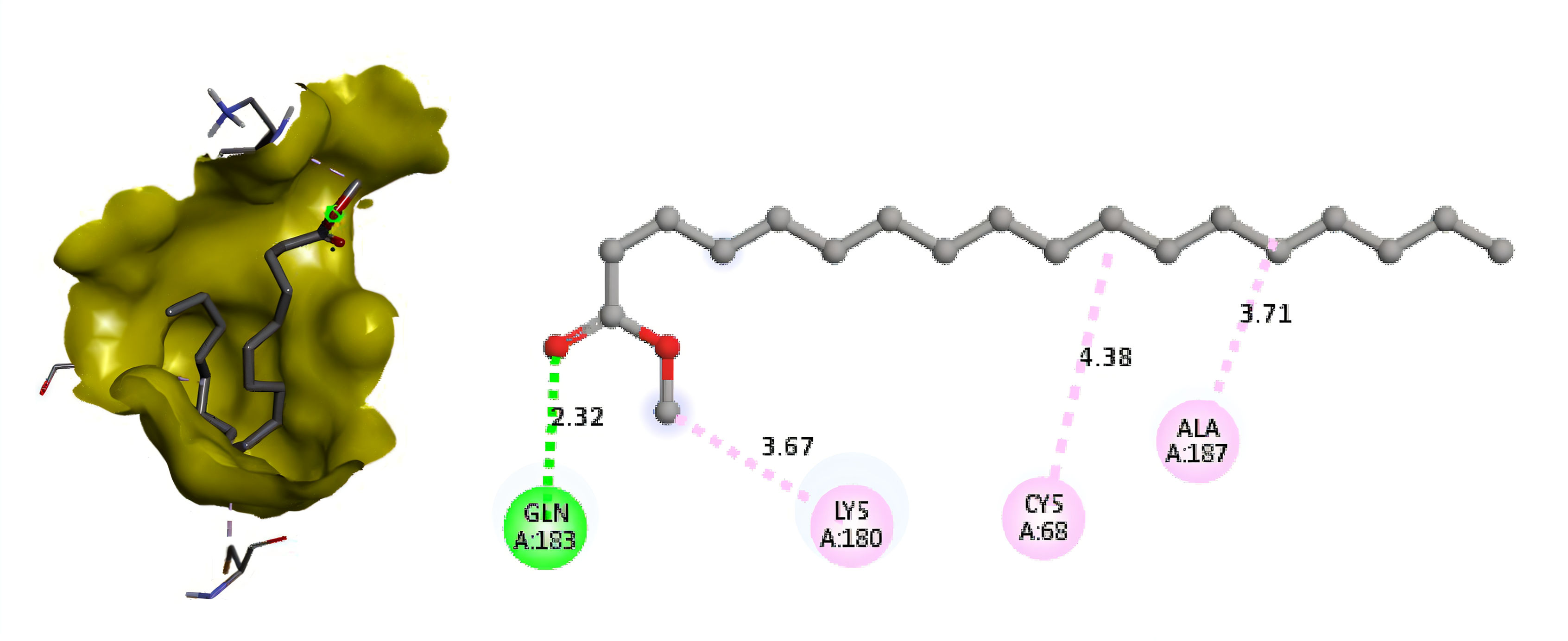

Supplement: Supplementary file 1 [file biomedicines-13-01658-s001.zip › Docking Interaction Images/CDK4_methylstearate.jpg]

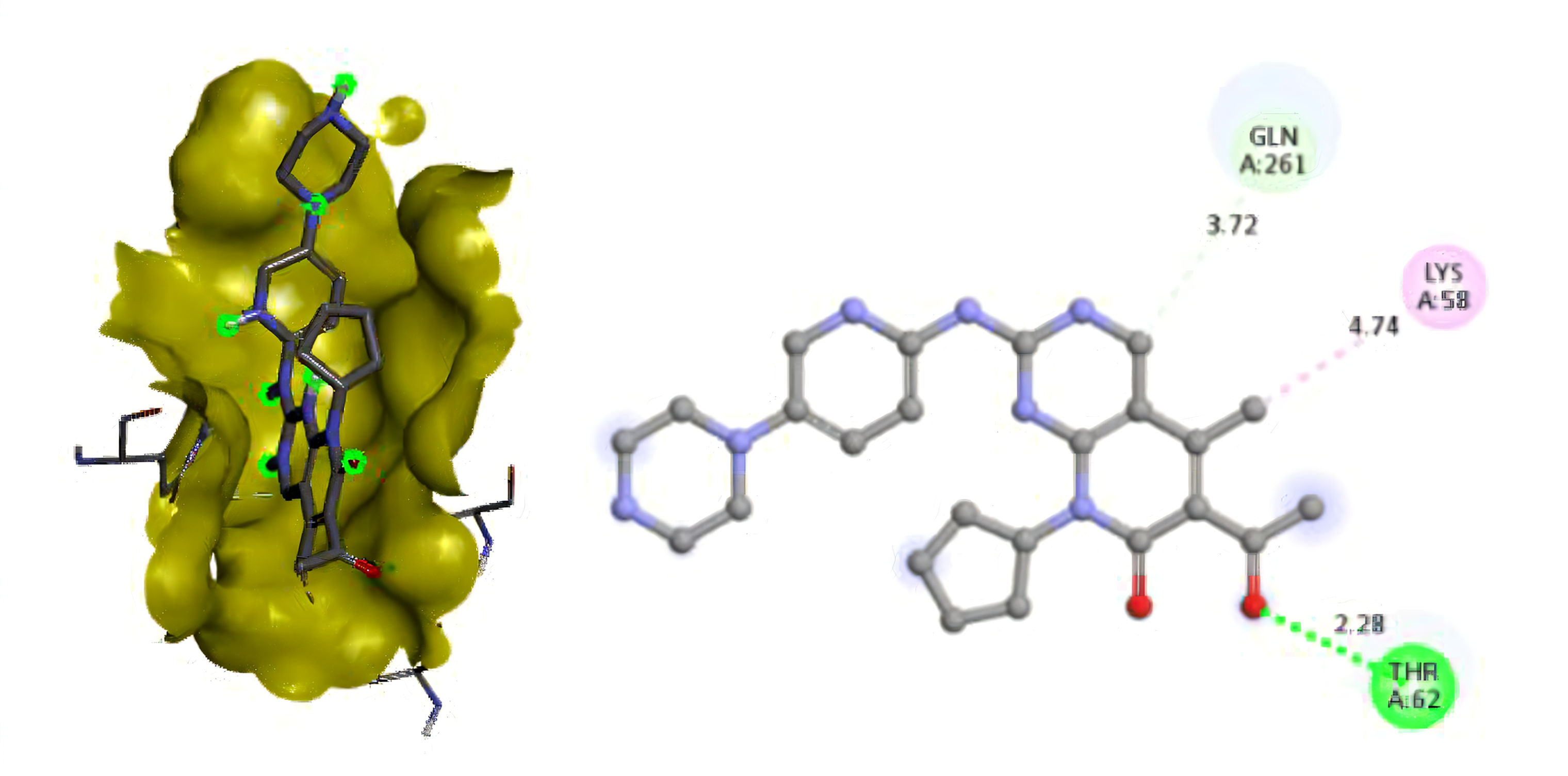

Supplement: Supplementary file 1 [file biomedicines-13-01658-s001.zip › Docking Interaction Images/CDK4_palbociclib.jpg]

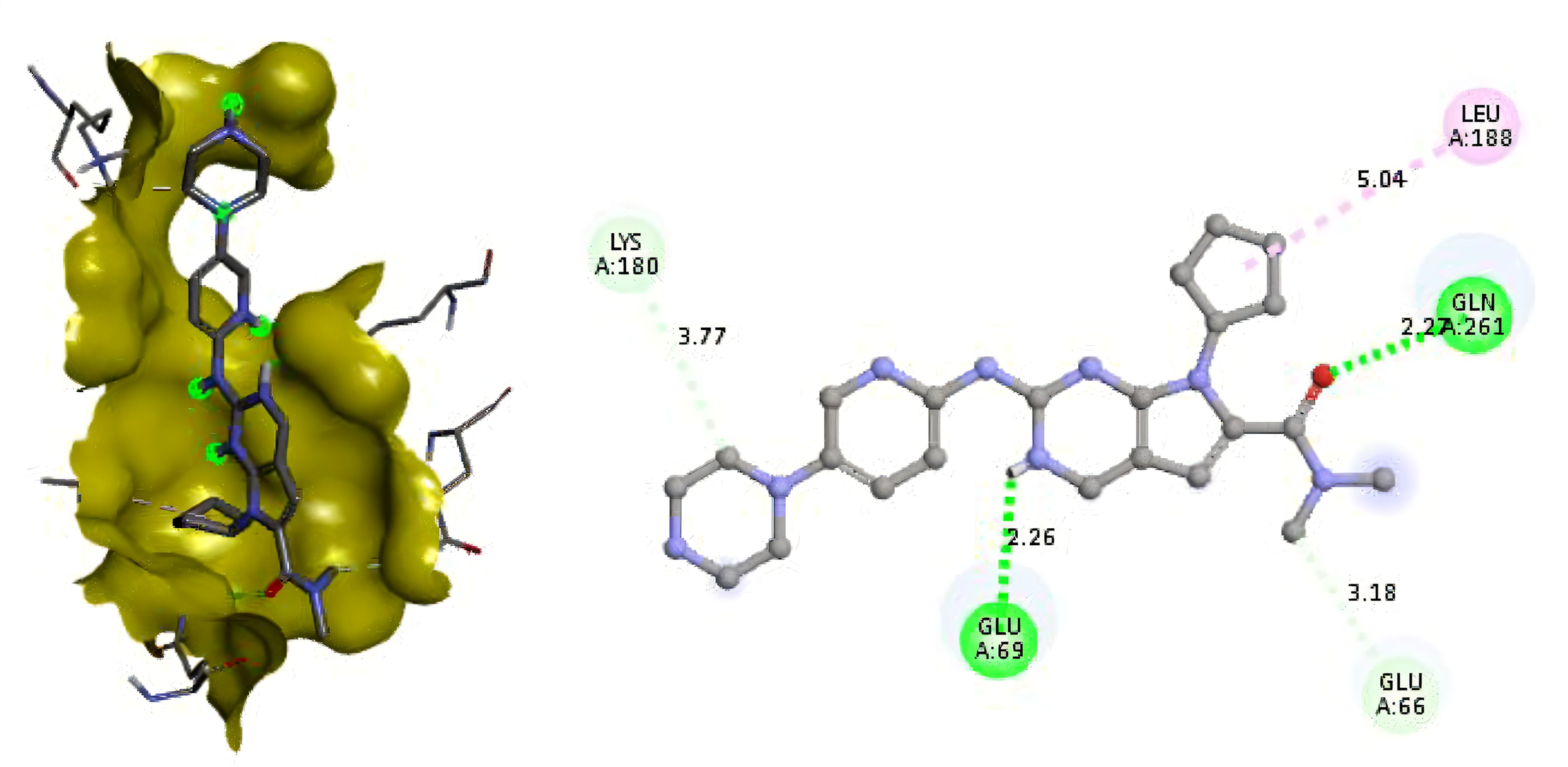

Supplement: Supplementary file 1 [file biomedicines-13-01658-s001.zip › Docking Interaction Images/CDK4_ribociclib.jpg]

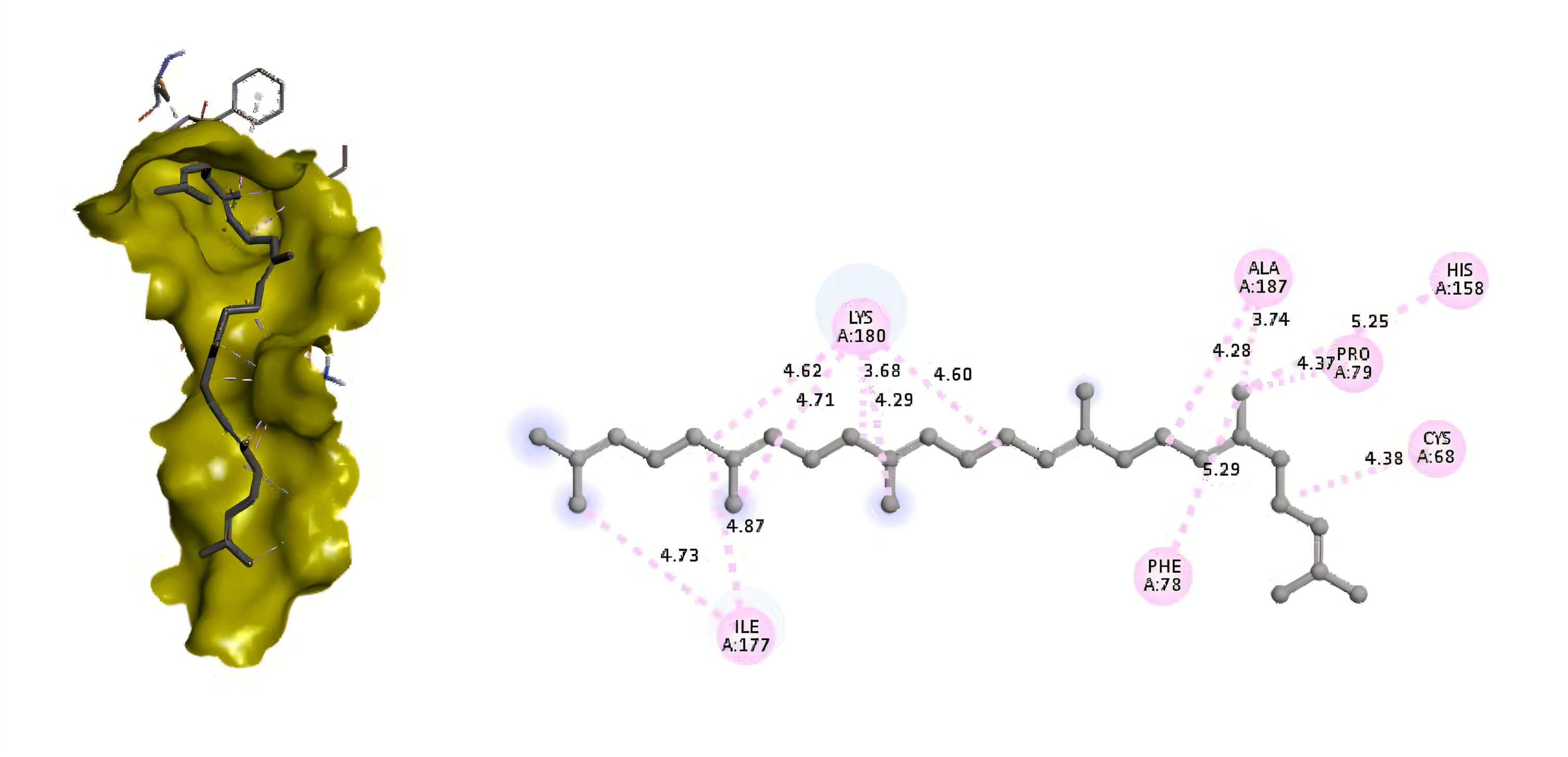

Supplement: Supplementary file 1 [file biomedicines-13-01658-s001.zip › Docking Interaction Images/CDK4_squalene.jpg]

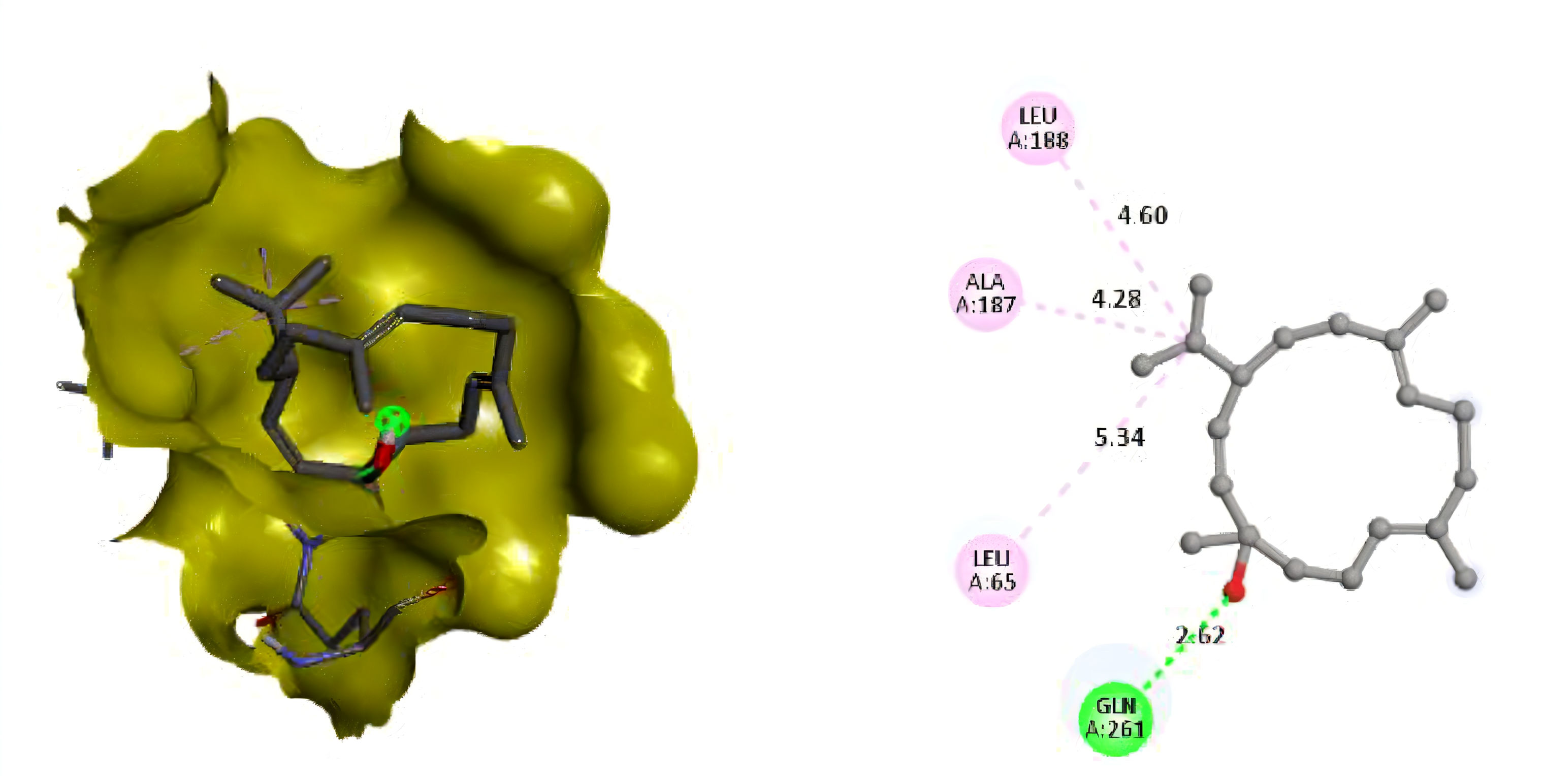

Supplement: Supplementary file 1 [file biomedicines-13-01658-s001.zip › Docking Interaction Images/CDK4_thunbergol.jpg]

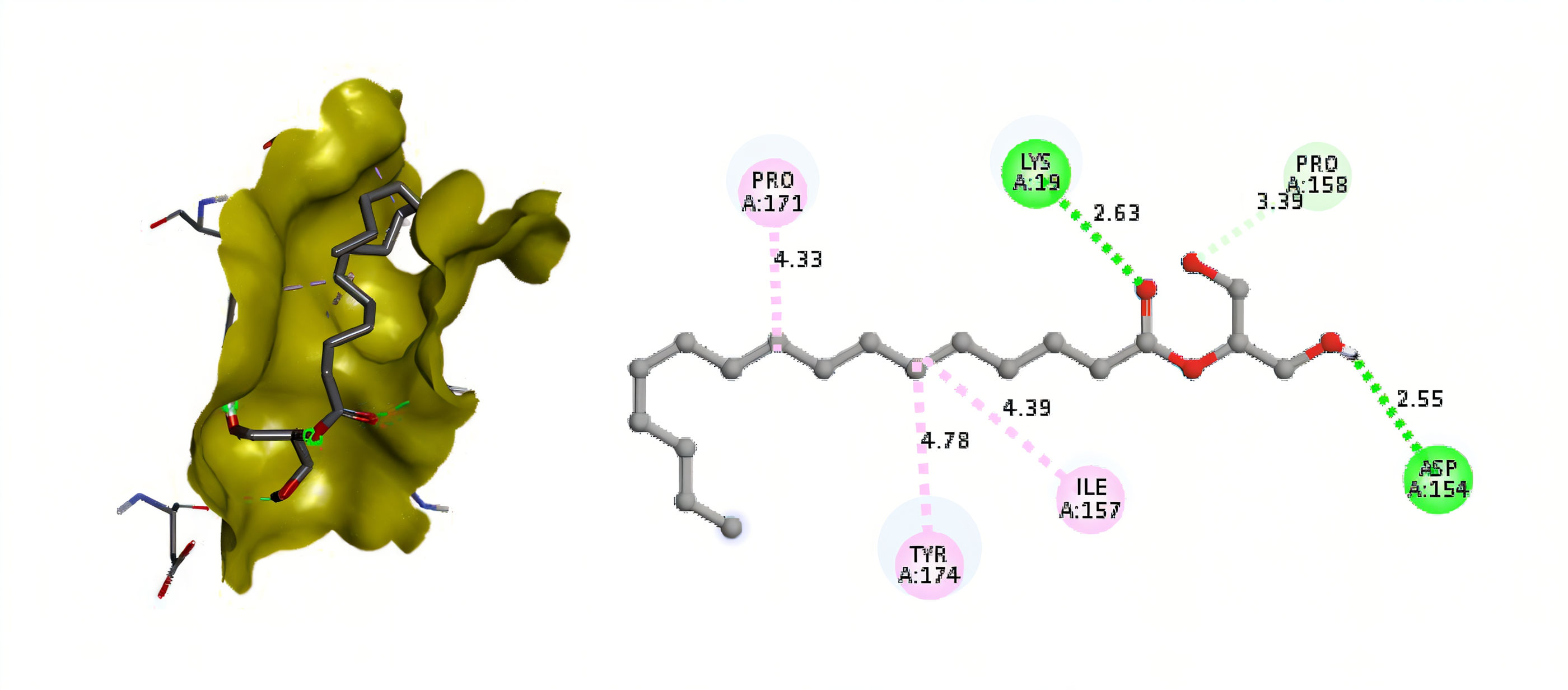

Supplement: Supplementary file 1 [file biomedicines-13-01658-s001.zip › Docking Interaction Images/CDK6_2-palmitoylglycerol.jpg]

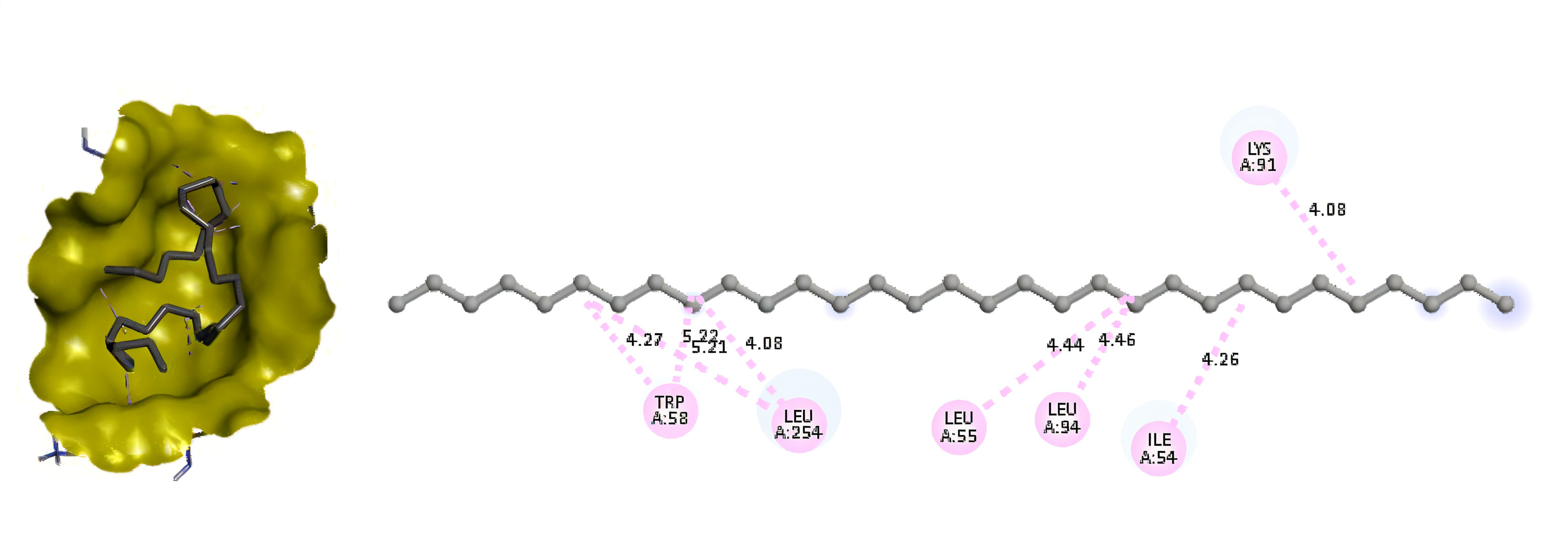

Supplement: Supplementary file 1 [file biomedicines-13-01658-s001.zip › Docking Interaction Images/CDK6_hentriacontane.jpg]

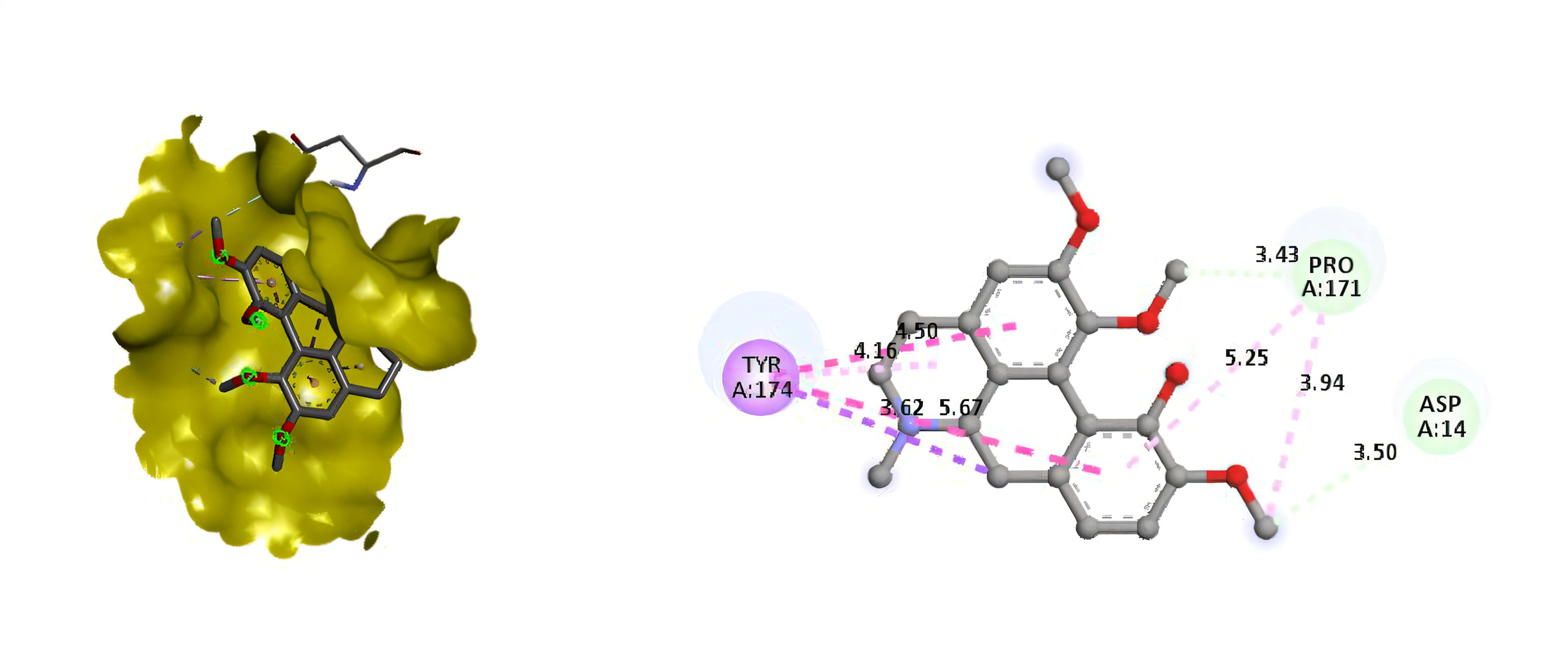

Supplement: Supplementary file 1 [file biomedicines-13-01658-s001.zip › Docking Interaction Images/CDK6_isocorydine.jpg]

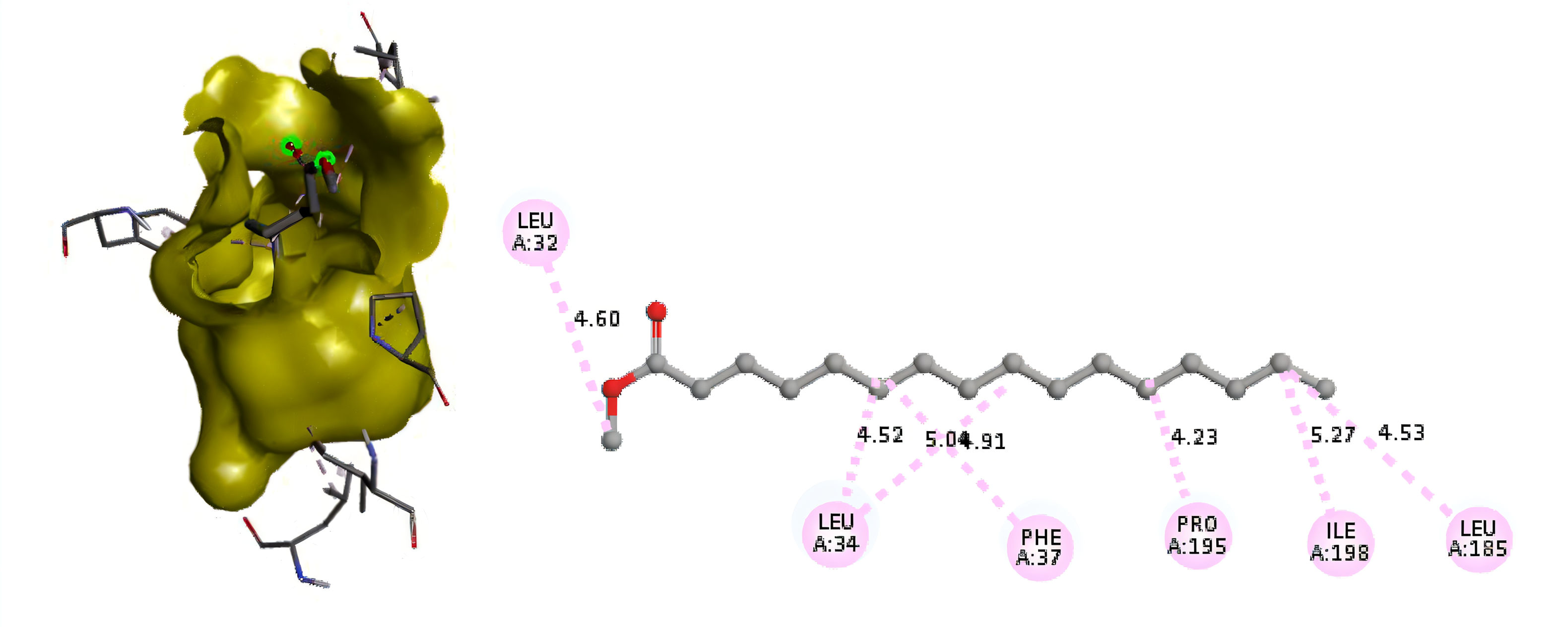

Supplement: Supplementary file 1 [file biomedicines-13-01658-s001.zip › Docking Interaction Images/CDK6_methylpalmitate.jpg]

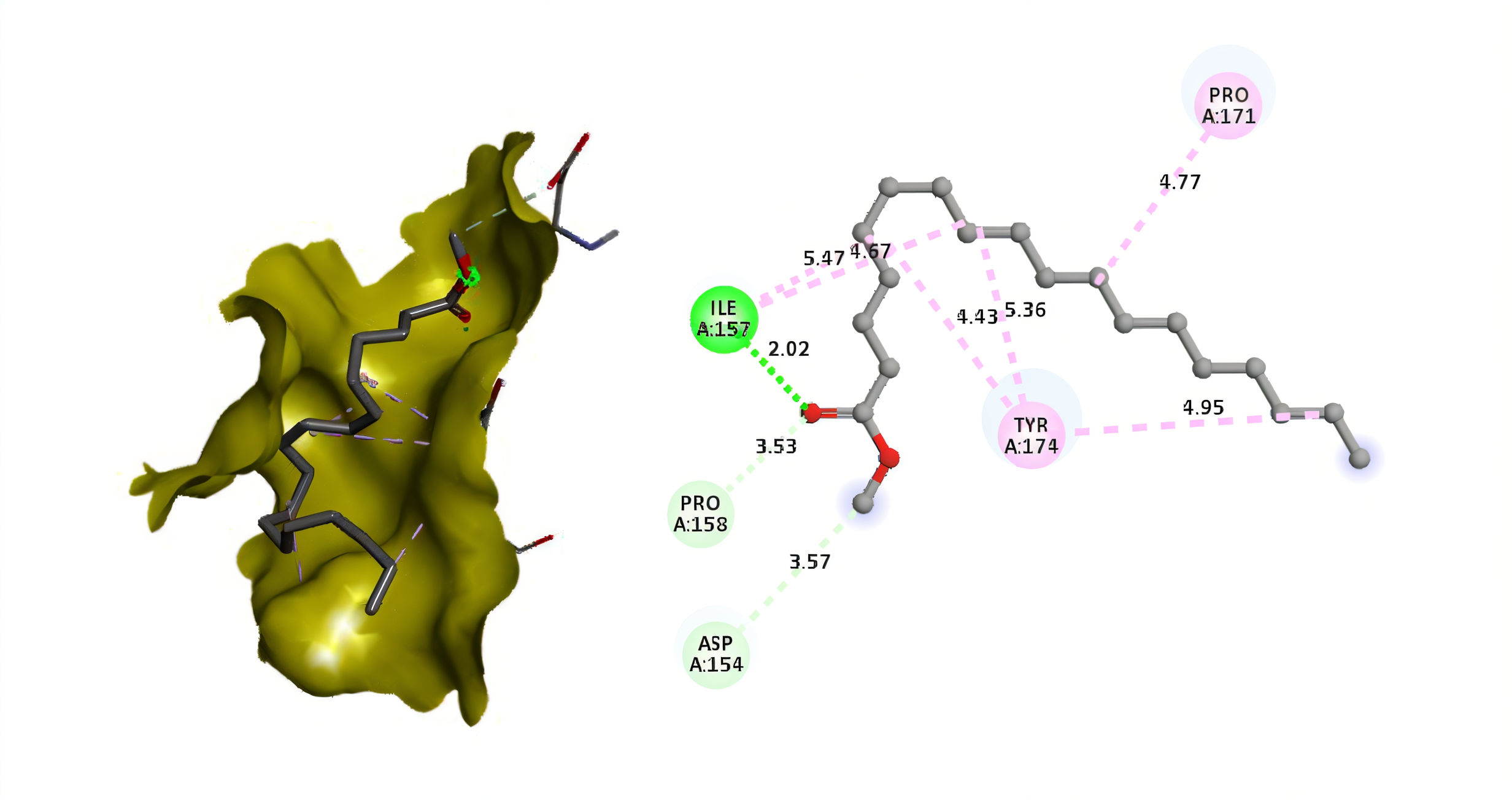

Supplement: Supplementary file 1 [file biomedicines-13-01658-s001.zip › Docking Interaction Images/CDK6_methylstearate.jpg]

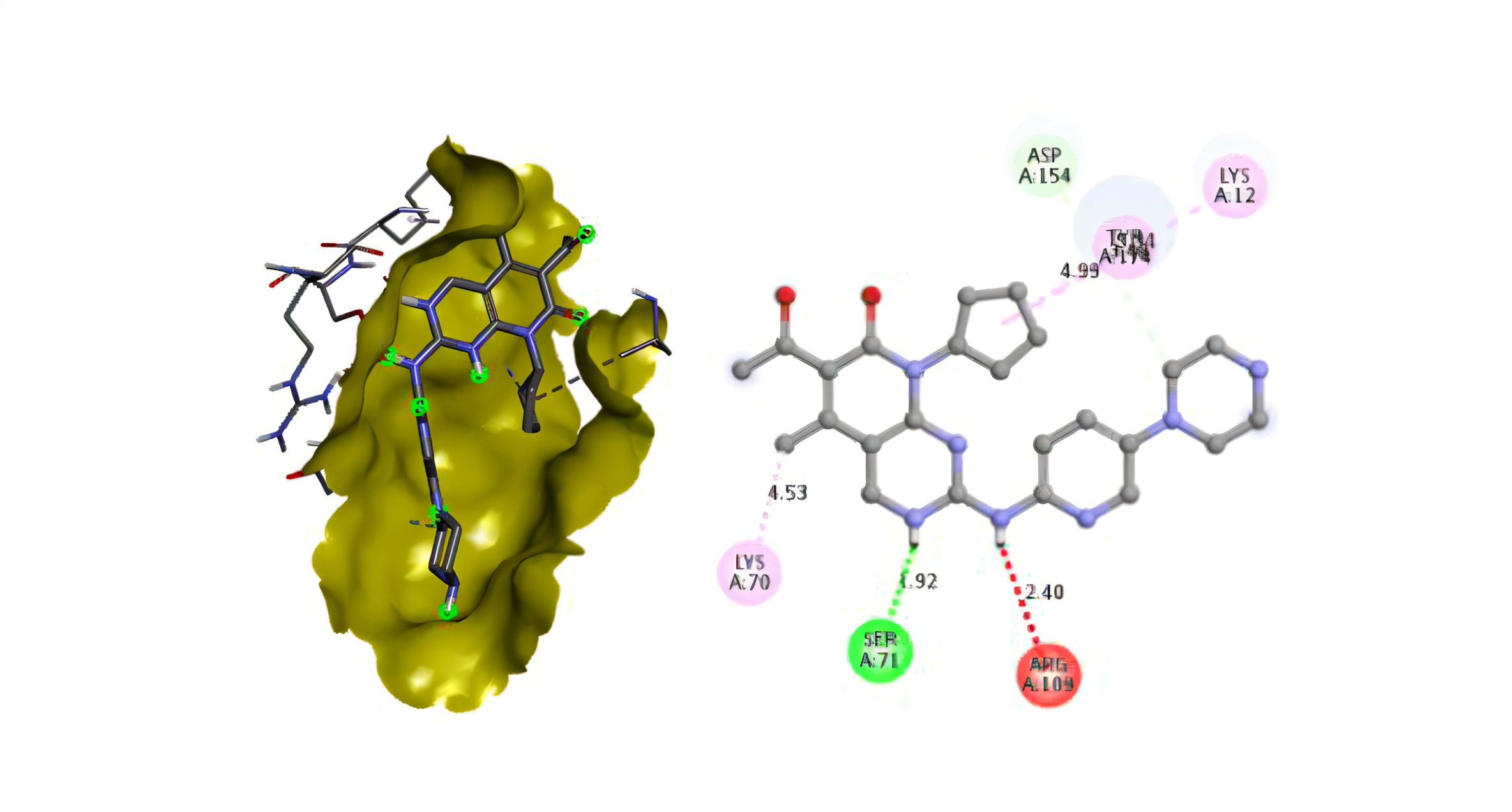

Supplement: Supplementary file 1 [file biomedicines-13-01658-s001.zip › Docking Interaction Images/CDK6_palbociclib.jpg]

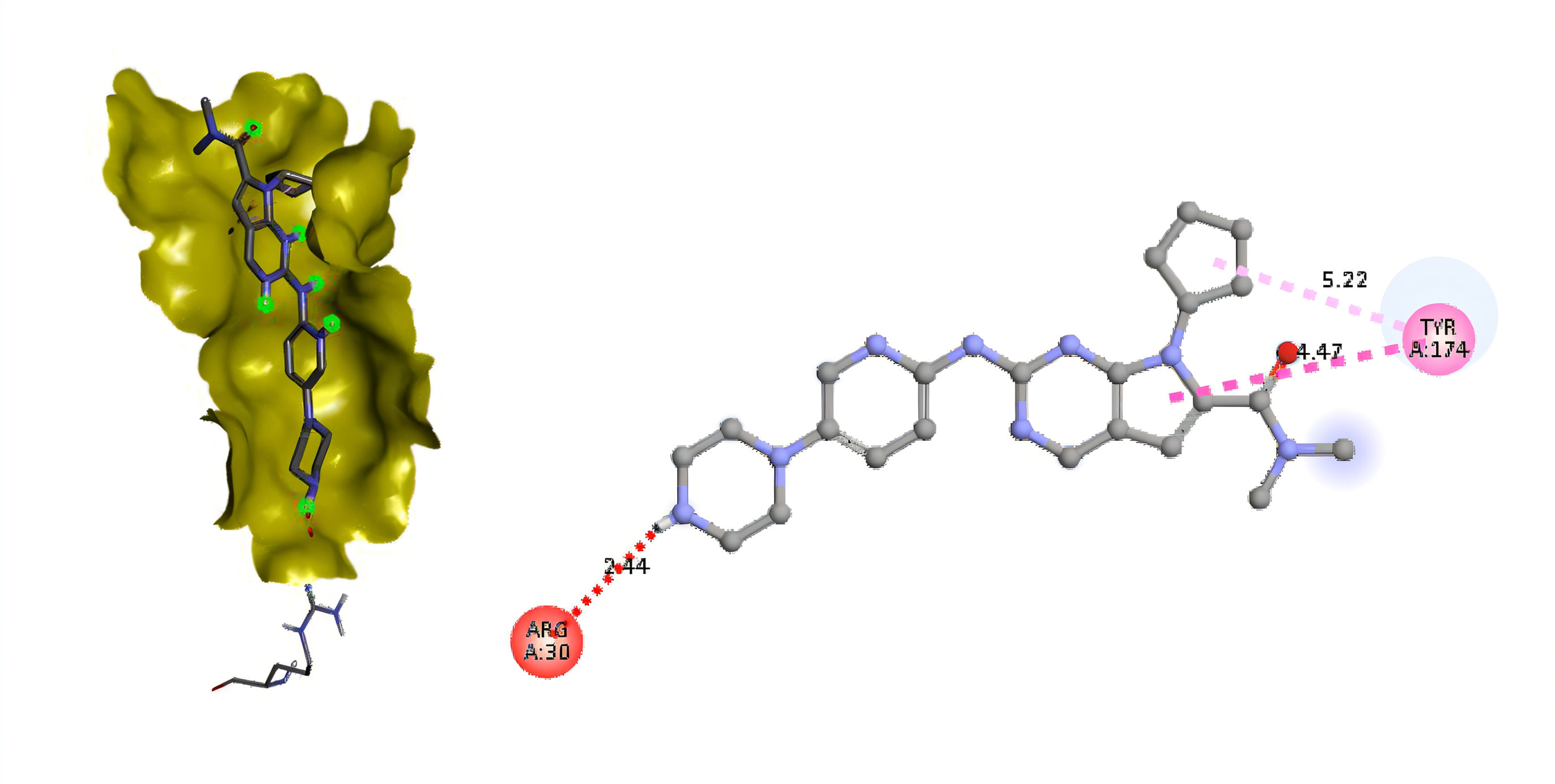

Supplement: Supplementary file 1 [file biomedicines-13-01658-s001.zip › Docking Interaction Images/CDK6_ribociclib.jpg]

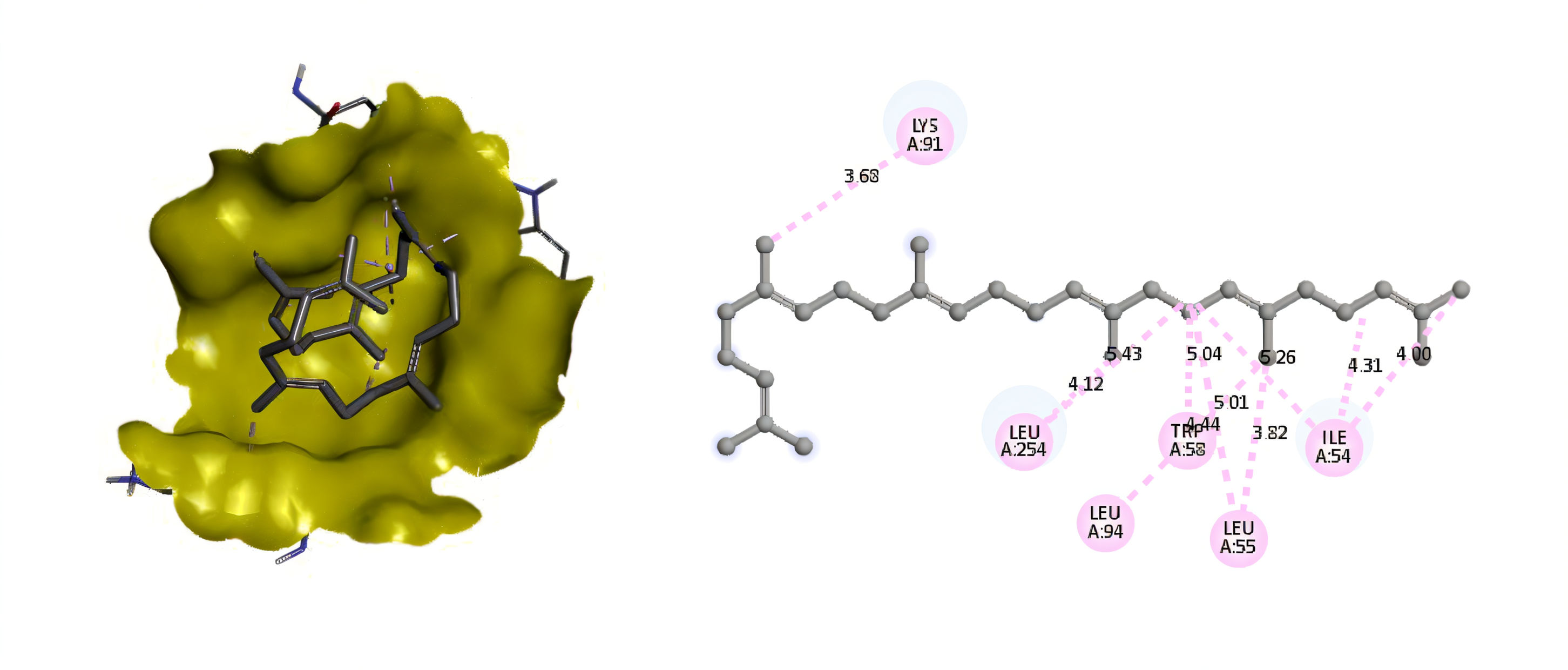

Supplement: Supplementary file 1 [file biomedicines-13-01658-s001.zip › Docking Interaction Images/CDK6_squalene.jpg]

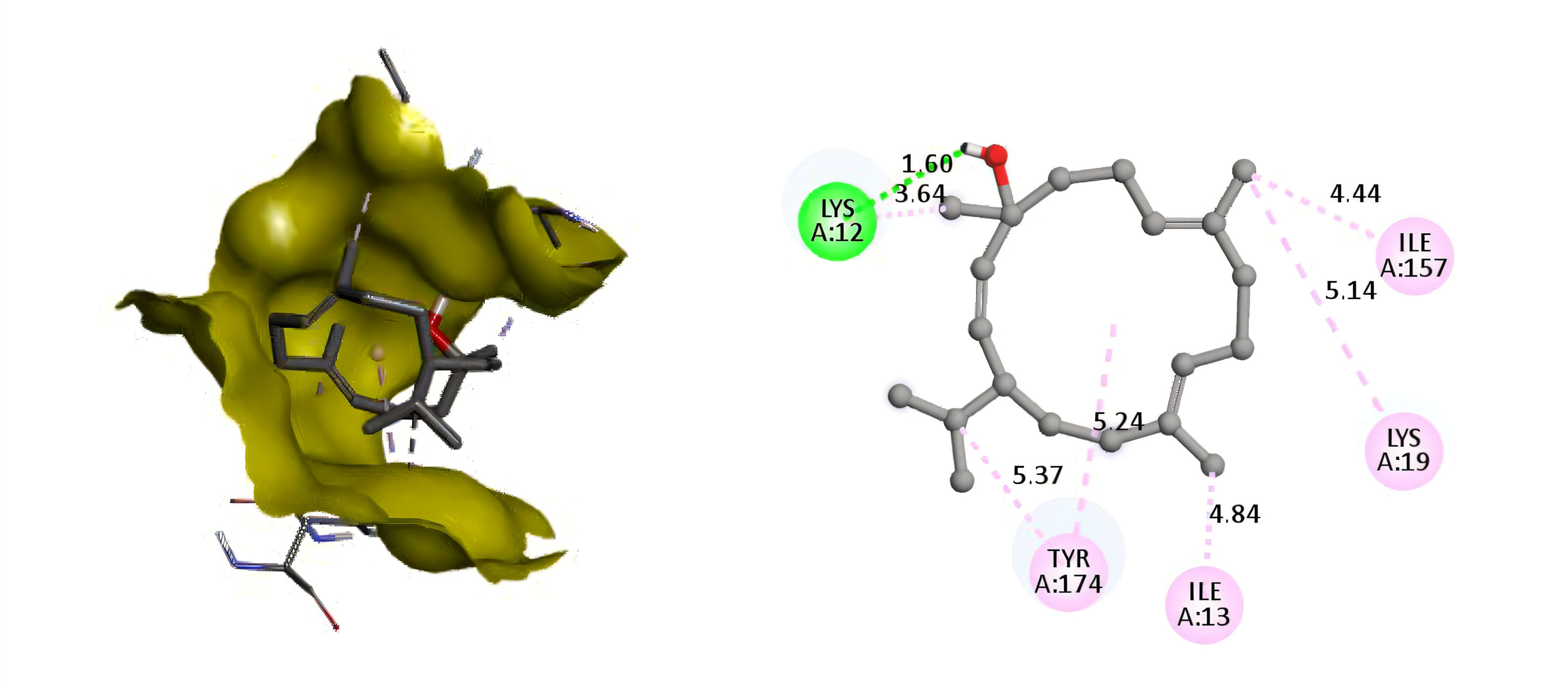

Supplement: Supplementary file 1 [file biomedicines-13-01658-s001.zip › Docking Interaction Images/CDK6_thunbergol.jpg]

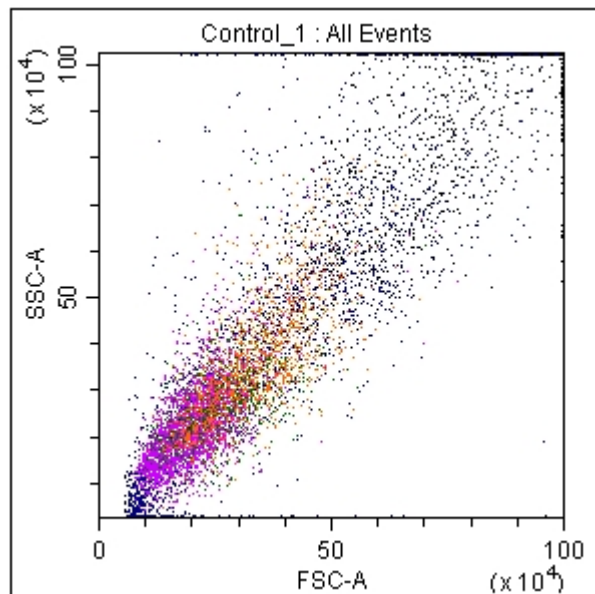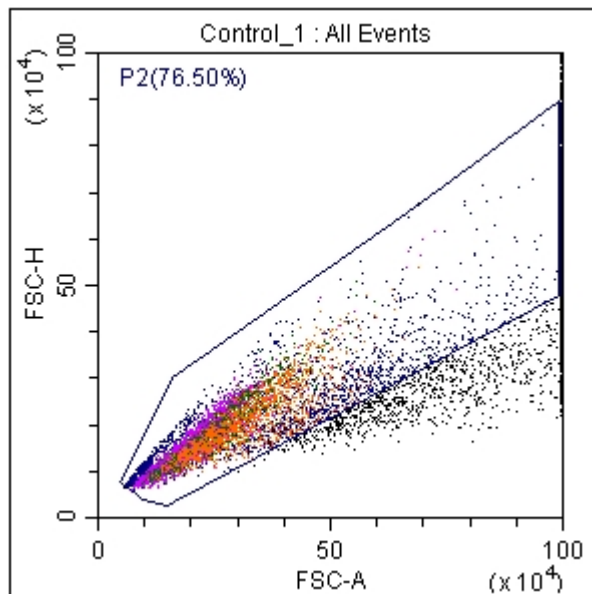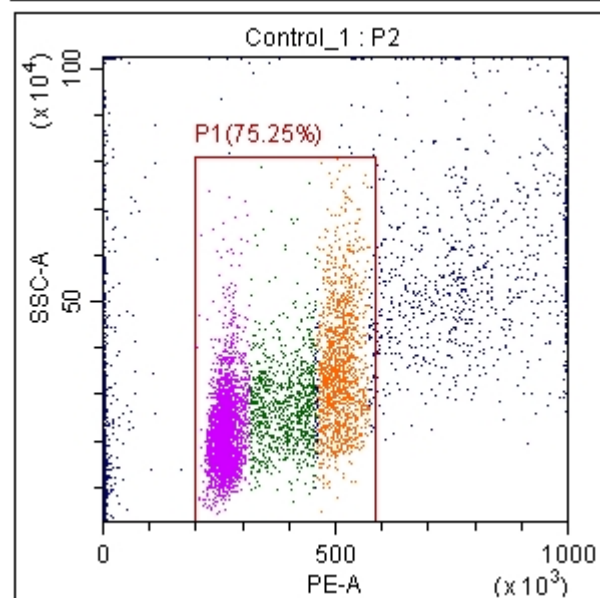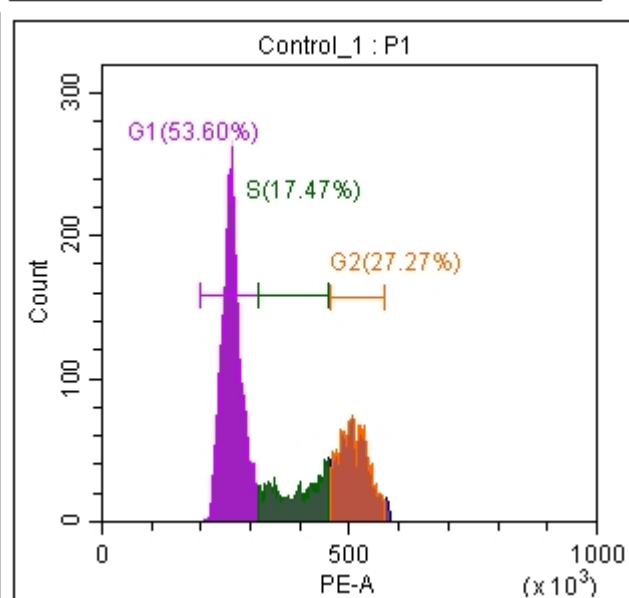

| Population   | Mean PE-A | Events | % Parent |
|--------------|-----------|--------|----------|
| ● All Events | 869519.6  | 8741   | 100.00%  |
| ● P1         | 355336.7  | 5032   | 75.25%   |
| ● P2         | 384521.3  | 6687   | 76.50%   |
| ● G1         | 261580.8  | 2697   | 53.60%   |
| ● S          | 389128.3  | 879    | 17.47%   |
| ● G2         | 507962.4  | 1372   | 27.27%   |

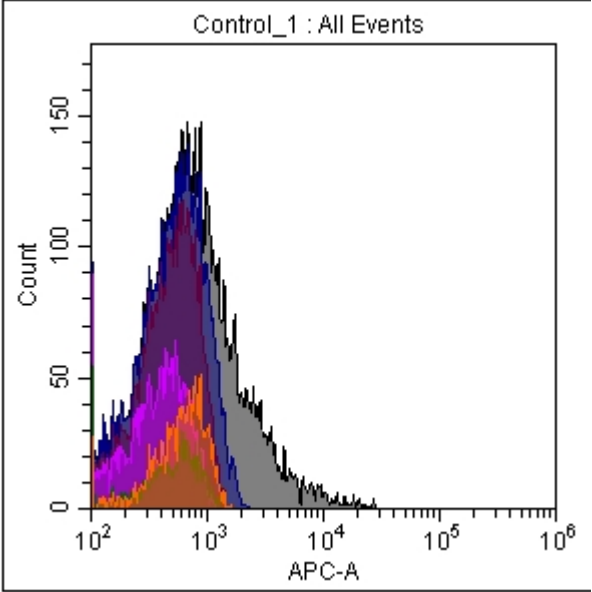

1

Supplement: Supplementary file 1 [file biomedicines-13-01658-s001.zip › Flow_Cytometry_Control_1.pdf]

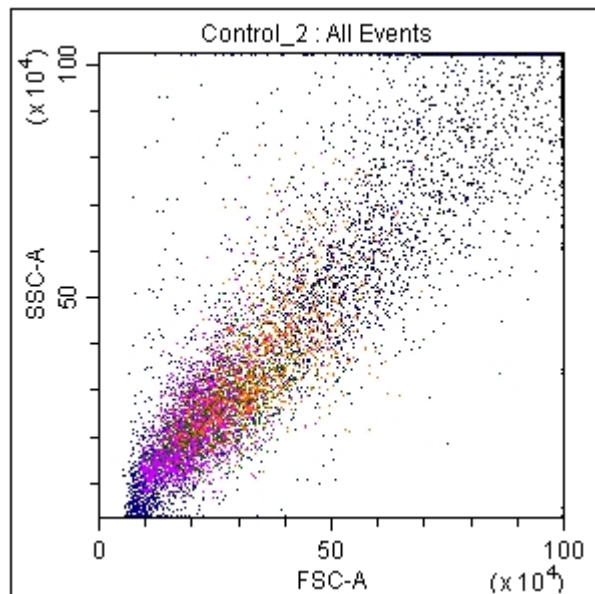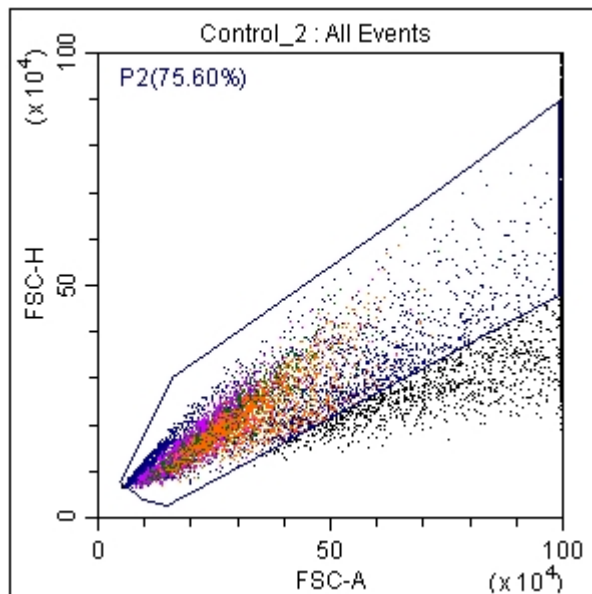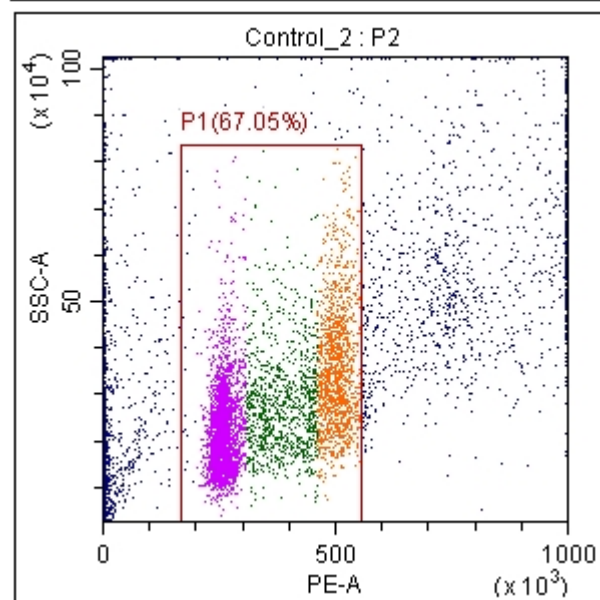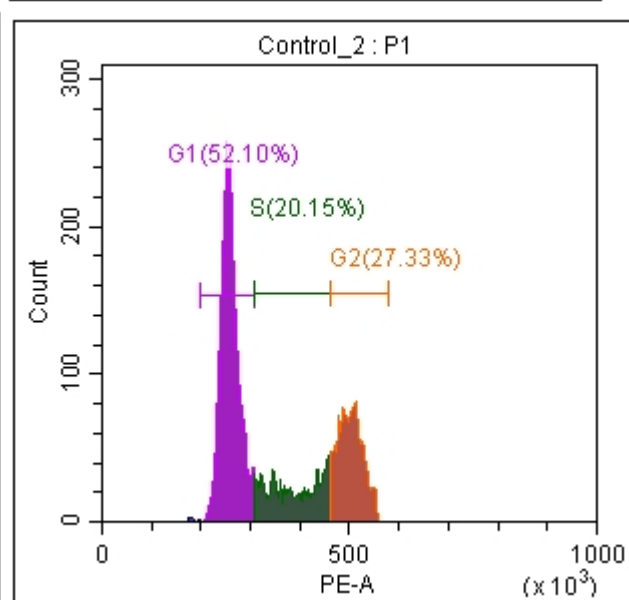

| Population   | Mean PE-A | Events | % Parent |
|--------------|-----------|--------|----------|
| ● All Events | 840687.4  | 9723   | 100.00%  |
| ● P1         | 349761.7  | 4929   | 67.05%   |
| ● P2         | 365684.6  | 7351   | 75.60%   |
| ● G1         | 257664.5  | 2568   | 52.10%   |
| ● S          | 386113.7  | 993    | 20.15%   |
| ● G2         | 501143.4  | 1347   | 27.33%   |

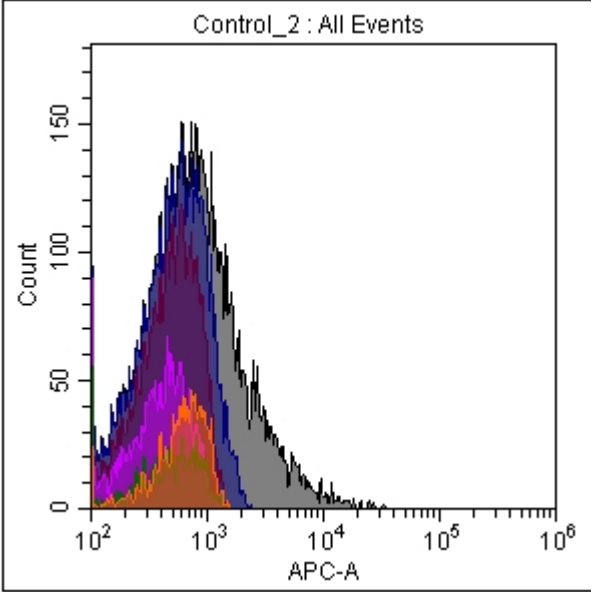

1

Supplement: Supplementary file 1 [file biomedicines-13-01658-s001.zip › Flow_Cytometry_Control_2.pdf]

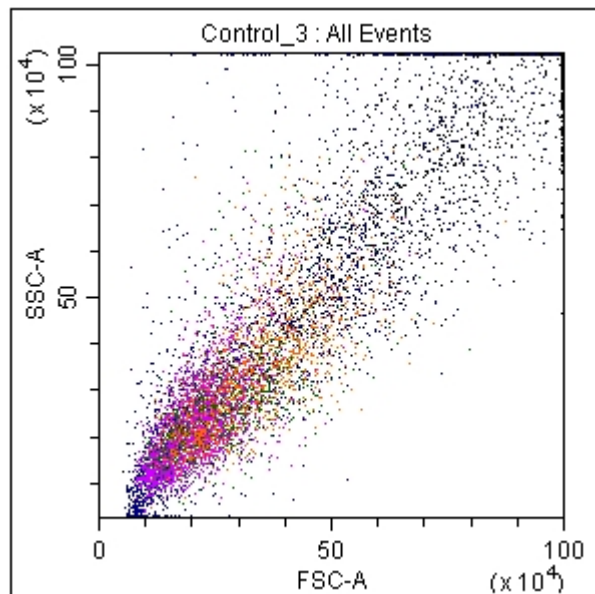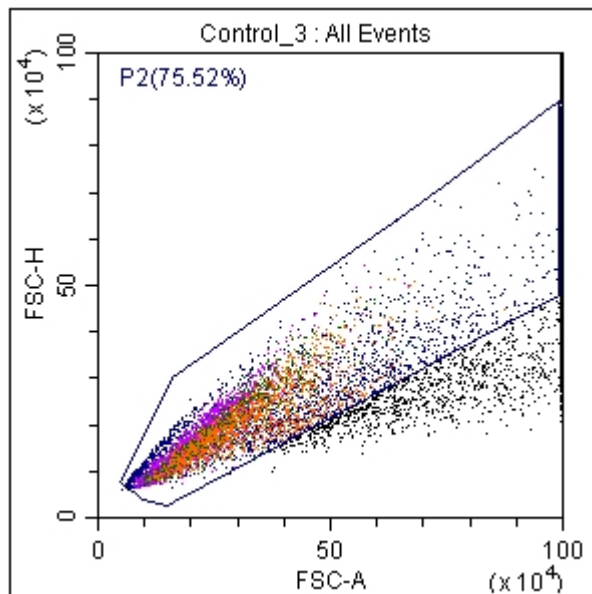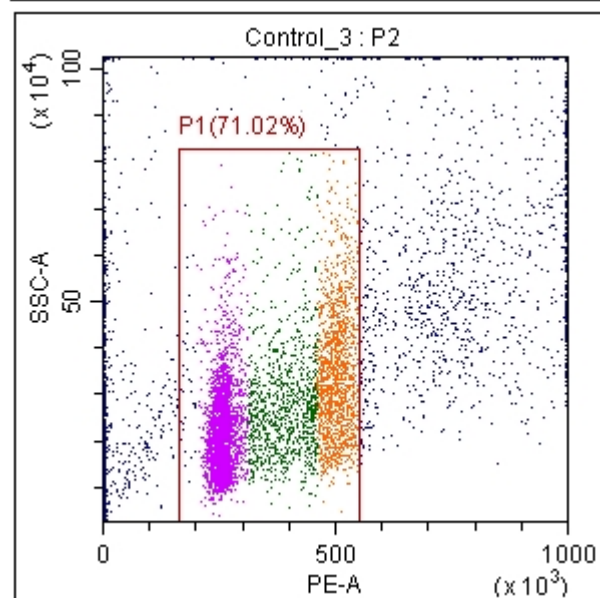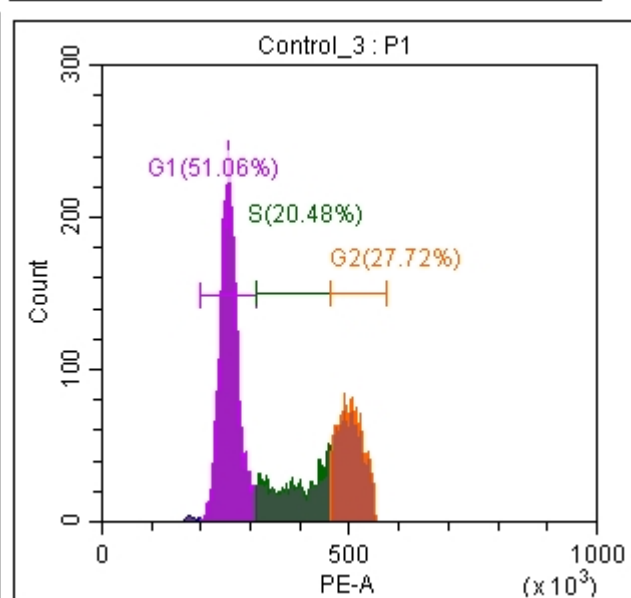

| Population   | Mean PE-A | Events | % Parent |
|--------------|-----------|--------|----------|
| ● All Events | 819681.9  | 9193   | 100.00%  |
| ● P1         | 351086.6  | 4931   | 71.02%   |
| ● P2         | 383506.1  | 6943   | 75.52%   |
| ● G1         | 256557.1  | 2518   | 51.06%   |
| ● S          | 390371.4  | 1010   | 20.48%   |
| ● G2         | 500658.4  | 1367   | 27.72%   |

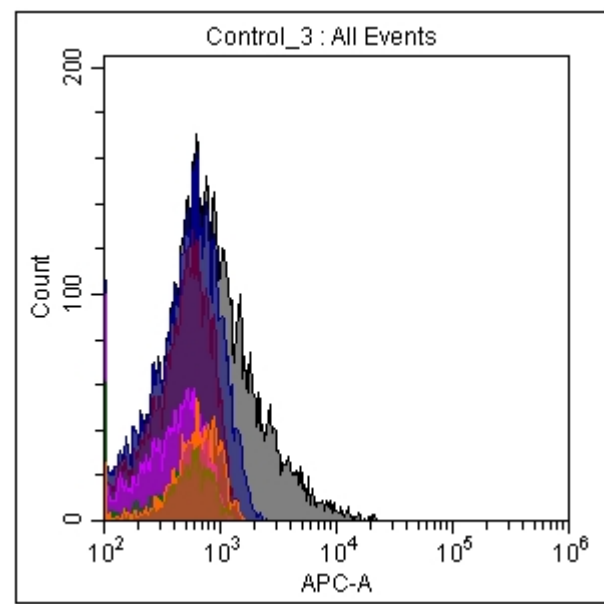

1

Supplement: Supplementary file 1 [file biomedicines-13-01658-s001.zip › Flow_Cytometry_Control_3.pdf]

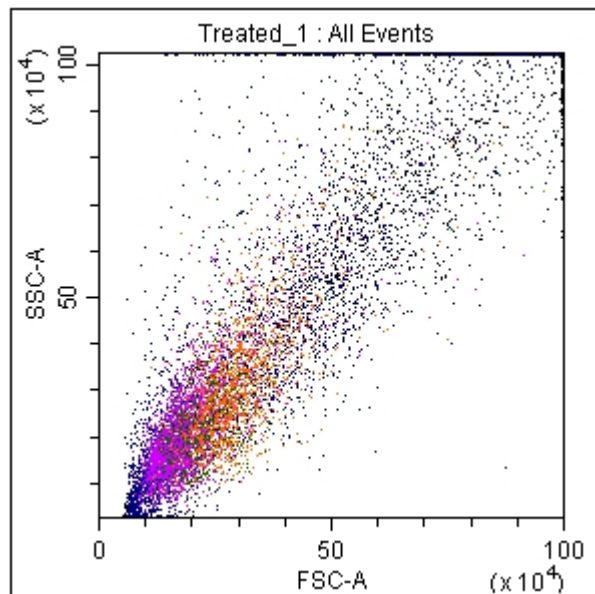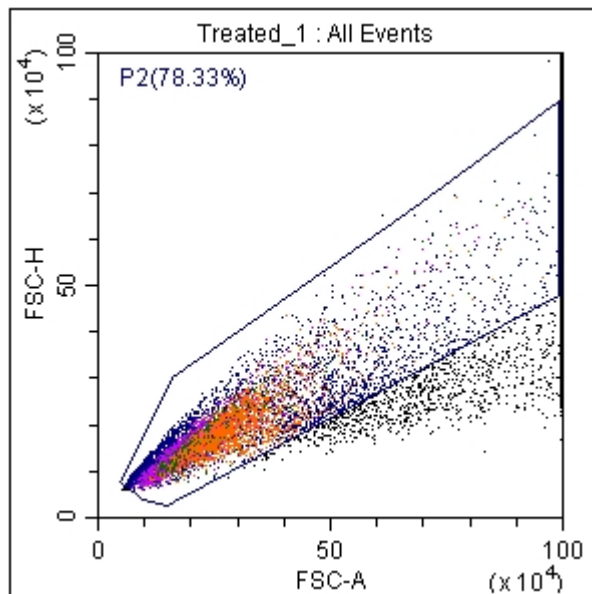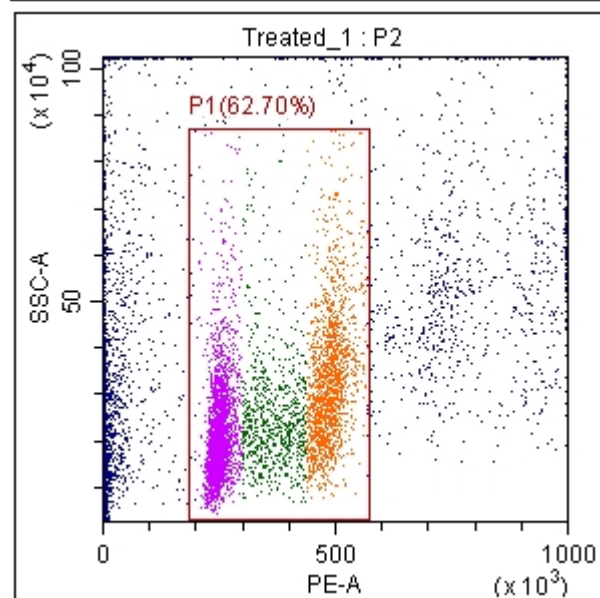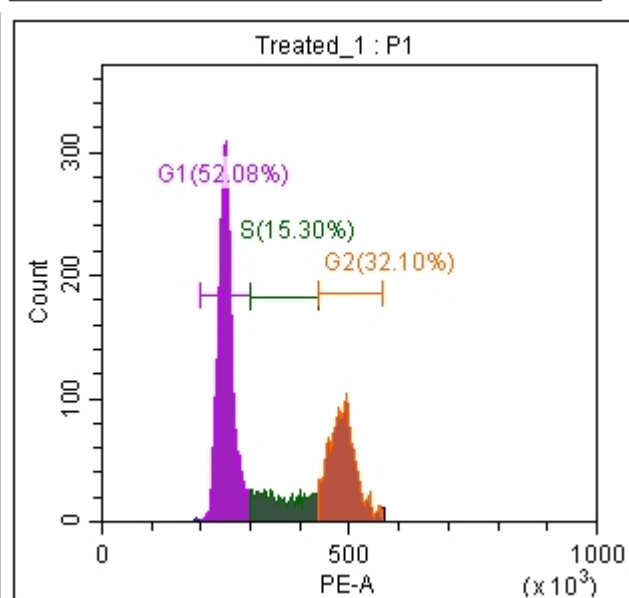

| Population   | Mean PE-A | Events | % Parent |
|--------------|-----------|--------|----------|
| ● All Events | 861430.6  | 10231  | 100.00%  |
| ● P1         | 343740.3  | 5025   | 62.70%   |
| ● P2         | 319692.3  | 8014   | 78.33%   |
| ● G1         | 249701.8  | 2617   | 52.08%   |
| ● S          | 364936.8  | 769    | 15.30%   |
| ● G2         | 485544.3  | 1613   | 32.10%   |

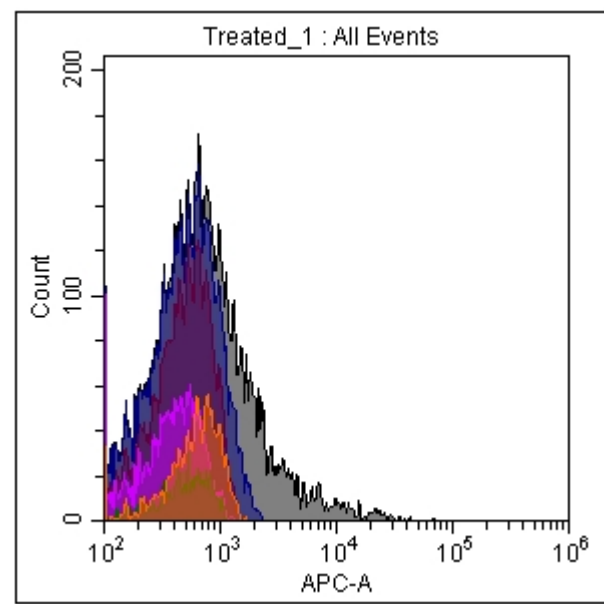

1

Supplement: Supplementary file 1 [file biomedicines-13-01658-s001.zip › Flow_Cytometry_Treated_1.pdf]

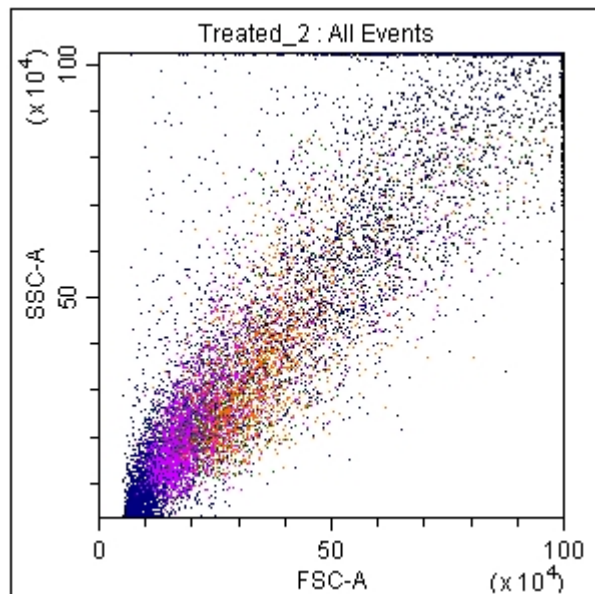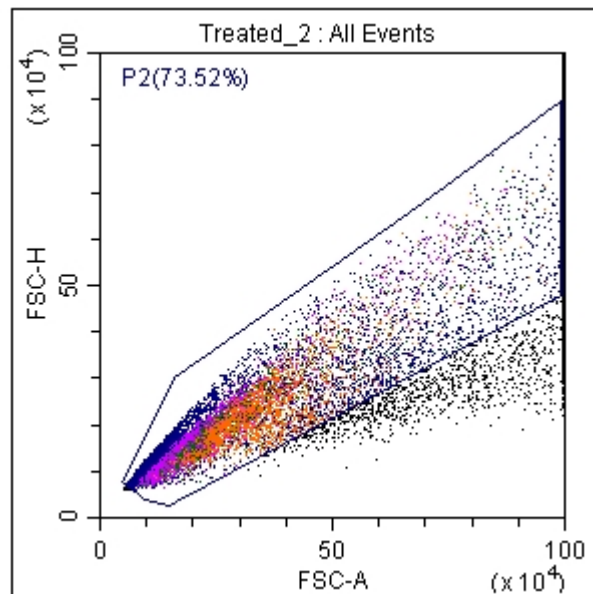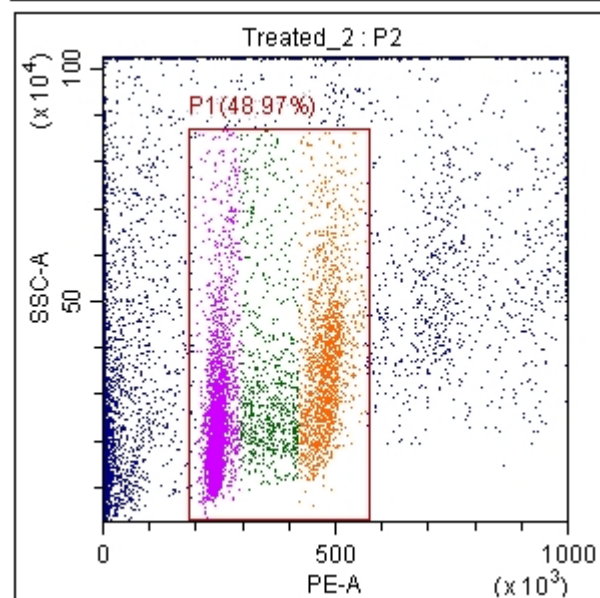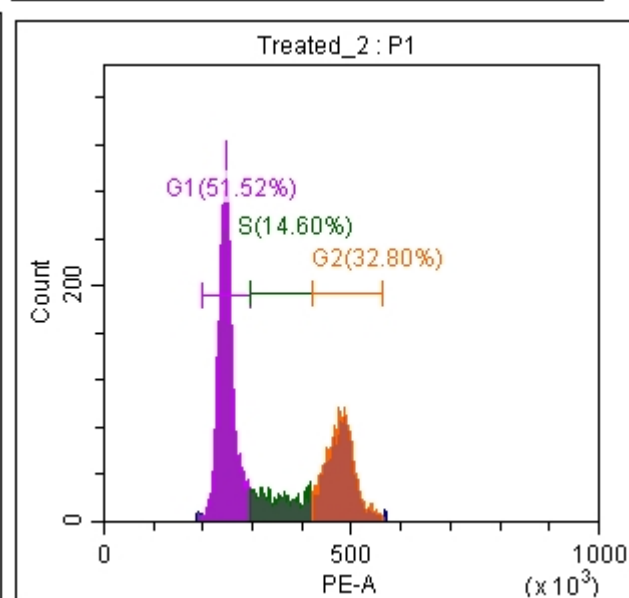

| Population   | Mean PE-A | Events | % Parent |
|--------------|-----------|--------|----------|
| ● All Events | 944238.4  | 13888  | 100.00%  |
| ● P1         | 338975.9  | 5000   | 48.97%   |
| ● P2         | 260799.4  | 10211  | 73.52%   |
| ● G1         | 246620.3  | 2576   | 51.52%   |
| ● S          | 354841.9  | 730    | 14.60%   |
| ● G2         | 477037.1  | 1640   | 32.80%   |

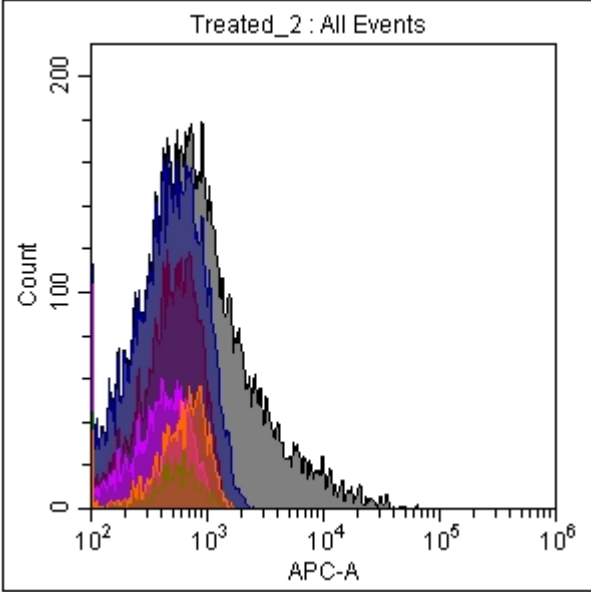

1

Supplement: Supplementary file 1 [file biomedicines-13-01658-s001.zip › Flow_Cytometry_Treated_2.pdf]

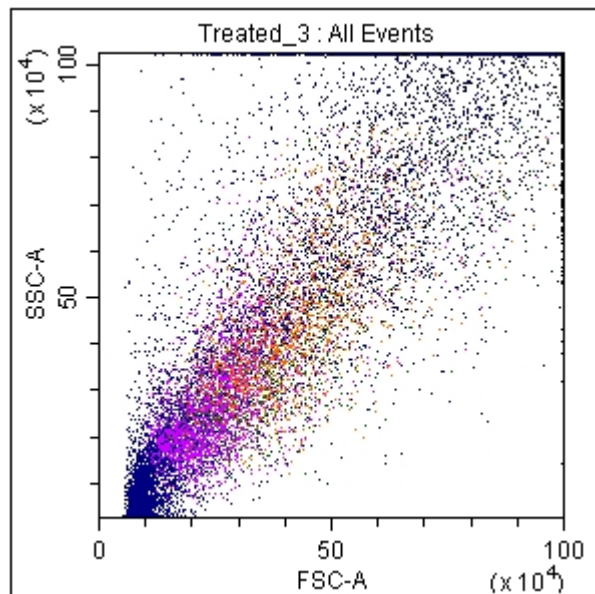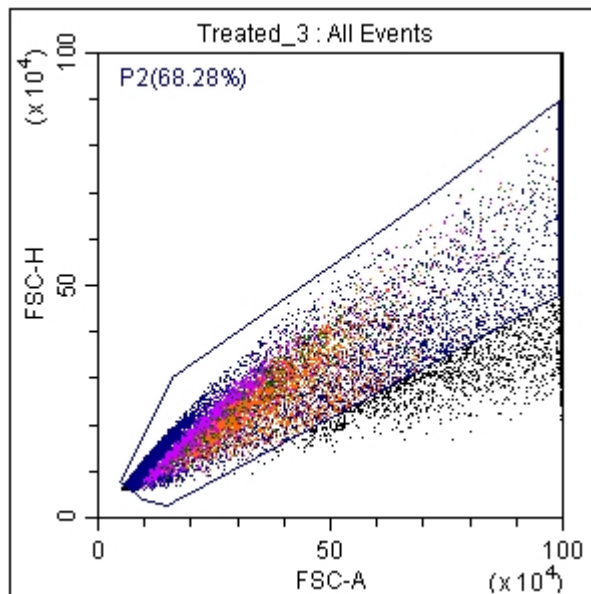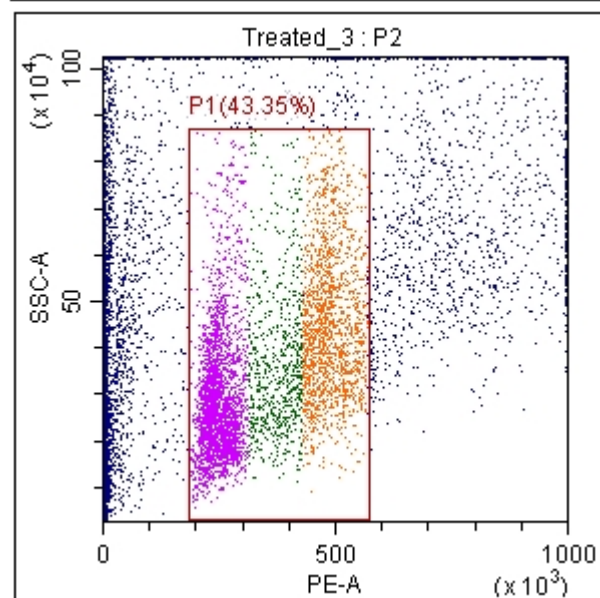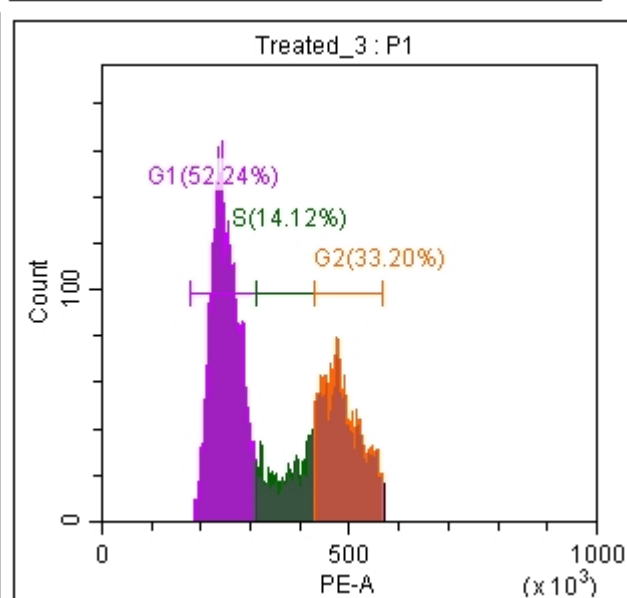

| Population   | Mean PE-A | Events | % Parent |
|--------------|-----------|--------|----------|
| ● All Events | 1110304.8 | 16891  | 100.00%  |
| ● P1         | 347193.6  | 5000   | 43.35%   |
| ● P2         | 254118.3  | 11533  | 68.28%   |
| ● G1         | 249029.1  | 2612   | 52.24%   |
| ● S          | 374428.4  | 706    | 14.12%   |
| ● G2         | 485828.6  | 1660   | 33.20%   |

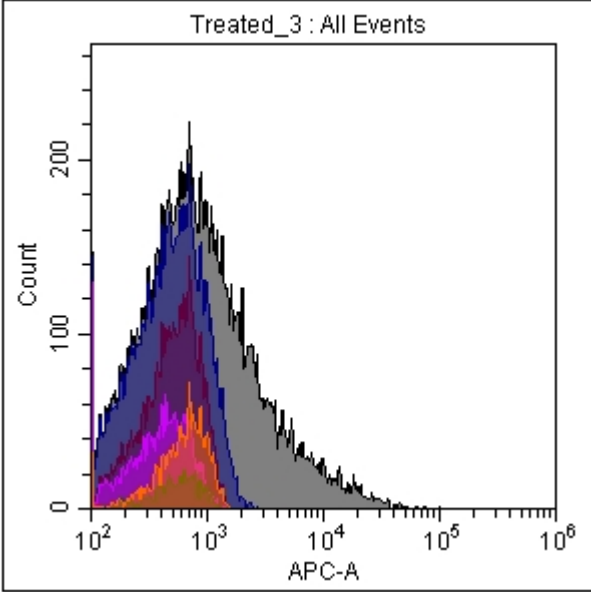

1

Supplement: Supplementary file 1 [file biomedicines-13-01658-s001.zip › Flow_Cytometry_Treated_3.pdf]
